# Supplementary material for: Effects of cerebral near-infrared spectroscopy on the outcome of patients undergoing cardiac surgery: a systematic review of randomised trials
Source: BMJ Open. 2017 Sep 7;7(9):e016613. doi: 10.1136/bmjopen-2017-016613 (PMC5595187; doi:10.1136/bmjopen-2017-016613)
Supplement: Supplementary file 1 [file bmjopen-2017-016613supp001.pdf]

Online only digital supplement: Efficacy of Near-Infrared Spectroscopy on the outcome of patients undergoing cardiac surgery: a systematic review of randomised trials.

Authors: GF Serraino, GJ Murphy

Table S1. Characteristics of Included Studies.

STUDY	*Location/ Funding*	*Sample size*	*Period*	*Inclusion/ Exclusion Criteria*	*Mean Age*<br>*-intervention group*<br>*-control group*<br>*Female (%)*<br>*- Intervention group*<br>*-control group*	*NIRS Device*	*Murkin Algorithm*	*Control*	*Outcomes*
Rogers, 2017[36]	3 UK centres<br>NIHR funded	204	2009-2014	Incl: CABG and non CABG.<br>Excl: Patients undergoing emergency cardiac surgery<br>Patients who are prevented from having blood and blood products according to a system of beliefs.	70±10<br>65.9±12.3<br>30%F<br>33%F	INVOS		SC	Primary: Cognitive function<br>Secondary: Units of RBC; Cerebral oxygenation; Oxygen delivery and utilisation during CPB: Quality of life; Infectious complications; Stroke; MI; ICU and H LOS; Post-operative acute kidney injury; Respiratory complications; Health and personal social services resource use and their costs. All-cause mortality within 30 days of surgery; Biochemical markers of organ injury

Deschamps, 2016 [29]	Multicentre<br>Regional funders<br>Canada	201	2012<br>–<br>2013	Incl: high-risk cardiac surgery<br>Excl: Off -pump coronary artery bypass surgery, emergency surgery, planned deep hypothermic circulatory arrest, acute endocarditis, presence of active delirium or encephalopathy.	69±12.6<br>72±9.4<br>27.4%F<br>28.3%F	FORE-SIGHT, EQUANOX Classic 7600, INVOS 5100C-PB		SC	Primary Outcome: success rate of reversing cerebral desaturations below 10% relative to baseline in the intervention group<br>Secondary Outcomes: cerebral desaturation load, 30-day follow-up for adverse events
Kara, 2015 [32]	Single centre,<br>Sakarya, Turkey	79	2013-2015	Incl: CABG<br>Excl: Other procedures, high degrees of aortic atherosclerosis	59.1±9.4<br>61.2±10.3<br>23.3%F<br>19.4%F	INVOS		SC	Primary: Cognitive impairement<br>(MoCa score)<br>Secondary: ICU and H LOS
Colak, 2015[28]	Single centre, Zagreb,<br>Croatia<br>Insitutional support grant	200	2009-2010	Incl: CABG<br>Excl: carotid artery stenosis, previous stroke or head injury, seizure, psychiatric illness, (NYHA III/IV) LVEF<25%, emergency off-pump CABG and severely impaired renal and liver function.	61.9±7.1<br>63.4±8.8<br>20%F<br>24%F	INVOS		SC	Primary: Cognitive impairement<br>MMSE<br>CTT1<br>GP test<br>Secondary: coma, stupor, transient ischaemic attack (TIA) or stroke, neuropsychological deficits
Deschamps, 2013[30]	Single centre, Quebec, Canada. Insitutional support grant	49	NS	Incl: CABG and non-CABG<br>Excl: Emergency surgery, first time CABG surgery, single valve surgery	70.2±9.2<br>71.1±7.9<br>15%F<br>15%F	INVOS	X	SC	Primary: rSO2<br>Secondary: ICU and H LOS

Vretzakis, 2013[35]	Single centre, Greece<br>Insitutional support grant	50	16-month period	Incl: CABG and non CABG<br><br>Excl: Emergency, re-do operations, combined cardiac - carotid surgery circulatory arrest. hematologic disease coagulation abnormality, advanced cirrhosis and renal dysfunction	67.3±8.5<br>65.9±9.5<br><br>12%F<br>15%F	INVOS		SC	Primary: RBC transfusion,<br>ICU and H LOS<br>Death<br><br>Secondary: Major complications
Mohandas, 2013[33]	Single centre, Bangalore, India<br>RCT	100	NS	Incl: On pump<br>Cardiac surgery<br><br>Excl: pre-existing neuropsychiatric disorders, inability to correctly perform the neurocognitive tests, and mini-mental state examination (MMSE) scores of less than 23	38.05±15.81<br>34.60±16.28<br><br>44%F<br>44%F	NONIN EQUANOX	X	SC	Primary: Cognitive decline:<br>MMSE<br>ASEM<br><br>Secondary: ICU LOS
Harilall, 2013[31]	Single centre, South Africa	40	NS	Incl: CABG<br><br>Excl: pregnancy, history of stroke or per- sistent neurological residue, history of transient ischaemic attack (TIA), stenosis of carotid artery	55.3±9.7<br><br>30%F (all)	INVOS	X	SC	Primary: rSO2<br><br>Secondary: S100B

Slater, 2008[34]	Single centre, New Jersey, USA	265	2004-2006	Incl: CABG<br><br>Excl: preexisting neuropsychiatric disorders, inability to correctly perform the neurocognitive tests, and mini- mental state examination score of 23 or less.	64.8±10.1<br>64.78±9.9<br><br>31%F (all)	INVOS	X	SC	Primary: Cognitive impairement<br><br>Secondary: ICU LOS
Murkin, 2007[10]	Single centre, Ontario, Canada<br>Grant and other support from the device manufacturer.	200	2002 – 2004	Incl: >18 yr, CABG	61.8±10.3<br>61.8±9.3<br><br>12.5%F (all)	INVOS	X	SC	Primary: 30 days Death, MMOM<br><br>Secondary: LOS

Table S2. Characteristics of Excluded Studies

STUDY	*Study Type/ Location*	*SAMPLE*	*PERIOD*	*INCLUS. CRIT.*	*EXCLUS. CRIT.*	*Mean Age*	*Female (%)*	*NIRS Device*	*Murkin Algorithm*	*Control*	*OUTCOMES*
Dullenkpof, 2007 [40]	Prosp, RCT, Single centreZurich, Switzerland **NO intervention**	35	NS	Elective cardiac surgery with insertion of PAC	Cerebral perfusion disturbence, CAD, intracardiac shunt	65.5±10.9	74.2	INVOS			Primary: rSO2
Kok, 2014 [39]	Single centre Groningen NH	59	2011 – 2012	CABG	Other procedures, difficulty completing cognitive tests, difficulty with dutch lang, impaired finction of the dominant arm or hand, history of head trauma, stroke or neurosurgery, severe CAD	62.8±9.4	10	Somanetics (Michigan) Casmed (Branford)		On pump vs Off Pump surgery	Primary: Cerebral desaturation Secondary: Postop cognitive disfunction; major complications; ICU and Hospital LOS

PAC pulmonary artery catheter, CABG coronary artery bypass grafts, ICU intensive care unit, LOS length of stay

Table S3. Sub Group Analyses

SUB GROUP	CABG	NON CABG	TEST FOR SUBGROUP DIFFERENCES	MURKIN	NON MURKIN	TEST FOR SUBGROUP DIFFERENCES	CONSENSUS NEUROCOGNITIVE ASSESSMENT	NON CONSENSUS NEUROCOGNITIVE ASSESSMENT	TEST FOR SUBGROUP DIFFERENCES	RESTRICTIVE RED CELL TRANSFUSION TRIGGER	NO RESTRICTIVE RED CELL TRANSFUSION TRIGGER	TEST FOR SUBGROUP DIFFERENCES
**MORTALITY RR (95% CI)**	0.33 (0.01 to 8.09) 1 trial, n=200, I^2 =NA	0.81 (0.30 to 2.18) 3 trials, n=408, I^2=0%	**Chi^2 = 0.50, df = 3 (P = 0.92), I^2 = 0%**	0.77 (0.26 to 2.32) 2 trials, n=289, I2 =NA	0.67 (0.11 to 3.94) 2 trials, n=328, I^2=0%	**Chi^2 = 0.50, df = 3 (P = 0.92), I^2 = 0%**	0.50 (0.05 to 5.42) 1 trial, n=178 I2=NA	0.94 (0.39 to 2.26) 3 trials, n=430 I2=4%	**Chi^2 = 0.23, df = 3 (P = 0.97), I^2 = 0%**	0.67 (0.11 to 3.94) 2 trials, n=328 I2=0%	0.77 (0.26 to 2.32) 2 trials, n= 280 I2= NA	**Chi^2 = 0.50, df = 3 (P = 0.92), I^2 = 0%**
**STROKE RR (95% CI)**	1.18 (0.38 to 3.63) 3 trials, n=630. I2 =43%	0.89 (0.26 to 3.10) 4 trials, n= 508 I2=1%	**Chi^2 = 5.66, df = 6 (P = 0.46), I^2 = 0%**	0.84 (0.33 to 2.11) 5 trials, n=810 I2=14%	3.00 (0.32 to 28.54) 3 trials, n=328 I2=0%	**Chi^2 = 5.66, df = 6 (P = 0.46), I^2 = 0%**	3.00 (0.12 to 72.66) 1 trial, n=178 I2: NA	1.00 (0.39 to 2.56) 6 trials, n=960 I2=22%	**Chi^2 = 5.66, df = 6 (P = 0.46), I^2 = 0%**	3.00 (0.32 to 28.54) 2 trials, n=328 I2=0%	0.84 (0.33 to 2.11) 5 trials, n=810 I2=14%	**Chi^2 = 5.66, df = 6 (P = 0.46), I^2 = 0%**
**MI RR (95% CI)**	0.92 (0.40 to 2.10) 3 trials, n=630, I2=0%	0.75 (0.17 to 3.30) 3 trials, n=408, I2=0%	**Chi^2 = 0.05, df = 1 (P = 0.82), I^2 = 0%**	0.92 (0.40 to 2.10) 4 trials, n=710, I2=0%	0.75 (0.17 to 3.30) 2 trials, n=328 I2=0%	**Chi^2 = 0.05, df = 1 (P = 0.82), I^2 = 0%**	0.50 (0.05 to 5.42) 1 trial, n=178 I2= NA	0.93 (0.43 to 1.99) 5 trials, n=860 I2=0%	**Chi^2 = 0.24, df = 1 (P = 0.63), I^2 = 0%**	0.75 (0.17 to 3.30) 2 trials, n=328 I2=0%	0.92 (0.40 to 2.10) 4 trials, n=710 I2=0%	**Chi^2 = 0.05, df = 1 (P = 0.82), I^2 = 0%**
**SEVERE AKI RR (95% CI)**	0.58 (0.08 to 4.41) 3 trials, n=630, I2=0%	0.88 (0.51 to 1.12) 4 trials, n=434, I2=0%	**Chi^2 = 0.15, df = 1 (P = 0.70), I^2 = 0%**	0.90 (0.51 to 0.19) 4 trials, n=710 I2=0%	0.67 (0.18 to 2.56) 2 trials, n=354 I2=12%	**Chi^2 = 0.15, df = 1 (P = 0.70), I^2 = 0%**	3.00 (0.12 to 72.49) 1 trial, n=150 I2=NA	0.96 (0.55 to 1.68) 3 trials, n=780 I2=0%	**Chi^2 = 0.48, df = 1 (P = 0.49), I^2 = 0%**	0.67 (0.18 to 2.56) 2 trials, n=354 I2= 12%	0.90 (0.51 to 1.19) 2 trials, n=630 I2=0%	**Chi^2 = 0.15, df = 1 (P = 0.70), I^2 = 0%**

**TRANSFUSION RR (95% CI)**	1.04 (0.89 to 1.23) 2 trials, n=390. I2=0%	0.85 (0.71 to 1.01) 2 trials, n=354. I2=0%	**Chi² = 2.81, df = 1 (P = 0.09), I² = 64.4%**	1.04 (0.89 to 1.23) 2 trials, n=390. I2=0%	0.85 (0.71 to 1.01) 2 trials, n=354 I2=0%	**Chi² = 2.81, df = 1 (P = 0.09), I² = 64.4%**	0.91 (0.65 to 1.28) 1 trial, n=150. I2=NA	0.94 (0.83 to 1.06) 5 trials, n=860 I2=66%	**Chi² = 0.04, df = 1 (P = 0.85), I² = 0%**	0.85 (0.71 to 1.01) 2 trials, n=354. I2= 0%	1.04 (0.89 to 1.23) 4 trials, n=710 I2=0%	**Chi² = 2.81, df = 1 (P = 0.09), I² = 64.4%**
**ICU LOS MD (95% CI)**	-0.39 (-0.71 to -0.06), 3 trials, n=469. I2=51%	-0.24 (-0.37 to -0.10), 5 trials, n=582. I2=82%	**Chi² = 0.70, df = 1 (P = 0.4), I² = 0%**	-0.19 (-0.35 to -0.03) 5 trials, n=618, I2=84%	-0.36 (-0.55 to -0.17) 3 trials, n=433, I2=0%	**Chi² = 1.87, df = 1 (P = 0.17), I² = 46.5%**	-0.37 (-0.59 to -0.15), 1 trial, n=204 I2= NA	-0.21 (-0.50 to 0.09) 6 trials, n=747 I2=81%	**Chi² = 0.77, df = 1 (P = 0.38), I² = 0%**	-0.36 (-0.57 to -0.14) 2 trials, n=354, I2=0%	-0.21 (-0.36 to -0.06), 6 trials, n=697, I2=81%	**Chi² = 1.18 df = 1 (P = 0.28), I² = 14.9%**
**HOSP LOS MD (95% CI)**	-056 (-1.08 to -0.04), 2 trials, n=279. I2=0%	-0.46 (-0.77 to -0.15), 4 trials, n=482, I2=0%	**Chi² = 0.1, df = 1 (P = 0.75), I² = 0%**	-0.43 (-1.55 to 0.69) 3 trials, n=328. I2=0%	-0.49 (-0.77 to -0.22) 3 trials, n=433 I2=0%	**Chi² = 0.01, df = 1 (P = 0.91), I² = 0%**	-0.50 (-0.82 to -0.18) 1 trial, n=204 I2= NA	-0.46 (0.05 to 0.93) 5 trials, n=557 I2=0%	**Chi² = 0.02, df = 1 (P = 0.89), I² = 0%**	-0.48 (-0.80 to -0.17), 2 trials n=354, I2=0%	-0.50 (-1.00 to -0.00) 4 trials, n=407 I2=0%	**Chi² = 0.00, df = 1 (P = 0.95), I² = 0%**

RR: Risk ratio, MD: Mean Difference, 95%CI: 95% confidence intervals, MI: Myocardial Infarction, AKI: Acute Kidney Injury, ICU: Intensive care unit, Hosp Hospital, LOS: Length of stay, CABG: coronary artery bypass grafting.

Figure. S1 PRISMA Flowchart of included studies

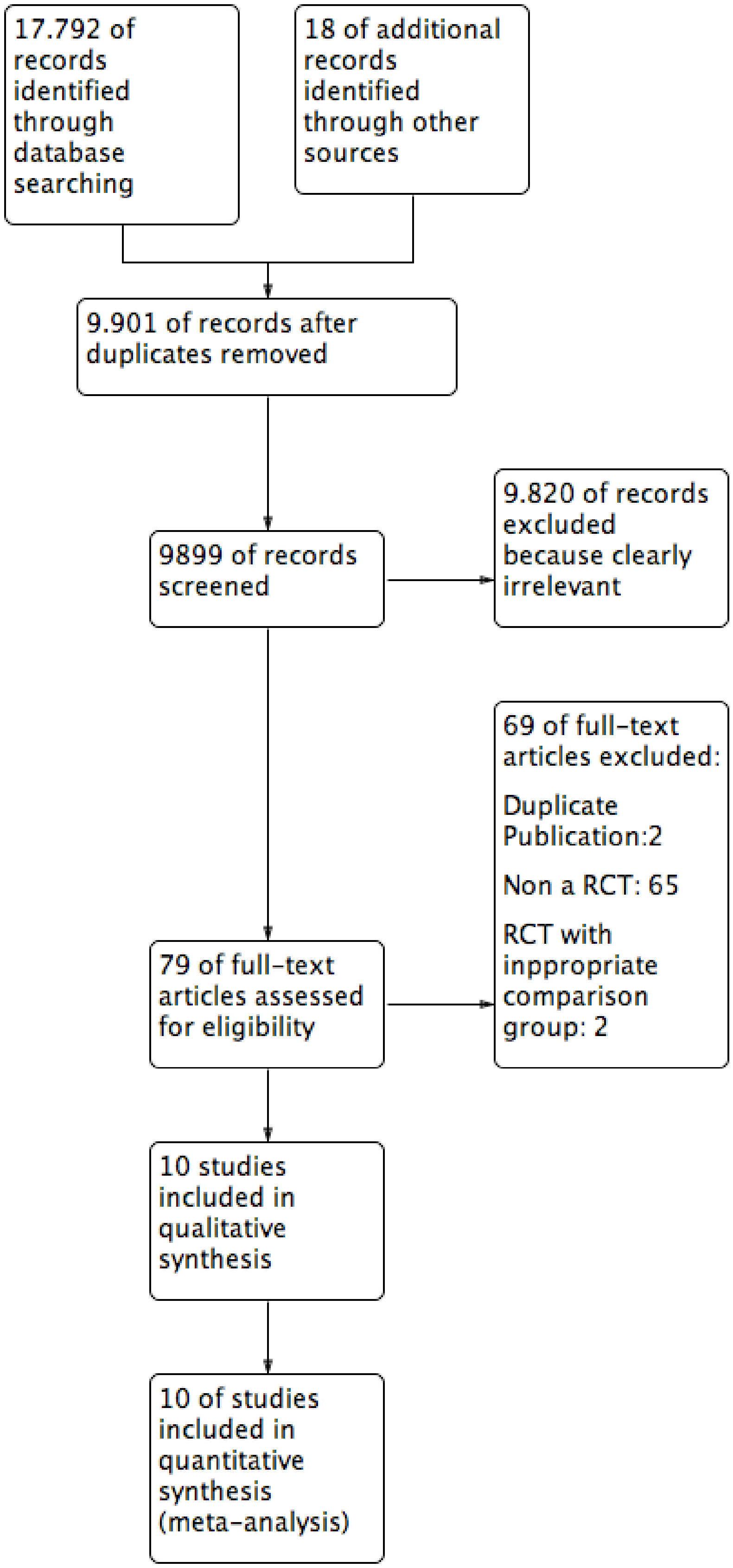

Figure S2: Effects of using NIRS on Bleeding, Transfusion and Time to discharge from ICU or Hospital.

RBC Transfusion

Study or Subgroup	NIRS Events	NIRS Total	Control Events	Control Total	Weight	Risk Ratio M-H, Random, 95% CI
Colak 2014	77	94	73	96	41.0%	1.08 [0.93, 1.25]
Murkin, 2007	8	100	10	100	4.0%	0.80 [0.33, 1.94]
Rogers 2017	37	98	44	106	19.3%	0.91 [0.65, 1.28]
Vretzakis 2013	51	75	63	75	35.7%	0.81 [0.67, 0.97]
**Total (95% CI)**		**367**		**377**	**100.0%**	**0.93 [0.77, 1.12]**
Total events	173		190			

Heterogeneity: Tau² = 0.02; Chi² = 6.07, df = 3 (P = 0.11); I² = 51%
Test for overall effect: Z = 0.77 (P = 0.44)

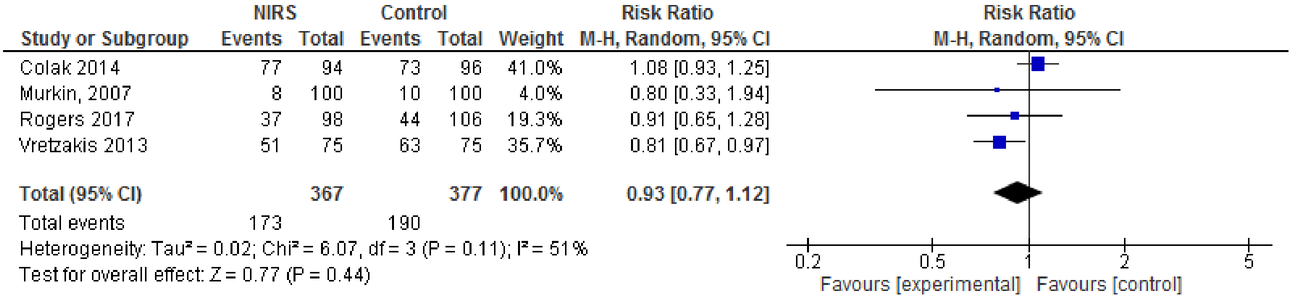

Reoperation for bleeding

Study or Subgroup	NIRS Events	NIRS Total	Control Events	Control Total	Weight	Risk Ratio M-H, Random, 95% CI
Colak 2014	0	94	1	96	9.7%	0.34 [0.01, 8.25]
Murkin, 2007	1	100	1	100	12.9%	1.00 [0.06, 15.77]
Rogers 2017	6	98	4	106	64.4%	1.62 [0.47, 5.58]
Vretzakis 2013	1	75	1	75	13.0%	1.00 [0.06, 15.69]
**Total (95% CI)**		**367**		**377**	**100.0%**	**1.23 [0.46, 3.32]**
Total events	8		7			

Heterogeneity: Tau² = 0.00; Chi² = 0.86, df = 3 (P = 0.83); I² = 0%
Test for overall effect: Z = 0.41 (P = 0.68)

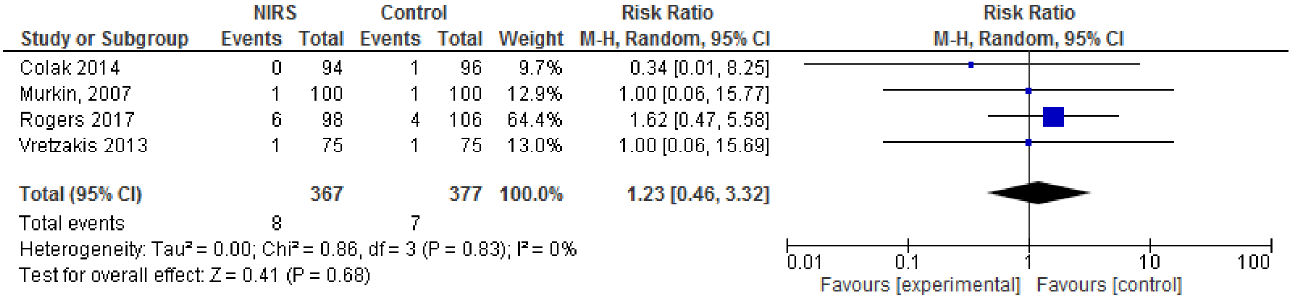

Intensive care Unit Length of Stay

Study or Subgroup	NIRS Mean	NIRS SD	NIRS Total	Control Mean	Control SD	Control Total	Weight	Mean Difference IV, Random, 95% CI
Colak 2014	2.7	6.2	94	1.9	0.9	96	5.6%	0.80 [-0.47, 2.07]
Deschamps 2013	2.99	2.26	23	0.39	2.05	25	5.9%	2.60 [1.38, 3.82]
Deschamps 2016	2.65	2.42	34	3.35	3.61	46	5.3%	-0.70 [-2.02, 0.62]
Kara, 2015	1.74	0.81	43	2.12	1.05	36	17.6%	-0.38 [-0.80, 0.04]
Mohandas 2013	1.49	0.38	50	1.7	0.49	50	22.6%	-0.21 [-0.38, -0.04]
Murkin, 2007	1.25	0.84	100	1.87	2.67	100	14.9%	-0.62 [-1.17, -0.07]
Rogers 2017	3.3	0.81	98	3.67	0.79	106	21.8%	-0.37 [-0.59, -0.15]
Vretzakis 2013	2.7	3.8	75	2.7	3.6	75	6.2%	0.00 [-1.18, 1.18]
**Total (95% CI)**			**517**			**534**	**100.0%**	**-0.13 [-0.47, 0.22]**

Heterogeneity: Tau² = 0.13; Chi² = 27.52, df = 7 (P = 0.0003); I² = 75%
Test for overall effect: Z = 0.71 (P = 0.48)

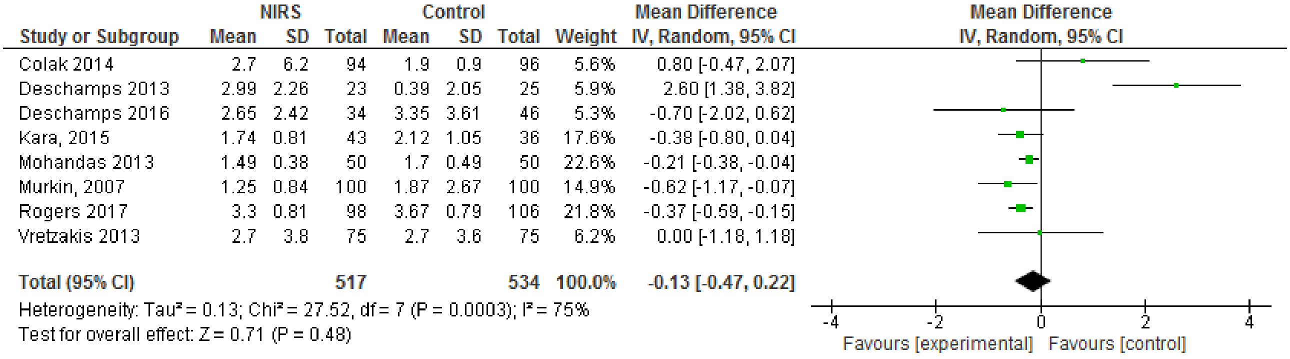

Hospital Length of Stay

Study or Subgroup	NIRS Mean	NIRS SD	NIRS Total	Control Mean	Control SD	Control Total	Weight	Mean Difference IV, Random, 95% CI
Deschamps 2013	7.6	5.4	23	7.9	3.2	25	3.2%	-0.30 [-2.84, 2.24]
Deschamps 2016	11	7.2	34	9.9	5.8	46	2.4%	1.10 [-1.84, 4.04]
Kara, 2015	7.15	1.39	43	7.67	1.14	36	66.3%	-0.52 [-1.08, 0.04]
Murkin, 2007	6.1	4.4	100	6.9	5.5	100	10.8%	-0.80 [-2.18, 0.58]
Rogers 2017	8.5	4.2	98	8.9	4.6	106	14.1%	-0.40 [-1.61, 0.81]
Vretzakis 2013	10.9	3.6	75	10.2	10.7	75	3.2%	0.70 [-1.85, 3.25]
**Total (95% CI)**			**373**			**388**	**100.0%**	**-0.45 [-0.90, 0.01]**

Heterogeneity: Tau² = 0.00; Chi² = 2.17, df = 5 (P = 0.83); I² = 0%
Test for overall effect: Z = 1.94 (P = 0.05)

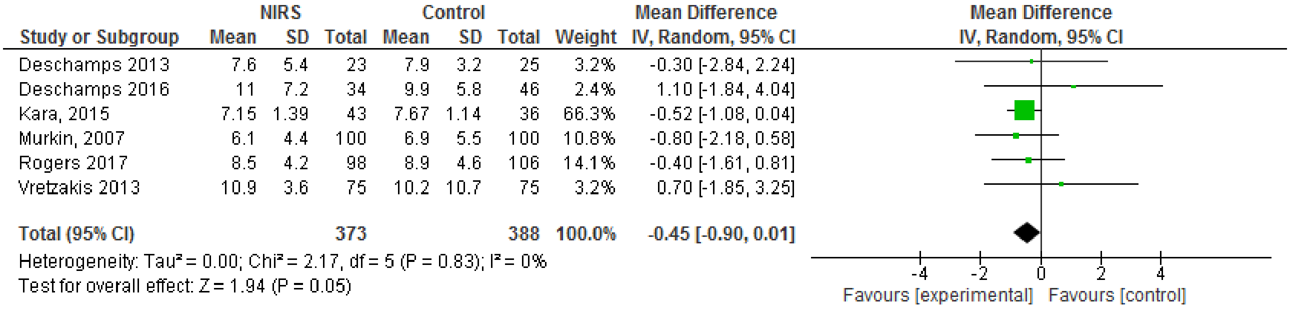

PROTOCOL INFORMATION

Protocol title: Efficacy of Near-Infrared Spectroscopy on the outcome of patients undergoing cardiac surgery: a systematic review.

Authors

**Prof Gavin J. Murphy**<br>British Heart Foundation<br>Professor of Cardiac Surgery<br>Department of Cardiovascular Sciences<br>University of Leicester<br>Glenfield Hospital Groby Road Leicester,<br>LE3 9QP Tel: 0116 258 3054<br>Email: gjm19@le.ac.uk	**Dr Giovanni Mariscalco**<br>Senior Lecturer<br>Department of Cardiovascular Sciences<br>University of Leicester<br>Glenfield Hospital Groby Road Leicester,<br>LE3 9QP Tel: 0116 258 3019<br>Email: gm247@leicester.ac.uk
**Dr Giuseppe Filiberto Serraino**<br>Clinical Research Fellow<br>Department of Cardiovascular Sciences University of Leicester Glenfield Hospital Groby Road Leicester,<br>LE3 9QP Email: gfs3@le.ac.uk	

Contact person: Professor Gavin J Murphy, British Heart Foundation Professor of Cardiac Surgery University of Leicester, Glenfield Hospital Groby Road Leicester, LE3 9QP. Tel: 0116 258 3054, Email: gjm19@le.ac.uk

Funding sources/sponsor

Leicester Cardiovascular Biomedical Research Unit

Dates

Anticipated or actual start date ---

Anticipated completion date ----

Type of review

Epidemiologic; Intervention

Language

English

Country

United Kingdom

Keywords

Systematic review; Cardiac Surgery; Near Infra-Red Spectroscopy; Near Infrared Oximetry; Cognitive dysfunction.

ABSTRACT

Background: Organ injury attributable to cardiopulmonarybypass is a major cause of morbidity, mortality and resource use in cardiac surgery patients. Regional tissue hypoxia during CPB has been implicated in perioperative organ injury. It has been suggested that goal directed patient specific optimisation of regional (brain) tissue hypoxia measured using Near Infra-Red Specroscopy (NIRS) may reduce the frequency of brain injury. Other investigators have suggested that cerebral NIRS based algorithsm may have utility for the protection of other organs, or a part of a restricitive red cell transfusion protocol.

Objectives: Our primary objective is to establish whether the use of perioperative goal directed interventions that aim to optimise cerebral Near Infrared Spectroscopy readings result in reductions in measures of cerebral injury (neurocognitive function, serum biomarkers), injury to other organs including the heart and brain, transfusion rates, mortality and resource use.

Search methods: We will conduct the search in October 2015. Potentially eligible trials will be identified by searching the Cochrane Central Register of Controlled Trials, MEDLINE , and EMBASE, using a combination of subject headings and text words to identify relevant trials. The Medline search strategy below will be adapted as appropriate for other databases.

((Cardiopulmonary Bypass) OR (Cardiac Surgery) OR (Coronary Artery Bypass) OR (Extra Corporeal Circulation)OR(Perioperative Morbidity)) AND ((Near Infra-Red Spectroscopy) OR (Oximetry) OR (Brain/Metabolism)OR(Cerebral Desaturation)OR(Cerebral Perfusion)OR(Cerebral Ischemia)OR(Cerebral Oximetry)OR(Cerebral Saturation)OR(Near Infrared Oximetry)(Cognitive)).

Selection Criteria: Two review authors will independently select references for further assessment by going through all titles and abstracts. Further selection will be based on review of full-text articles for selected references.

Data Collection and Analysis: Two review authors will independently extract study data. We will calculated the risk ratio (RR) with 95% confidence interval (CI) for binary outcomes and the mean difference (MD) or the standardised mean difference (SMD) with 95% CI for continuous outcomes. We will perform meta-analysis when possible, when I^2 is less than or equal to 80% using a fixed-effect or random-effects model, using Review Manager software. The range of point estimates for individual studies will be presented when

$I^2 > 80\%$. Heterogenity will be explored using subgroup analyses. Sensitivity analyses will explore the robustness of our primary analysis to exclusion of studies at high risk of bias.

Summary: We will use GRADEpro software to prepare the 'Summary of findings'table. We will judge the overall quality of the evidence foreach outcome as 'high,' 'moderate,' 'low' or 'very low' accordingto the GRADE (Grades of Recommendation, Assessment, Developmentand Evaluation) approach.

BACKGROUND

Organ injury is a common and severe complication of cardiac surgery with cardiopulmonary bypass (CPB), where it contributes to mortality, morbidity and the increased use of hospital resources [1]. In a recent trial where data on organ dysfunction was prospectively collected clinically significant kidney, lung and myocardial injury occurred in 34%, 16% an 11% of all patients and contributed to 41%, 36% and 24% of all deaths respectively [2]. The pathophysiology of CPB associated morbidity is multifactorial but is thought to involve regional hypoperfusion and tissue hypoxia [3, 4], often within vascular beds which are already abnormal due to advanced age or comorbidities such as diabetes [5, 6]. Adequate tissue oxygen delivery during CPB is achieved by optimisation of several parameters including CPB pump flows, perfusion pressure, haematocrit and the oxygen saturation of arterial blood. In contemporary clinical practice in adult cardiac surgery, the adequacy of perfusion is determined by the use of global measures of oxygen utilisation such as the mixed venous oxygen saturation (SvO2) or evidence of tissue hypoxia as implied by elevated serum lactate, a marker of cell anaerobic metabolism, or other indicators of metabolic acidosis [7]. However, in some patients, particularly those with pre-existing end organ dysfunction, global measures may not detect regional hypoxia and it has been suggested that measures of regional or tissue specific measures of oxygenation may have greater clinical utility [8-11].

The Intervention

Direct measures of regional tissue oxygen levels such as gastric tonometry, laser Doppler flowmetry of the intestinal mucosa, or cerebral venous oxygen saturation using jugular bulb catheters, can be used to measure and optimise tissue oxygenation but these modalities are invasive, which limits their use. More

recently, Near Infra-red Spectroscopy (NIRS) has emerged as a technique to monitor regional tissue oxygenation non-invasively and accurately with the added benefit that it can accurately measure tissue oxygenation in one of the most important end organs, the brain. NIRS sensors, when applied to the forehead, can determine the relative saturation / desaturation of blood within the cerebral arterioles and venules of the forebrain. Sas the total volume of blood is domintaed by th enemous compartment this measure is considered to accurately reflect cerebral venous oxygen saturation [12, 13] and is now approved as a non-invasive measure of regional cerebral oxygenation by the Medicines and Healthcare Products Regulatory Agency (MHRA). Early clinical studies suggested that the use of this technoliogy to optimise cerebral oxygenation during CPB have shown that optimisation of cerebral oxygenation using NIRS can be associated not only with reduced neurological morbidity but also with a reduction in renal complications and other major adverse clinical events [14-16]. However in the absence of high quality evidence there is uncertainty as to the efficacy or cost effectiveness of this device and there is wide variation in its use [ ].

Indications for red blood cell transfusion during cardiac surgery

Cerebral oximetry may also be used during CPB to develop goal-directed, patient-specific indicators of the need for red blood cell (RBC) transfusion if used as part of a wider algorithm designed to optimise cerebral oxygenation. The primary goal of RBC transfusion is to optimise tissue oxygenation. Currently, most RBC transfusions are given solely as a response to a haematocrit that has fallen below an arbitrary threshold and not as a response to incipient tissue hypoxia [18, 19]. The haematocrit is a poor indicator of regional tissue hypoxia and, therefore, is inadequate as an indicator of the need for transfusion.The haematocrit below which oxygen delivery to tissues is reduced such that anaerobic metabolism occurs is known as the critical Haematocrit (Hcrit). In healthy human adults very low haematocrits of less than 15 can cause organ hypoxia [9, 11] and transfusion in such cases is thought to be beneficial [20, 21]. The Hcrit for patients during cardiac surgery, however, is unclear. It is thought to be higher than 15, as most patients are elderly and have comorbid conditions, but it is also likely that the Hcrit varies considerably both between cardiac patients as well as for individual patients over the course of the perioperative period [22]. Hcrit is increased by conditions which impair autoregulation such as diabetes or increased age [5, 6] and during CPB, Hcrit is

also affected by multiple factors that affect the balance of oxygen supply and oxygen demand such as hypothermia, re-warming, pump flow or perfusion pressure [23]. Uncertainty about when an individual may benefit from a transfusion is reflected in the wide range in reported haematocrit transfusion thresholds used during CPB (from 17-25) [24-26] as well as the wide variation in transfusion rates in cardiac surgical patients across units in the United Kingdom (UK) (35-75%) [27]. Because the Hcrit for any particular patient at any given time is unknown, the ability to directly measure tissue oxygenation and therefore incipient tissue hypoxia may have distinct advantages over the use of generic and pre-specified haematocrit transfusion thresholds.

How the intervention might work

Existing protocols to optimise tissue oxygenation during CPB deploy manipulations of CPB (according to levels of blood markers such as lactate or pH) and the transfusion of RBC (according to the haematocrit). These methods are often treated independently of each other, and in the case of trandsfusion commonly guided by protocolised thresholds, despite the fact that both have the underlying aim of improving tissue oxygenation [7].

In contrast the patient-specific algorithm differs from the standard care in two key ways. First it is 'goal-directed', in that the algorithm is specifically targeted to maintain cerebral oxygen delivery during CPB (monitored by NIRS), a measure of regional rather than global tissue hypoxia. Second it allows the manipulation of multiple variables to optimise regional oxygenation for individual patients. This represents a form of personalised medicine. The most widely cited patient-specific algorithm developed by Murkin and colleagues [28] aims to optimise the cerebral oxygen supply / demand balance during CPB by: (a) increasing oxygen supply using hyperoxygenation, increased pump flow, perfusion pressure or hypercapnic cerebral vasodilation, (b) Increasing oxygen offloading by the use of nitrates, or (c) reducing oxygen demand by deepening anaesthesia. Cerebral oxygen saturations approaching a low threshold in the presence of anaemia (a haematocrit of between 18 and 23) and despite optimisation of other parameters suggest that the cerebral Hcrit is about to be reached and transfusion is indicated. Therefore, this algorithm is patient and time specific and goal-directed to optimise a validated objective measure of tissue oxygenation. This

should potentially reduce healthcare costs associated with complications attributable to tissue hypoxia during CPB or to unnecessary allogeneic RBC transfusions.

Why is it important to do this review

Numerous studies have reported the incidence of postoperative neurocognitive decline around 50% [29, 30]. Central venous oxygen desauration is common following cardiac surgery and this has been implicated in cognitive decline [3, 4]. Multiple studies have evaluated the efficacy of NIRS based algorithms that allow optimisation of cerebral saturation. Although some of these studies have been reported as positive [8] overall these results of these studies have been have been inconclusive, and no recent systematic review has attempted to summarise the available evidence. The use of this technology is variable and there is evidence of equipoise as to its effectiveness. To resolve this uncertainty we propose to undertake a systematic review and meta-analysis of the available evidence from RCTs.

OBJECTIVES

(1) To establish whether the use of perioperative goal directed interventions that aim to optimise cerebral Near Infrared Spectroscopy readings result in reductions in measures of cerebral injury (neuroscogitive function, serum biomarkers), morbidity, mortality and resource use.

(2) Does the use of perioperative goal directed interventions that aim to optimise cerebral Near Infrared Spectroscopy readings result in reductions in red cell transfusion, non red cell transfusion, or bleeding.

METHODS

Types of Studies

Randomized controlled trials irrespective of blinding, language, publication status, date of publication and sample size.

Types of Participants

Patients undergoing cardiac surgery for acquired or congenital disease, or aortovascular disease with or without cardiopulmonary bypass. No age restriction will be applied. There are no exclusion criteria.

Types of Interventions

Intervention: Goal directed NIRS algorithm.

Comparator /control: An untread group, or alternative (non NIRS based) goal directed therapy.

Types of Outcome Measure

Primary outcomes:

Mortality: 30 day or hospital all-cause mortality.

Secondary outcomes:

1. Acute brain injury; stroke, TIA as defined by study authors.
2. Low cardiac output as defined by study authors.
3. Myocardial Infarction as defined by study authors.
4. Acute kidney Injury Stage 3 [32] or requiring haemofiltration as defined by study authors.
5. Neurocognitive function; group means as described by neurocognitive tests. Tests recommended by a consensus statement to test all key domains of cognitive function [33] are marked with *. Studies will be categorised as Yes/ No as to whether they have assessed the key domains described in the Consensus Statement. Key domians [ ] that may be assessed are as follows
   - Attention: Sustained and divided attention: Consensus statements reccomend the Trail-Making Test parts A* and B* [35, 36].
   - Verbal memory: Consensus statements reccomend the RAVLT and Rey Auditory Verbal Learning Test* [36]
   - Visuo-spatial: as the Block Design from the Wechsler Adult Intelligence Scale [37]
   - Psychomotor speed: Consensus statements reccomend tests such the Digit Symbol Test from the Wechsler Adult Intelligence Scale [37]
   - Executive function/Verbal fluency: Consensus statements reccomend tests such the Controlled Oral Word Association Test [38]
   - Motor coordination: Consensus statements reccomend tests such the Grooved Pegboard Test*, dominant and non-dominant hand [33].
6. Neurocognitive dysfunction, as a dichotomous outcome reported by the study authors; A consensus definition is a change in a single test of >1 SD. This may be defined as change in a group mean (adjusted for baseline) or for individual patients [39]. Studies will be categorised as defining cognitive dysfunction using a Consensus versus a non-Consensus definitions.
7. Risk of receiving blood transfusion as defined by study authors.
8. Reoperation for bleeding as defined by study authors.
9. Resoiurce Use: ICU and hospital LOS as defined by study authors.
10. S100B levels as reported by study authors.

Search methods for identification of studies

Potentially eligible trials will be identified by searching the Cochrane Central Register of Controlled Trials, MEDLINE , and EMBASE, using a combination of subject headings and text words to identify relevant trials. The Medline search strategy below will be adapted as appropriate for other databases.

((Cardiopulmonary Bypass) OR (Cardiac Surgery) OR (Coronary Artery Bypass) OR (Extra Corporeal Circulation)OR(Perioperative Morbidity)) AND ((Near Infra-Red Spectroscopy) OR (Oximetry) OR (Brain/Metabolism)OR(Cerebral Desaturation)OR(Cerebral Perfusion)OR(Cerebral Ischemia)OR(Cerebral Oximetry)OR(Cerebral Saturation)OR(Near Infrared Oximetry)(Cognitive)).

To identify ongoing or unpublished trials we will also search the Clinicaltrials.gov using the following search terms:

Search terms: Randomized

Study Type: Interventional Studies

Conditions: Cardiac surgery OR Cardiopulmonary bypass

Interventions: Near Infra-Red Spectroscopy OR Near Infrared Oximetry OR Cerebral Desauturation

We will also examined the reference lists of eligible trials and reviews. Searches will not be restricted by language or publication status.

Data Collection and Analysis

The review will be performed in accordance with instructions given in the Cochrane Handbook for Systematic Reviews of Interventions (Higgins 2011).

Selection of Studies

Two reviewers GJM, GFS will identify trials for inclusion independently of each other. Exluded studies and the reason for exclusion will be recorded.

Data extraction (selection and coding)

Two authors will independently screen the search output to identify records of potentially eligible trials examining the outcomes, the full texts of which will be retrieved and assessed for inclusion.

A standardised form will be used to extract data from the included studies for assessment of study quality and evidence synthesis. Extracted information will include:

- Year and language of publication

- Country of Particiapnt recruitment
- Year of conduct of the trial
- Study setting; university teaching hospital, non universityteaching hospital
- Study population; inclusion and exlusion criteria
- Sample size
- Participant demographics
- Baseline characteristics
- Type of surgery
- Details of NIRS algorithm (Murkin, non Murkin [41])
- Deatails of Comparator; non NIRS goal directed therapy, standard care (protocolised care)
- Outcomes and times of measurement
- Information for assessment of the risk of bias.

Two review authors will extract data independently, discrepancies will be identified and resolved through discussion (with a third author where necessary). Missing data will be requested from study authors. If there is doubt as to whether trials share participiants completely or partially (with common authors and centres) we will contact the study authors to ascertain whether the studt report has been duplicated.

Risk of Bias

The following bias risk domains will be assessed as Low, Uncertain, or High based on the instructions given in the in the Cochrane Handbook for Systematic Reviews of Interventions [42];

- Sequence Generation
- Allocation Concealment
- Blinding of participant, personnel
- Blinding of outcome Assessors
- Incomplete outcome data
- Selective outcome reporting
- Source of funding bias

Trials will be classified as having a low risk of bias if they are graded as being at low risk of bias in all of these domains.

Two review authors will independently assess the risk of bias in all of the studies. Discrepancies will be resolved by discussion with a third author.

Assessment of Reporting Bias

Publication bias will be assessed by the visual assessment of funnel plots and Eggers test [44].

Measures of treatment Effect

For dichotomous variables, we will calculate the risk ratio (RR) with 95% confidence interval (CI). For continuous variables, we will calculate the mean difference (MD) with 95% CI for outcomes such as hospital

stay, and standardised mean difference (SMD) with 95% CI for quality of life (when different scales were used).

Dealing with missing data

We will perform an intention-to-treat analysis where possible. For dichotomous data presented only as percentages we will estimate frequencies using reported sample sizes for this outcome. For continuous outcomes if the mean and the standard deviation were not available from the trial report, we will seek this information from the trial authors. If this information is still not available, we will calculate the mean and standard deviation from median (interquartile ranges) using the software available in Review Manager Version 5.

Assessment of Heterogeneity

We anticipate that major sources of clinical heterogeneity will be associated with different patient groups (adults, children, congenital versus acquired disease), the use of different goal directed NIRS algorithms, the use of co-interventions such as restrictive transfusion thresholds, and differences in the methodology used to assess neurocognitive dysfunction. We will explore heterogeneity within each meta-analysis using a Chi^2 test with significance set at a P value of 0.10, and we expressed the percentage of heterogeneity due to variation rather than to chance as I^2 [45].We defined heterogeneity as follows:

I^2 0-40%: no or mild heterogeneity

I^2 40-80%: moderate heterogeneity

$I^2 > 80\%$: sever heterogeneity

In the presence of severe heterogeneity meta-analysis will not be performed.

Data Synthesis

Meta-analyses will be performed using the software package Review Manager version 5.2 and in accordance with the recommendations of the *Cochrane Handbook for Systematic Reviews of Intervention* [46].

For the primary analysis we will compare the results of a random-effects model versus a fixed effects model to assess the effects of small studies. For continuous outcomes, we will pool mean differences or

standardised mean differences by using the inverse variance method. We will use the generic inverse variance method to pool hazard ratios.

Subgroup Analyses

Subgroup analyses will be performed in trials in which the Murkin algorithm [41] was used to guide goal directed therapy versus those that did not, and by participant group; CABG versus non CABG, adults versus children, assessment of neurocognitive function that incorporates test described in a previous consensus statement, and studies that combined the NIRS algorithm with a restrictive red cell transfusion trigger. Test for sub-group differences with Review Manager will be used with a P value of <0.05 considered statistically significant.

Sensitivity Analyses

Sensitivity analysis will exclude trials with unclear or high risk of bias for random sequence generation; unclear or high risk of bias due to lack of blinding of participants, healthcare providers or outcome assessors, and unclear or high risk of bias due to incomplete outcome data, and unclear of high risk of bias for source of funder.

Summary of findings

We will presentthe main results of the review in a 'Summary of findings' table. We will include the follwing outcomes.

- Risk of mortality
- Risk of stroke, myocardial infarction, or severe acute kidney injury
- Risk of red cell transfusion
- Neurocognitive Impairment
- Resource Use; ICU and hospital length of stay.

We will use GRADEpro software to prepare the 'Summary of findings'table. We will judge the overall quality of the evidence foreach outcome as 'high,' 'moderate,' 'low' or 'very low' accordingto the GRADE (Grades of Recommendation, Assessment, Developmentand Evaluation) approach [46].We will consider the following.

- Impact of risk of bias of individual trials.
- Precision of pooled estimate.Inconsistency or heterogeneity (clinical, methodological and statistical).

- Indirectness of evidence.
- Impact of selective reporting and publication bias on effect estimate.

- **COMPETING INTERESTS**

The authors declare that they have no competing interests.

AUTHORS' CONTRIBUTIONS

___ had full access to all of the data in the study and take responsibility for the integrity of the data and the accuracy of the data analysis.

Study concept and design: ___.

Acquisition of data: ___.

Analysis and interpretation of data: _____.

Drafting of the manuscript: _______.

Statistical analysis: ______

Study supervision: GJM.

REFERENCES

1. Murphy, G J and G D Angelini. Side effects of cardiopulmonary bypass: what is the reality? J Card Surg, 2004. 19(6): p. 481-488.
2. Murphy GJ,
3. Sapire, K J, S P Gopinath, G Farhat, D R Thakar, A Gabrielli, J W Jones, C S Robertson, and B Chance. Cerebral oxygenation during warming after cardiopulmonary bypass. Crit Care Med, 1997. 25(10): p. 1655-1662.
4. Yao, F S, C C Tseng, C Y Ho, S K Levin, and P Illner. Cerebral oxygen desaturation is associated with early postoperative neuropsychological dysfunction in patients undergoing cardiac surgery. J Cardiothorac Vasc Anesth, 2004. 18(5): p. 552-558.
5. Croughwell, N, M Lyth, T J Quill, M Newman, W J Greeley, L R Smith, and J G Reves. Diabetic patients have abnormal cerebral autoregulation during cardiopulmonary bypass. Circulation, 1990. 82(5 Suppl): p. IV407-412.
6. Miyoshi, S, T Morita, Y Kadoi, and F Goto. Analysis of the factors related to a decrease in jugular venous oxygen saturation in patients with diabetes mellitus during normothermic cardiopulmonary bypass. Surg Today, 2005. 35(7): p. 530-534.
7. Monitoring during CPB
8. Haisjackl, M, G Luz, H Sparr, R Germann, N Salak, B Friesenecker, E Deusch, S Meusburger, and W Hasibeder. The effects of progressive anemia on jejunal mucosal and serosal tissue oxygenation in pigs. Anesth Analg, 1997. 84(3): p. 538-544.
9. Leung, J M, R B Weiskopf, J Feiner, H W Hopf, S Kelley, M Viele, J Lieberman, J Watson, M Noorani, D Pastor, H Yeap, R Ho, and P Toy. Electrocardiographic ST-segment changes during acute, severe isovolemic hemodilution in humans. Anesthesiology, 2000. 93(4): p. 1004-1010.
10. Tsai, A G, P Cabrales, and M Intaglietta. Microvascular perfusion upon exchange transfusion with stored red blood cells in normovolemic anemic conditions. Transfusion, 2004. 44(11): p. 1626-1634.
11. Weiskopf, R B, J H Kramer, M Viele, M Neumann, J R Feiner, J J Watson, H W Hopf, and P Toy. Acute severe isovolemic anemia impairs cognitive function and memory in humans. Anesthesiology, 2000. 92(6): p. 1646-1652.
12. Kim, M B, D S Ward, C R Cartwright, J Kolano, S Chlebowski, and L C Henson. Estimation of jugular venous O2 saturation from cerebral oximetry or arterial O2 saturation during isocapnic hypoxia. J Clin Monit Comput, 2000. 16(3): p. 191-199.
13. Pollard, V, D S Prough, A E DeMelo, D J Deyo, T Uchida, and H F Stoddart. Validation in volunteers of a near-infrared spectroscope for monitoring brain oxygenation in vivo. Anesth Analg, 1996. 82(2): p. 269-277.
14. Alexander J.C.Jr, K M A, & Dance G.R. Reduced postoperative length of stay may result from using cerebral oximetry monitoring to guide treatment. The Annals of Thoracic Surgery, 2002. 73(1): p. S373.
15. Goldman, S, F Sutter, F Ferdinand, and C Trace. Optimizing intraoperative cerebral oxygen delivery using noninvasive cerebral oximetry decreases the incidence of stroke for cardiac surgical patients. Heart Surg Forum, 2004. 7(5): p. E376-381.
16. Murkin, J M, S J Adams, R J Novick, M Quantz, D Bainbridge, I Iglesias, A Cleland, B Schaefer, B Irwin, and S Fox. Monitoring brain oxygen saturation during coronary bypass surgery: a randomized, prospective study. Anesth Analg, 2007. 104(1): p. 51-58.
17. Variability Paper
18. Vincent, J L, J F Baron, K Reinhart, L Gattinoni, L Thijs, A Webb, A Meier-Hellmann, G Nollet, D Peres-Bota, and A B C Investigators. Anemia and blood transfusion in critically ill patients. JAMA, 2002. 288(12): p. 1499-1507.
19. Walsh, T S, M Garrioch, C Maciver, R J Lee, F MacKirdy, D B McClelland, J Kinsella, C Wallis, and G Audit of Transfusion in Intensive Care in Scotland Study. Red cell requirements for intensive care units adhering to evidence-based transfusion guidelines. Transfusion, 2004. 44(10): p. 1405-1411.

20. Casutt, M, B Seifert, T Pasch, E R Schmid, M I Turina, and D R Spahn. Factors influencing the individual effects of blood transfusions on oxygen delivery and oxygen consumption. Crit Care Med, 1999. 27(10): p. 2194-2200.
21. Weiskopf, R B, J Feiner, H Hopf, J Lieberman, H E Finlay, C Quah, J H Kramer, A Bostrom, and P Toy. Fresh blood and aged stored blood are equally efficacious in immediately reversing anemia-induced brain oxygenation deficits in humans. Anesthesiology, 2006. 104(5): p. 911-920.
22. Utoh, J, S Moriyama, K Okamoto, R Kunitomo, M Hara, and N Kitamura. The effects of cardiopulmonary bypass on postoperative oxygen metabolism. Surg Today, 1999. 29(1): p. 28-33.
23. Shann KG, Likosky DS, Murkin JM, Baker RA, Baribeau YR, DeFoe GR, Dickinson TA, Gardner TJ, Grocott HP, O'Connor GT, Rosinski DJ, Sellke FW, Willcox TW. An evidence-based review of the practice of cardiopulmonary bypass in adults: a focus on neurologic injury, glycemic control, hemodilution, and the inflammatory response. J Thorac Cardiovasc Surg. 2006 Aug;132(2):283-90.
24. DeFoe, G R, C S Ross, E M Olmstead, S D Surgenor, M P Fillinger, R C Groom, R J Forest, J W Pieroni, C S Warren, M E Bogosian, C F Krumholz, C Clark, R A Clough, P W Weldner, S J Lahey, B J Leavitt, C A Marrin, D C Charlesworth, P Marshall, and G T O'Connor. Lowest hematocrit on bypass and adverse outcomes associated with coronary artery bypass grafting. Northern New England Cardiovascular Disease Study Group. Ann Thorac Surg, 2001. 71(3): p. 769-776.
25. Fang, W C, R E Helm, K H Krieger, T K Rosengart, W J DuBois, C Sason, M L Lesser, O W Isom, and J P Gold. Impact of minimum hematocrit during cardiopulmonary bypass on mortality in patients undergoing coronary artery surgery. Circulation, 1997. 96(9 Suppl): p. II-194-199.
26. Von Heymann, C, M Sander, A Foer, A Heinemann, B Spiess, J Braun, M Kramer, J Grosse, P Dohmen, S Dushe, J Halle, W F Konertz, K D Wernecke, and C Spies. The impact of an hematocrit of 20% during normothermic cardiopulmonary bypass for elective low risk coronary artery bypass graft surgery on oxygen delivery and clinical outcome--a randomized controlled study. Crit Care, 2006. 10(2): p. R58.
27. Murpjhy GJ Audit
28. Denault A, Deschamps A, Murkin JM. A proposed algorithm for the intraoperative use of cerebral near-infrared spectroscopy. Semin Cardiothorac Vasc Anesth. 2007 Dec;11(4):274-81
29. Newman MF, Kirchner JL, Phillips-Bute, et al. Longitudinal assessment of neurocognitive function after coronary artery bypass surgery. New Engl J Med 2001;344:395–402.
30. Selnes OA, Goldsborough MA, Borowicz LM Jr, Enger C, Quaskey SA, McKhann GM. Determinants of cognitive change after coronary artery bypass graft surgery: a multi- factorial problem. Ann Thorac Surg 1999;67:1669 –76.
31. NIRS AKI
32. Mehta, R L, J A Kellum, S V Shah, B A Molitoris, C Ronco, D G Warnock, A Levin, and N Acute Kidney Injury. Acute Kidney Injury Network: report of an initiative to improve outcomes in acute kidney injury. Crit Care, 2007. 11(2): p. R31.
33. Murkin, J M, S P Newman, D A Stump, and J A Blumenthal. Statement of consensus on assessment of neurobehavioral outcomes after cardiac surgery. Ann Thorac Surg, 1995. 59(5): p. 1289-1295.
34. Mahanna, E P, J A Blumenthal, W D White, N D Croughwell, C P Clancy, L R Smith, and M F Newman. Defining neuropsychological dysfunction after coronary artery bypass grafting. Ann Thorac Surg, 1996. 61(5): p. 1342-1347.
35. SP Newman. Analysis and interpretation of neuropsychologic tests in cardiac surgery Ann Thorac Surg, 59 (1995), pp. 1351–1355
36. Spreen O., S, E. A compendium of neuropsychological tests. 1998, New York: Oxford University Press
37. Wechsler, D. Manual of the Wechsler Test of Adult Reading (WTAR-UK). 2001, New York Psychological Corporation (Iglesias)
38. Benton, A H, R. Multilingual Aphasia Examination. 1976, Iowa City: University of Iow
39. Murkin JM, Stump DA, Blumenthal JA, McKhann G. Defining dysfunction: group means versus incidence analysis-a statement of consensus. Ann Thorac Surg. 1997 Sep;64(3):904-5.
40. Higgins JPT, Deeks JJ. Chapter 7: Selecting studies and collecting data In: Higgins JPT,Green S (editors). Cochrane Handbook for Systematic Reviews of Interventions Version 5.1.0 [updated March 2011]. The Cochrane Collaboration, 2011. www.cochrane-handbook.org.

41. Denault A, Deschamps A, Murkin JM. A proposed algorithm for the intraoperative use of cerebral near-infrared spectroscopy. Semin Cardiothorac Vasc Anesth. 2007 Dec;11(4):274-81
42. Higgins JPT, Altman DG, Sterne JAC. Chapter 8: Assessing risk of bias in included studies. Cochrane Handbook for Systematic Reviews of Interventions Version 5.1.0 [updated March 2011]. The Cochrane Collaboration, 2011. www.cochrane-handbook.org.
43. Sterne JAC, Egger M, Moher D. Chapter 10: Addressing reporting biases In: Higgins JPT, Green S (editors). Cochrane Handbook for Systematic Reviews of Interventions Version 5.1.0 [updated March 2011]. The Cochrane Collaboration, 2011. www.cochranehandbook.org.
44. Higgins JPT, Thompson SG. Quantifying heterogeneity in a meta-analysis. Statistics in Medicine 2002;21(11):1539–58.
45. Higgins JPT, Green S (editors). Cochrane Handbook for Systematic Reviews of Interventions Version 5.1.0 [updated March 2011]. The Cochrane Colloboration, 2011. www.cochrane-handbook.org.
46. Schünemann HJ, Oxman AD, Vist GE, Higgins JPT, Deeks JJ, Glasziou P, et al.Chapter 12: Interpreting results and drawing conclusions. Higgins JPT, Green S(editors). Cochrane Handbook for Systematic Reviewsof Interventions Version 5.1.0 [updated March 2011].The Cochrane Collaboration, 2011. www.cochrane-handbook.org.
