# Supplementary material for: Effects of cerebral near-infrared spectroscopy on the outcome of patients undergoing cardiac surgery: a systematic review of randomised trials
Source: BMJ Open. 2017 Sep 7;7(9):e016613. doi: 10.1136/bmjopen-2017-016613 (PMC5595187; doi:10.1136/bmjopen-2017-016613)
Supplement: Author's manuscript [file bmjopen-2017-016613.draft_revisions.pdf]

BMJ Open is committed to open peer review. As part of this commitment we make the peer review history of every article we publish publicly available.

When an article is published we post the peer reviewers' comments and the authors' responses online. We also post the versions of the paper that were used during peer review. These are the versions that the peer review comments apply to.

The versions of the paper that follow are the versions that were submitted during the peer review process. They are not the versions of record or the final published versions. They should not be cited or distributed as the published version of this manuscript.

BMJ Open is an open access journal and the full, final, typeset and author-corrected version of record of the manuscript is available on our site with no access controls, subscription charges or pay-per-view fees (http://bmjopen.bmj.com).

If you have any questions on BMJ Open's open peer review process please email editorial.bmjopen@bmj.com

BMJ Open

Effects of cerebral Near-Infrared Spectroscopy on the outcome of patients undergoing cardiac surgery: a systematic review of randomised trials.

Journal:	*BMJ Open*
Manuscript ID	bmjopen-2017-016613
Article Type:	Research
Date Submitted by the Author:	28-Feb-2017
Complete List of Authors:	Serraino, Giuseppe; University of Leicester College of Medicine Biological Sciences and Psychology, Cardiovascular Sciences<br>Murphy, Gavin; University of Leicester College of Medicine Biological Sciences and Psychology, Cardiovascular Sciences
**Primary Subject Heading**:	Cardiovascular medicine
Secondary Subject Heading:	Surgery, Anaesthesia, Neurology
Keywords:	Cardiopulmonary bypass, cerebral oxygenation, near Infra-red spectroscopy

SCHOLARONE™
Manuscripts

For peer review only

Title: Effects of cerebral Near-Infrared Spectroscopy on the outcome of patients undergoing cardiac surgery: a systematic review of randomised trials.

Authors: Giuseppe F Serraino, Gavin J Murphy

Authors Affiliation: Department of Cardiovascular Sciences and NIHR Leicester Biomedical Research Unit in Cardiovascular Medicine, University of Leicester, Clinical Sciences Wing, Glenfield General Hospital, Leicester, LE3 9QP

Correspondence to: Professor Gavin Murphy, Department of Cardiovascular Sciences and NIHR Leicester Biomedical Research Unit in Cardiovascular Medicine, University of Leicester, Clinical Sciences Wing, Glenfield General Hospital, Leicester, LE3 9QP. Email: gjm19@le.ac.uk Tel: +44 116 2583021, Fax: +44 116 2583054.

Total word count: 4012

Keywords: Cardiopulmonary bypass, cerebral oxygenation, near Infra-red spectroscopy.

For peer review only

For peer review only - http://bmjopen.bmj.com/site/about/guidelines.xhtml

Abstract

Objectives: Goal directed optimisation of cerebral oxygenation using Near Infra-red Spectroscopy (NIRS) during cardiopulmonary bypass is widely used. We tested the hypotheses that the use of NIRS cerebral oximetry results in reductions in cerebral injury (neurocognitive function, serum biomarkers), injury to other organs including the heart and brain, transfusion rates, mortality and resource use.

Design: Systematic review and meta-analysis.

Setting: Tertiary cardiac surgery centres in North America, Europe and Asia.

Participants: A search of Cochrane Central Register of Controlled Trials, Clinical Trials.gov, MEDLINE, and EMBASE and the Cumulative Index to Nursing and Allied Health Literature (CINAHL) Plus from inception to November 2016 identified 10 randomised trials, enrolling a total of 1466 patients, all in adult cardiac surgery.

Interventions: NIRS based algorithms designed to optimise cerebral oxygenation versus standard care (non NIRS based) protocols in cardiac surgery patients during cardiopulmonary bypass.

Outcome Measures: Mortality, organ injury affecting the brain, heart and kidneys, red cell transfusion and resource use.

Results: Two of the 10 trials identified in the literature search were considered at low risk of bias. Random effects meta-analysis demonstrated similar mortality (Risk Ratio (RR) 0.76, 95% Confidence Intervals (CI) 0.30 to 1.96), major morbidity including stroke (RR 1. 08, 95% CI 0.40 to 2.91), red cell transfusion, and resource use, in NIRS treated patients and controls, with little or no heterogeneity. Grades of Recommendation, Assessment, Development and Evaluation (GRADE) assessment of the quality of the evidence was Low or Very Low for all of the outcomes assessed.

Conclusions: The results of this systematic review did not support the hypotheses that cerebral NIRS based algorithms have clinical benefits in cardiac surgery.

Registration: PROSPERO CRD42015027696

For peer review only

For peer review only - http://bmjopen.bmj.com/site/about/guidelines.xhtml

Strengths and limitations of the Study

- We performed a systematic review of randomised trials evaluating the clinical benefits of near infrared spectroscopy based cerebral oximetry monitoring during cardiopulmonary bypass.
- This is most comprehensive review of this technology to date.
- All of the existing trials had important methodological limitations, including importantly a lack of blinding of clinical personnel.
- GRADE assessment of the quality of the evidence was Low or Very Low for all of the outcomes assessed indicating a high likelihood that the findings of the review may be altered by subsequent trials.

For peer review only

For peer review only - http://bmjopen.bmj.com/site/about/guidelines.xhtml

Introduction

Brain injury is a common and severe complication of cardiac surgery with cardiopulmonary bypass (CPB), affecting up to 40% of patients, where it contributes to morbidity, mortality, and the increased use of hospital resources [1]. The pathophysiology of CPB associated brain injury is multifactorial [2] but is thought to involve regional hypoperfusion and tissue hypoxia,[3,4] often within vascular beds which are already abnormal due to advanced age or comorbidities such as diabetes.[5,6] Previous studies have suggested that cerebral oxygenation may be measured non-invasively using Near Infra-red Spectroscopy (NIRS) sensors applied to the forehead,[7] and more importantly that targeted interventions during bypass that increase NIRS measured cerebral oxygenation have clinical benefits including reductions in brain injury,[8] and reductions in injury to other organ systems as a result of improved overall perfusion.[9,10] It has also been suggested that the use of NIRS may allow the safe application of restrictive red cell transfusion thresholds where there is evidence of adequate cerebral oxygenation; a putative personalised transfusion indicator.[7,11] However, there is uncertainty as to the clinical benefits of NIRS[12] and this leads to variability in the use of these devices.[13] To address this uncertainty we performed a systematic review and meta-analysis of randomised trials that had evaluated the effects of personalised NIRS based algorithms on clinical outcomes including mortality, organ (brain, heart, kidney) injury, transfusion and resource use.

Methods

Systematic review

The review followed the methods described in Cochrane Handbook for Systematic Reviews of Interventions.[14] The review protocol was registered prospectively; PROSPERO CRD42015027696. A detailed description of the methods and the protocol are available in the online only digital supplement.

Search methods: The literature search was conducted in November 2016. Potentially eligible trials were identified by searching the Cochrane Central Register of Controlled Trials, MEDLINE, and EMBASE, using a

For peer review only - http://bmjopen.bmj.com/site/about/guidelines.xhtml

combination of subject headings and text words. The Medline search strategy below was adapted as appropriate for other databases: ((Cardiopulmonary Bypass) OR (Cardiac Surgery) OR (Coronary Artery Bypass) OR (Extra Corporeal Circulation) OR (Perioperative Morbidity)) AND ((Near Infra-Red Spectroscopy) OR (Oximetry) OR (Brain/Metabolism) OR (Cerebral Desaturation) OR (Cerebral Perfusion) OR (Cerebral Ischemia) OR (Cerebral Oximetry) OR (Cerebral Saturation) OR (Near Infrared Oximetry) OR (Cognitive)).

Selection Criteria: Two reviewers (GFS, GJM) independently selected references for further assessment by going through all titles and abstracts. Study selection was based on review of full-text articles for selected references.

Outcomes: The primary outcome was 30 day or hospital all-cause mortality. Secondary outcomes (as defined in the online supplement) included acute brain injury (stroke), myocardial infarction, severe acute kidney injury, neurocognitive dysfunction, red cell transfusion, resource use charcaterised by length of stay in the intensive car eunit and hospital, and serum S100B, a biomarker of brain injury.

Data Collection and Analysis: Two reviewers (FS, GJM) independently extracted study data. Risk of bias assessments followed the Cochrane Handbook for Systematic Reviews of Interventions [15]. Risk ratios (RR) were calculated for binary outcomes and the mean difference (MD) or the standardised mean difference (SMD) for continuous outcomes. We explored heterogeneity within each meta-analysis using a Chi^2 test with significance set at a P value of 0.10, and expressed the percentage of heterogeneity due to variation rather than to chance as I^2.[16] Quantitative meta-analysis was performed when the I^2 was less than or equal to 80%. A random-effects model was used for the primary analysis using Review Manager software [14]. These results were compared to the results of fixed effects models to assess for small study effects. The range of point estimates for individual studies was presented when $I^2 < 80\%$. Heterogeneity was explored using subgroup analyses. Funnel plots were used to assess for publication bias. Sensitivity analyses explored the robustness of our primary analysis to exclusion of studies at high risk of bias.

For peer review only - http://bmjopen.bmj.com/site/about/guidelines.xhtml

Summary: GRADEpro software was used to prepare the 'Summary of findings' table. The overall quality of the evidence for each outcome was judged according to the Grades of Recommendation, Assessment, Development and Evaluation (GRADE) approach [17].

Results

Description of Studies

Search Results: We identified a total of 17792 references through electronic searches of the Cochrane Central Register of Controlled Trials (CENTRAL) (n=1347), MEDLINE (n=9924), EMBASE (n=6159), and the Cumulative Index to Nursing and Allied Health Literature (CINAHL) Plus (n=362). From ClinicalTrials.gov, we identified 18 trials that potentially met the inclusion criteria. We have shown the flow of search results in Figure 1. We excluded 7909 duplicates, then 9820 clearly irrelevant references, by reading titles and abstracts. No additional references were identified by reference searching. Of 79 study reports retrieved in full text, we excluded 69 references for the reasons listed in Figure S1 in the online only digital supplement. In total, 11 publications describing 9 randomised controlled trials fulfilled the inclusion and exclusion criteria provided quantitative data for this review [10,18-25] (Table S1 in the online only digital supplement). We also included data from a recent trial undertaken by our own unit, the results of which are unpublished.[26]

Description of excluded studies: Four trials that met our inclusion criteria were excluded after review of the full manuscript (Table S2 in the online only digital supplement). There were two reports; an interim analysis,[27] and a post hoc analysis[28] of a trial that was also reported in full.[10] Another trial compared NIRS values in on- versus of-pump CABG patients.[29] A fourth trial by Dullenkopf and colleagues[30] was reported in the abstract as an RCT, however the randomisation of placing the NIRS sensors on either the right or left for head and there was no clinical NIRS intervention versus a control comparison.

Included studies: Ten trials that evaluated NIRS based algorithms in patients undergoing cardiac surgery were identified.[10,18-25]

For peer review only

For peer review only - http://bmjopen.bmj.com/site/about/guidelines.xhtml

Participants: Overall, 1466 participants took part in the 10 trials included in the review. The average age of participants in these trials ranged from 34.6 ± 16.3 to 71 ± 11.2 years and the proportion of females ranged from 12.5% to 66% in the trials that provided this information.[10,18-25] The proportion of post randomisation withdrawals ranged from 0% to 4.11%. After withdrawal occurred, 1452 participants were included in the quantitative meta-analyses in this systematic review. Five trials were conducted in patients undergoing CABG only,[10,18,21,22,24] and 5 trials were conducted in patients undergoing valve or CABG and valve surgery or other cardiac surgical procedure.[19,20,23,25,26] Further details of participants are listed in Table S1 in the online only digital supplement.

Intervention: Cerebral NIRS values were measured with the INVOS (Somanetics Corporation, Troy, MI) in 9 trials. In 1 trial[19] three different devices were used; the INVOS, the FORE-SIGHT (CAS Medical Systems Inc., USA), EQUANOX Classic 7600 (Nonin Medical Inc., USA). In 2 trials the target NIRS values were >75% of baseline.[10,21] In 3 trials the targets values were >80%.[19,20,22,24] In 1 trial the target NIRS values were greater than an absolute NIRS value of 60% or >20% compared to the mean value during pulmonary artery catheter insertion.[25] In 2 trials the target NIRS value was a combination of >80% of baseline or an absolute measure > 50%.[18,23] In 1 trial the target regional oxygen saturation values were specified as >70% of pre-induction values or an absolute value of >50%.[26] Seven trials used a version of the Murkin algorithm to optimise NIRS values.[10,18-21,23,24,26] Two studies used non-Murkin algorithms.[22,25]

Control: All trials used standard (protocolised) care as the control group. In 7 trials NIRS values were measured in the control group, although this was hidden from the clinical staff.[10,19-21,23,24,26] No trial considered an alternative patient specific goal directed algorithm.

Co-interventions: In 2 trials the Murkin algorithm was combined with a restrictive transfusion trigger.[25,26] In these studies a pre-specified objective was to determine whether NIRS could be utilised as part of a patient specific red cell transfusion indicator.

For peer review only

For peer review only - http://bmjopen.bmj.com/site/about/guidelines.xhtml

Risk of bias in included studies

Risk of bias in individual trials is shown in Figure 2A, and the proportions of trials with low risk, unclear risk and high risk of bias in each of the domains are shown in Figure 2B. Clinical personnel were not blinded in any trial. However, 2 trials were considered at low risk of bias in every domain.[25,26]

Sequence generation: Random sequence generation was adequate in 7 trials,[10,18,19,21,24-26] and unclear in 2 trials.[20,23] There was a high risk of bias for random sequence generation in 1 trial.[22]

Allocation: Allocation concealment was adequate in 7 trials,[10,18,19,21,24-26] and unclear in 2 trials.[20,23] There was a high risk of bias for random sequence generation in 1 trial.[22]

Blinding: Theatre staff were unblinded in all of the studies. There was evidence of blinding of patients and clinical staff caring for patients postoperatively in 4 trials,[20,22,25,26] and unclear evidence in 6 trials.[10,18,19,21,23,24] Two trials reported the frequency of protocol compliance.[25,26] In 8 trials non-compliance was not monitored or not specified. There was evidence of blinding of outcome assessors in 5 trials [10,18,22,25,26] and unclear evidence of blinding of outcome assessors in 5 trials.[19-21,23,24]

Incomplete outcome data: 6 trials reported completeness of follow-up for the primary outcome.[10,19,21,22,25,26] Of these, 5 trials reported <10% loss to follow-up, and 1 trial reported >10% loss to follow-up.[25] Four trials that failed to report completeness of follow-up were considered to be at high risk of attrition bias.[18,20,23,24]

Selective reporting: Only 1 of the 10 trials included in this review had a published trial protocol.[26] Another 5 trials had reported details of primary and secondary outcomes in trial registries.[18-20,24,25] Of these, 3 trials failed to report all the pre specified outcomes.[18,20,24] The remaining trials were considered at high risk of selective outcome reporting bias.

Source of funding bias: Sources of funding were reported in 6 trials. Of these 2 trials were funded by independent sources. In 1 trial the study was supported in part by the NIRS device manufacturer and was therefore considered at high risk of funding bias.[10] Three studies that failed to report the source of funding

For peer review only - http://bmjopen.bmj.com/site/about/guidelines.xhtml

were considered at uncertain risk of funding bias (Table 1).

Effects of intervention

The summary effect estimates for primary and secondary outcomes are described in Table 1, Figure 2, and Figure S2 in the online only digital supplement.

Mortality: Four trials with 608 participants reported this outcome.[10,20,25,26] There was no statistically significant difference in mortality between NIRS versus controls; RR 0.76, 95% CI 0.30 to 1.96; I^2=0%; Chi^2 test for heterogeneity P value 0.92.

Stroke: Seven trials with 1138 participants reported this outcome.[10,18,19,23-26] There was no statistically significant difference in the frequency of stroke between NIRS versus controls; RR 1. 08, 95% CI 0.40 to 2.91; I2=0%; Chi2 test for heterogeneity P value 0.46.

Myocardial infarction: Six trials with 1038 participants reported this outcome.[10,18,19,24-26] There was no statistically significant difference in the frequency of myocardial infarction between NIRS versus controls; RR 0.90, 95% CI 0.43 to 1.89; I2=0%; Chi2 test for heterogeneity P value 0.86.

Severe Acute Kidney injury: Six trials with 1064 participants reported this outcome.[10,18,20,24-26] There was no statistically significant difference in the frequency of severe acute kidney injury between NIRS versus controls; RR 0.88, 95% CI 0.58 to 1.34; I2=0%; Chi2 test for heterogeneity P value 0.89.

Red cell transfusion: Four trials with 744 participants reported this outcome.[10,18,25,26] There was no statistically significant difference in the frequency of red cell transfusion between NIRS versus controls; RR 0.93, 95% CI 0.77 to 1.12; I2=51%; Chi2 test for heterogeneity P value 0.11.

Reoperation for Bleeding: Four trials with 744 participants reported this outcome.[10,18,25,26] There was no difference in the frequency of reoperation for bleeding between NIRS versus controls; RR 1.11, 95% CI 0.41 to 3.04; I2=0%; Chi2 test for heterogeneity P value 0.69.

For peer review only - http://bmjopen.bmj.com/site/about/guidelines.xhtml

Neurocognitive testing and measuring neurocognitive dysfunction: Five trials that recruited 813 patients reported this outcome.[18,22-24,26] One trial[19] reported only the incidence of delirium. Details of these trials are listed in Table 2. The Consensus Statement for the Assessment of Neurocognitive function in Cardiac Surgery[31] recommends that that the following Core tests be performed at baseline and up to 3 months post-surgery; the Rey auditory verbal learning test, the Trail-Making test Part A and Trail-Making Test Part B, and the Grooved Pegboard test to assess the neurocognitive domains Attention, Verbal Memory and Motor Coordination. The Consensus Statement defines cognitive decline as a difference for the individual of >1SD from baseline, or a difference of >1SD between group means, with adjustment for baseline for at least one test. There was significant heterogeneity for this outcome. Only 2 trials[24,26] measured cognitive function as recommended by the consensus statement. Both trials reported no difference between the groups for neurocognitive function. In 1 trial no test data was presented.[24] In the other[26] there was a significant difference between the groups for the Controlled Oral Word Association Test, which assesses the domain Executive Function/ Verbal Fluency, however this is not a specified core domain in the consensus statement. The three remaining trials used non-consensus testing protocols and non-consensus definitions of neurocognitive decline, and only 1 tested patients at 3 months post-surgery. Because of the degree of heterogeneity we did not perform meta-analyses of these outcome.

ICU LOS: Eight trials with 1051 participants reported this outcome.[10,18-20,22,23,25,26] There were no statistically significant differences in the duration of ICU stay between NIRS versus controls with moderate heterogeneity; RR MD 0.00, 95% CI -0.44 to 0.44; I^2=73%; Chi2 test for heterogeneity P value 0.0005.

Hospital LOS: Six trials with 761 participants reported this outcome.[10,19,20,22,25,26] Hospital LOS was less in the NIRS group however this was not statistically significant; RR MD -0.45, 95% CI -0.90 to 0.01; I^2=0%; Chi2 test for heterogeneity P value 0.83.

S100B: Two trials reported this outcome in 138 patients.[21,26] One trial with 40 participants measured S100B pre- and postoperatively and reported a significant reduction in S100B in NIRS patients, MD -99.87, 95% CI -105.18 to -94.56. The time of the post-surgery sample was not reported. Another with 98 participants

For peer review only

measured S100B preoperatively and at 4 post surgery time points; on return to ICU, and at 6, 12-24, 24-48 and 96 hours. There was no difference between the groups; Ratio of Geometric Means 1.06, 95% CI (0.95, 1.19), p=0.29. No meta-analysis was performed due to the heterogeneity for this outcome.

Subgroup analyses

Results of these subgroup analyses for Murkin vs non- Murkin algorithms, CABG vs non CABG, and assessment of neurocognitive function using tests described in a previous consensus statement are shown in Table S3 in the online only digital supplement.

Publication Bias: A funnel plot of standard error versus risk ratio for the included outcomes showed an asymmetrical distribution that indicated publication bias. However, since there was an insufficient number of trials providing data (less than 10 studies identified for each outcome), we did not perform this analysis.

Sensitivity Analyses

Fixed effects models did not materially change the results of our primary analyses. We also conducted sensitivity analyses in two trials identified as being at low risk of bias [25,26]. These trials reported outcomes in 328 participants. Both trials incorporated restrictive red cell transfusion thresholds within the NIRS algorithm. They reported no difference between NIRS and control groups for mortality: RR 0.67, 95% CI 0.11 to 4.08; I2=0%; Chi2 test for heterogeneity P value 0.71; stroke: RR 3.0 95% CI -0.32to 28.54; I2=0%; Chi2 test for heterogeneity P value 1.00; myocardial infarction: RR 0.76, 95% CI 0.17 to 3.41; I2=0%; Chi2 test for heterogeneity P value 0.66; severe acute kidney injury: RR 0.83, 95% CI 0.44 to 1.54; I2=0%; Chi2 test for heterogeneity P value 0.42; reoperation for bleeding: RR 1.50, 95% CI 0.48 to 4.62; I2=0%, Chi2 test for heterogeneity P value 0.75; ICU length of stay: RR-0.03, 95% CI -0.69 to 0.62; I2=0%; Chi2 test for heterogeneity P value 0.95 and hospital length of stay: RR -0.20, 95% CI -1.29 to 0.89; I^2=0%; Chi^2 test for heterogeneity P value 0.45. Analyses of these trials suggested that the use of NIRS based algorithms resulted in reductions in red cell transfusion: RR 0.83, 95% CI 0.71 to 0.98; I2=0%; Chi2 test for

For peer review only - http://bmjopen.bmj.com/site/about/guidelines.xhtml

heterogeneity P value 0.52, One of these trials, the PASPORT trial reported the results of neurocognitive assessments and concluded that there was no difference between NIRS and control groups.

GRADE Assessment

A summary of the main findings of the review are presented in Table 3. GRADE assessments of the results were either Low or Very Low for all the outcomes, indicating a high likelihood that these conclusions may be altered by subsequent trials.

For peer review only

For peer review only - http://bmjopen.bmj.com/site/about/guidelines.xhtml

Discussion

Main Findings

A systematic review and meta-analysis of existing trials did not demonstrate clinical benefits attributable to the use of personalised NIRS based algorithms during CPB. The use of NIRS did not result in reductions in mortality, injury to the brain, heart or kidneys, or reductions in resource use. A qualitative review of studies that had evaluated the effects of NIRS on neurocognitive function did not show clear evidence of benefit. An analysis of two trials at low risk of bias where NIRS was applied along with a restrictive red cell transfusion threshold demonstrated a reduction in red cell transfusion with this approach, with no difference between NIRS treated and controls with respect to clinical outcomes or resource use. Overall the GRADE quality of the evidence was low or very low for all of the outcomes measured.

Strengths and weaknesses

This is the most comprehensive evaluation of NIRS based patient specific goal direct algorithms in cardiac surgery to date. The results supersede those of a previous quantitative review of NIRS in cardiac surgery that considered randomised (2 studies) and observational analyses (27 studies).[32] Our searches also identified a Cochrane review protocol with similar aims however the results of this review have yet to be published.[33] The current review used comprehensive search strategies in a wide range of registries and data sources, had access to the full texts of all identified trials, used contemporary risk of bias assessments (GRADE), and assessed a wide range of outcomes after cardiac surgery. The main limitation of the review is that we did not have access to all of the source data. Although additional unpublished information was also obtained from three authors and included in the meta-analysis we realise that consistent analyses of all studies can only be done when data on individual patients are combined. In addition, the review identified important limitations of existing data; all of the 10 RCTs had limitations in terms of methodological quality. The risk of procedural bias was high in these trials as there was no blinding of clinical personnel. Furthermore only two from 10 trials attempted to define the likelihood of procedural bias by describing the degree of protocol adherence. The reporting of outcomes was also heterogeneous between trials limiting

For peer review only

For peer review only - http://bmjopen.bmj.com/site/about/guidelines.xhtml

the number of studies that could be included in each outcome. This was most evident for the outcome cognitive function. Many trials did not report important clinical outcomes; such as death, although it is highly likely that this outcome was measured. Furthermore, in many cases although the trial report stated that important assessments had been made, particularly with respect to the testing of cognitive function, the results of these assessments were not reported. Despite these limitations the results were remarkably consistent, with low or no heterogeneity for all of the analyses, all of which suggested that there are no clinical benefits attributable to the use of NIRS based algorithms.

Clinical Importance

NIRS technology and NIRS based algorithms are used in cardiac surgery centres worldwide, although there is clear evidence of equipoise with respect to the clinical benefits this technology.[12,13] The results presented here do not support the hypotheses that the use of NIRS may reduce brain injury,[8] or by using the brain as the index organ, reducing injury to the heart or kidneys as the result of improved overall perfusion.[9] It is possible that our results are attributable to chance; our GRADE assessment of the systematic review results was very low for all the pre-specified outcomes, indicating a high likelihood that these conclusions may be altered by subsequent trials. There was almost no heterogeneity for these outcomes, however, and these findings were consistent with the results of the PASPORT trial.[26] This multicentre trial recruited a larger cohort than almost all the previous trials, was at low risk of bias, and demonstrated no benefit for NIRS based algorithms for a range of outcomes including cognitive function, and biomarkers of myocardial, renal and neurological injury. [26]

Our systematic review indicated that the combination of NIRS and a restrictive transfusion threshold resulted in a reduction in red cell transfusion, without any difference between NIRS treated patients and controls with respect to clinical outcomes or resource use. This may be interpreted as showing that NIRS may be used safely to implement restrictive transfusion thresholds. These findings must be interpreted with caution however, these 2 trials enrolled only 387 patients, clinical staff were not blinded to group

For peer review only - http://bmjopen.bmj.com/site/about/guidelines.xhtml

For peer review only

allocation in these trials, and endpoints such as red cell transfusion are susceptible to performance bias. A final consideration is that the review did not identify any trial in paediatric cardiac surgery, a common setting for the use of these devices, and thus has identified a knowledge gap with respect to the utility of this intervention in these patients.

Conclusions

Existing evidence suggests that the use of NIRS based patient specific algorithms that aim to optimise cerebral oximetry do not result in reductions in mortality, major morbidity or resource use in adult cardiac surgery. Assessment of the quality of the evidence indicates that there is a need for further randomised trials at low risk of bias to assess the clinical utility of NIRS in both adult and paediatric cardiac surgery. Future studies should be powered to assess the effect of NIRS on important clinical outcomes.

Sources of Funding

This work was supported by the National Institute for Health Research Programme Grants for Applied Health number RP-PG-0407-10384. GFS is supported by the Leicester NIHR Cardiovascular Biomedical Research Unit. GJM is supported by British Heart Foundation Grants RG/13/6/29947 and CH/12/1/29419.

Disclosures

Both authors have completed the Unified Competing Interest form at www.icmje.org/coi_disclosure.pdf (available on request from the corresponding author). Dr Serraino declares no conflict of interest. Professor Murphy reports grants from British Heart Foundation during the conduct of the study and grants from British Heart Foundation, grants from National Institute for Health Research, grants from Zimmer Biomet, personal fees from Abbvie, and personal fees from Thrasos Inc, outside the submitted work.

Details of Contributors

Both of the study authors, external and internal, had full access to all of the data (including statistical reports and tables) in the study and can take responsibility for the integrity of the data and the accuracy of

For peer review only - http://bmjopen.bmj.com/site/about/guidelines.xhtml

the data analysis. Murphy conceived and designed the study, and undertook the systematic review. Serraino wrote the protocol, undertook the systematic review, performed the analysis and drafted the report along with Murphy. Both authors reviewed the report for important intellectual content and approved the final version.

Acknowledgements

The authors gratefully acknowledge the assistance of the Mr Giovanni Mariscalco who reviewed the study protocol for important intellectual content.

Data Sharing

No additional data available.

References:

1. Selnes OA, Gottesman RF, Grega MA, Baumgartner WA, Zeger SL, McKhann GM. Cognitive and neurologic outcomes after coronary-artery bypass surgery. N Engl J Med 2012; 366: 250-7
2. Murphy GJ, Angelini GD: Side effects of cardiopulmonary bypass: what is the reality? J Card Surg 2004; 19: 481-8
3. Kim MB, Ward DS, Cartwright CR, Kolano J, Chlebowski S, Henson LC. Estimation of jugular venous O2 saturation from cerebral oximetry or arterial O2 saturation during isocapnic hypoxia. J Clin Monit Comput 2000; 16: 191-9
4. Sapire KJ, Gopinath SP, Farhat G, et al. Cerebral oxygenation during warming after cardiopulmonary bypass. Crit Care Med 1997; 25: 1655-62
5. Croughwell N, Lyth M, Quill TJ, et al. Diabetic patients have abnormal cerebral autoregulation during cardiopulmonary bypass. Circulation 1990; 82: IV407-12
6. Miyoshi S, Morita T, Kadoi Y, Goto F. Analysis of the factors related to a decrease in jugular venous oxygen saturation in patients with diabetes mellitus during normothermic cardiopulmonary bypass. Surg Today 2005; 35: 530-4

For peer review only - http://bmjopen.bmj.com/site/about/guidelines.xhtml

7. Vretzakis G, Georgopoulou S, Stamoulis K, S et al. Cerebral oximetry in cardiac anesthesia. J Thorac Dis 2014; 6 Suppl 1: S60-9
8. Goldman S, Sutter F, Ferdinand F, Trace C. Optimizing intraoperative cerebral oxygen delivery using noninvasive cerebral oximetry decreases the incidence of stroke for cardiac surgical patients. Heart Surg Forum 2004; 7: E376-81
9. Murkin JM. Cerebral oximetry: monitoring the brain as the index organ. Anesthesiology 2011; 114: 12-3
10. Murkin JM, Adams SJ, Novick RJ, et al. Monitoring brain oxygen saturation during coronary bypass surgery: a randomized, prospective study. Anesth Analg 2007; 104: 51-8
11. Klein HG, Flegel WA, Natanson C. Red Blood Cell Transfusion: Precision vs Imprecision Medicine. JAMA 2015; 314: 1557-8
12. Kane JM, Steinhorn DM. Lack of irrefutable validation does not negate clinical utility of near-infrared spectroscopy monitoring: learning to trust new technology. J Crit Care 2009; 24: 472 e1-7
13. Zacharias DG, Lilly K, Shaw CL, et al. Survey of the clinical assessment and utility of near-infrared cerebral oximetry in cardiac surgery. J Cardiothorac Vasc Anesth 2014; 28: 308-16
14. Higgins JPT, Green S. Cochrane Handbook for Systematic Reviews of Interventions Version 5.1.0, The Cochrane Collaboration, 2011
15. Higgins JPT, Sterne JAC: Assessing risk of bias in included studies, Cochrane Handbook for Systematic Reviews of Interventions Version 5.1.0. Edited by Higgins JPT GS, The Cochrane Collaboration, 2011
16. Higgins JP, Thompson SG: Quantifying heterogeneity in a meta-analysis. Stat Med 2002; 21: 1539-58
17. Schünemann HJ, Vist GE, Higgins JPT, Deeks JJ, Glasziou P. Interpreting results and drawing conclusions, Cochrane Handbook for Systematic Reviewsof Interventions Version 5.1.0 Edited by Higgins JPT GS, The Cochrane Collaboration, 2011
18. Colak Z, Borojevic M, Bogovic A, Ivancan V, Biocina B, Majeric-Kogler V. Influence of intraoperative cerebral oximetry monitoring on neurocognitive function after coronary artery bypass surgery: a randomized, prospective study. Eur J Cardiothorac Surg 2015; 47: 447-54
19. Deschamps A, Hall R, Grocott H, et al. Cerebral Oximetry Monitoring to Maintain Normal Cerebral Oxygen Saturation during High-risk Cardiac Surgery: A Randomized Controlled Feasibility Trial. Anesthesiology 2016; 124: 826-36
20. Deschamps A, Lambert J, Couture P, et al. Reversal of decreases in cerebral saturation in high-risk cardiac surgery. J Cardiothorac Vasc Anesth 2013; 27: 1260-6
21. Harilall Y, Adam JK, Biccard BM, Reddi A. The effect of optimising cerebral tissue oxygen saturation on markers of neurological injury during coronary artery bypass graft surgery. Heart Lung Circ 2014; 23: 68-74
22. Kara I, Erkin A, Sacli H, et al. The Effects of Near-Infrared Spectroscopy on the Neurocognitive Functions in the Patients Undergoing Coronary Artery Bypass Grafting with Asymptomatic Carotid Artery Disease: A Randomized Prospective Study. Ann Thorac Cardiovasc Surg 2015; 21: 544-50
23. Mohandas BS, Jagadeesh AM, Vikram SB. Impact of monitoring cerebral oxygen saturation on the outcome of patients undergoing open heart surgery. Ann Card Anaesth 2013; 16: 102-6
24. Slater JP, Guarino T, Stack J, et al. Cerebral oxygen desaturation predicts cognitive decline and longer hospital stay after cardiac surgery. Ann Thorac Surg 2009; 87: 36-44; discussion 44-5

For peer review only - http://bmjopen.bmj.com/site/about/guidelines.xhtml

25. Vretzakis G, Georgopoulou S, Stamoulis K, et al. Monitoring of brain oxygen saturation (INVOS) in a protocol to direct blood transfusions during cardiac surgery: a prospective randomized clinical trial. J Cardiothorac Surg 2013; 8: 145
26. Reeves BC. A randomised controlled trial of patient-specific oxygen monitoring to reduce blood transfusion during heart surgery, Biomed central, 2009, ISRCTN23557269
27. Murkin JM AS, Schaefer B. Monitoring cerebral oxygen saturation significantly decreases major organ morbidity in CABG patients: A randomized blinded study. Heart Surg Forum 2004; 7: 515
28. Murkin JM, Adams SJ, Pardy E, et al. Monitoring brain oxygen saturation during coronary bypass surgery improves outcomes in diabetic patients: a post hoc analysis. Heart Surg Forum 2011; 14: E1-6
29. Kok WF, van Harten AE, Koene BM, et al. A pilot study of cerebral tissue oxygenation and postoperative cognitive dysfunction among patients undergoing coronary artery bypass grafting randomised to surgery with or without cardiopulmonary bypass*. Anaesthesia 2014; 69: 613-22
30. Dullenkopf A, Baulig W, Weiss M, Schmid ER. Cerebral near-infrared spectroscopy in adult patients after cardiac surgery is not useful for monitoring absolute values but may reflect trends in venous oxygenation under clinical conditions. J Cardiothorac Vasc Anesth 2007; 21: 535-9
31. Murkin JM, Newman SP, Stump DA, Blumenthal JA. Statement of consensus on assessment of neurobehavioral outcomes after cardiac surgery. Ann Thorac Surg 1995; 59: 1289-95
32. Zheng F, Sheinberg R, Yee MS, Ono M, Zheng Y, Hogue CW. Cerebral near-infrared spectroscopy monitoring and neurologic outcomes in adult cardiac surgery patients: a systematic review. Anesth Analg 2013; 116: 663-76
33. Yu Y ZK, Zhang L, Zong H, Meng L, Han R. Cerebral near-infrared spectroscopy (NIRS) for perioperative monitoring of brain oxygenation in children and adults. Cochrane Database of Systematic Reviews 2014; Issue 1. CD010947.

For peer review only

For peer review only - http://bmjopen.bmj.com/site/about/guidelines.xhtml

Table 1. Summary of findings for all primary and secondary outcomes

STUDY	*MORTALITY*		*MI N (%)*		*STROKE N (%)*		*AKI N (%)*		*BLEEDING N (%)*		*TRANSFUSION N (%)*		*ICU LOS (H) MEAN (SD)*		*HOSP LOS (D) MEAN (SD)*	
	NIRS	Control	NIRS	Control	NIRS	Control	NIRS	Control	NIRS	Control	NIRS	Control	NIRS	Control	NIRS	Control
**PASPORT, 2017[26]**	1 (1)	2 (1.88)	1 (1)	2 (1.88)	1 (1)	0	2 (2)	5 (4.71)	6 (6.1)	4 (3.77)	37 (37.7)	44 (45.8)	3.3 (0.81)	3.67 (0.79)	7.5 (1.15)	8 (1.17)
**DESCHAMPS, 2016 [19]**	4 (11.7)	6 (13)	0	0	1 (2.9)	2 (4.3)	12 (35.2)	17 (47.2)	-	-	-	-	2.99 (2.26)	0.39 (2.05)	11 (7.2)	9.9 (5.8)
**KARA, 2015[22]**	-	-	-	-	-	-	-	-	-	-	-	-	1.74 (0.81)	2.12 (1.05)	7.15 (1.39)	7.67 (1.14)
**COLAK, 2015[18]**	-	-	8 (8.5)	7 (7.29)	4 (4.25)	1 (1)	0	1 (1)	0	1 (1)	77 (81)	73 (76)	2.7 (6.2)	1.9 (0.9)	-	-
**DESCHAMPS, 2013[20]**	-	-	-	-	-	-	-	-	-	-	-	-	2.99 (2.26)	0.39 (2.05)	7.6 (5.4)	7.9 (3.2)
**VRETZAKIS, 2013[25]**	1 (1.33)	1 (1.33)	2 (2.66)	2 (2.66)	1 (1.33)	0	1 (1.33)	0	1 (1.33)	1 (1.33)	51 (68)	63 (84)	2.7 (3.8)	2.7 (3.6)	10.9 (3.6)	10.2 (10.7)
**MOHANDAS, 2013[23]**	-	-	-	-	0	2 (4)	-	-	-	-	-	-	1.49 (0.38)	1.7 (0.49)	-	-
**HARILALL, 2013[21]**	-	-	-	-	-	-	-	-	-	-	-	-	-			

For peer review only - http://bmjopen.bmj.com/site/about/guidelines.xhtml

**SLATER, 2008**[24]	-	-	1 (0.8)	1 (0.86)	1 (0.8)	0	1 (0.8)	1 (0.86)	-	-	-	-	-	-	-	
**MURKIN, 2007**[10]	0	1 (1%)	1 (1%)	3 (3%)	1 (1%)	4 (4%)	0	0	1 (1%)	1 (1%)	8 (8)	10 (10)	1.25 (0.84)	1.87 (2.67)	6.1 (4.4)	6.9 (5.5)
**SUMMARY EFFECT ESTIMATE RR(95% CI)**	**0.76 (0.30 to 1.96)**		**0.90 (0.43 to 1.89)**		**1.08 (0.40 to 2.91)**		**0.88 (0.52 to 1.49)**		**1.23 (0.46 to 3.32)**		**0.93 (0.77 to 1.12)**		**0.00 (-0.44 to 0.44)**		**-0.45 (-0.90 to 0.01)**	

MI, Myocardial Infarction, AKI, Acute Kidney Injury, ICU, Intensive Care Unit, Hosp, Hospital, Length of Stay, LOS.

For peer review only

For peer review only - http://bmjopen.bmj.com/site/about/guidelines.xhtml

Table 2. Assessment of Neurocognitive Dysfunction

**Domain**	**Test Protocol**	**Kara, 2015[22]**	**Colak, 2015[18]**	**PASPORT 2017[26]**	**Mohandas, 2013[23]**	**Slater, 2008[24]**
**Timing**		Baseline and and pre-discharge	Baseline and 7 days	Baseline, 7 days and three months	Baseline, 7 days and three months	Baseline,pre-discharge and three months
**Attention**	Trail- Making Test parts A and B or equivalent		X (CTT)	X		X (SCW)
**Verbal Memory**	Rey Auditory Verbal Learning Test (VLT) or Hopkins VLT			X		X
**Visuo-Spatial**	Block Design from the Wechsler Adult Intelligence Scale			X		
**Psychomotor Speed**	Digit Symbol Test from the Wechsler Adult Intelligence Scale			X		
**Executive Funtion/ Verbal Fluency**	Controlled Oral Word Association Test			X		
**Motor Cordination**	Grooved Pegboard Test		X	X		X
**Other**	Saccadic and anti-saccadic eye movements		X		X	X
	Montreal Cognitive Asessment	X				
**Measurement of Confounders**						
	MMSE		X	X	X	X
	Anxiety Depression			X		X

For peer review only

Domain	Test Protocol	Kara, 2015[22]	Colak, 2015[18]	PASPORT 2017[26]	Mohandas, 2013[23]	Slater, 2008[24]
	Adult Reading Test			X		
	Medication			X		
	Delirium Rating Score					X
**Presented Test Data**		X		X	X	
**Definition of Cognitive Decline**		The maximum score to get from this test is 30.<br>>25=Normal 19–25= mild cognitive imp. <19= serious cognitive imp..	Dichotomous:decrease in an MMSE score for three or more points from baseline value<br>AND<br>decrease of 1 SD (standard deviation) or more in performance on CTT 1 and GP tests.	Difference in group mean (p<0.05) in >3 of 6 domains	Postoperative MMSE impairment was defined as a decrease in scores by more than 20% of the preoperative values. Postoperative ASEM impairment was defined as a decrease of scores to more than 30% of preoperative values.	Cognitive decline was defined as a decline of one standard deviation or more in performance on one or more of the neuropsychologic tests.
**Reportd Difference in Cognitive Function**		X	X		X	
**Estimate of Difference**		Mild Cognitive Impairment 7/43 NIRS, 16/36 Control, p=0.01.Severe Cognitive Impairment 0/42 NIRS, 3/36 Control, p-0.09	28% NIRS vs 52% Control, p=0.0002	Significant difference in COWAT Mean Difference 3.73 (95%CI 1.5 to 5.96), p=0.0011	ASEM at 3 months Mean 15.69 (SD 3.99) NIRS, Mean 17.68 (SD 1.79), p<0.001	58% NIRS vs 61% Control

CTT: Colour Trail Test 1, SCW: Stroop Colour and Word; GP Test: Grooved Pegboard Test; MMSE: Mini- Mental State Examinations; ASEM: Anti saccadic eye

For peer review only - http://bmjopen.bmj.com/site/about/guidelines.xhtml

movements.

For peer review only

For peer review only - http://bmjopen.bmj.com/site/about/guidelines.xhtml

Table 3. Summary of main findings of systematic review and Grade Assessment of trial results.

**Near Infra Red Spectrodscopy Algorithm compared to Control (Standard Care) in Cardiac Surgery**					
**Patient population:** Adult Cardiac surgery, **Setting:** Tertiary cardiac centres					
**Intervention:** Near Infra Red Spectroscopy Algorithms for Personalised Optimisation of Cerebral Oxygenation					
**Control:** Standard care					
Outcomes	Anticipated absolute effects* (95% CI)		Relative Effect (95% CI)	N of Participants (studies)	Quality og the evidence (GRADE)
	Risk with Control	Risk with NIRS			
Mortality	32 per 1000	**25 per 1000** (10 to 63)	**RR 0.76** (0.30 to 1.96)	608 (4 RCTs)	⊕⊕◯◯ Low
Red cell transfusion	504 per 1000	**469 per 1000** (388 to 564)	**RR 0.93** (0.77 to 1.12)	744 (4RCTs)	⊕⊕◯◯ Low
Stroke	16 per 1000	**17 per 1000** 6 to 46)	**RR 1.08** (0.40 to 2.91)	1138 (7 RCTs)	⊕◯◯◯ Very Low
Myocardial infarction	29 per 1000	**26 per 1000** (12 to 54)	**RR 0.90** (0.43 to 1.89)	1038 (6 RCTs)	⊕◯◯◯ Very Low

For peer review only

For peer review only - http://bmjopen.bmj.com/site/about/guidelines.xhtml

Renal failure	71 per 1000	**62 per 1000**<br>(41 to 95)	**RR 0.88**<br>(0.58 to 1.34)	1043 (6 RCTs)	⊕◯◯◯<br>Very Low
Reoperation for bleeding	19 per 1000	**21 per 1000**<br>(8 to 56)	**RR 1.11**<br>(0.41 to 3.04)	744 (4 RCTs)	⊕◯◯◯<br>Very Low
ICU length of Stay<br>(ICU LOS)		The mean ICU LOS in the intervention group was 0 (0.44 lower to 0.44 higher)			⊕◯◯◯<br>Very Low
Hospital length of stay<br>(H LOS)		The mean H LOS was 0.45 lower<br>(0.9 lower to 0.01 higher)			⊕⊕◯◯<br>Low
***The risk in the intervention group** (and its 95% confidence interval) is based on the assumed risk in the comparison group and the **relative effect** of the intervention (and its 95%CI)					
CI: Confidence interval; RR: Risk ratio; MD: Mean Difference; ICU: Intensive Care Unit; NIRS: Near Infra-red Spectroscopy					

For peer review only

Figure Legends

Figure 1. Risk of bias summaries for **A.** Individual studies and **B.** All studies**.**

Figure 2. Forrest plots demonstrating summary effect estimates for A. Mortality**, B.** Myocardial Infarction**, C.** Stroke**, D.** Stage 3 acute kidney injury or renal replacement therapy**.** Effect estimates derived using Random Effects meta-analysis. Heterogeneity expressed as Chi Squared and I^2.

For peer review only

For peer review only - http://bmjopen.bmj.com/site/about/guidelines.xhtml

For peer review only

For peer review only - http://bmjopen.bmj.com/site/about/guidelines.xhtml

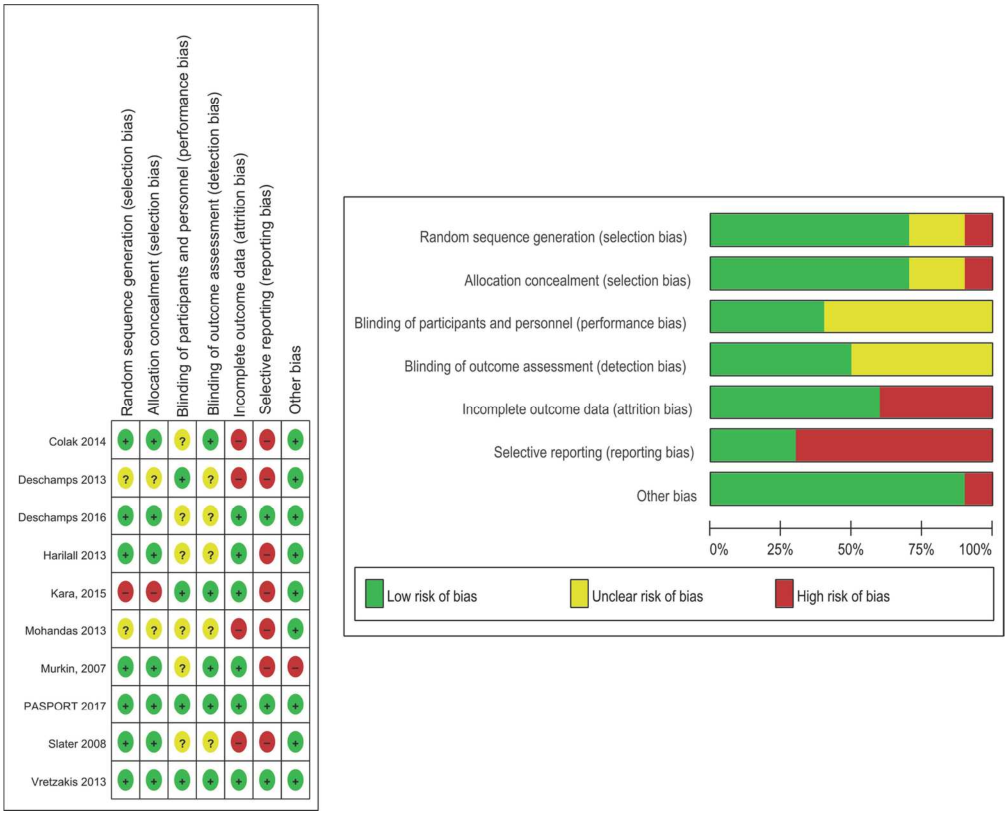

Figure 1

142x114mm (300 x 300 DPI)

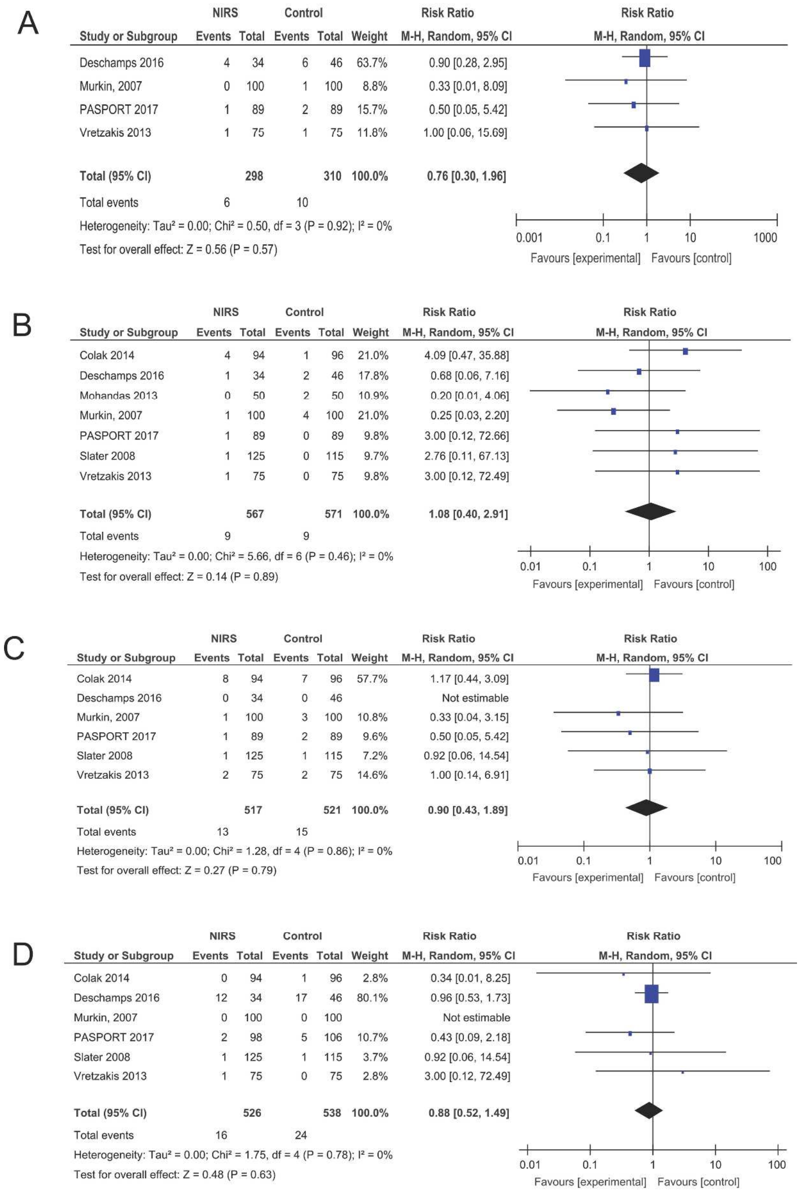

Figure 2

255x386mm (300 x 300 DPI)

Online only digital supplement: Efficacy of Near-Infrared Spectroscopy on the outcome of patients undergoing cardiac surgery: a systematic review of randomised trials.

Authors: GF Serraino, GJ Murphy

Expanded Methods

Types of Studies

Randomized controlled trials irrespective of blinding, language, publication status, date of publication and sample size.

Types of Participants

Patients undergoing cardiac surgery for acquired or congenital disease or aortovascular disease with or without cardiopulmonary bypass were included. No age restriction was applied. There were no exclusion criteria.

Types of Interventions

Intervention: Goal directed NIRS algorithm.

Comparator /control: An untreated group, or alternative (non NIRS based) goal directed therapy.

Types of Outcome Measure

Primary outcomes:

Mortality: 30 day or hospital all-cause mortality.

Secondary outcomes:

1. Acute brain injury; stroke, TIA as defined by study authors.
2. Low cardiac output as defined by study authors.
3. Myocardial Infarction as defined by study authors.
4. Acute kidney Injury Stage 3 or requiring hemofiltration as defined by study authors.
5. Neurocognitive function; group means as described by neurocognitive tests. Tests recommended by a consensus statement to test all key domains of cognitive function [31] are marked with *. Studies were categorised as Yes/ No as to whether they had assessed the key domains described in the

For peer review only

Consensus Statement. Key domains that were assessed [32] and examples of likely tests that were recorded are as follows:

- Attention: Sustained and divided attention: Consensus statements recommend the Trail-Making Test parts A* and B* [33,34].
- Verbal memory: Consensus statements recommend the RAVLT and Rey Auditory Verbal Learning Test* [34]
- Visual-spatial: as the Block Design from the Wechsler Adult Intelligence Scale. [35]
- Psychomotor speed: Consensus statements recommend tests such the Digit Symbol Test from the Wechsler Adult Intelligence Scale. [35]
- Executive function/Verbal fluency: Consensus statements recommend tests such the Controlled Oral Word Association Test. [36]
- Motor coordination: Consensus statements recommend tests such the Grooved Pegboard Test*, dominant and non-dominant hand. [33]
- Frontal Lobe: Saccadic and anti-saccadic eye movements.

Assessment of important covariates (Mini Mental State, Anxiety and Depression, Intellectual Ability, and Concomitant medication) in these analyses were documented.

6. Neurocognitive dysfunction, as reported by the study authors; A consensus definition is a change in a single test of >1 SD. This was defined as either change >1 SD in a group mean (adjusted for baseline) or a change in neurocognitive scores of >1 SD for individual patients.[37] Studies were further categorised as defining cognitive dysfunction using a Consensus versus a non-Consensus definitions.
7. Risk of receiving blood transfusion as defined by study authors.
8. Reoperation for bleeding as defined by study authors.
9. Resource Use: ICU and hospital LOS as defined by study authors.
10. S100B levels, a marker of brain injury, as reported by study authors.

Search methods for identification of studies

For peer review only

Potentially eligible trials were identified by searching the *Cochrane Central Register of Controlled Trials*, *MEDLINE* , and *EMBASE* and the *Cumulative Index to Nursing and Allied Health Literature (CINAHL) Plus*, using a combination of subject headings and text words to identify relevant trials. The Medline search strategy below was adapted as appropriate for other databases:

((Cardiopulmonary Bypass) OR (Cardiac Surgery) OR (Coronary Artery Bypass) OR (Extra Corporeal Circulation) OR (Perioperative Morbidity)) AND ((Near Infra-Red Spectroscopy) OR (Oximetry) OR (Brain/Metabolism) OR (Cerebral Desaturation) OR (Cerebral Perfusion) OR (Cerebral Ischemia) OR (Cerebral Oximetry) OR (Cerebral Saturation) OR (Near Infrared Oximetry) OR (Cognitive)).

To identify on going or unpublished trials we also searched the Clinicaltrials.gov using the following search terms:

Search terms: Randomized

Study Type: Interventional Studies

Conditions: ((Cardiopulmonary Bypass) OR (Cardiac Surgery) OR (Coronary Artery Bypass) OR (Extra Corporeal Circulation) OR (Perioperative Morbidity))

Interventions: ((Near Infra-Red Spectroscopy) OR (Oximetry) OR (Brain/Metabolism) OR (Cerebral Desaturation) OR (Cerebral Perfusion) OR (Cerebral Ischemia) OR (Cerebral Oximetry) OR (Cerebral Saturation) OR (Near Infrared Oximetry) OR (Cognitive))

We will also examined the reference lists of eligible trials and reviews. Searches were not restricted by language or publication status.

Data Collection and Analysis

The review was performed in accordance with instructions given in the Cochrane Handbook for Systematic Reviews of Interventions [38].

Selection of Studies

Two reviewers GJM and GFS identified trials for inclusion independently of each other. Excluded studies and the reason for exclusion were recorded.

Data extraction (selection and coding)

For peer review only

Two authors independently screened the search output to identify records of potentially eligible trials examining the outcomes, the full texts of which were retrieved and assessed for inclusion.

A standardised form was used to extract data from the included studies for assessment of study quality and evidence synthesis. Extracted information included:

- Year and language of publication
- Country of Participant recruitment
- Year of conduct of the trial
- Study setting; university teaching hospital, non-university teaching hospital
- Study population; inclusion and exclusion criteria
- Sample size
- Participant demographics
- Baseline characteristics
- Type of surgery
- Details of NIRS algorithm (Murkin, non Murkin[39]) and co-interventions (restrictive versus non-restrictive transfusion thresholds)
- Details of Comparator; non NIRS goal directed therapy, standard care (protocolised care)
- Outcomes and times of measurement

Two review authors extracted data independently; discrepancies were identified and resolved through discussion. Missing data was requested from study authors. If there is doubt as to whether trials shared participants completely or partially (with common authors and centres) we contacted the study authors to ascertain whether the study report has been duplicated.

Risk of Bias

The following bias risk domains were assessed as Low, Uncertain, or High based on the instructions given in the in the Cochrane Handbook for Systematic Reviews of Interventions [15];

- Sequence Generation
- Allocation Concealment

For peer review only - http://bmjopen.bmj.com/site/about/guidelines.xhtml

- Blinding of participant, personnel
- Blinding of outcome Assessors
- Incomplete outcome data
- Selective outcome reporting
- Source of funding bias

Trials were classified as having a low risk of bias if they are graded as being at low risk of bias in all of these domains. Two review authors independently assessed the risk of bias in all of the studies. Discrepancies were resolved by discussion.

Assessment of Reporting Bias

Where 10 or more studies were identified for each outcome we assessed publication bias by the visual assessment of funnel plots and Eggers test [40].

Measures of treatment Effect

For dichotomous variables, we calculated the risk ratio (RR) with 95% confidence interval (CI). For continuous variables, we calculated the mean difference (MD) with 95% CI for outcomes such as hospital stay, and standardised mean difference (SMD) with 95% CI for quality of life (when different scales were used).

Dealing with missing data

We perform an intention-to-treat analysis where possible. For dichotomous data presented only as percentages we estimated frequencies using reported sample sizes for this outcome. For continuous outcomes if the mean and the standard deviation were not available from the trial report, we sought this information from the trial authors. If this information was still not available, we calculated the mean and standard deviation from median (interquartile ranges) using the software available in Review Manager Version 5.

Assessment of Heterogeneity

We anticipated that major sources of clinical heterogeneity would associated with different patient groups (adults, children, congenital versus acquired disease), the use of different goal directed NIRS algorithms,

For peer review only

the use of co-interventions such as restrictive transfusion thresholds, and differences in the methodology used to assess neurocognitive dysfunction. We explored heterogeneity within each meta-analysis using a Chi^2 test with significance set at a P value of 0.10, and expressed the percentage of heterogeneity due to variation rather than to chance as I^2.[16] We defined heterogeneity as follows:

I^2 0-40%: no or mild heterogeneity

I^2 40-80%: moderate heterogeneity

I^2 > 80%: severe heterogeneity

In the presence of severe heterogeneity meta-analysis was not performed.

Data Synthesis

Meta-analyses was performed using the software package Review Manager version 5.2 and in accordance with the recommendations of the *Cochrane Handbook for Systematic Reviews of Intervention* [14]. For the primary analysis we compared the results of a random-effects model versus a fixed effects model to assess the effects of small studies. For continuous outcomes, pooled mean differences or standardised mean differences by using the inverse variance method.

Subgroup Analyses

Subgroup analyses was performed in trials in which the Murkin algorithm was used to guide goal directed therapy versus those that did not, and by participant group; CABG versus non CABG, adults versus children, assessment of neurocognitive function that incorporated tests described in a previous consensus statement, and studies that combined the NIRS algorithm with a restrictive red cell transfusion trigger. Test for sub-group differences with Review Manager was used with a P value of <0.05 considered statistically significant.[17]

Sensitivity Analyses

Sensitivity analysis excluded trials with high risk of bias for any of the following: random sequence generation; allocation concealment, blinding of participants, healthcare providers or outcome assessors, incomplete outcome data, attrition, and other sources of bias including source of funder.

Summary of findings

For peer review only

We presented the main results of the review in a 'Summary of findings' table. We included the following outcomes.

- Risk of mortality
- Risk of stroke, myocardial infarction, or severe acute kidney injury
- Risk of red cell transfusion
- Neurocognitive Impairment
- Resource Use: ICU and Hospital length of stay.

We used GRADEpro software to prepare the 'Summary of findings' table. We judged the overall quality of the evidence for each outcome as 'high,' 'moderate,' 'low' or 'very low' according to the GRADE (Grades of Recommendation, Assessment, Development and Evaluation) approach [17]. We considered the following.

- Impact of risk of bias of individual trials.
- Precision of pooled estimate. Inconsistency or heterogeneity (clinical, methodological and statistical).
- Indirectness of evidence.
- Impact of selective reporting and publication bias on effect estimate.

For peer review only

Table S1. Characteristics of Included Studies.

STUDY	*Location/ Funding*	*Sample size*	*Period*	*Inclusion/ Exclusion Criteria*	*Mean Age -intervention group -control group* ***Female (%)*** *- Intervention group -control group*	*NIRS Device*	*Murkin Algorithm*	*Control*	*Outcomes*
Deschamps, 2016 [19]	Multicentre<br>Regional funders<br>Canada	201	2012 – 2013	Incl: high-risk cardiac surgery<br>Excl: Off -pump coronary artery bypass surgery, emergency surgery, planned deep hypothermic circulatory arrest, acute endocarditis, presence of active delirium or encephalopathy.	69±12.6<br>72±9.4<br>27.4%F<br>28.3%F	FORE-SIGHT, EQUANOX Classic 7600, INVOS 5100C-PB		SC	Primary Outcome: success rate of reversing cerebral desaturations below 10% relative to baseline in the intervention group<br>Secondary Outcomes: cerebral desaturation load, 30-day follow-up for adverse events

For peer review only - http://bmjopen.bmj.com/site/about/guidelines.xhtml

Kara, 2015[22]	Single centre, Sakarya, Turkey	79	2013-2015	Incl: CABG<br>Excl: Other procedures, high degrees of aortic atherosclerosis	59.1±9.4<br>61.2±10.3<br>23.3%F<br>19.4%F	INVOS	SC	Primary: Cognitive impairement (MoCa score)<br>Secondary: ICU and H LOS
PASPORT, 2017[26]	3 UK centres<br>NIHR funded	204	2009-2014	Incl: CABG and non CABG.<br>Excl: Patients undergoing emergency cardiac surgery<br>Patients who are prevented from having blood and blood products according to a system of beliefs.	70±10<br>65.9±12.3<br>30%F<br>33%F	?	SC	Primary: Cognitive function<br>Secondary: Units of RBC; Cerebral oxygenation; Oxygen delivery and utilisation during CPB: Quality of life; Infectious complications; Stroke; MI; ICU and H LOS; Post-operative acute kidney injury; Respiratory complications; Health and personal social services resource use and their costs. All-cause mortality within 30 days of surgery; Biochemical markers of organ injury
Colak, 2015[18]	Single centre, Zagreb, Croatia<br>Insitutional support grant	200	2009-2010	Incl: CABG<br>Excl: carotid artery stenosis, previous stroke or head injury, seizure, psychiatric illness, (NYHA III/IV) LVEF<25%, emergency off-pump CABG and severely impaired renal and liver function.	61.9±7.1<br>63.4±8.8<br>20%F<br>24%F	INVOS	SC	Primary: Cognitive impairement<br>MMSE<br>CTT1<br>GP test<br>Secondary: coma, stupor, transient ischaemic attack (TIA) or stroke, neuropsychological deficits

For peer review only

Deschamps, 2013[20]	Single centre, Quebec, Canada. Insitutional support grant	49	NS	Incl: CABG and non-CABG<br>Excl: Emergency surgery, first time CABG surgery, single valve surgery	70.2±9.2<br>71.1±7.9<br>15%F<br>15%F	INVOS	X	SC	Primary: rSO2<br>Secondary: ICU and H LOS
Vretzakis, 2013[25]	Single centre, Greece Insitutional support grant	50	16-month period	Incl: CABG and non CABG<br>Excl: Emergency, re-do operations, combined cardiac - carotid surgery circulatory arrest. hematologic disease coagulation abnormality, advanced cirrhosis and renal dysfunction	67.3±8.5<br>65.9±9.5<br>12%F<br>15%F	INVOS		SC	Primary: RBC transfusion,<br>ICU and H LOS<br>Death<br>Secondary: Major complications
Mohandas, 2013[23]	Single centre, Bangalore, India<br>RCT	100	NS	Incl: On pump<br>Cardiac surgery<br>Excl: pre-existing neuropsychiatric disorders, inability to correctly perform the neurocognitive tests, and mini-mental state examination (MMSE) scores of less than 23	38.05±15.81<br>34.60±16.28<br>44%F<br>44%F	NONIN EQUANOX	X	SC	Primary: Cognitive decline:<br>MMSE<br>ASEM<br>Secondary: ICU LOS

For peer review only

Harilall, 2013[21]	Single centre, South Africa	40	NS	Incl: CABG<br><br>Excl: pregnancy, history of stroke or per- sistent neurological residue, history of transient ischaemic attack (TIA), stenosis of carotid artery	55.3±9.7<br><br>30%F (all)	INVOS	X	SC	Primary: rSO2<br><br>Secondary: S100B
Slater, 2008[24]	Single centre, New Jersey, USA	265	2004-2006	Incl: CABG<br><br>Excl: preexisting neuropsychiatric disorders, inability to correctly perform the neurocognitive tests, and mini- mental state examination score of 23 or less.	64.8±10.1<br>64.78±9.9<br><br>31%F (all)	INVOS	X	SC	Primary: Cognitive impairement<br><br>Secondary: ICU LOS
Murkin, 2007[10]	Single centre, Ontario, Canada<br><br>Industry support	200	2002 – 2004	Incl: >18 yr, CABG	61.8±10.3<br>61.8±9.3<br><br>12.5%F (all)	INVOS	X	SC	Primary: 30 days Death, MMOM<br><br>Secondary: LOS

Table S2. Characteristics of Excluded Studies

STUDY	*Study Type/ Location*	*SAMPLE*	*PERIOD*	*INCLUS. CRIT.*	*EXCLUS. CRIT.*	*Mean Age*	*Female (%)*	*NIRS Device*	*Murkin Algorithm*	*Control*	*OUTCOMES*
Dullenkpof, 2007 [30]	Prosp, RCT, Single centreZurich, Switzerland **NO intervention**	35	NS	Elective cardiac surgery with insertion of PAC	Cerebral perfusion disturbence, CAD, intracardiac shunt	65.5±10.9	74.2	INVOS			Primary: rSO2
Kok, 2014 [29]	Single centre Groningen NH	59	2011 – 2012	CABG	Other procedures, difficulty completing cognitive tests, difficulty with dutch lang, impaired finction of the dominant arm or hand, history of head trauma, stroke or neurosurgery, severe CAD	62.8±9.4	10	Somanetics (Michigan) Casmed (Branford)		On pump vs Off Pump surgery	Primary: Cerebral desaturation Secondary: Postop cognitive disfunction; major complications; ICU and Hospital LOS

PAC pulmonary artery catheter, CABG coronary artery bypass grafts, ICU intensive care unit, LOS lengthof stay

For peer review only

Table S3. Sub Group Analyses

SUB GROUP	CABG	NON CABG	TEST FOR SUBGROUP DIFFERENCES	MURKIN	NON MURKIN	TEST FOR SUBGROUP DIFFERENCES	CONSENSUS NEUROCOGNITIVE ASSESSMENT	NON CONSENSUS NEUROCOGNITIVE ASSESSMENT	TEST FOR SUBGROUP DIFFERENCES	RESTRICTIVE RED CELL TRANSFUSION TRIGGER	NO RESTRICTIVE RED CELL TRANSFUSION TRIGGER	TEST FOR SUBGROUP DIFFERENCES
**MORTALITY RR (95% CI)**	0.33 (0.01 to 8.09) 1 trial, n=200, I^2=NA	0.81 (0.30 to 2.18) 3 trials, n=408, I^2=0%	**Chi^2 = 0.50, df = 3 (P = 0.92), I^2 = 0%**	0.77 (0.26 to 2.32) 2 trials, n=289, I2 =NA	0.67 (0.11 to 3.94) 2 trials, n=328, I^2=0%	**Chi^2 = 0.50, df = 3 (P = 0.92), I^2 = 0%**	0.50 (0.05 to 5.42) 1 trial, n=178 I2=NA	0.94 (0.39 to 2.26) 3 trials, n=430 I2=4%	**Chi^2 = 0.23, df = 3 (P = 0.97), I^2 = 0%**	0.67 (0.11 to 3.94) 2 trials, n=328 I2=0%	0.77 (0.26 to 2.32) 2 trials, n= 280 I2= NA	**Chi^2 = 0.50, df = 3 (P = 0.92), I^2 = 0%**
**STROKE RR (95% CI)**	1.18 (0.38 to 3.63) 3 trials, n=630. I2 =43%	0.89 (0.26 to 3.10) 4 trials, n= 508 I2=1%	**Chi^2 = 5.66, df = 6 (P = 0.46), I^2 = 0%**	0.84 (0.33 to 2.11) 5 trials, n=810 I2=14%	3.00 (0.32 to 28.54) 3 trials, n=328 I2=0%	**Chi^2 = 5.66, df = 6 (P = 0.46), I^2 = 0%**	3.00 (0.12 to 72.66) 1 trial, n=178 I2: NA	1.00 (0.39 to 2.56) 6 trials, n=960 I2=22%	**Chi^2 = 5.66, df = 6 (P = 0.46), I^2 = 0%**	3.00 (0.32 to 28.54) 2 trials, n=328 I2=0%	0.84 (0.33 to 2.11) 5 trials, n=810 I2=14%	**Chi^2 = 5.66, df = 6 (P = 0.46), I^2 = 0%**
**MI RR (95% CI)**	0.92 (0.40 to 2.10) 3 trials, n=630, I2=0%	0.75 (0.17 to 3.30) 3 trials, n=408, I2=0%	**Chi^2 = 0.05, df = 1 (P = 0.82), I^2 = 0%**	0.92 (0.40 to 2.10) 4 trials, n=710, I2=0%	0.75 (0.17 to 3.30) 2 trials, n=328 I2=0%	**Chi^2 = 0.05, df = 1 (P = 0.82), I^2 = 0%**	0.50 (0.05 to 5.42) 1 trial, n=178 I2= NA	0.93 (0.43 to 1.99) 5 trials, n=860 I2=0%	**Chi^2 = 0.24, df = 1 (P = 0.63), I^2 = 0%**	0.75 (0.17 to 3.30) 2 trials, n=328 I2=0%	0.92 (0.40 to 2.10) 4 trials, n=710 I2=0%	**Chi^2 = 0.05, df = 1 (P = 0.82), I^2 = 0%**
**SEVERE AKI RR (95% CI)**	0.58 (0.08 to 4.41)	0.88 (0.51 to 1.12)	**Chi^2 = 0.15, df = 1 (P = 0.70), I^2 = 0%**	0.90 (0.51 to 0.19)	0.67 (0.18 to 2.56)	**Chi^2 = 0.15, df = 1 (P = 0.70), I^2 = 0%**	3.00 (0.12 to 72.49) 1 trial, n=150	0.96 (0.55 to 1.68) 3 trials, n=780	**Chi^2 = 0.48, df = 1 (P = 0.49), I^2 = 0%**	0.67 (0.18 to 2.56) 2 trials, n=354	0.90 (0.51 to 1.19) 2 trials, n=630	**Chi^2 = 0.15, df = 1 (P = 0.70), I^2 = 0%**

For peer review only

	3 trials, n=630, I2=0%	4 trials, n=434, I2=0%		4 trials, n=710 I2=0%	2 trials, n=354 I2=12%		I2=NA	I2=0%		I2= 12%	I2=0%	
**TRANSFUSION RR (95% CI)**	1.04 (0.89 to 1.23) 2 trials, n=390. I2=0%	0.85 (0.71 to 1.01) 2 trials, n=354. I2=0%	**Chi² = 2.81, df = 1 (P = 0.09), I² = 64.4%**	1.04 (0.89 to 1.23) 2 trials, n=390. I2=0%	0.85 (0.71 to 1.01) 2 trials, n=354 I2=0%	**Chi² = 2.81, df = 1 (P = 0.09), I² = 64.4%**	0.91 (0.65 to 1.28) 1 trial, n=150. I2=NA	0.94 (0.83 to 1.06) 5 trials, n=860 I2=66%	**Chi² = 0.04, df = 1 (P = 0.85), I² = 0%**	0.85 (0.71 to 1.01) 2 trials, n=354. I2= 0%	1.04 (0.89 to 1.23) 4 trials, n=710 I2=0%	**Chi² = 2.81, df = 1 (P = 0.09), I² = 64.4%**
**ICU LOS MD (95% CI)**	-0.39 (-0.71 to -0.06), 3 trials, n=469. I2=51%	-0.24 (-0.37 to -0.10), 5 trials, n=582. I2=82%	**Chi² = 0.70, df = 1 (P = 0.4), I² = 0%**	-0.19 (-0.35 to -0.03) 5 trials, n=618, I2=84%	-0.36 (-0.55 to -0.17) 3 trials, n=433, I2=0%	**Chi² = 1.87, df = 1 (P = 0.17), I² = 46.5%**	-0.37 (-0.59 to -0.15), 1 trial, n=204 I2= NA	-0.21 (-0.50 to 0.09) 6 trials, n=747 I2=81%	**Chi² = 0.77, df = 1 (P = 0.38), I² = 0%**	-0.36 (-0.57 to -0.14) 2 trials, n=354, I2=0%	-0.21 (-0.36 to -0.06), 6 trials, n=697, I2=81%	**Chi² = 1.18 df = 1 (P = 0.28), I² = 14.9%**
**HOSP LOS MD (95% CI)**	-056 (-1.08 to -0.04), 2 trials, n=279. I2=0%	-0.46 (-0.77 to -0.15), 4 trials, n=482, I2=0%	**Chi² = 0.1, df = 1 (P = 0.75), I² = 0%**	-0.43 (-1.55 to 0.69) 3 trials, n=328. I2=0%	-0.49 (-0.77 to -0.22) 3 trials, n=433 I2=0%	**Chi² = 0.01, df = 1 (P = 0.91), I² = 0%**	-0.50 (-0.82 to -0.18) 1 trial, n=204 I2= NA	-0.46 (0.05 to 0.93) 5 trials, n=557 I2=0%	**Chi² = 0.02, df = 1 (P = 0.89), I² = 0%**	-0.48 (-0.80 to -0.17), 2 trials n=354, I2=0%	-0.50 (-1.00 to -0.00) 4 trials, n=407 I2=0%	**Chi² = 0.00, df = 1 (P = 0.95), I² = 0%**

RR: Risk ratio, MD: Mean Difference, 95%CI: 95% confidence intervals, MI: Myocardial Infarction, AKI: Acute Kidney Injury, ICU: Intensive care unit, Hosp Hospital, LOS: Length of stay, CABG: coronary artery bypass grafting.

For peer review only

Figure. S1 PRISMA Flowchart of included studies

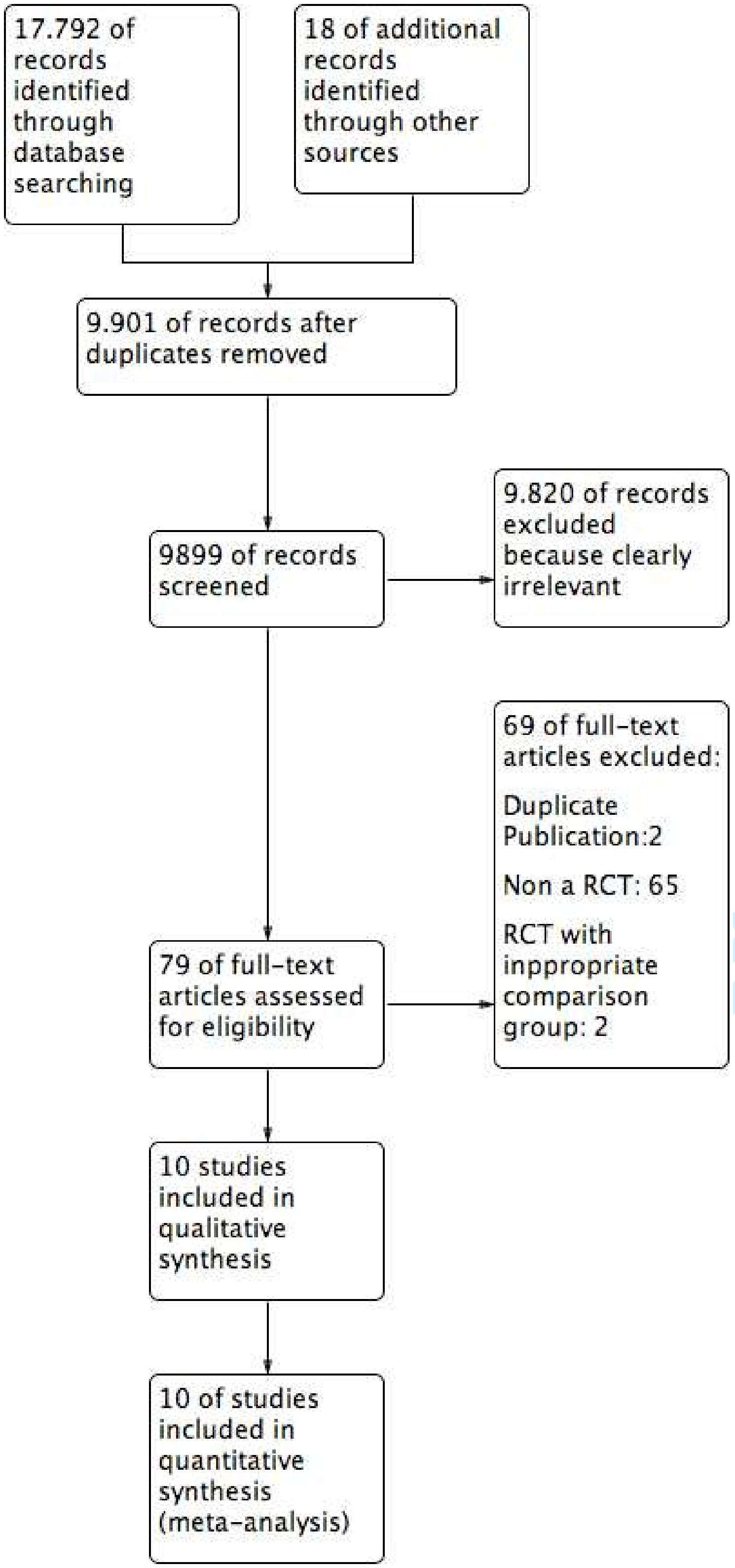

Figure S2: Effects of using NIRS on Bleeding, Transfusion and Time to discharge from ICU or Hospital.

RBC Transfusion

Study or Subgroup	NIRS Events	NIRS Total	Control Events	Control Total	Weight	Risk Ratio M-H, Random, 95% CI
Colak 2014	77	94	73	96	41.0%	1.08 [0.93, 1.25]
Murkin, 2007	8	100	10	100	4.0%	0.80 [0.33, 1.94]
PASPORT 2017	37	98	44	106	19.3%	0.91 [0.65, 1.28]
Vretzakis 2013	51	75	63	75	35.7%	0.81 [0.67, 0.97]
**Total (95% CI)**		**367**		**377**	**100.0%**	**0.93 [0.77, 1.12]**
Total events	173		190			

Heterogeneity: Tau² = 0.02; Chi² = 6.07, df = 3 (P = 0.11); I² = 51%
Test for overall effect: Z = 0.77 (P = 0.44)

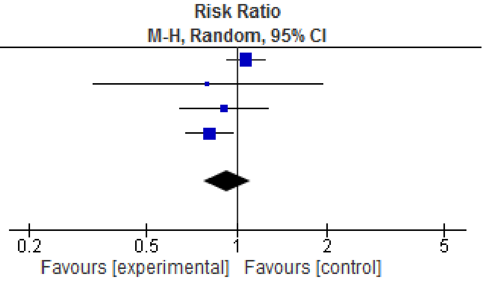

Reoperation for bleeding

Study or Subgroup	NIRS Events	NIRS Total	Control Events	Control Total	Weight	Risk Ratio M-H, Random, 95% CI
Colak 2014	0	94	1	96	9.7%	0.34 [0.01, 8.25]
Murkin, 2007	1	100	1	100	12.9%	1.00 [0.06, 15.77]
PASPORT 2017	6	98	4	106	64.4%	1.62 [0.47, 5.58]
Vretzakis 2013	1	75	1	75	13.0%	1.00 [0.06, 15.69]
**Total (95% CI)**		**367**		**377**	**100.0%**	**1.23 [0.46, 3.32]**
Total events	8		7			

Heterogeneity: Tau² = 0.00; Chi² = 0.86, df = 3 (P = 0.83); I² = 0%
Test for overall effect: Z = 0.41 (P = 0.68)

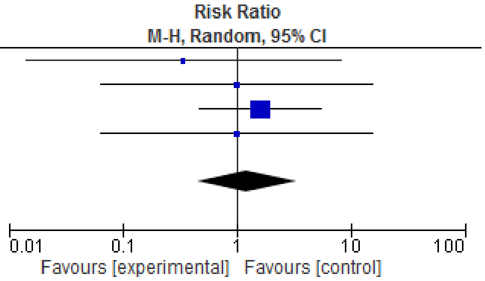

Intensive care Unit Length of Stay

Study or Subgroup	NIRS Mean	NIRS SD	NIRS Total	Control Mean	Control SD	Control Total	Weight	Mean Difference IV, Random, 95% CI
Colak 2014	2.7	6.2	94	1.9	0.9	96	5.6%	0.80 [-0.47, 2.07]
Deschamps 2013	2.99	2.26	23	0.39	2.05	25	5.9%	2.60 [1.38, 3.82]
Deschamps 2016	2.65	2.42	34	3.35	3.61	46	5.3%	-0.70 [-2.02, 0.62]
Kara, 2015	1.74	0.81	43	2.12	1.05	36	17.6%	-0.38 [-0.80, 0.04]
Mohandas 2013	1.49	0.38	50	1.7	0.49	50	22.6%	-0.21 [-0.38, -0.04]
Murkin, 2007	1.25	0.84	100	1.87	2.67	100	14.9%	-0.62 [-1.17, -0.07]
PASPORT 2017	3.3	0.81	98	3.67	0.79	106	21.8%	-0.37 [-0.59, -0.15]
Vretzakis 2013	2.7	3.8	75	2.7	3.6	75	6.2%	0.00 [-1.18, 1.18]
**Total (95% CI)**			**517**			**534**	**100.0%**	**-0.13 [-0.47, 0.22]**

Heterogeneity: Tau² = 0.13; Chi² = 27.52, df = 7 (P = 0.0003); I² = 75%
Test for overall effect: Z = 0.71 (P = 0.48)

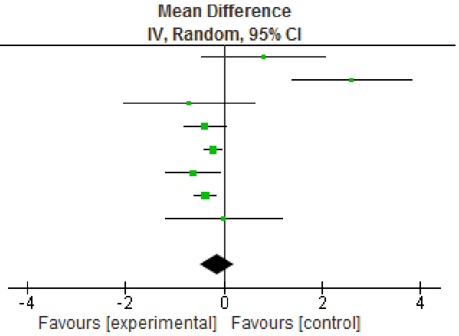

Hospital Length of Stay

Study or Subgroup	NIRS Mean	NIRS SD	NIRS Total	Control Mean	Control SD	Control Total	Weight	Mean Difference IV, Random, 95% CI
Deschamps 2013	7.6	5.4	23	7.9	3.2	25	3.2%	-0.30 [-2.84, 2.24]
Deschamps 2016	11	7.2	34	9.9	5.8	46	2.4%	1.10 [-1.84, 4.04]
Kara, 2015	7.15	1.39	43	7.67	1.14	36	66.3%	-0.52 [-1.08, 0.04]
Murkin, 2007	6.1	4.4	100	6.9	5.5	100	10.8%	-0.80 [-2.18, 0.58]
Pasport 2015	8.5	4.2	98	8.9	4.6	106	14.1%	-0.40 [-1.61, 0.81]
Vretzakis 2013	10.9	3.6	75	10.2	10.7	75	3.2%	0.70 [-1.85, 3.25]
**Total (95% CI)**			**373**			**388**	**100.0%**	**-0.45 [-0.90, 0.01]**

Heterogeneity: Tau² = 0.00; Chi² = 2.17, df = 5 (P = 0.83); I² = 0%
Test for overall effect: Z = 1.94 (P = 0.05)

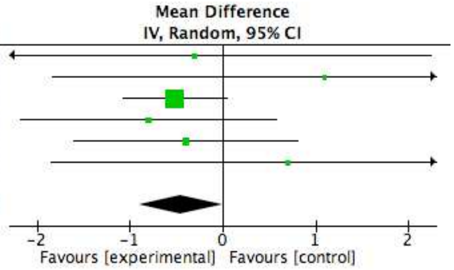

PROTOCOL INFORMATION

Protocol title: Efficacy of Near-Infrared Spectroscopy on the outcome of patients undergoing cardiac surgery: a systematic review.

For peer review only

Authors

**Prof Gavin J. Murphy**<br>British Heart Foundation<br>Professor of Cardiac Surgery<br>Department of Cardiovascular Sciences<br>University of Leicester<br>Glenfield Hospital Groby Road Leicester,<br>LE3 9QP Tel: 0116 258 3054<br>Email: gjm19@le.ac.uk	**Dr Giovanni Mariscalco**<br>Senior Lecturer<br>Department of Cardiovascular Sciences<br>University of Leicester<br>Glenfield Hospital Groby Road Leicester,<br>LE3 9QP Tel: 0116 258 3019<br>Email: gm247@leicester.ac.uk
**Dr Giuseppe Filiberto Serraino**<br>Clinical Research Fellow<br>Department of Cardiovascular Sciences University of Leicester Glenfield Hospital Groby Road Leicester,<br>LE3 9QP Email: gfs3@le.ac.uk	

Contact person: Professor Gavin J Murphy, British Heart Foundation Professor of Cardiac Surgery University of Leicester, Glenfield Hospital Groby Road Leicester, LE3 9QP. Tel: 0116 258 3054, Email: gjm19@le.ac.uk

Funding sources/sponsor

Leicester Cardiovascular Biomedical Research Unit

Dates

Anticipated or actual start date ---

Anticipated completion date ----

Type of review

Epidemiologic; Intervention

Language

English

Country

United Kingdom

Keywords

Systematic review; Cardiac Surgery; Near Infra-Red Spectroscopy; Near Infrared Oximetry; Cognitive dysfunction.

For peer review only

ABSTRACT

Background: Organ injury attributable to cardiopulmonarybypass is a major cause of morbidity, mortality and resource use in cardiac surgery patients. Regional tissue hypoxia during CPB has been implicated in perioperative organ injury. It has been suggested that goal directed patient specific optimisation of regional (brain) tissue hypoxia measured using Near Infra-Red Specroscopy (NIRS) may reduce the frequency of brain injury. Other investigators have suggested that cerebral NIRS based algorithsm may have utility for the protection of other organs, or a part of a restricitive red cell transfusion protocol.

Objectives: Our primary objective is to establish whether the use of perioperative goal directed interventions that aim to optimise cerebral Near Infrared Spectroscopy readings result in reductions in measures of cerebral injury (neurocognitive function, serum biomarkers), injury to other organs including the heart and brain, transfusion rates, mortality and resource use.

Search methods: We will conduct the search in October 2015. Potentially eligible trials will be identified by searching the Cochrane Central Register of Controlled Trials, MEDLINE , and EMBASE, using a combination of subject headings and text words to identify relevant trials. The Medline search strategy below will be adapted as appropriate for other databases.

((Cardiopulmonary Bypass) OR (Cardiac Surgery) OR (Coronary Artery Bypass) OR (Extra Corporeal Circulation)OR(Perioperative Morbidity)) AND ((Near Infra-Red Spectroscopy) OR (Oximetry) OR (Brain/Metabolism)OR(Cerebral Desaturation)OR(Cerebral Perfusion)OR(Cerebral Ischemia)OR(Cerebral Oximetry)OR(Cerebral Saturation)OR(Near Infrared Oximetry)(Cognitive)).

Selection Criteria: Two review authors will independently select references for further assessment by going through all titles and abstracts. Further selection will be based on review of full-text articles for selected references.

Data Collection and Analysis: Two review authors will independently extract study data. We will calculated the risk ratio (RR) with 95% confidence interval (CI) for binary outcomes and the mean difference (MD) or the standardised mean difference (SMD) with 95% CI for continuous outcomes. We will perform meta-analysis when possible, when I^2 is less than or equal to 80% using a fixed-effect or random-effects model, using Review Manager software. The range of point estimates for individual studies will be presented when

For peer review only

I^2 > 80%. Heterogenity will be explored using subgroup analyses. Sensitivity analyses will explore the robustness of our primary analysis to exclusion of studies at high risk of bias.

Summary: We will use GRADEpro software to prepare the ’Summary of findings’table. We will judge the overall quality of the evidence foreach outcome as ‘high,’ ‘moderate,’ ‘low’ or ‘very low’ accordingto the GRADE (Grades of Recommendation, Assessment, Developmentand Evaluation) approach.

BACKGROUND

Organ injury is a common and severe complication of cardiac surgery with cardiopulmonary bypass (CPB), where it contributes to mortality, morbidity and the increased use of hospital resources [1]. In a recent trial where data on organ dysfunction was prospectively collected clinically significant kidney, lung and myocardial injury occurred in 34%, 16% an 11% of all patients and contributed to 41%, 36% and 24% of all deaths respectively [2]. The pathophysiology of CPB associated morbidity is multifactorial but is thought to involve regional hypoperfusion and tissue hypoxia [3, 4], often within vascular beds which are already abnormal due to advanced age or comorbidities such as diabetes [5, 6]. Adequate tissue oxygen delivery during CPB is achieved by optimisation of several parameters including CPB pump flows, perfusion pressure, haematocrit and the oxygen saturation of arterial blood. In contemporary clinical practice in adult cardiac surgery, the adequacy of perfusion is determined by the use of global measures of oxygen utilisation such as the mixed venous oxygen saturation (SvO2) or evidence of tissue hypoxia as implied by elevated serum lactate, a marker of cell anaerobic metabolism, or other indicators of metabolic acidosis [7]. However, in some patients, particularly those with pre-existing end organ dysfunction, global measures may not detect regional hypoxia and it has been suggested that measures of regional or tissue specific measures of oxygenation may have greater clinical utility [8-11].

The Intervention

Direct measures of regional tissue oxygen levels such as gastric tonometry, laser Doppler flowmetry of the intestinal mucosa, or cerebral venous oxygen saturation using jugular bulb catheters, can be used to

For peer review only

measure and optimise tissue oxygenation but these modalities are invasive, which limits their use. More recently, Near Infra-red Spectroscopy (NIRS) has emerged as a technique to monitor regional tissue oxygenation non-invasively and accurately with the added benefit that it can accurately measure tissue oxygenation in one of the most important end organs, the brain. NIRS sensors, when applied to the forehead, can determine the relative saturation / desaturation of blood within the cerebral arterioles and venules of the forebrain. Sas the total volume of blood is domintaed by th enemous compartment this measure is considered to accurately reflect cerebral venous oxygen saturation [12, 13] and is now approved as a non-invasive measure of regional cerebral oxygenation by the Medicines and Healthcare Products Regulatory Agency (MHRA). Early clinical studies suggested that the use of this technoliogy to optimise cerebral oxygenation during CPB have shown that optimisation of cerebral oxygenation using NIRS can be associated not only with reduced neurological morbidity but also with a reduction in renal complications and other major adverse clinical events [14-16]. However in the absence of high quality evidence there is uncertainty as to the efficacy or cost effectiveness of this device and there is wide variation in its use [ ].

Indications for red blood cell transfusion during cardiac surgery

Cerebral oximetry may also be used during CPB to develop goal-directed, patient-specific indicators of the need for red blood cell (RBC) transfusion if used as part of a wider algorithm designed to optimise cerebral oxygenation. The primary goal of RBC transfusion is to optimise tissue oxygenation. Currently, most RBC transfusions are given solely as a response to a haematocrit that has fallen below an arbitrary threshold and not as a response to incipient tissue hypoxia [18, 19]. The haematocrit is a poor indicator of regional tissue hypoxia and, therefore, is inadequate as an indicator of the need for transfusion.The haematocrit below which oxygen delivery to tissues is reduced such that anaerobic metabolism occurs is known as the critical Haematocrit (Hcrit). In healthy human adults very low haematocrits of less than 15 can cause organ hypoxia [9, 11] and transfusion in such cases is thought to be beneficial [20, 21]. The Hcrit for patients during cardiac surgery, however, is unclear. It is thought to be higher than 15, as most patients are elderly and have comorbid conditions, but it is also likely that the Hcrit varies considerably both between cardiac

For peer review only

patients as well as for individual patients over the course of the perioperative period [22]. Hcrit is increased by conditions which impair autoregulation such as diabetes or increased age [5, 6] and during CPB, Hcrit is also affected by multiple factors that affect the balance of oxygen supply and oxygen demand such as hypothermia, re-warming, pump flow or perfusion pressure [23]. Uncertainty about when an individual may benefit from a transfusion is reflected in the wide range in reported haematocrit transfusion thresholds used during CPB (from 17-25) [24-26] as well as the wide variation in transfusion rates in cardiac surgical patients across units in the United Kingdom (UK) (35-75%) [27]. Because the Hcrit for any particular patient at any given time is unknown, the ability to directly measure tissue oxygenation and therefore incipient tissue hypoxia may have distinct advantages over the use of generic and pre-specified haematocrit transfusion thresholds.

How the intervention might work

Existing protocols to optimise tissue oxygenation during CPB deploy manipulations of CPB (according to levels of blood markers such as lactate or pH) and the transfusion of RBC (according to the haematocrit). These methods are often treated independently of each other, and in the case of trandsfusion commonly guided by protocolised thresholds, despite the fact that both have the underlying aim of improving tissue oxygenation [7].

In contrast the patient-specific algorithm differs from the standard care in two key ways. First it is 'goal-directed', in that the algorithm is specifically targeted to maintain cerebral oxygen delivery during CPB (monitored by NIRS), a measure of regional rather than global tissue hypoxia. Second it allows the manipulation of multiple variables to optimise regional oxygenation for individual patients. This represents a form of personalised medicine. The most widely cited patient-specific algorithm developed by Murkin and colleagues [28] aims to optimise the cerebral oxygen supply / demand balance during CPB by: (a) increasing oxygen supply using hyperoxygenation, increased pump flow, perfusion pressure or hypercapnic cerebral vasodilation, (b) Increasing oxygen offloading by the use of nitrates, or (c) reducing oxygen demand by deepening anaesthesia. Cerebral oxygen saturations approaching a low threshold in the presence of

For peer review only

anaemia (a haematocrit of between 18 and 23) and despite optimisation of other parameters suggest that the cerebral Hcrit is about to be reached and transfusion is indicated. Therefore, this algorithm is patient and time specific and goal-directed to optimise a validated objective measure of tissue oxygenation. This should potentially reduce healthcare costs associated with complications attributable to tissue hypoxia during CPB or to unnecessary allogeneic RBC transfusions.

Why is it important to do this review

Numerous studies have reported the incidence of postoperative neurocognitive decline around 50% [29, 30]. Central venous oxygen desauration is common following cardiac surgery and this has been implicated in cognitive decline [3, 4]. Multiple studies have evaluated the efficacy of NIRS based algorithms that allow optimisation of cerebral saturation. Although some of these studies have been reported as positive [8] overall these results of these studies have been have been inconclusive, and no recent systematic review has attempted to summarise the available evidence. The use of this technology is variable and there is evidence of equipoise as to its effectiveness. To resolve this uncertainty we propose to undertake a systematic review and meta-analysis of the available evidence from RCTs.

OBJECTIVES

(1) To establish whether the use of perioperative goal directed interventions that aim to optimise cerebral Near Infrared Spectroscopy readings result in reductions in measures of cerebral injury (neuroscogitive function, serum biomarkers), morbidity, mortality and resource use.

(2) Does the use of perioperative goal directed interventions that aim to optimise cerebral Near Infrared Spectroscopy readings result in reductions in red cell transfusion, non red cell transfusion, or bleeding.

METHODS

Types of Studies

For peer review only

Randomized controlled trials irrespective of blinding, language, publication status, date of publication and sample size.

Types of Participants

Patients undergoing cardiac surgery for acquired or congenital disease, or aortovascular disease with or without cardiopulmonary bypass. No age restriction will be applied. There are no exclusion criteria.

Types of Interventions

Intervention: Goal directed NIRS algorithm.

Comparator /control: An untread group, or alternative (non NIRS based) goal directed therapy.

Types of Outcome Measure

Primary outcomes:

Mortality: 30 day or hospital all-cause mortality.

Secondary outcomes:

11. Acute brain injury; stroke, TIA as defined by study authors.
12. Low cardiac output as defined by study authors.
13. Myocardial Infarction as defined by study authors.
14. Acute kidney Injury Stage 3 [32] or requiring haemofiltration as defined by study authors.
15. Neurocognitive function; group means as described by neurocognitive tests. Tests recommended by a consensus statement to test all key domains of cognitive function [33] are marked with *. Studies will be categorised as Yes/ No as to whether they have assessed the key domains described in the Consensus Statement. Key domians [ ] that may be assessed are as follows
    - Attention: Sustained and divided attention: Consensus statements reccomend the Trail-Making Test parts A* and B* [35, 36].
    - Verbal memory: Consensus statements reccomend the RAVLT and Rey Auditory Verbal Learning Test* [36]
    - Visuo-spatial: as the Block Design from the Wechsler Adult Intelligence Scale [37]
    - Psychomotor speed: Consensus statements reccomend tests such the Digit Symbol Test from the Wechsler Adult Intelligence Scale [37]
    - Executive function/Verbal fluency: Consensus statements reccomend tests such the Controlled Oral Word Association Test [38]

For peer review only

- Motor coordination: Consensus statements reccomend tests such the Grooved Pegboard Test*, dominant and non-dominant hand [33].

16. Neurocognitive dysfunction, as a dichotomous outcome reported by the study authors; A consensus definition is a change in a single test of >1 SD. This may be defined as change in a group mean (adjusted for baseline) or for individual patients [39]. Studies will be categorised as defining cognitive dysfunction using a Consensus versus a non-Consensus definitions.
17. Risk of receiving blood transfusion as defined by study authors.
18. Reoperation for bleeding as defined by study authors.
19. Resoiurce Use: ICU and hospital LOS as defined by study authors.
20. S100B levels as reported by study authors.

Search methods for identification of studies

Potentially eligible trials will be identified by searching the Cochrane Central Register of Controlled Trials, MEDLINE , and EMBASE, using a combination of subject headings and text words to identify relevant trials. The Medline search strategy below will be adapted as appropriate for other databases.

((Cardiopulmonary Bypass) OR (Cardiac Surgery) OR (Coronary Artery Bypass) OR (Extra Corporeal Circulation)OR(Perioperative Morbidity)) AND ((Near Infra-Red Spectroscopy) OR (Oximetry) OR (Brain/Metabolism)OR(Cerebral Desaturation)OR(Cerebral Perfusion)OR(Cerebral Ischemia)OR(Cerebral Oximetry)OR(Cerebral Saturation)OR(Near Infrared Oximetry)(Cognitive)).

To identify ongoing or unpublished trials we will also search the Clinicaltrials.gov using the following search terms:

Search terms: Randomized

Study Type: Interventional Studies

Conditions: Cardiac surgery OR Cardiopulmonary bypass

Interventions: Near Infra-Red Spectroscopy OR Near Infrared Oximetry OR Cerebral Desauturation

We will also examined the reference lists of eligible trials and reviews. Searches will not be restricted by language or publication status.

Data Collection and Analysis

The review will be performed in accordance with instructions given in the Cochrane Handbook for Systematic Reviews of Interventions (Higgins 2011).

Selection of Studies

For peer review only

Two reviewers GJM, GFS will identify trials for inclusion independently of each other. Exluded studies and the reason for exclusion will be recorded.

Data extraction (selection and coding)

Two authors will independently screen the search output to identify records of potentially eligible trials examining the outcomes, the full texts of which will be retrieved and assessed for inclusion.

A standardised form will be used to extract data from the included studies for assessment of study quality and evidence synthesis. Extracted information will include:

- Year and language of publication
- Country of Particiapnt recruitment
- Year of conduct of the trial
- Study setting; university teaching hospital, non universityteaching hospital
- Study population; inclusion and exlusion criteria
- Sample size
- Participant demographics
- Baseline characteristics
- Type of surgery
- Details of NIRS algorithm (Murkin, non Murkin [41])
- Deatails of Comparator; non NIRS goal directed therapy, standard care (protocolised care)
- Outcomes and times of measurement
- Information for assessment of the risk of bias.

Two review authors will extract data independently, discrepancies will be identified and resolved through discussion (with a third author where necessary). Missing data will be requested from study authors. If there is doubt as to whether trials share participiants completely or partially (with common authors and centres) we will contact the study authors to ascertain whether the studt report has been duplicated.

Risk of Bias

The following bias risk domains will be assessed as Low, Uncertain, or High based on the instructions given in the in the Cochrane Handbook for Systematic Reviews of Interventions [42];

- Sequence Generation
- Allocation Concealment
- Blinding of participant, personnel
- Blinding of outcome Assessors
- Incomplete outcome data
- Selective outcome reporting
- Source of funding bias

Trials will be classified as having a low risk of bias if they are graded as being at low risk of bias in all of these domains.

For peer review only

Two review authors will independently assess the risk of bias in all of the studies. Discrepancies will be resolved by discussion with a third author.

Assessment of Reporting Bias

Publication bias will be assessed by the visual assessment of funnel plots and Eggers test [44].

Measures of treatment Effect

For dichotomous variables, we will calculate the risk ratio (RR) with 95% confidence interval (CI). For continuous variables, we will calculate the mean difference (MD) with 95% CI for outcomes such as hospital stay, and standardised mean difference (SMD) with 95% CI for quality of life (when different scales were used).

Dealing with missing data

We will perform an intention-to-treat analysis where possible. For dichotomous data presented only as percentages we will estimate frequencies using reported sample sizes for this outcome. For continuous outcomes if the mean and the standard deviation were not available from the trial report, we will seek this information from the trial authors. If this information is still not available, we will calculate the mean and standard deviation from median (interquartile ranges) using the software available in Review Manager Version 5.

Assessment of Heterogeneity

We anticipate that major sources of clinical heterogeneity will be associated with different patient groups (adults, children, congenital versus acquired disease), the use of different goal directed NIRS algorithms, the use of co-interventions such as restrictive transfusion thresholds, and differences in the methodology used to assess neurocognitive dysfunction. We will explore heterogeneity within each meta-analysis using a Chi^2 test with significance set at a P value of 0.10, and we expressed the percentage of heterogeneity due to variation rather than to chance as I^2 [45].We defined heterogeneity as follows:

I^2 0-40%: no or mild heterogeneity

I^2 40-80%: moderate heterogeneity

I^2 > 80%: sever heterogeneity

For peer review only

In the presence of severe heterogeneity meta-analysis will not be performed.

Data Synthesis

Meta-analyses will be performed using the software package Review Manager version 5.2 and in accordance with the recommendations of the *Cochrane Handbook for Systematic Reviews of Intervention* [46].

For the primary analysis we will compare the results of a random-effects model versus a fixed effects model to assess the effects of small studies. For continuous outcomes, we will pool mean differences or standardised mean differences by using the inverse variance method. We will use the generic inverse variance method to pool hazard ratios.

Subgroup Analyses

Subgroup analyses will be performed in trials in which the Murkin algorithm [41] was used to guide goal directed therapy versus those that did not, and by participant group; CABG versus non CABG, adults versus children, assessment of neurocognitive function that incorporates test described in a previous consensus statement, and studies that combined the NIRS algorithm with a restrictive red cell transfusion trigger. Test for sub-group differences with Review Manager will be used with a P value of <0.05 considered statistically significant.

Sensitivity Analyses

Sensitivity analysis will exclude trials with unclear or high risk of bias for random sequence generation; unclear or high risk of bias due to lack of blinding of participants, healthcare providers or outcome assessors, and unclear or high risk of bias due to incomplete outcome data, and unclear of high risk of bias for source of funder.

Summary of findings

We will presentthe main results of the review in a 'Summary of findings' table. We will include the follwing outcomes.

- Risk of mortality
- Risk of stroke, myocardial infarction, or severe acute kidney injury
- Risk of red cell transfusion
- Neurocognitive Impairment

For peer review only

- Resource Use; ICU and hospital length of stay.

We will use GRADEpro software to prepare the ’Summary of findings’table. We will judge the overall quality of the evidence foreach outcome as ‘high,’ ‘moderate,’ ‘low’ or ‘very low’ accordingto the GRADE (Grades of Recommendation, Assessment, Developmentand Evaluation) approach [46].We will consider the following.

- Impact of risk of bias of individual trials.
- Precision of pooled estimate.Inconsistency or heterogeneity (clinical, methodological and statistical).
- Indirectness of evidence.
- Impact of selective reporting and publication bias on effect estimate.

- **COMPETING INTERESTS**

The authors declare that they have no competing interests.

AUTHORS’ CONTRIBUTIONS

___ had full access to all of the data in the study and take responsibility for the integrity of the data and the accuracy of the data analysis.

Study concept and design: ___.

Acquisition of data: ___.

Analysis and interpretation of data: _____.

Drafting of the manuscript: _______.

Statistical analysis: _______

Study supervision: GJM.

For peer review only

For peer review only

REFERENCES

1. Murphy, G J and G D Angelini. Side effects of cardiopulmonary bypass: what is the reality? J Card Surg, 2004. 19(6): p. 481-488.
2. Murphy GJ,
3. Sapire, K J, S P Gopinath, G Farhat, D R Thakar, A Gabrielli, J W Jones, C S Robertson, and B Chance. Cerebral oxygenation during warming after cardiopulmonary bypass. Crit Care Med, 1997. 25(10): p. 1655-1662.
4. Yao, F S, C C Tseng, C Y Ho, S K Levin, and P Illner. Cerebral oxygen desaturation is associated with early postoperative neuropsychological dysfunction in patients undergoing cardiac surgery. J Cardiothorac Vasc Anesth, 2004. 18(5): p. 552-558.
5. Croughwell, N, M Lyth, T J Quill, M Newman, W J Greeley, L R Smith, and J G Reves. Diabetic patients have abnormal cerebral autoregulation during cardiopulmonary bypass. Circulation, 1990. 82(5 Suppl): p. IV407-412.
6. Miyoshi, S, T Morita, Y Kadoi, and F Goto. Analysis of the factors related to a decrease in jugular venous oxygen saturation in patients with diabetes mellitus during normothermic cardiopulmonary bypass. Surg Today, 2005. 35(7): p. 530-534.
7. Monitoring during CPB
8. Haisjackl, M, G Luz, H Sparr, R Germann, N Salak, B Friesenecker, E Deusch, S Meusburger, and W Hasibeder. The effects of progressive anemia on jejunal mucosal and serosal tissue oxygenation in pigs. Anesth Analg, 1997. 84(3): p. 538-544.
9. Leung, J M, R B Weiskopf, J Feiner, H W Hopf, S Kelley, M Viele, J Lieberman, J Watson, M Noorani, D Pastor, H Yeap, R Ho, and P Toy. Electrocardiographic ST-segment changes during acute, severe isovolemic hemodilution in humans. Anesthesiology, 2000. 93(4): p. 1004-1010.
10. Tsai, A G, P Cabrales, and M Intaglietta. Microvascular perfusion upon exchange transfusion with stored red blood cells in normovolemic anemic conditions. Transfusion, 2004. 44(11): p. 1626-1634.
11. Weiskopf, R B, J H Kramer, M Viele, M Neumann, J R Feiner, J J Watson, H W Hopf, and P Toy. Acute severe isovolemic anemia impairs cognitive function and memory in humans. Anesthesiology, 2000. 92(6): p. 1646-1652.
12. Kim, M B, D S Ward, C R Cartwright, J Kolano, S Chlebowski, and L C Henson. Estimation of jugular venous O2 saturation from cerebral oximetry or arterial O2 saturation during isocapnic hypoxia. J Clin Monit Comput, 2000. 16(3): p. 191-199.
13. Pollard, V, D S Prough, A E DeMelo, D J Deyo, T Uchida, and H F Stoddart. Validation in volunteers of a near-infrared spectroscope for monitoring brain oxygenation in vivo. Anesth Analg, 1996. 82(2): p. 269-277.
14. Alexander J.C.Jr, K M A, & Dance G.R. Reduced postoperative length of stay may result from using cerebral oximetry monitoring to guide treatment. The Annals of Thoracic Surgery, 2002. 73(1): p. S373.
15. Goldman, S, F Sutter, F Ferdinand, and C Trace. Optimizing intraoperative cerebral oxygen delivery using noninvasive cerebral oximetry decreases the incidence of stroke for cardiac surgical patients. Heart Surg Forum, 2004. 7(5): p. E376-381.
16. Murkin, J M, S J Adams, R J Novick, M Quantz, D Bainbridge, I Iglesias, A Cleland, B Schaefer, B Irwin, and S Fox. Monitoring brain oxygen saturation during coronary bypass surgery: a randomized, prospective study. Anesth Analg, 2007. 104(1): p. 51-58.
17. Variability Paper
18. Vincent, J L, J F Baron, K Reinhart, L Gattinoni, L Thijs, A Webb, A Meier-Hellmann, G Nollet, D Peres-Bota, and A B C Investigators. Anemia and blood transfusion in critically ill patients. JAMA, 2002. 288(12): p. 1499-1507.
19. Walsh, T S, M Garrioch, C Maciver, R J Lee, F MacKirdy, D B McClelland, J Kinsella, C Wallis, and G Audit of Transfusion in Intensive Care in Scotland Study. Red cell requirements for intensive care units adhering to evidence-based transfusion guidelines. Transfusion, 2004. 44(10): p. 1405-1411.

For peer review only

20. Casutt, M, B Seifert, T Pasch, E R Schmid, M I Turina, and D R Spahn. Factors influencing the individual effects of blood transfusions on oxygen delivery and oxygen consumption. Crit Care Med, 1999. 27(10): p. 2194-2200.
21. Weiskopf, R B, J Feiner, H Hopf, J Lieberman, H E Finlay, C Quah, J H Kramer, A Bostrom, and P Toy. Fresh blood and aged stored blood are equally efficacious in immediately reversing anemia-induced brain oxygenation deficits in humans. Anesthesiology, 2006. 104(5): p. 911-920.
22. Utoh, J, S Moriyama, K Okamoto, R Kunitomo, M Hara, and N Kitamura. The effects of cardiopulmonary bypass on postoperative oxygen metabolism. Surg Today, 1999. 29(1): p. 28-33.
23. Shann KG, Likosky DS, Murkin JM, Baker RA, Baribeau YR, DeFoe GR, Dickinson TA, Gardner TJ, Grocott HP, O'Connor GT, Rosinski DJ, Sellke FW, Willcox TW. An evidence-based review of the practice of cardiopulmonary bypass in adults: a focus on neurologic injury, glycemic control, hemodilution, and the inflammatory response. J Thorac Cardiovasc Surg. 2006 Aug;132(2):283-90.
24. DeFoe, G R, C S Ross, E M Olmstead, S D Surgenor, M P Fillinger, R C Groom, R J Forest, J W Pieroni, C S Warren, M E Bogosian, C F Krumholz, C Clark, R A Clough, P W Weldner, S J Lahey, B J Leavitt, C A Marrin, D C Charlesworth, P Marshall, and G T O'Connor. Lowest hematocrit on bypass and adverse outcomes associated with coronary artery bypass grafting. Northern New England Cardiovascular Disease Study Group. Ann Thorac Surg, 2001. 71(3): p. 769-776.
25. Fang, W C, R E Helm, K H Krieger, T K Rosengart, W J DuBois, C Sason, M L Lesser, O W Isom, and J P Gold. Impact of minimum hematocrit during cardiopulmonary bypass on mortality in patients undergoing coronary artery surgery. Circulation, 1997. 96(9 Suppl): p. II-194-199.
26. Von Heymann, C, M Sander, A Foer, A Heinemann, B Spiess, J Braun, M Kramer, J Grosse, P Dohmen, S Dushe, J Halle, W F Konertz, K D Wernecke, and C Spies. The impact of an hematocrit of 20% during normothermic cardiopulmonary bypass for elective low risk coronary artery bypass graft surgery on oxygen delivery and clinical outcome--a randomized controlled study. Crit Care, 2006. 10(2): p. R58.
27. Murpjhy GJ Audit
28. Denault A, Deschamps A, Murkin JM. A proposed algorithm for the intraoperative use of cerebral near-infrared spectroscopy. Semin Cardiothorac Vasc Anesth. 2007 Dec;11(4):274-81
29. Newman MF, Kirchner JL, Phillips-Bute, et al. Longitudinal assessment of neurocognitive function after coronary artery bypass surgery. New Engl J Med 2001;344:395–402.
30. Selnes OA, Goldsborough MA, Borowicz LM Jr, Enger C, Quaskey SA, McKhann GM. Determinants of cognitive change after coronary artery bypass graft surgery: a multi- factorial problem. Ann Thorac Surg 1999;67:1669 –76.
31. NIRS AKI
32. Mehta, R L, J A Kellum, S V Shah, B A Molitoris, C Ronco, D G Warnock, A Levin, and N Acute Kidney Injury. Acute Kidney Injury Network: report of an initiative to improve outcomes in acute kidney injury. Crit Care, 2007. 11(2): p. R31.
33. Murkin, J M, S P Newman, D A Stump, and J A Blumenthal. Statement of consensus on assessment of neurobehavioral outcomes after cardiac surgery. Ann Thorac Surg, 1995. 59(5): p. 1289-1295.
34. Mahanna, E P, J A Blumenthal, W D White, N D Croughwell, C P Clancy, L R Smith, and M F Newman. Defining neuropsychological dysfunction after coronary artery bypass grafting. Ann Thorac Surg, 1996. 61(5): p. 1342-1347.
35. SP Newman. Analysis and interpretation of neuropsychologic tests in cardiac surgery Ann Thorac Surg, 59 (1995), pp. 1351–1355
36. Spreen O., S, E. A compendium of neuropsychological tests. 1998, New York: Oxford University Press
37. Wechsler, D. Manual of the Wechsler Test of Adult Reading (WTAR-UK). 2001, New York Psychological Corporation (Iglesias)
38. Benton, A H, R. Multilingual Aphasia Examination. 1976, Iowa City: University of Iow
39. Murkin JM, Stump DA, Blumenthal JA, McKhann G. Defining dysfunction: group means versus incidence analysis-a statement of consensus. Ann Thorac Surg. 1997 Sep;64(3):904-5.
40. Higgins JPT, Deeks JJ. Chapter 7: Selecting studies and collecting data In: Higgins JPT,Green S (editors). Cochrane Handbook for Systematic Reviews of Interventions Version 5.1.0 [updated March 2011]. The Cochrane Collaboration, 2011. www.cochrane-handbook.org.

For peer review only

41. Denault A, Deschamps A, Murkin JM. A proposed algorithm for the intraoperative use of cerebral near-infrared spectroscopy. Semin Cardiothorac Vasc Anesth. 2007 Dec;11(4):274-81
42. Higgins JPT, Altman DG, Sterne JAC. Chapter 8: Assessing risk of bias in included studies. Cochrane Handbook for Systematic Reviews of Interventions Version 5.1.0 [updated March 2011]. The Cochrane Collaboration, 2011. www.cochrane-handbook.org.
43. Sterne JAC, Egger M, Moher D. Chapter 10: Addressing reporting biases In: Higgins JPT, Green S (editors). Cochrane Handbook for Systematic Reviews of Interventions Version 5.1.0 [updated March 2011]. The Cochrane Collaboration, 2011. www.cochranehandbook.org.
44. Higgins JPT, Thompson SG. Quantifying heterogeneity in a meta-analysis. Statistics in Medicine 2002;21(11):1539–58.
45. Higgins JPT, Green S (editors). Cochrane Handbook for Systematic Reviews of Interventions Version 5.1.0 [updated March 2011]. The Cochrane Colloboration, 2011. www.cochrane-handbook.org.
46. Schünemann HJ, Oxman AD, Vist GE, Higgins JPT, Deeks JJ, Glasziou P, et al.Chapter 12: Interpreting results and drawing conclusions. Higgins JPT, Green S(editors). Cochrane Handbook for Systematic Reviewsof Interventions Version 5.1.0 [updated March 2011].The Cochrane Collaboration, 2011. www.cochrane-handbook.org.

For peer review only

PRISMA Checklist:Efficacy of Near-Infrared Spectroscopy on the outcome of patients undergoing cardiac surgery: a systematic review of randomised trials.
Authors: GF Serraino, GJ Murphy

Section/topic	#	Checklist item	Reported on page #
**TITLE**			
Title	1	Identify the report as a systematic review, meta-analysis, or both.	1
Structured summary	2	Provide a structured summary including, as applicable: background; objectives; data sources; study eligibility criteria, participants, and interventions; study appraisal and synthesis methods; results; limitations; conclusions and implications of key findings; systematic review registration number.	2
Rationale	3	Describe the rationale for the review in the context of what is already known.	4
Objectives	4	Provide an explicit statement of questions being addressed with reference to participants, interventions, comparisons, outcomes, and study design (PICOS).	4
Protocol and registration	5	Indicate if a review protocol exists, if and where it can be accessed (e.g., Web address), and, if available, provide registration information including registration number.	4
Eligibility criteria	6	Specify study characteristics (e.g., PICOS, length of follow-up) and report characteristics (e.g., years considered, language, publication status) used as criteria for eligibility, giving rationale.	5
Information sources	7	Describe all information sources (e.g., databases with dates of coverage, contact with study authors to identify additional studies) in the search and date last	4

For peer review only

Section/topic	#	Checklist item	Reported on page #
		searched.	
Search	8	Present full electronic search strategy for at least one database, including any limits used, such that it could be repeated.	4
Study selection	9	State the process for selecting studies (i.e., screening, eligibility, included in systematic review, and, if applicable, included in the meta-analysis).	5
Data collection process	10	Describe method of data extraction from reports (e.g., piloted forms, independently, in duplicate) and any processes for obtaining and confirming data from investigators.	5
Data items	11	List and define all variables for which data were sought (e.g., PICOS, funding sources) and any assumptions and simplifications made.	5
Risk of bias in individual studies	12	Describe methods used for assessing risk of bias of individual studies (including specification of whether this was done at the study or outcome level), and how this information is to be used in any data synthesis.	5
Summary measures	13	State the principal summary measures (e.g., risk ratio, difference in means).	5
Synthesis of results	14	Describe the methods of handling data and combining results of studies, if done, including measures of consistency (e.g., I^2) for each meta-analysis.	5
Risk of bias across studies	15	Specify any assessment of risk of bias that may affect the cumulative evidence (e.g., publication bias, selective reporting within studies).	5
Additional analyses	16	Describe methods of additional analyses (e.g., sensitivity or subgroup analyses, meta-regression), if done, indicating which were pre-specified.	6
Study selection	17	Give numbers of studies screened, assessed for eligibility, and included in the review, with reasons for exclusions at each stage, ideally with a flow diagram.	6

For peer review only

Section/topic	#	Checklist item	Reported on page #
Study characteristics	18	For each study, present characteristics for which data were extracted (e.g., study size, PICOS, follow-up period) and provide the citations.	6
Risk of bias within studies	19	Present data on risk of bias of each study and, if available, any outcome-level assessment (see Item 12).	8
Results of individual studies	20	For all outcomes considered (benefits or harms), present, for each study: (a) simple summary data for each intervention group and (b) effect estimates and confidence intervals, ideally with a forest plot.	9
Synthesis of results	21	Present results of each meta-analysis done, including confidence intervals and measures of consistency.	10
Risk of bias across studies	22	Present results of any assessment of risk of bias across studies (see Item 15).	11
Additional analysis	23	Give results of additional analyses, if done (e.g., sensitivity or subgroup analyses, meta-regression [see Item 16]).	11
Summary of evidence	24	Summarize the main findings including the strength of evidence for each main outcome; consider their relevance to key groups (e.g., health care providers, users, and policy makers).	12
Limitations	25	Discuss limitations at study and outcome level (e.g., risk of bias), and at review level (e.g., incomplete retrieval of identified research, reporting bias).	13
Conclusions	26	Provide a general interpretation of the results in the context of other evidence, and implications for future research.	14
Funding	27	Describe sources of funding for the systematic review and other support (e.g.,	15

For peer review only

Section/topic	#	Checklist item	Reported on page #
		supply of data); role of funders for the systematic review.	

For peer review only

BMJ Open

Effects of cerebral Near-Infrared Spectroscopy on the outcome of patients undergoing cardiac surgery: a systematic review of randomised trials.

Journal:	*BMJ Open*
Manuscript ID	bmjopen-2017-016613.R1
Article Type:	Research
Date Submitted by the Author:	12-May-2017

Complete List of Authors:	Serraino, Giuseppe; University of Leicester College of Medicine Biological Sciences and Psychology, Cardiovascular Sciences<br>Murphy, Gavin; University of Leicester College of Medicine Biological Sciences and Psychology, Cardiovascular Sciences

**Primary Subject Heading**:	Cardiovascular medicine
Secondary Subject Heading:	Surgery, Anaesthesia, Neurology
Keywords:	Cardiopulmonary bypass, cerebral oxygenation, near Infra-red spectroscopy

SCHOLARONE™ Manuscripts

For peer review only

Title: Effects of cerebral Near-Infrared Spectroscopy on the outcome of patients undergoing cardiac surgery: a systematic review of randomised trials.

Authors: Giuseppe F Serraino, Gavin J Murphy

Authors Affiliation: Department of Cardiovascular Sciences and NIHR Leicester Biomedical Research Unit in Cardiovascular Medicine, University of Leicester, Clinical Sciences Wing, Glenfield General Hospital, Leicester, LE3 9QP

Correspondence to: Professor Gavin Murphy, Department of Cardiovascular Sciences and NIHR Leicester Biomedical Research Unit in Cardiovascular Medicine, University of Leicester, Clinical Sciences Wing, Glenfield General Hospital, Leicester, LE3 9QP. Email: gjm19@le.ac.uk Tel: +44 116 2583021, Fax: +44 116 2583054.

Total word count: 4795

Keywords: Cardiopulmonary bypass, cerebral oxygenation, near Infra-red spectroscopy.

For peer review only

For peer review only - http://bmjopen.bmj.com/site/about/guidelines.xhtml

Abstract

Objectives: Goal directed optimisation of cerebral oxygenation using Near Infra-red Spectroscopy (NIRS) during cardiopulmonary bypass is widely used. We tested the hypotheses that the use of NIRS cerebral oximetry results in reductions in cerebral injury (neurocognitive function, serum biomarkers), injury to other organs including the heart and brain, transfusion rates, mortality and resource use.

Design: Systematic review and meta-analysis.

Setting: Tertiary cardiac surgery centres in North America, Europe and Asia.

Participants: A search of Cochrane Central Register of Controlled Trials, Clinical Trials.gov, MEDLINE, and EMBASE and the Cumulative Index to Nursing and Allied Health Literature (CINAHL) Plus from inception to November 2016 identified 10 randomised trials, enrolling a total of 1466 patients, all in adult cardiac surgery.

Interventions: NIRS based algorithms designed to optimise cerebral oxygenation versus standard care (non NIRS based) protocols in cardiac surgery patients during cardiopulmonary bypass.

Outcome Measures: Mortality, organ injury affecting the brain, heart and kidneys, red cell transfusion and resource use.

Results: Two of the 10 trials identified in the literature search were considered at low risk of bias. Random effects meta-analysis demonstrated similar mortality (Risk Ratio (RR) 0.76, 95% Confidence Intervals (CI) 0.30 to 1.96), major morbidity including stroke (RR 1. 08, 95% CI 0.40 to 2.91), red cell transfusion, and resource use, in NIRS treated patients and controls, with little or no heterogeneity. Grades of Recommendation, Assessment, Development and Evaluation (GRADE) assessment of the quality of the evidence was Low or Very Low for all of the outcomes assessed.

Conclusions: The results of this systematic review did not support the hypotheses that cerebral NIRS based algorithms have clinical benefits in cardiac surgery.

Registration: PROSPERO CRD42015027696

For peer review only

For peer review only - http://bmjopen.bmj.com/site/about/guidelines.xhtml

Strengths and limitations of the Study

- We performed a systematic review of randomised trials evaluating the clinical benefits of near infrared spectroscopy based cerebral oximetry monitoring during cardiopulmonary bypass.
- This is most comprehensive review of this technology to date.
- All of the existing trials had important methodological limitations, including importantly a lack of blinding of clinical personnel.
- GRADE assessment of the quality of the evidence was Low or Very Low for all of the outcomes assessed indicating a high likelihood that the findings of the review may be altered by subsequent trials.

For peer review only

For peer review only - http://bmjopen.bmj.com/site/about/guidelines.xhtml

Introduction

Brain injury is a common and severe complication of cardiac surgery with cardiopulmonary bypass (CPB), affecting up to 40% of patients, where it contributes to morbidity, mortality, and the increased use of hospital resources [1]. The pathophysiology of CPB associated brain injury is multifactorial [2] but is thought to involve regional hypoperfusion and tissue hypoxia,[3,4] often within vascular beds which are already abnormal due to advanced age or comorbidities such as diabetes.[5,6] Previous studies have suggested that cerebral oxygenation may be measured non-invasively using Near Infra-red Spectroscopy (NIRS) sensors applied to the forehead,[7] and more importantly that targeted interventions during bypass that increase NIRS measured cerebral oxygenation have clinical benefits including reductions in brain injury,[8] and reductions in injury to other organ systems as a result of improved overall perfusion.[9,10] It has also been suggested that the use of NIRS may allow the safe application of restrictive red cell transfusion thresholds where there is evidence of adequate cerebral oxygenation; a putative personalised transfusion indicator.[7,11] However, there is uncertainty as to the clinical benefits of NIRS[12] and this leads to variability in the use of these devices.[13] To address this uncertainty we performed a systematic review and meta-analysis of randomised trials that had evaluated the effects of personalised NIRS based algorithms on clinical outcomes including mortality, organ (brain, heart, kidney) injury, transfusion and resource use.

Methods

A systematic review was performed using the methods described in Cochrane Handbook for Systematic Reviews of Interventions.[14] The review protocol was registered prospectively; PROSPERO CRD42015027696.

Types of Studies

Randomized controlled trials irrespective of blinding, language, publication status, date of publication and sample size.

For peer review only

For peer review only - http://bmjopen.bmj.com/site/about/guidelines.xhtml

Types of Participants

Patients undergoing cardiac surgery for acquired or congenital disease or aortovascular disease with or without cardiopulmonary bypass were included. No age restriction was applied. There were no exclusion criteria.

Types of Interventions

Intervention: Goal directed NIRS algorithm during cardiac surgery with cardiopulmonary bypass.

Comparator /control: An untreated group, or alternative (non NIRS based) goal directed therapy.

Types of Outcome Measure

Primary outcomes:

Mortality: 30 day or hospital all-cause mortality.

Secondary outcomes:

1. Acute brain injury; stroke, TIA as defined by study authors.
2. Low cardiac output as defined by study authors.
3. Myocardial Infarction as defined by study authors.
4. Acute kidney Injury Stage 3 or requiring hemofiltration as defined by study authors.
5. Neurocognitive function; group means as described by neurocognitive tests. Tests recommended by a consensus statement to test all key domains of cognitive function [15] are marked with *. Studies were categorised as Yes/ No as to whether they had assessed the key domains described in the Consensus Statement. Key domains that were assessed [16] and examples of likely tests that were recorded are as follows:
    - Attention: Sustained and divided attention: Consensus statements recommend the Trail-Making Test parts A* and B*.[17,18]
    - Verbal memory: Consensus statements recommend the RAVLT and Rey Auditory Verbal Learning Test*.[18]
    - Visual-spatial: as the Block Design from the Wechsler Adult Intelligence Scale.[19]

For peer review only - http://bmjopen.bmj.com/site/about/guidelines.xhtml

- Psychomotor speed: Consensus statements recommend tests such the Digit Symbol Test from the Wechsler Adult Intelligence Scale.[19]
- Executive function/Verbal fluency: Consensus statements recommend tests such the Controlled Oral Word Association Test.[20]
- Motor coordination: Consensus statements recommend tests such the Grooved Pegboard Test*, dominant and non-dominant hand.[17]
- Frontal Lobe: Saccadic and anti-saccadic eye movements.

Assessment of important covariates (Mini Mental State, Anxiety and Depression, Intellectual Ability, and Concomitant medication) in these analyses were documented.

6. Neurocognitive dysfunction, as reported by the study authors; A consensus definition is a change in a single test of >1 SD. This was defined as either change >1 SD in a group mean (adjusted for baseline) or a change in neurocognitive scores of >1 SD for individual patients.[21] Studies were further categorised as defining cognitive dysfunction using a Consensus versus a non-Consensus definitions.
7. Risk of receiving blood transfusion as defined by study authors.
8. Reoperation for bleeding as defined by study authors.
9. Resource Use: ICU and hospital LOS as defined by study authors.
10. S100B levels, a marker of brain injury, as reported by study authors.

Search methods for identification of studies

Potentially eligible trials were identified by searching the *Cochrane Central Register of Controlled Trials*, *MEDLINE* , and *EMBASE* and the *Cumulative Index to Nursing and Allied Health Literature (CINAHL) Plus*, using a combination of subject headings and text words to identify relevant trials. The search was performed from Inception until November 2016. The Medline search strategy below was adapted as appropriate for other databases:

For peer review only - http://bmjopen.bmj.com/site/about/guidelines.xhtml

((Cardiopulmonary Bypass) OR (Cardiac Surgery) OR (Coronary Artery Bypass) OR (Extra Corporeal Circulation) OR (Perioperative Morbidity)) AND ((Near Infra-Red Spectroscopy) OR (Oximetry) OR (Brain/Metabolism) OR (Cerebral Desaturation) OR (Cerebral Perfusion) OR (Cerebral Ischemia) OR (Cerebral Oximetry) OR (Cerebral Saturation) OR (Near Infrared Oximetry) OR (Cognitive)).

To identify on going or unpublished trials we also searched the Clinicaltrials.gov using the following search terms:

Search terms: Randomized

Study Type: Interventional Studies

Conditions: ((Cardiopulmonary Bypass) OR (Cardiac Surgery) OR (Coronary Artery Bypass) OR (Extra Corporeal Circulation) OR (Perioperative Morbidity))

Interventions: ((Near Infra-Red Spectroscopy) OR (Oximetry) OR (Brain/Metabolism) OR (Cerebral Desaturation) OR (Cerebral Perfusion) OR (Cerebral Ischemia) OR (Cerebral Oximetry) OR (Cerebral Saturation) OR (Near Infrared Oximetry) OR (Cognitive))

We will also examined the reference lists of eligible trials and reviews. Searches were not restricted by language or publication status.

Data Collection and Analysis

The review was performed in accordance with instructions given in the Cochrane Handbook for Systematic Reviews of Interventions.[22]

Selection of Studies

Two reviewers GJM and GFS identified trials for inclusion independently of each other. Excluded studies and the reason for exclusion were recorded.

Data extraction (selection and coding)

Two authors independently screened the search output to identify records of potentially eligible trials examining the outcomes, the full texts of which were retrieved and assessed for inclusion.

For peer review only - http://bmjopen.bmj.com/site/about/guidelines.xhtml

A standardised form was used to extract data from the included studies for assessment of study quality and evidence synthesis. Extracted information included:

- Year and language of publication
- Country of Participant recruitment
- Year of conduct of the trial
- Study setting; university teaching hospital, non-university teaching hospital
- Study population; inclusion and exclusion criteria
- Sample size
- Participant demographics
- Baseline characteristics
- Type of surgery
- Details of NIRS algorithm (Murkin, non Murkin[23]) and co-interventions (restrictive versus non-restrictive transfusion thresholds)
- Details of Comparator; non NIRS goal directed therapy, standard care (protocolised care)
- Outcomes and times of measurement

Two review authors extracted data independently; discrepancies were identified and resolved through discussion. Missing data was requested from study authors. If there is doubt as to whether trials shared participants completely or partially (with common authors and centres) we contacted the study authors to ascertain whether the study report has been duplicated.

Risk of Bias

The following bias risk domains were assessed as Low, Uncertain, or High based on the instructions given in the in the Cochrane Handbook for Systematic Reviews of Interventions;[24]

- Sequence Generation
- Allocation Concealment
- Blinding of participant, personnel

For peer review only - http://bmjopen.bmj.com/site/about/guidelines.xhtml

- Blinding of outcome Assessors
- Incomplete outcome data
- Selective outcome reporting
- Source of funding bias

Trials were classified as having a low risk of bias if they are graded as being at low risk of bias in all of these domains. Two review authors independently assessed the risk of bias in all of the studies. Discrepancies were resolved by discussion.

Assessment of Reporting Bias

Where 10 or more studies were identified for each outcome we assessed publication bias by the visual assessment of funnel plots and Eggers test.[25]

Measures of treatment Effect

For dichotomous variables, we calculated the risk ratio (RR) with 95% confidence interval (CI). For continuous variables, we calculated the mean difference (MD) with 95% CI for outcomes such as hospital stay, and standardised mean difference (SMD) with 95% CI for quality of life (when different scales were used).

Dealing with missing data

We perform an intention-to-treat analysis where possible. For dichotomous data presented only as percentages we estimated frequencies using reported sample sizes for this outcome. For continuous outcomes if the mean and the standard deviation were not available from the trial report, we sought this information from the trial authors. If this information was still not available, we calculated the mean and standard deviation from median (interquartile ranges) using the software available in Review Manager Version 5.

Assessment of Heterogeneity

We anticipated that major sources of clinical heterogeneity would associated with different patient groups (adults, children, congenital versus acquired disease), the use of different goal directed NIRS algorithms,

For peer review only - http://bmjopen.bmj.com/site/about/guidelines.xhtml

the use of co-interventions such as restrictive transfusion thresholds, and differences in the methodology used to assess neurocognitive dysfunction. We explored heterogeneity within each meta-analysis using a Chi^2 test with significance set at a P value of 0.10, and expressed the percentage of heterogeneity due to variation rather than to chance as I^2.[26] We defined heterogeneity as follows:

I^2 0-40%: no or mild heterogeneity

I^2 40-80%: moderate heterogeneity

I^2 > 80%: severe heterogeneity

In the presence of severe heterogeneity meta-analysis was not performed.

Data Synthesis

Meta-analyses was performed using the software package Review Manager version 5.2 and in accordance with the recommendations of the *Cochrane Handbook for Systematic Reviews of Intervention.*[14] For the primary analysis we compared the results of a random-effects model versus a fixed effects model to assess the effects of small studies. For continuous outcomes, pooled mean differences or standardised mean differences by using the inverse variance method.

Subgroup Analyses

Subgroup analyses was performed in trials in which the Murkin algorithm was used to guide goal directed therapy versus those that did not, and by participant group; CABG versus non CABG, adults versus children, assessment of neurocognitive function that incorporated tests described in a previous consensus statement, and studies that combined the NIRS algorithm with a restrictive red cell transfusion trigger. Test for sub-group differences with Review Manager was used with a P value of <0.05 considered statistically significant.[27]

Sensitivity Analyses

Sensitivity analysis excluded trials with high risk of bias for any of the following: random sequence generation; allocation concealment, blinding of participants, healthcare providers or outcome assessors, incomplete outcome data, attrition, and other sources of bias including source of funder.

For peer review only - http://bmjopen.bmj.com/site/about/guidelines.xhtml

Summary of findings

We presented the main results of the review in a 'Summary of findings' table. We included the following outcomes.

- Risk of mortality
- Risk of stroke, myocardial infarction, or severe acute kidney injury
- Risk of red cell transfusion
- Neurocognitive Impairment
- Resource Use: ICU and Hospital length of stay.

We used GRADEpro software to prepare the 'Summary of findings' table. We judged the overall quality of the evidence for each outcome as 'high,' 'moderate,' 'low' or 'very low' according to the GRADE (Grades of Recommendation, Assessment, Development and Evaluation) approach.[27] We considered the following.

- Impact of risk of bias of individual trials.
- Precision of pooled estimate. Inconsistency or heterogeneity (clinical, methodological and statistical).
- Indirectness of evidence.
- Impact of selective reporting and publication bias on effect estimate.

Results

Description of Studies

Search Results: We identified a total of 17792 references through electronic searches of the Cochrane Central Register of Controlled Trials (CENTRAL) (n=1347), MEDLINE (n=9924), EMBASE (n=6159), and the Cumulative Index to Nursing and Allied Health Literature (CINAHL) Plus (n=362). From ClinicalTrials.gov, we identified 18 trials that potentially met the inclusion criteria. We have shown the flow of search results in Figure 1. We excluded 7909 duplicates, then 9820 clearly irrelevant references, by reading titles and abstracts. No additional references were identified by reference searching. Of 79 study reports retrieved in

For peer review only - http://bmjopen.bmj.com/site/about/guidelines.xhtml

full text, we excluded 69 references for the reasons listed in Figure S1 in the online only digital supplement. In total, 11 publications describing 9 randomised controlled trials fulfilled the inclusion and exclusion criteria provided quantitative data for this review [10,28-35] (Table S1 in the online only digital supplement). We also included data from a recent trial undertaken by our own unit, the results of which are in press.[36]

Description of excluded studies: Four trials that met our inclusion criteria were excluded after review of the full manuscript (Table S2 in the online only digital supplement). There were two additional reports from one trial; an interim analysis,[37] and a post hoc analysis,[38] that was also reported in full.[10] Another trial compared NIRS values in on- versus of-pump CABG patients.[39] A fourth trial by Dullenkopf and colleagues[40] was reported in the abstract as an RCT, however the randomisation of placing the NIRS sensors on either the right or left for head and there was no clinical NIRS intervention versus a control comparison.

Included studies: Ten trials that evaluated NIRS based algorithms in patients undergoing cardiac surgery were identified.[10,28-35]

Participants: Overall, 1466 participants took part in the 10 trials included in the review. The average age of participants in these trials ranged from 34.6 ± 16.3 to 71 ± 11.2 years and the proportion of females ranged from 12.5% to 66% in the trials that provided this information.[10,28-35] The proportion of post randomisation withdrawals ranged from 0% to 4.11%. After withdrawal occurred, 1452 participants were included in the quantitative meta-analyses in this systematic review. Five trials were conducted in patients undergoing CABG only,[10,28,31,32,34] and 5 trials were conducted in patients undergoing valve or CABG and valve surgery or other cardiac surgical procedure.[29,30,33,35,36] Further details of participants are listed in Table S1 in the online only digital supplement.

Intervention: Cerebral NIRS values were measured with the INVOS (Somanetics Corporation, Troy, MI) in 9 trials. In 1 trial[29] three different devices were used; the INVOS, the FORE-SIGHT (CAS Medical Systems Inc., USA), and the EQUANOX Classic 7600 (Nonin Medical Inc., USA). In 2 trials the target NIRS values were >75% of baseline.[10,31] In 4 trials the targets values were >80%.[29,30,32,34] In 1 trial the target NIRS values were

For peer review only - http://bmjopen.bmj.com/site/about/guidelines.xhtml

greater than an absolute NIRS value of 60% or >20% compared to the mean value during pulmonary artery catheter insertion.[35] In 2 trials the target NIRS value was a combination of >80% of baseline or an absolute measure > 50%.[28,33] In 1 trial the target regional oxygen saturation values were specified as >70% of pre-induction values or an absolute value of >50%.[36] Seven trials used a version of the Murkin algorithm to optimise NIRS values.[10,28-31,33,34,36] Two studies used non-Murkin algorithms.[32,35]

Control: All trials used standard (protocolised) care as the control group. In 7 trials NIRS values were measured in the control group, although the values were hidden from the clinical staff.[10,29-31,33,34,36] No trial considered an alternative patient specific goal directed algorithm.

Co-interventions: In 2 trials the Murkin algorithm was combined with a restrictive transfusion trigger.[35,36] In these studies a pre-specified objective was to determine whether NIRS could be utilised as part of a patient specific red cell transfusion indicator.

Risk of bias in included studies

Risk of bias in individual trials is shown in Figure 2A, and the proportions of trials with low risk, unclear risk and high risk of bias in each of the domains are shown in Figure 2B. Clinical personnel were not blinded in any trial. However, 2 trials were considered at low risk of bias in every domain.[35,36]

Sequence generation: Random sequence generation was adequate in 7 trials,[10,28,29,31,34-36] and unclear in 2 trials.[30,33] There was a high risk of bias for random sequence generation in 1 trial.[32]

Allocation: Allocation concealment was adequate in 7 trials,[10,28,29,31,34-36] and unclear in 2 trials.[30,33] There was a high risk of bias for random sequence generation in 1 trial.[32]

Blinding: Theatre staff were unblinded to group allocation in all of the studies. There was evidence of blinding of patients and clinical staff caring for patients postoperatively in 4 trials,[30,32,35,36] and unclear evidence in 6 trials.[10,28,29,31,33,34] Two trials reported the frequency of protocol compliance.[35,36] In 8 trials non-compliance was not monitored or not specified. There was evidence of blinding of outcome assessors

For peer review only

For peer review only - http://bmjopen.bmj.com/site/about/guidelines.xhtml

in 5 trials [10,28,32,35,36] and unclear evidence of blinding of outcome assessors in 5 trials.[29-31,33,34]

Incomplete outcome data: 6 trials reported completeness of follow-up for the primary outcome.[10,29,31,32,35,36] Of these, 5 trials reported <10% loss to follow-up, and 1 trial reported >10% loss to follow-up.[35] Four trials that failed to report completeness of follow-up were considered to be at high risk of attrition bias.[28,30,33,34]

Selective reporting: Only 1 of the 10 trials included in this review had a published trial protocol.[36] Another 5 trials had reported details of primary and secondary outcomes in trial registries.[28-30,34,35] Of these, 3 trials failed to report all the pre specified outcomes.[28,30,34] Of the remaining four trials, none were registered or had published protocols, and were considered at high risk of selective outcome reporting bias.

Source of funding bias: Sources of funding were reported in 6 trials. Of these 2 trials were funded by independent sources. In 1 trial the study was supported in part by the NIRS device manufacturer and was therefore considered at high risk of funding bias.[10] Three studies that failed to report the source of funding were considered at uncertain risk of funding bias (Table 1).

Effects of intervention

The summary effect estimates for primary and secondary outcomes are described in Table 1, Figure 2, and Figure S2 in the online only digital supplement.

Mortality: Four trials with 608 participants reported this outcome.[10,30,35,36] There was no statistically significant difference in mortality between NIRS versus controls; RR 0.76, 95% CI 0.30 to 1.96; I^2=0%; Chi^2 test for heterogeneity P value 0.92.

Stroke: Seven trials with 1138 participants reported this outcome.[10,28,29,33-36] There was no statistically significant difference in the frequency of stroke between NIRS versus controls; RR 1. 08, 95% CI 0.40 to 2.91; I2=0%; Chi2 test for heterogeneity P value 0.46.

For peer review only

For peer review only - http://bmjopen.bmj.com/site/about/guidelines.xhtml

Myocardial infarction: Six trials with 1038 participants reported this outcome.[10,28,29,34-36] There was no statistically significant difference in the frequency of myocardial infarction between NIRS versus controls; RR 0.90, 95% CI 0.43 to 1.89; I2=0%; Chi2 test for heterogeneity P value 0.86.

Severe Acute Kidney injury: Six trials with 1064 participants reported this outcome.[10,28,30,34-36] There was no statistically significant difference in the frequency of severe acute kidney injury between NIRS versus controls; RR 0.88, 95% CI 0.58 to 1.34; I2=0%; Chi2 test for heterogeneity P value 0.89.

Red cell transfusion: Four trials with 744 participants reported this outcome.[10,28,35,36] There was no statistically significant difference in the frequency of red cell transfusion between NIRS versus controls; RR 0.93, 95% CI 0.77 to 1.12; I2=51%; Chi2 test for heterogeneity P value 0.11.

Reoperation for Bleeding: Four trials with 744 participants reported this outcome.[10,28,35,36] There was no difference in the frequency of reoperation for bleeding between NIRS versus controls; RR 1.11, 95% CI 0.41 to 3.04; I2=0%; Chi2 test for heterogeneity P value 0.69.

Neurocognitive testing and measuring neurocognitive dysfunction: Five trials that recruited 813 patients reported this outcome.[28,32-34,36] One trial[29] reported only the incidence of delirium. Details of these trials are listed in Table 2. The Consensus Statement for the Assessment of Neurocognitive function in Cardiac Surgery[15] recommends that that the following Core tests be performed at baseline and up to 3 months post-surgery; the Rey auditory verbal learning test, the Trail-Making test Part A and Trail-Making Test Part B, and the Grooved Pegboard test to assess the neurocognitive domains Attention, Verbal Memory and Motor Coordination. The Consensus Statement defines cognitive decline as a difference for the individual of >1SD from baseline, or a difference of >1SD between group means, with adjustment for baseline for at least one test. There was significant heterogeneity for this outcome. Only 2 trials[34,36] measured cognitive function as recommended by the consensus statement. Both trials reported no difference between the groups for neurocognitive function. In 1 trial no test data was presented.[34] In the other[36] there was a significant difference between the groups for the Controlled Oral Word Association Test, which assesses

For peer review only - http://bmjopen.bmj.com/site/about/guidelines.xhtml

the domain Executive Function/ Verbal Fluency, however this is not a specified core domain in the consensus statement. The three remaining trials used non-consensus testing protocols and non-consensus definitions of neurocognitive decline, and only 1 tested patients at 3 months post-surgery. Because of the degree of heterogeneity we did not perform meta-analyses of these outcome.

ICU LOS: Eight trials with 1051 participants reported this outcome.[10,28-30,32,33,35,36] There were no statistically significant differences in the duration of ICU stay between NIRS versus controls with moderate heterogeneity; RR MD 0.00, 95% CI -0.44 to 0.44; I^2=73%; Chi2 test for heterogeneity P value 0.0005.

Hospital LOS: Six trials with 761 participants reported this outcome.[10,29,30,32,35,36] Hospital LOS was less in the NIRS group however this was not statistically significant; RR MD -0.45, 95% CI -0.90 to 0.01; I^2=0%; Chi2 test for heterogeneity P value 0.83.

S100B: Two trials reported this outcome in 138 patients.[31,36] One trial with 40 participants measured S100B pre- and postoperatively and reported a significant reduction in S100B in NIRS patients, MD -99.87, 95% CI -105.18 to -94.56. The time of the post-surgery sample was not reported. Another with 98 participants measured S100B preoperatively and at 4 post-surgery time points; on return to ICU, and at 6, 12-24, 24-48 and 96 hours. There was no difference between the groups; Ratio of Geometric Means 1.06, 95% CI (0.95, 1.19), p=0.29. No meta-analysis was performed due to the heterogeneity for this outcome.

Subgroup analyses

Results of these subgroup analyses for Murkin vs non- Murkin algorithms, CABG vs non CABG, and assessment of neurocognitive function using tests described in a previous consensus statement are shown in Table S3 in the online only digital supplement.

Publication Bias: A funnel plot of standard error versus risk ratio for the included outcomes showed an asymmetrical distribution that indicated publication bias. However, since there was an insufficient number of trials providing data (less than 10 studies identified for each outcome), we did not perform this analysis.

For peer review only - http://bmjopen.bmj.com/site/about/guidelines.xhtml

For peer review only

Sensitivity Analyses

Fixed effects models did not materially change the results of our primary analyses. We also conducted sensitivity analyses in two trials identified as being at low risk of bias.[35,36] These trials reported outcomes in 328 participants. Both trials incorporated restrictive red cell transfusion thresholds within the NIRS algorithm. They reported no difference between NIRS and control groups for mortality: RR 0.67, 95% CI 0.11 to 4.08; I2=0%; Chi2 test for heterogeneity P value 0.71; stroke: RR 3.0 95% CI -0.32to 28.54; I2=0%; Chi2 test for heterogeneity P value 1.00; myocardial infarction: RR 0.76, 95% CI 0.17 to 3.41; I2=0%; Chi2 test for heterogeneity P value 0.66; severe acute kidney injury: RR 0.83, 95% CI 0.44 to 1.54; I2=0%; Chi2 test for heterogeneity P value 0.42; reoperation for bleeding: RR 1.50, 95% CI 0.48 to 4.62; I2=0%, Chi2 test for heterogeneity P value 0.75; ICU length of stay: RR-0.03, 95% CI -0.69 to 0.62; I2=0%; Chi2 test for heterogeneity P value 0.95 and hospital length of stay: RR -0.20, 95% CI -1.29 to 0.89; I^2=0%; Chi^2 test for heterogeneity P value 0.45. Analyses of these trials suggested that the use of NIRS based algorithms resulted in reductions in red cell transfusion: RR 0.83, 95% CI 0.71 to 0.98; I2=0%; Chi2 test for heterogeneity P value 0.52, One of these trials, the PASPORT trial [36] reported the results of neurocognitive assessments and concluded that there was no difference between NIRS and control groups.

GRADE Assessment

A summary of the main findings of the review are presented in Table 3. GRADE assessments of the results were either Low or Very Low for all the outcomes, indicating a high likelihood that these conclusions may be altered by subsequent trials.

For peer review only - http://bmjopen.bmj.com/site/about/guidelines.xhtml

Discussion

Main Findings

A systematic review and meta-analysis of existing trials did not demonstrate clinical benefits attributable to the use of personalised NIRS based algorithms during CPB. The use of NIRS did not result in reductions in mortality, injury to the brain, heart or kidneys, or reductions in resource use. A qualitative review of studies that had evaluated the effects of NIRS on neurocognitive function did not show clear evidence of benefit. An analysis of two trials at low risk of bias where NIRS was applied along with a restrictive red cell transfusion threshold demonstrated a reduction in red cell transfusion with this approach, with no difference between NIRS treated and controls with respect to clinical outcomes or resource use. Overall the GRADE quality of the evidence was low or very low for all of the outcomes measured.

Strengths and weaknesses

This is the most comprehensive evaluation of NIRS based patient specific goal direct algorithms in cardiac surgery to date. The results supersede those of a previous quantitative review of NIRS in cardiac surgery that considered randomised (2 studies) and observational analyses (27 studies).[41] Our searches also identified a Cochrane review protocol with similar aims however the results of this review have yet to be published.[42] The current review used comprehensive search strategies in a wide range of registries and data sources, had access to the full texts of all identified trials, used contemporary risk of bias assessments (GRADE), and assessed a wide range of outcomes after cardiac surgery. The main limitation of the review is that we did not have access to all of the source data. Although additional unpublished information was also obtained from three authors and included in the meta-analysis we realise that consistent analyses of all studies can only be done when data on individual patients are combined. In addition, the review identified important limitations of existing data; all of the 10 RCTs had limitations in terms of methodological quality. The risk of procedural bias was high in these trials as there was no blinding of clinical personnel. Furthermore only two from 10 trials attempted to define the likelihood of procedural bias by describing the degree of protocol adherence. The reporting of outcomes was also heterogeneous between trials limiting

For peer review only - http://bmjopen.bmj.com/site/about/guidelines.xhtml

the number of studies that could be included in each outcome. This was most evident for the outcome cognitive function. Many trials did not report important clinical outcomes; such as death, although it is highly likely that this outcome was measured. Furthermore, in many cases although the trial report stated that important assessments had been made, particularly with respect to the testing of cognitive function, the results of these assessments were not reported. Despite these limitations the results were remarkably consistent, with low or no heterogeneity for all of the analyses, all of which suggested that there are no clinical benefits attributable to the use of NIRS based algorithms.

Clinical Importance

NIRS technology and NIRS based algorithms are used in cardiac surgery centres worldwide, although there is clear evidence of equipoise with respect to the clinical benefits this technology.[12,13] The results presented here do not support the hypotheses that the use of NIRS may reduce brain injury,[8] or by using the brain as the index organ, reducing injury to the heart or kidneys as the result of improved overall perfusion during cardiac surgery with cardiopulmonary bypass.[9] They do not provide insights into the role of NIRS in procedures that require deep hypothermic circulatory arrest. It is possible that our results are attributable to chance; our GRADE assessment of the systematic review results was very low for all the pre-specified outcomes, indicating a high likelihood that these conclusions may be altered by subsequent trials. There was almost no heterogeneity for these outcomes, however, and these findings were consistent with the results of the PASPORT trial.[36] This multicentre trial recruited a larger cohort than almost all the previous trials, was at low risk of bias, and demonstrated no benefit for NIRS based algorithms for a range of outcomes including cognitive function, and biomarkers of myocardial, renal and neurological injury.[36]

Our systematic review indicated that the combination of NIRS and a restrictive transfusion threshold resulted in a reduction in red cell transfusion, without any difference between NIRS treated patients and controls with respect to clinical outcomes or resource use. This may be interpreted as showing that NIRS may be used safely to implement restrictive transfusion thresholds. These findings must be interpreted

For peer review only

with caution however, these 2 trials enrolled only 387 patients, clinical staff were not blinded to group allocation in these trials, and endpoints such as red cell transfusion are susceptible to performance bias. A final consideration is that the review did not identify any trial in paediatric cardiac surgery, a common setting for the use of these devices, and thus has identified a knowledge gap with respect to the utility of this intervention in these patients.

Conclusions

Existing evidence suggests that the use of NIRS based patient specific algorithms that aim to optimise cerebral oximetry do not result in reductions in mortality, major morbidity or resource use in adult cardiac surgery. Assessment of the quality of the evidence indicates that there is a need for further randomised trials at low risk of bias to assess the clinical utility of NIRS in both adult and paediatric cardiac surgery. To determine the clinical utility of these devices future studies should be designed to evaluate effectiveness (freedom from death and disability) as well as efficacy (stroke, renal failure, low cardiac output).

Sources of Funding

This work was supported by the National Institute for Health Research Programme Grants for Applied Health number RP-PG-0407-10384. GFS is supported by the Leicester NIHR Cardiovascular Biomedical Research Unit. GJM is supported by British Heart Foundation Grants RG/13/6/29947 and CH/12/1/29419.

Disclosures

Both authors have completed the Unified Competing Interest form at www.icmje.org/coi_disclosure.pdf (available on request from the corresponding author). Dr Serraino declares no conflict of interest. Professor Murphy reports grants from British Heart Foundation during the conduct of the study and grants from British Heart Foundation, grants from National Institute for Health Research, grants from Zimmer Biomet, personal fees from Abbvie, and personal fees from Thrasos Inc, outside the submitted work.

For peer review only

Details of Contributors

Both of the study authors, external and internal, had full access to all of the data (including statistical reports and tables) in the study and can take responsibility for the integrity of the data and the accuracy of the data analysis. Murphy conceived and designed the study, and undertook the systematic review. Serraino wrote the protocol, undertook the systematic review, performed the analysis and drafted the report along with Murphy. Both authors reviewed the report for important intellectual content and approved the final version.

Acknowledgements

The authors gratefully acknowledge the assistance of the Mr Giovanni Mariscalco who reviewed the study protocol for important intellectual content.

Data Sharing

No additional data available.

For peer review only - http://bmjopen.bmj.com/site/about/guidelines.xhtml

References:

1. Selnes OA, Gottesman RF, Grega MA, Baumgartner WA, Zeger SL, McKhann GM. Cognitive and neurologic outcomes after coronary-artery bypass surgery. N Engl J Med 2012; 366: 250-7
2. Murphy GJ, Angelini GD. Side effects of cardiopulmonary bypass: what is the reality? J Card Surg 2004; 19: 481-8
3. Kim MB, Ward DS, Cartwright CR, Kolano J, Chlebowski S, Henson LC. Estimation of jugular venous O2 saturation from cerebral oximetry or arterial O2 saturation during isocapnic hypoxia. J Clin Monit Comput 2000; 16: 191-9
4. Sapire KJ, Gopinath SP, Farhat G, et al. Cerebral oxygenation during warming after cardiopulmonary bypass. Crit Care Med 1997; 25: 1655-62
5. Croughwell N, Lyth M, Quill TJ, et al. Diabetic patients have abnormal cerebral autoregulation during cardiopulmonary bypass. Circulation 1990; 82: IV407-12
6. Miyoshi S, Morita T, Kadoi Y, Goto F. Analysis of the factors related to a decrease in jugular venous oxygen saturation in patients with diabetes mellitus during normothermic cardiopulmonary bypass. Surg Today 2005; 35: 530-4
7. Vretzakis G, Georgopoulou S, Stamoulis K, et al. Cerebral oximetry in cardiac anesthesia. J Thorac Dis 2014; 6 Suppl 1: S60-9
8. Goldman S, Sutter F, Ferdinand F, Trace C. Optimizing intraoperative cerebral oxygen delivery using noninvasive cerebral oximetry decreases the incidence of stroke for cardiac surgical patients. Heart Surg Forum 2004; 7: E376-81
9. Murkin JM. Cerebral oximetry: monitoring the brain as the index organ. Anesthesiology 2011; 114: 12-3
10. Murkin JM, Adams SJ, Novick RJ, et al. Monitoring brain oxygen saturation during coronary bypass surgery: a randomized, prospective study. Anesth Analg 2007; 104: 51-8
11. Klein HG, Flegel WA, Natanson C. Red Blood Cell Transfusion: Precision vs Imprecision Medicine. JAMA 2015; 314: 1557-8
12. Kane JM, Steinhorn DM. Lack of irrefutable validation does not negate clinical utility of near-infrared spectroscopy monitoring: learning to trust new technology. J Crit Care 2009; 24: 472 e1-7
13. Zacharias DG, Lilly K, Shaw CL, et al. Survey of the clinical assessment and utility of near-infrared cerebral oximetry in cardiac surgery. J Cardiothorac Vasc Anesth 2014; 28: 308-16
14. Higgins JPT, Green, S. Cochrane Handbook for Systematic Reviews of Interventions Version 5.1.0, The Cochrane Collaboration, 2011
15. Murkin JM, Newman SP, Stump DA, Blumenthal JA. Statement of consensus on assessment of neurobehavioral outcomes after cardiac surgery. Ann Thorac Surg 1995; 59: 1289-95
16. Mahanna EP, Blumenthal JA, White WD, et al. Defining neuropsychological dysfunction after coronary artery bypass grafting. Ann Thorac Surg 1996; 61: 1342-7
17. Newman SP. Analysis and interpretation of neuropsychologic tests in cardiac surgery. Ann Thorac Surg 1995; 59: 1351-5
18. Spreen OSE. A compendium of neuropsychological tests, A compendium of neuropsychological tests. Edited by OSE Spreen. New York, Oxford University Press, 1998
19. Weschler D. Manual of the Wechsler Test of Adult Reading (WTAR-UK), Manual of the Wechsler Test of Adult Reading (WTAR-UK). Edited by D W. New York, Psychological Corporation (Iglesias), 2001
20. Benton AHR. Multilingual Aphasia Examination, Multilingual Aphasia Examination. Edited by AHR B. Iowa City, University of Iowa, 1976
21. Murkin JM, Stump DA, Blumenthal JA, McKhann G. Defining dysfunction: group means versus incidence analysis--a statement of consensus. Ann Thorac Surg 1997; 64: 904-5
22. Higgins JPT Deeks JJ. Selecting studies and collecting data Cochrane Handbook for Systematic Reviews of Interventions Version 5.1.0. Edited by Higgins JPT Green S, The Cochrane Collaboration, 2011

For peer review only - http://bmjopen.bmj.com/site/about/guidelines.xhtml

23. Denault A, Deschamps A, Murkin JM. A proposed algorithm for the intraoperative use of cerebral near-infrared spectroscopy. Semin Cardiothorac Vasc Anesth 2007; 11: 274-81
24. Higgins JPT Altman DG, Sterne JAC: Assessing risk of bias in included studies, Cochrane Handbook for Systematic Reviews of Interventions Version 5.1.0. Edited by Higgins JPT Green S, The Cochrane Collaboration, 2011
25. Sterne JAC Egger M, Moher D. Addressing reporting biases Cochrane Handbook for Systematic Reviews of Interventions Version 5.1.0. Edited by Higgins JPT Green S, The Cochrane Collaboration, 2011
26. Higgins JP, Thompson SG. Quantifying heterogeneity in a meta-analysis. Stat Med 2002; 21: 1539-58
27. Schünemann HJ Oxman AD, Vist GE, Higgins JPT, Deeks JJ, Glasziou P. Interpreting results and drawing conclusions, Cochrane Handbook for Systematic Reviewsof Interventions Version 5.1.0 Edited by Higgins JPT Green S, The Cochrane Collaboration, 2011
28. Colak Z, Borojevic M, Bogovic A, Ivancan V, Biocina B, Majeric-Kogler V. Influence of intraoperative cerebral oximetry monitoring on neurocognitive function after coronary artery bypass surgery: a randomized, prospective study. Eur J Cardiothorac Surg 2015; 47: 447-54
29. Deschamps A, Hall R, Grocott H, et al. Cerebral Oximetry Monitoring to Maintain Normal Cerebral Oxygen Saturation during High-risk Cardiac Surgery: A Randomized Controlled Feasibility Trial. Anesthesiology 2016; 124: 826-36
30. Deschamps A, Lambert J, Couture P, et al. Reversal of decreases in cerebral saturation in high-risk cardiac surgery. J Cardiothorac Vasc Anesth 2013; 27: 1260-6
31. Harilall Y, Adam JK, Biccard BM, Reddi A. The effect of optimising cerebral tissue oxygen saturation on markers of neurological injury during coronary artery bypass graft surgery. Heart Lung Circ 2014; 23: 68-74
32. Kara I, Erkin A, Sacli H, et al. The Effects of Near-Infrared Spectroscopy on the Neurocognitive Functions in the Patients Undergoing Coronary Artery Bypass Grafting with Asymptomatic Carotid Artery Disease: A Randomized Prospective Study. Ann Thorac Cardiovasc Surg 2015; 21: 544-50
33. Mohandas BS, Jagadeesh AM, Vikram SB. Impact of monitoring cerebral oxygen saturation on the outcome of patients undergoing open heart surgery. Ann Card Anaesth 2013; 16: 102-6
34. Slater JP, Guarino T, Stack J, et al. Cerebral oxygen desaturation predicts cognitive decline and longer hospital stay after cardiac surgery. Ann Thorac Surg 2009; 87: 36-44; discussion 44-5
35. Vretzakis G, Georgopoulou S, Stamoulis K, et al. Monitoring of brain oxygen saturation (INVOS) in a protocol to direct blood transfusions during cardiac surgery: a prospective randomized clinical trial. J Cardiothorac Surg 2013; 8: 145
36. Rogers CA, Stoica S, Ellis L, et al. A Randomised Trial of Near Infra-Red Spectroscopy for the Personalised Optimisation of Cerebral Tissue Oxygenation during Cardiac Surgery. British Journal of Anaesthesia 2017; in press
37. Murkin JM AS, Schaefer B. Monitoring cerebral oxygen saturation significantly decreases major organ morbidity in CABG patients: A randomized blinded study. Heart Surg Forum 2004; 7: 515
38. Murkin JM, Adams SJ, Pardy E, Quantz M, McKenzie FN, Guo L. Monitoring brain oxygen saturation during coronary bypass surgery improves outcomes in diabetic patients: a post hoc analysis. Heart Surg Forum 2011; 14: E1-6
39. Kok WF, van Harten AE, Koene BM, et al. A pilot study of cerebral tissue oxygenation and postoperative cognitive dysfunction among patients undergoing coronary artery bypass grafting randomised to surgery with or without cardiopulmonary bypass*. Anaesthesia 2014; 69: 613-22
40. Dullenkopf A, Baulig W, Weiss M, Schmid ER. Cerebral near-infrared spectroscopy in adult patients after cardiac surgery is not useful for monitoring absolute values but may reflect trends in venous oxygenation under clinical conditions. J Cardiothorac Vasc Anesth 2007; 21: 535-9
41. Zheng F, Sheinberg R, Yee MS, Ono M, Zheng Y, Hogue CW. Cerebral near-infrared spectroscopy monitoring and neurologic outcomes in adult cardiac surgery patients: a systematic review. Anesth Analg 2013; 116: 663-76

For peer review only

For peer review only - http://bmjopen.bmj.com/site/about/guidelines.xhtml

42. Yu Y, Zhang K, Zhang L, Zong H, Meng L, Han R. Cerebral near-infrared spectroscopy (NIRS) for perioperative monitoring of brain oxygenation in children and adults. Cochrane Database of Systematic Reviews 2014; Issue 1.

For peer review only

For peer review only - http://bmjopen.bmj.com/site/about/guidelines.xhtml

Table 1. Summary of findings for all primary and secondary outcomes

STUDY	*MORTALITY*		*MI N (%)*		*STROKE N (%)*		*AKI N (%)*		*BLEEDING N (%)*		*TRANSFUSION N (%)*		*ICU LOS (H) MEAN (SD)*		*HOSP LOS (D) MEAN (SD)*	
	NIRS	Control	NIRS	Control	NIRS	Control	NIRS	Control	NIRS	Control	NIRS	Control	NIRS	Control	NIRS	Control
**ROGERS, 2017**[36]	1 (1)	2 (1.88)	1 (1)	2 (1.88)	1 (1)	0	2 (2)	5 (4.71)	6 (6.1)	4 (3.77)	37 (37.7)	44 (45.8)	3.3 (0.81)	3.67 (0.79)	7.5 (1.15)	8 (1.17)
**DESCHAMPS, 2016** [29]	4 (11.7)	6 (13)	0	0	1 (2.9)	2 (4.3)	12 (35.2)	17 (47.2)	-	-	-	-	2.99 (2.26)	0.39 (2.05)	11 (7.2)	9.9 (5.8)
**KARA, 2015**[32]	-	-	-	-	-	-	-	-	-	-	-	-	1.74 (0.81)	2.12 (1.05)	7.15 (1.39)	7.67 (1.14)
**COLAK, 2015**[28]	-	-	8 (8.5)	7 (7.29)	4 (4.25)	1 (1)	0	1 (1)	0	1 (1)	77 (81)	73 (76)	2.7 (6.2)	1.9 (0.9)	-	-
**DESCHAMPS, 2013**[30]	-	-	-	-	-	-	-	-	-	-	-	-	2.99 (2.26)	0.39 (2.05)	7.6 (5.4)	7.9 (3.2)
**VRETZAKIS, 2013**[35]	1 (1.33)	1 (1.33)	2 (2.66)	2 (2.66)	1 (1.33)	0	1 (1.33)	0	1 (1.33)	1 (1.33)	51 (68)	63 (84)	2.7 (3.8)	2.7 (3.6)	10.9 (3.6)	10.2 (10.7)
**MOHANDAS, 2013**[33]	-	-	-	-	0	2 (4)	-	-	-	-	-	-	1.49 (0.38)	1.7 (0.49)	-	-
**HARILALL, 2013**[31]	-	-	-	-	-	-	-	-	-	-	-	-	-			

For peer review only - http://bmjopen.bmj.com/site/about/guidelines.xhtml

**SLATER, 2008[34]**	-	-	1 (0.8)	1 (0.86)	1 (0.8)	0	1 (0.8)	1 (0.86)	-	-	-	-	-	-	-	
**MURKIN, 2007[10]**	0	1 (1%)	1 (1%)	3 (3%)	1 (1%)	4 (4%)	0	0	1 (1%)	1 (1%)	8 (8)	10 (10)	1.25 (0.84)	1.87 (2.67)	6.1 (4.4)	6.9 (5.5)
**SUMMARY EFFECT ESTIMATE RR(95% CI)**	**0.76 (0.30 to 1.96)**		**0.90 (0.43 to 1.89)**		**1.08 (0.40 to 2.91)**		**0.88 (0.52 to 1.49)**		**1.23 (0.46 to 3.32)**		**0.93 (0.77 to 1.12)**		**0.00 (-0.44 to 0.44)**		**-0.45 (-0.90 to 0.01)**	

MI, Myocardial Infarction, AKI, Acute Kidney Injury, ICU, Intensive Care Unit, Hosp, Hospital, Length of Stay, LOS.

For peer review only

For peer review only - http://bmjopen.bmj.com/site/about/guidelines.xhtml

Table 2. Assessment of Neurocognitive Dysfunction

Domain	Test Protocol	Kara, 2015[32]	Colak, 2015[28]	Rogers, 2017[36]	Mohandas, 2013[33]	Slater, 2008[34]
**Timing**		Baseline and and pre-discharge	Baseline and 7 days	Baseline, 7 days and three months	Baseline, 7 days and three months	Baseline,pre-discharge and three months
**Attention**	Trail- Making Test parts A and B or equivalent		X (CTT)	X		X (SCW)
**Verbal Memory**	Rey Auditory Verbal Learning Test (VLT) or Hopkins VLT			X		X
**Visuo-Spatial**	Block Design from the Wechsler Adult Intelligence Scale			X		
**Psychomotor Speed**	Digit Symbol Test from the Wechsler Adult Intelligence Scale			X		
**Executive Funtion/ Verbal Fluency**	Controlled Oral Word Association Test			X		
**Motor Cordination**	Grooved Pegboard Test		X	X		X
**Other**	Saccadic and anti-saccadic eye movements		X		X	X
	Montreal Cognitive Asessment	X				
**Measurement of Confounders**						
	MMSE		X	X	X	X
	Anxiety Depression			X		X

For peer review only

For peer review only - http://bmjopen.bmj.com/site/about/guidelines.xhtml

Domain	Test Protocol	Kara, 2015[32]	Colak, 2015[28]	Rogers, 2017[36]	Mohandas, 2013[33]	Slater, 2008[34]
	Adult Reading Test			X		
	Medication			X		
	Delirium Rating Score					X
**Presented Test Data**		X		X	X	
**Definition of Cognitive Decline**		The maximum score to get from this test is 30. >25=Normal 19–25= mild cognitive imp. <19= serious cognitive imp..	Dichotomous:decrease in an MMSE score for three or more points from baseline value AND decrease of 1 SD (standard deviation) or more in performance on CTT 1 and GP tests.	Difference in group mean (p<0.05) in >3 of 6 domains	Postoperative MMSE impairment was defined as a decrease in scores by more than 20% of the preoperative values. Postoperative ASEM impairment was defined as a decrease of scores to more than 30% of preoperative values.	Cognitive decline was defined as a decline of one standard deviation or more in performance on one or more of the neuropsychologic tests.
**Reportd Difference in Cognitive Function**		X	X		X	
**Estimate of Difference**		Mild Cognitive Impairment 7/43 NIRS, 16/36 Control, p=0.01.Severe Cognitive Impairment 0/42 NIRS, 3/36 Control, p-0.09	28% NIRS vs 52% Control, p=0.0002	Significant difference in COWAT Mean Difference 3.73 (95%CI 1.5 to 5.96), p=0.0011	ASEM at 3 months Mean 15.69 (SD 3.99) NIRS, Mean 17.68 (SD 1.79), p<0.001	58% NIRS vs 61% Control

CTT: Colour Trail Test 1, SCW: Stroop Colour and Word; GP Test: Grooved Pegboard Test; MMSE: Mini- Mental State Examinations; ASEM: Anti saccadic eye

For peer review only

For peer review only - http://bmjopen.bmj.com/site/about/guidelines.xhtml

movements.

For peer review only

For peer review only - http://bmjopen.bmj.com/site/about/guidelines.xhtml

Table 3. Summary of main findings of systematic review and Grade Assessment of trial results.

**Near Infra Red Spectrodscopy Algorithm compared to Control (Standard Care) in Cardiac Surgery**					
**Patient population:** Adult Cardiac surgery, **Setting:** Tertiary cardiac centres					
**Intervention:** Near Infra Red Spectroscopy Algorithms for Personalised Optimisation of Cerebral Oxygenation					
**Control:** Standard care					
Outcomes	Anticipated absolute effects* (95% CI)		Relative Effect (95% CI)	N of Participants (studies)	Quality og the evidence (GRADE)
	Risk with Control	Risk with NIRS			
Mortality	32 per 1000	**25 per 1000** (10 to 63)	**RR 0.76** (0.30 to 1.96)	608 (4 RCTs)	⊕⊕◯◯ Low
Red cell transfusion	504 per 1000	**469 per 1000** (388 to 564)	**RR 0.93** (0.77 to 1.12)	744 (4RCTs)	⊕⊕◯◯ Low
Stroke	16 per 1000	**17 per 1000** 6 to 46)	**RR 1.08** (0.40 to 2.91)	1138 (7 RCTs)	⊕◯◯◯ Very Low
Myocardial infarction	29 per 1000	**26 per 1000** (12 to 54)	**RR 0.90** (0.43 to 1.89)	1038 (6 RCTs)	⊕◯◯◯ Very Low

For peer review only
For peer review only - http://bmjopen.bmj.com/site/about/guidelines.xhtml

Renal failure	71 per 1000	**62 per 1000**<br>(41 to 95)	**RR 0.88**<br>(0.58 to 1.34)	1043 (6 RCTs)	⊕◯◯◯<br>Very Low
Reoperation for bleeding	19 per 1000	**21 per 1000**<br>(8 to 56)	**RR 1.11**<br>(0.41 to 3.04)	744 (4 RCTs)	⊕◯◯◯<br>Very Low
ICU length of Stay (ICU LOS)		The mean ICU LOS in the intervention group was 0 (0.44 lower to 0.44 higher)			⊕◯◯◯<br>Very Low
Hospital length of stay (H LOS)		The mean H LOS was 0.45 lower (0.9 lower to 0.01 higher)			⊕⊕◯◯<br>Low
***The risk in the intervention group** (and its 95% confidence interval) is based on the assumed risk in the comparison group and the **relative effect** of the intervention (and its 95%CI)					
CI: Confidence interval; RR: Risk ratio; MD: Mean Difference; ICU: Intensive Care Unit; NIRS: Near Infra-red Spectroscopy					

For peer review only

Figure Legends

Figure 1. Risk of bias summaries for **A.** Individual studies and **B.** All studies**.**

Figure 2. Forrest plots demonstrating summary effect estimates for A. Mortality**, B.** Myocardial Infarction**, C.** Stroke**, D.** Stage 3 acute kidney injury or renal replacement therapy**.** Effect estimates derived using Random Effects meta-analysis. Heterogeneity expressed as Chi Squared and I^2.

For peer review only

For peer review only - http://bmjopen.bmj.com/site/about/guidelines.xhtml

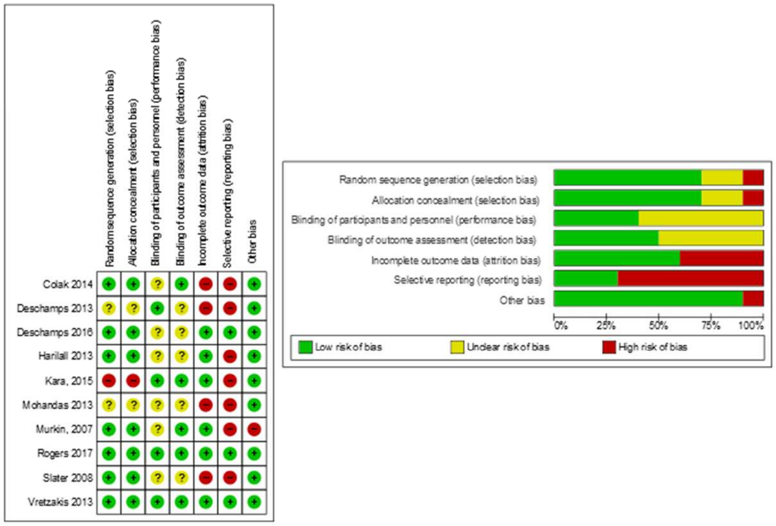

Figure 1.

197x278mm (96 x 96 DPI)

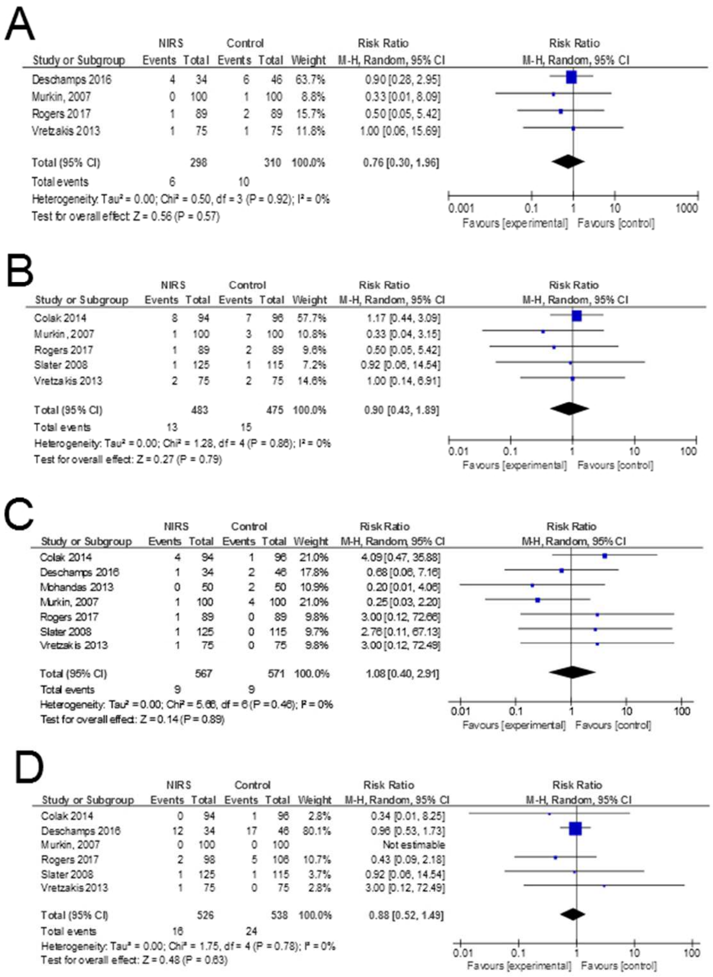

Figure 2.

197x278mm (96 x 96 DPI)

Online only digital supplement: Efficacy of Near-Infrared Spectroscopy on the outcome of patients undergoing cardiac surgery: a systematic review of randomised trials.

Authors: GF Serraino, GJ Murphy

For peer review only

Table S1. Characteristics of Included Studies.

STUDY	***Location/ Funding***	***Sample size***	***Period***	***Inclusion/ Exclusion Criteria***	***Mean Age***<br>***-intervention group***<br>***-control group***<br>***Female (%)***<br>***- Intervention group***<br>***-control group***	***NIRS Device***	***Murkin Algorithm***	***Control***	***Outcomes***
Rogers, 2017[36]	3 UK centres<br>NIHR funded	204	2009-2014	Incl: CABG and non CABG.<br>Excl: Patients undergoing emergency cardiac surgery<br>Patients who are prevented from having blood and blood products according to a system of beliefs.	70±10<br>65.9±12.3<br>30%F<br>33%F	INVOS		SC	Primary: Cognitive function<br>Secondary: Units of RBC; Cerebral oxygenation; Oxygen delivery and utilisation during CPB: Quality of life; Infectious complications; Stroke; MI; ICU and H LOS; Post-operative acute kidney injury; Respiratory complications; Health and personal social services resource use and their costs. All-cause mortality within 30 days of surgery; Biochemical markers of organ injury

For peer review only

Deschamps, 2016 [29]	Multicentre<br>Regional funders<br>Canada	201	2012 – 2013	Incl: high-risk cardiac surgery<br>Excl: Off -pump coronary artery bypass surgery, emergency surgery, planned deep hypothermic circulatory arrest, acute endocarditis, presence of active delirium or encephalopathy.	69±12.6<br>72±9.4<br>27.4%F<br>28.3%F	FORE-SIGHT, EQUANOX Classic 7600, INVOS 5100C-PB		SC	Primary Outcome: success rate of reversing cerebral desaturations below 10% relative to baseline in the intervention group<br>Secondary Outcomes: cerebral desaturation load, 30-day follow-up for adverse events
Kara, 2015 [32]	Single centre,<br>Sakarya, Turkey	79	2013-2015	Incl: CABG<br>Excl: Other procedures, high degrees of aortic atherosclerosis	59.1±9.4<br>61.2±10.3<br>23.3%F<br>19.4%F	INVOS		SC	Primary: Cognitive impairement<br>(MoCa score)<br>Secondary: ICU and H LOS
Colak, 2015[28]	Single centre, Zagreb,<br>Croatia<br>Insitutional support grant	200	2009-2010	Incl: CABG<br>Excl: carotid artery stenosis, previous stroke or head injury, seizure, psychiatric illness, (NYHA III/IV) LVEF<25%, emergency off-pump CABG and severely impaired renal and liver function.	61.9±7.1<br>63.4±8.8<br>20%F<br>24%F	INVOS		SC	Primary: Cognitive impairement<br>MMSE<br>CTT1<br>GP test<br>Secondary: coma, stupor, transient ischaemic attack (TIA) or stroke, neuropsychological deficits
Deschamps, 2013[30]	Single centre, Quebec, Canada. Insitutional support grant	49	NS	Incl: CABG and non-CABG<br>Excl: Emergency surgery, first time CABG surgery, single valve surgery	70.2±9.2<br>71.1±7.9<br>15%F<br>15%F	INVOS	X	SC	Primary: rSO2<br>Secondary: ICU and H LOS

For peer review only

Vretzakis, 2013[35]	Single centre, Greece<br>Insitutional support grant	50	16-month period	Incl: CABG and non CABG<br><br>Excl: Emergency, re-do operations, combined cardiac - carotid surgery circulatory arrest. hematologic disease coagulation abnormality, advanced cirrhosis and renal dysfunction	67.3±8.5<br>65.9±9.5<br><br>12%F<br>15%F	INVOS		SC	Primary: RBC transfusion,<br>ICU and H LOS<br>Death<br><br>Secondary: Major complications
Mohandas, 2013[33]	Single centre, Bangalore, India<br>RCT	100	NS	Incl: On pump<br>Cardiac surgery<br><br>Excl: pre-existing neuropsychiatric disorders, inability to correctly perform the neurocognitive tests, and mini-mental state examination (MMSE) scores of less than 23	38.05±15.81<br>34.60±16.28<br><br>44%F<br>44%F	NONIN EQUANOX	X	SC	Primary: Cognitive decline:<br>MMSE<br>ASEM<br><br>Secondary: ICU LOS
Harilall, 2013[31]	Single centre, South Africa	40	NS	Incl: CABG<br><br>Excl: pregnancy, history of stroke or per- sistent neurological residue, history of transient ischaemic attack (TIA), stenosis of carotid artery	55.3±9.7<br><br>30%F (all)	INVOS	X	SC	Primary: rSO2<br><br>Secondary: S100B

For peer review only

Slater, 2008[34]	Single centre, New Jersey, USA	265	2004-2006	Incl: CABG<br><br>Excl: preexisting neuropsychiatric disorders, inability to correctly perform the neurocognitive tests, and mini- mental state examination score of 23 or less.	64.8±10.1<br>64.78±9.9<br><br>31%F (all)	INVOS	X	SC	Primary: Cognitive impairement<br><br>Secondary: ICU LOS
Murkin, 2007[10]	Single centre, Ontario, Canada<br><br>Grant and other support from the device manufacturer.	200	2002 – 2004	Incl: >18 yr, CABG	61.8±10.3<br>61.8±9.3<br><br>12.5%F (all)	INVOS	X	SC	Primary: 30 days Death, MMOM<br><br>Secondary: LOS

For peer review only

Table S2. Characteristics of Excluded Studies

STUDY	*Study Type/ Location*	*SAMPLE*	*PERIOD*	*INCLUS. CRIT.*	*EXCLUS. CRIT.*	*Mean Age*	*Female (%)*	*NIRS Device*	*Murkin Algorithm*	*Control*	*OUTCOMES*
Dullenkpof, 2007 [40]	Prosp, RCT,<br>Single centreZurich, Switzerland<br>**NO intervention**	35	NS	Elective cardiac surgery with insertion of PAC	Cerebral perfusion disturbence, CAD, intracardiac shunt	65.5±10.9	74.2	INVOS			Primary: rSO2
Kok, 2014 [39]	Single centre<br>Groningen NH	59	2011 – 2012	CABG	Other procedures, difficulty completing cognitive tests, difficulty with dutch lang, impaired finction of the dominant arm or hand, history of head trauma, stroke or neurosurgery, severe CAD	62.8±9.4	10	Somanetics<br>(Michigan)<br>Casmed<br>(Branford)		On pump vs Off Pump<br>surgery	Primary: Cerebral desaturation<br>Secondary: Postop cognitive disfunction;<br>major complications;<br>ICU and Hospital LOS

PAC pulmonary artery catheter, CABG coronary artery bypass grafts, ICU intensive care unit, LOS length of stay

For peer review only

Table S3. Sub Group Analyses

SUB GROUP	CABG	NON CABG	TEST FOR SUBGROUP DIFFERENCES	MURKIN	NON MURKIN	TEST FOR SUBGROUP DIFFERENCES	CONSENSUS NEUROCOGNITIVE ASSESSMENT	NON CONSENSUS NEUROCOGNITIVE ASSESSMENT	TEST FOR SUBGROUP DIFFERENCES	RESTRICTIVE RED CELL TRANSFUSION TRIGGER	NO RESTRICTIVE RED CELL TRANSFUSION TRIGGER	TEST FOR SUBGROUP DIFFERENCES
**MORTALITY RR (95% CI)**	0.33 (0.01 to 8.09) 1 trial, n=200, I^2 =NA	0.81 (0.30 to 2.18) 3 trials, n=408, I^2=0%	**Chi^2 = 0.50, df = 3 (P = 0.92), I^2 = 0%**	0.77 (0.26 to 2.32) 2 trials, n=289, I2 =NA	0.67 (0.11 to 3.94) 2 trials, n=328, I^2=0%	**Chi^2 = 0.50, df = 3 (P = 0.92), I^2 = 0%**	0.50 (0.05 to 5.42) 1 trial, n=178 I2=NA	0.94 (0.39 to 2.26) 3 trials, n=430 I2=4%	**Chi^2 = 0.23, df = 3 (P = 0.97), I^2 = 0%**	0.67 (0.11 to 3.94) 2 trials, n=328 I2=0%	0.77 (0.26 to 2.32) 2 trials, n= 280 I2= NA	**Chi^2 = 0.50, df = 3 (P = 0.92), I^2 = 0%**
**STROKE RR (95% CI)**	1.18 (0.38 to 3.63) 3 trials, n=630. I2 =43%	0.89 (0.26 to 3.10) 4 trials, n= 508 I2=1%	**Chi^2 = 5.66, df = 6 (P = 0.46), I^2 = 0%**	0.84 (0.33 to 2.11) 5 trials, n=810 I2=14%	3.00 (0.32 to 28.54) 3 trials, n=328 I2=0%	**Chi^2 = 5.66, df = 6 (P = 0.46), I^2 = 0%**	3.00 (0.12 to 72.66) 1 trial, n=178 I2: NA	1.00 (0.39 to 2.56) 6 trials, n=960 I2=22%	**Chi^2 = 5.66, df = 6 (P = 0.46), I^2 = 0%**	3.00 (0.32 to 28.54) 2 trials, n=328 I2=0%	0.84 (0.33 to 2.11) 5 trials, n=810 I2=14%	**Chi^2 = 5.66, df = 6 (P = 0.46), I^2 = 0%**
**MI RR (95% CI)**	0.92 (0.40 to 2.10) 3 trials, n=630, I2=0%	0.75 (0.17 to 3.30) 3 trials, n=408, I2=0%	**Chi^2 = 0.05, df = 1 (P = 0.82), I^2 = 0%**	0.92 (0.40 to 2.10) 4 trials, n=710, I2=0%	0.75 (0.17 to 3.30) 2 trials, n=328 I2=0%	**Chi^2 = 0.05, df = 1 (P = 0.82), I^2 = 0%**	0.50 (0.05 to 5.42) 1 trial, n=178 I2= NA	0.93 (0.43 to 1.99) 5 trials, n=860 I2=0%	**Chi^2 = 0.24, df = 1 (P = 0.63), I^2 = 0%**	0.75 (0.17 to 3.30) 2 trials, n=328 I2=0%	0.92 (0.40 to 2.10) 4 trials, n=710 I2=0%	**Chi^2 = 0.05, df = 1 (P = 0.82), I^2 = 0%**
**SEVERE AKI RR (95% CI)**	0.58 (0.08 to 4.41) 3 trials, n=630, I2=0%	0.88 (0.51 to 1.12) 4 trials, n=434, I2=0%	**Chi^2 = 0.15, df = 1 (P = 0.70), I^2 = 0%**	0.90 (0.51 to 0.19) 4 trials, n=710 I2=0%	0.67 (0.18 to 2.56) 2 trials, n=354 I2=12%	**Chi^2 = 0.15, df = 1 (P = 0.70), I^2 = 0%**	3.00 (0.12 to 72.49) 1 trial, n=150 I2=NA	0.96 (0.55 to 1.68) 3 trials, n=780 I2=0%	**Chi^2 = 0.48, df = 1 (P = 0.49), I^2 = 0%**	0.67 (0.18 to 2.56) 2 trials, n=354 I2= 12%	0.90 (0.51 to 1.19) 2 trials, n=630 I2=0%	**Chi^2 = 0.15, df = 1 (P = 0.70), I^2 = 0%**

For peer review only - http://bmjopen.bmj.com/site/about/guidelines.xhtml

**TRANSFUSION RR (95% CI)**	1.04 (0.89 to 1.23) 2 trials, n=390. I2=0%	0.85 (0.71 to 1.01) 2 trials, n=354. I2=0%	**Chi^2 = 2.81, df = 1 (P = 0.09), I^2 = 64.4%**	1.04 (0.89 to 1.23) 2 trials, n=390. I2=0%	0.85 (0.71 to 1.01) 2 trials, n=354 I2=0%	**Chi^2 = 2.81, df = 1 (P = 0.09), I^2 = 64.4%**	0.91 (0.65 to 1.28) 1 trial, n=150. I2=NA	0.94 (0.83 to 1.06) 5 trials, n=860 I2=66%	**Chi^2 = 0.04, df = 1 (P = 0.85), I^2 = 0%**	0.85 (0.71 to 1.01) 2 trials, n=354. I2= 0%	1.04 (0.89 to 1.23) 4 trials, n=710 I2=0%	**Chi^2 = 2.81, df = 1 (P = 0.09), I^2 = 64.4%**
**ICU LOS MD (95% CI)**	-0.39 (-0.71 to - 0.06), 3 trials, n=469. I2=51%	-0.24 (-0.37 to - 0.10), 5 trials, n=582. I2=82%	**Chi^2 = 0.70, df = 1 (P = 0.4), I^2 = 0%**	-0.19 (-0.35 to - 0.03) 5 trials, n=618, I2=84%	-0.36 (-0.55 to - 0.17) 3 trials, n=433, I2=0%	**Chi^2 = 1.87, df = 1 (P = 0.17), I^2 = 46.5%**	-0.37 (-0.59 to -0.15), 1 trial, n=204 I2= NA	-0.21 (-0.50 to 0.09) 6 trials, n=747 I2=81%	**Chi^2 = 0.77, df = 1 (P = 0.38), I^2 = 0%**	-0.36 (-0.57 to - 0.14) 2 trials, n=354, I2=0%	-0.21 (-0.36 to - 0.06), 6 trials, n=697, I2=81%	**Chi^2 = 1.18 df = 1 (P = 0.28), I^2 = 14.9%**
**HOSP LOS MD (95% CI)**	-056 (-1.08 to - 0.04), 2 trials, n=279. I2=0%	-0.46 (-0.77 to - 0.15), 4 trials, n=482, I2=0%	**Chi^2 = 0.1, df = 1 (P = 0.75), I^2 = 0%**	-0.43 (-1.55 to 0.69) 3 trials, n=328. I2=0%	-0.49 (-0.77 to - 0.22) 3 trials, n=433 I2=0%	**Chi^2 = 0.01, df = 1 (P = 0.91), I^2 = 0%**	-0.50 (-0.82 to -0.18) 1 trial, n=204 I2= NA	-0.46 (0.05 to 0.93) 5 trials, n=557 I2=0%	**Chi^2 = 0.02, df = 1 (P = 0.89), I^2 = 0%**	-0.48 (-0.80 to - 0.17), 2 trials n=354, I2=0%	-0.50 (-1.00 to - 0.00) 4 trials, n=407 I2=0%	**Chi^2 = 0.00, df = 1 (P = 0.95), I^2 = 0%**

RR: Risk ratio, MD: Mean Difference, 95%CI: 95% confidence intervals, MI: Myocardial Infarction, AKI: Acute Kidney Injury, ICU: Intensive care unit, Hosp Hospital, LOS: Length of stay, CABG: coronary artery bypass grafting.

For peer review only

Figure. S1 PRISMA Flowchart of included studies

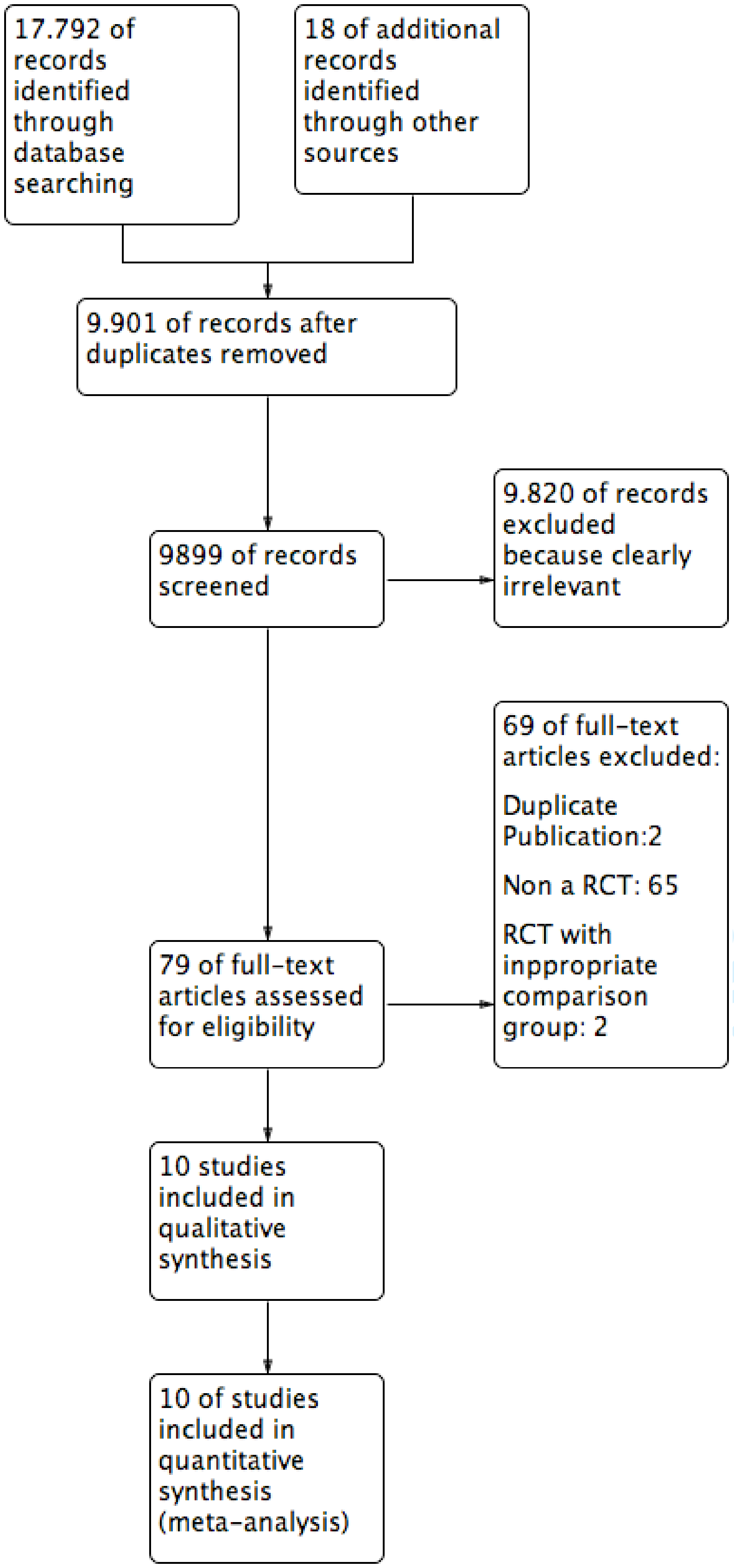

For peer review only - http://bmjopen.bmj.com/site/about/guidelines.xhtml

Figure S2: Effects of using NIRS on Bleeding, Transfusion and Time to discharge from ICU or Hospital.

RBC Transfusion

Study or Subgroup	NIRS Events	NIRS Total	Control Events	Control Total	Weight	Risk Ratio M-H, Random, 95% CI
Colak 2014	77	94	73	96	41.0%	1.08 [0.93, 1.25]
Murkin, 2007	8	100	10	100	4.0%	0.80 [0.33, 1.94]
Rogers 2017	37	98	44	106	19.3%	0.91 [0.65, 1.28]
Vretzakis 2013	51	75	63	75	35.7%	0.81 [0.67, 0.97]
**Total (95% CI)**		**367**		**377**	**100.0%**	**0.93 [0.77, 1.12]**
Total events	173		190			

Heterogeneity: $Tau^2 = 0.02$; $Chi^2 = 6.07$, df = 3 (P = 0.11); $I^2 = 51\%$
Test for overall effect: Z = 0.77 (P = 0.44)

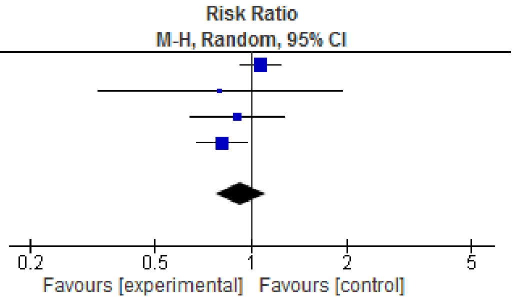

Reoperation for bleeding

Study or Subgroup	NIRS Events	NIRS Total	Control Events	Control Total	Weight	Risk Ratio M-H, Random, 95% CI
Colak 2014	0	94	1	96	9.7%	0.34 [0.01, 8.25]
Murkin, 2007	1	100	1	100	12.9%	1.00 [0.06, 15.77]
Rogers 2017	6	98	4	106	64.4%	1.62 [0.47, 5.58]
Vretzakis 2013	1	75	1	75	13.0%	1.00 [0.06, 15.69]
**Total (95% CI)**		**367**		**377**	**100.0%**	**1.23 [0.46, 3.32]**
Total events	8		7			

Heterogeneity: $Tau^2 = 0.00$; $Chi^2 = 0.86$, df = 3 (P = 0.83); $I^2 = 0\%$
Test for overall effect: Z = 0.41 (P = 0.68)

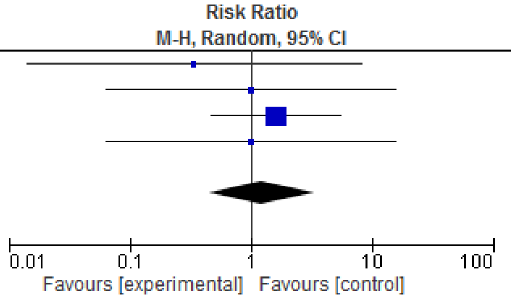

Intensive care Unit Length of Stay

Study or Subgroup	NIRS Mean	NIRS SD	NIRS Total	Control Mean	Control SD	Control Total	Weight	Mean Difference IV, Random, 95% CI
Colak 2014	2.7	6.2	94	1.9	0.9	96	5.6%	0.80 [-0.47, 2.07]
Deschamps 2013	2.99	2.26	23	0.39	2.05	25	5.9%	2.60 [1.38, 3.82]
Deschamps 2016	2.65	2.42	34	3.35	3.61	46	5.3%	-0.70 [-2.02, 0.62]
Kara, 2015	1.74	0.81	43	2.12	1.05	36	17.6%	-0.38 [-0.80, 0.04]
Mohandas 2013	1.49	0.38	50	1.7	0.49	50	22.6%	-0.21 [-0.38, -0.04]
Murkin, 2007	1.25	0.84	100	1.87	2.67	100	14.9%	-0.62 [-1.17, -0.07]
Rogers 2017	3.3	0.81	98	3.67	0.79	106	21.8%	-0.37 [-0.59, -0.15]
Vretzakis 2013	2.7	3.8	75	2.7	3.6	75	6.2%	0.00 [-1.18, 1.18]
**Total (95% CI)**			**517**			**534**	**100.0%**	**-0.13 [-0.47, 0.22]**

Heterogeneity: $Tau^2 = 0.13$; $Chi^2 = 27.52$, df = 7 (P = 0.0003); $I^2 = 75\%$
Test for overall effect: Z = 0.71 (P = 0.48)

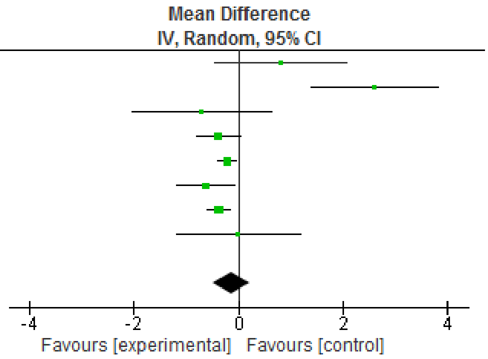

Hospital Length of Stay

Study or Subgroup	NIRS Mean	NIRS SD	NIRS Total	Control Mean	Control SD	Control Total	Weight	Mean Difference IV, Random, 95% CI
Deschamps 2013	7.6	5.4	23	7.9	3.2	25	3.2%	-0.30 [-2.84, 2.24]
Deschamps 2016	11	7.2	34	9.9	5.8	46	2.4%	1.10 [-1.84, 4.04]
Kara, 2015	7.15	1.39	43	7.67	1.14	36	66.3%	-0.52 [-1.08, 0.04]
Murkin, 2007	6.1	4.4	100	6.9	5.5	100	10.8%	-0.80 [-2.18, 0.58]
Rogers 2017	8.5	4.2	98	8.9	4.6	106	14.1%	-0.40 [-1.61, 0.81]
Vretzakis 2013	10.9	3.6	75	10.2	10.7	75	3.2%	0.70 [-1.85, 3.25]
**Total (95% CI)**			**373**			**388**	**100.0%**	**-0.45 [-0.90, 0.01]**

Heterogeneity: $Tau^2 = 0.00$; $Chi^2 = 2.17$, df = 5 (P = 0.83); $I^2 = 0\%$
Test for overall effect: Z = 1.94 (P = 0.05)

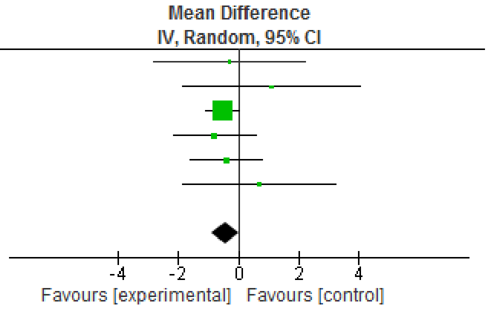

PROTOCOL INFORMATION

Protocol title: Efficacy of Near-Infrared Spectroscopy on the outcome of patients undergoing cardiac surgery: a systematic review.

For peer review only

Authors

**Prof Gavin J. Murphy**<br>British Heart Foundation<br>Professor of Cardiac Surgery<br>Department of Cardiovascular Sciences<br>University of Leicester<br>Glenfield Hospital Groby Road Leicester,<br>LE3 9QP Tel: 0116 258 3054<br>Email: gjm19@le.ac.uk	**Dr Giovanni Mariscalco**<br>Senior Lecturer<br>Department of Cardiovascular Sciences<br>University of Leicester<br>Glenfield Hospital Groby Road Leicester,<br>LE3 9QP Tel: 0116 258 3019<br>Email: gm247@leicester.ac.uk
**Dr Giuseppe Filiberto Serraino**<br>Clinical Research Fellow<br>Department of Cardiovascular Sciences University of Leicester Glenfield Hospital Groby Road Leicester,<br>LE3 9QP Email: gfs3@le.ac.uk	

Contact person: Professor Gavin J Murphy, British Heart Foundation Professor of Cardiac Surgery University of Leicester, Glenfield Hospital Groby Road Leicester, LE3 9QP. Tel: 0116 258 3054, Email: gjm19@le.ac.uk

Funding sources/sponsor

Leicester Cardiovascular Biomedical Research Unit

Dates

Anticipated or actual start date ---

Anticipated completion date ----

Type of review

Epidemiologic; Intervention

Language

English

Country

United Kingdom

Keywords

Systematic review; Cardiac Surgery; Near Infra-Red Spectroscopy; Near Infrared Oximetry; Cognitive dysfunction.

For peer review only

ABSTRACT

Background: Organ injury attributable to cardiopulmonarybypass is a major cause of morbidity, mortality and resource use in cardiac surgery patients. Regional tissue hypoxia during CPB has been implicated in perioperative organ injury. It has been suggested that goal directed patient specific optimisation of regional (brain) tissue hypoxia measured using Near Infra-Red Specroscopy (NIRS) may reduce the frequency of brain injury. Other investigators have suggested that cerebral NIRS based algorithsm may have utility for the protection of other organs, or a part of a restricitive red cell transfusion protocol.

Objectives: Our primary objective is to establish whether the use of perioperative goal directed interventions that aim to optimise cerebral Near Infrared Spectroscopy readings result in reductions in measures of cerebral injury (neurocognitive function, serum biomarkers), injury to other organs including the heart and brain, transfusion rates, mortality and resource use.

Search methods: We will conduct the search in October 2015. Potentially eligible trials will be identified by searching the Cochrane Central Register of Controlled Trials, MEDLINE , and EMBASE, using a combination of subject headings and text words to identify relevant trials. The Medline search strategy below will be adapted as appropriate for other databases.

((Cardiopulmonary Bypass) OR (Cardiac Surgery) OR (Coronary Artery Bypass) OR (Extra Corporeal Circulation)OR(Perioperative Morbidity)) AND ((Near Infra-Red Spectroscopy) OR (Oximetry) OR (Brain/Metabolism)OR(Cerebral Desaturation)OR(Cerebral Perfusion)OR(Cerebral Ischemia)OR(Cerebral Oximetry)OR(Cerebral Saturation)OR(Near Infrared Oximetry)(Cognitive)).

Selection Criteria: Two review authors will independently select references for further assessment by going through all titles and abstracts. Further selection will be based on review of full-text articles for selected references.

Data Collection and Analysis: Two review authors will independently extract study data. We will calculated the risk ratio (RR) with 95% confidence interval (CI) for binary outcomes and the mean difference (MD) or the standardised mean difference (SMD) with 95% CI for continuous outcomes. We will perform meta-analysis when possible, when I^2 is less than or equal to 80% using a fixed-effect or random-effects model, using Review Manager software. The range of point estimates for individual studies will be presented when

For peer review only

$I^2 > 80\%$. Heterogenity will be explored using subgroup analyses. Sensitivity analyses will explore the robustness of our primary analysis to exclusion of studies at high risk of bias.

Summary: We will use GRADEpro software to prepare the 'Summary of findings'table. We will judge the overall quality of the evidence foreach outcome as 'high,' 'moderate,' 'low' or 'very low' accordingto the GRADE (Grades of Recommendation, Assessment, Developmentand Evaluation) approach.

BACKGROUND

Organ injury is a common and severe complication of cardiac surgery with cardiopulmonary bypass (CPB), where it contributes to mortality, morbidity and the increased use of hospital resources [1]. In a recent trial where data on organ dysfunction was prospectively collected clinically significant kidney, lung and myocardial injury occurred in 34%, 16% an 11% of all patients and contributed to 41%, 36% and 24% of all deaths respectively [2]. The pathophysiology of CPB associated morbidity is multifactorial but is thought to involve regional hypoperfusion and tissue hypoxia [3, 4], often within vascular beds which are already abnormal due to advanced age or comorbidities such as diabetes [5, 6]. Adequate tissue oxygen delivery during CPB is achieved by optimisation of several parameters including CPB pump flows, perfusion pressure, haematocrit and the oxygen saturation of arterial blood. In contemporary clinical practice in adult cardiac surgery, the adequacy of perfusion is determined by the use of global measures of oxygen utilisation such as the mixed venous oxygen saturation (SvO2) or evidence of tissue hypoxia as implied by elevated serum lactate, a marker of cell anaerobic metabolism, or other indicators of metabolic acidosis [7]. However, in some patients, particularly those with pre-existing end organ dysfunction, global measures may not detect regional hypoxia and it has been suggested that measures of regional or tissue specific measures of oxygenation may have greater clinical utility [8-11].

The Intervention

Direct measures of regional tissue oxygen levels such as gastric tonometry, laser Doppler flowmetry of the intestinal mucosa, or cerebral venous oxygen saturation using jugular bulb catheters, can be used to measure and optimise tissue oxygenation but these modalities are invasive, which limits their use. More

For peer review only

recently, Near Infra-red Spectroscopy (NIRS) has emerged as a technique to monitor regional tissue oxygenation non-invasively and accurately with the added benefit that it can accurately measure tissue oxygenation in one of the most important end organs, the brain. NIRS sensors, when applied to the forehead, can determine the relative saturation / desaturation of blood within the cerebral arterioles and venules of the forebrain. Sas the total volume of blood is domintaed by th enemous compartment this measure is considered to accurately reflect cerebral venous oxygen saturation [12, 13] and is now approved as a non-invasive measure of regional cerebral oxygenation by the Medicines and Healthcare Products Regulatory Agency (MHRA). Early clinical studies suggested that the use of this technoliogy to optimise cerebral oxygenation during CPB have shown that optimisation of cerebral oxygenation using NIRS can be associated not only with reduced neurological morbidity but also with a reduction in renal complications and other major adverse clinical events [14-16]. However in the absence of high quality evidence there is uncertainty as to the efficacy or cost effectiveness of this device and there is wide variation in its use [ ].

Indications for red blood cell transfusion during cardiac surgery

Cerebral oximetry may also be used during CPB to develop goal-directed, patient-specific indicators of the need for red blood cell (RBC) transfusion if used as part of a wider algorithm designed to optimise cerebral oxygenation. The primary goal of RBC transfusion is to optimise tissue oxygenation. Currently, most RBC transfusions are given solely as a response to a haematocrit that has fallen below an arbitrary threshold and not as a response to incipient tissue hypoxia [18, 19]. The haematocrit is a poor indicator of regional tissue hypoxia and, therefore, is inadequate as an indicator of the need for transfusion.The haematocrit below which oxygen delivery to tissues is reduced such that anaerobic metabolism occurs is known as the critical Haematocrit (Hcrit). In healthy human adults very low haematocrits of less than 15 can cause organ hypoxia [9, 11] and transfusion in such cases is thought to be beneficial [20, 21]. The Hcrit for patients during cardiac surgery, however, is unclear. It is thought to be higher than 15, as most patients are elderly and have comorbid conditions, but it is also likely that the Hcrit varies considerably both between cardiac patients as well as for individual patients over the course of the perioperative period [22]. Hcrit is increased by conditions which impair autoregulation such as diabetes or increased age [5, 6] and during CPB, Hcrit is

For peer review only

also affected by multiple factors that affect the balance of oxygen supply and oxygen demand such as hypothermia, re-warming, pump flow or perfusion pressure [23]. Uncertainty about when an individual may benefit from a transfusion is reflected in the wide range in reported haematocrit transfusion thresholds used during CPB (from 17-25) [24-26] as well as the wide variation in transfusion rates in cardiac surgical patients across units in the United Kingdom (UK) (35-75%) [27]. Because the Hcrit for any particular patient at any given time is unknown, the ability to directly measure tissue oxygenation and therefore incipient tissue hypoxia may have distinct advantages over the use of generic and pre-specified haematocrit transfusion thresholds.

How the intervention might work

Existing protocols to optimise tissue oxygenation during CPB deploy manipulations of CPB (according to levels of blood markers such as lactate or pH) and the transfusion of RBC (according to the haematocrit). These methods are often treated independently of each other, and in the case of trandsfusion commonly guided by protocolised thresholds, despite the fact that both have the underlying aim of improving tissue oxygenation [7].

In contrast the patient-specific algorithm differs from the standard care in two key ways. First it is 'goal-directed', in that the algorithm is specifically targeted to maintain cerebral oxygen delivery during CPB (monitored by NIRS), a measure of regional rather than global tissue hypoxia. Second it allows the manipulation of multiple variables to optimise regional oxygenation for individual patients. This represents a form of personalised medicine. The most widely cited patient-specific algorithm developed by Murkin and colleagues [28] aims to optimise the cerebral oxygen supply / demand balance during CPB by: (a) increasing oxygen supply using hyperoxygenation, increased pump flow, perfusion pressure or hypercapnic cerebral vasodilation, (b) Increasing oxygen offloading by the use of nitrates, or (c) reducing oxygen demand by deepening anaesthesia. Cerebral oxygen saturations approaching a low threshold in the presence of anaemia (a haematocrit of between 18 and 23) and despite optimisation of other parameters suggest that the cerebral Hcrit is about to be reached and transfusion is indicated. Therefore, this algorithm is patient and time specific and goal-directed to optimise a validated objective measure of tissue oxygenation. This

For peer review only

should potentially reduce healthcare costs associated with complications attributable to tissue hypoxia during CPB or to unnecessary allogeneic RBC transfusions.

Why is it important to do this review

Numerous studies have reported the incidence of postoperative neurocognitive decline around 50% [29, 30]. Central venous oxygen desauration is common following cardiac surgery and this has been implicated in cognitive decline [3, 4]. Multiple studies have evaluated the efficacy of NIRS based algorithms that allow optimisation of cerebral saturation. Although some of these studies have been reported as positive [8] overall these results of these studies have been have been inconclusive, and no recent systematic review has attempted to summarise the available evidence. The use of this technology is variable and there is evidence of equipoise as to its effectiveness. To resolve this uncertainty we propose to undertake a systematic review and meta-analysis of the available evidence from RCTs.

OBJECTIVES

(1) To establish whether the use of perioperative goal directed interventions that aim to optimise cerebral Near Infrared Spectroscopy readings result in reductions in measures of cerebral injury (neuroscogitive function, serum biomarkers), morbidity, mortality and resource use.

(2) Does the use of perioperative goal directed interventions that aim to optimise cerebral Near Infrared Spectroscopy readings result in reductions in red cell transfusion, non red cell transfusion, or bleeding.

METHODS

Types of Studies

Randomized controlled trials irrespective of blinding, language, publication status, date of publication and sample size.

Types of Participants

Patients undergoing cardiac surgery for acquired or congenital disease, or aortovascular disease with or without cardiopulmonary bypass. No age restriction will be applied. There are no exclusion criteria.

For peer review only

Types of Interventions

Intervention: Goal directed NIRS algorithm.

Comparator /control: An untread group, or alternative (non NIRS based) goal directed therapy.

Types of Outcome Measure

Primary outcomes:

Mortality: 30 day or hospital all-cause mortality.

Secondary outcomes:

1. Acute brain injury; stroke, TIA as defined by study authors.
2. Low cardiac output as defined by study authors.
3. Myocardial Infarction as defined by study authors.
4. Acute kidney Injury Stage 3 [32] or requiring haemofiltration as defined by study authors.
5. Neurocognitive function; group means as described by neurocognitive tests. Tests recommended by a consensus statement to test all key domains of cognitive function [33] are marked with *. Studies will be categorised as Yes/ No as to whether they have assessed the key domains described in the Consensus Statement. Key domians [ ] that may be assessed are as follows
   - Attention: Sustained and divided attention: Consensus statements reccomend the Trail-Making Test parts A* and B* [35, 36].
   - Verbal memory: Consensus statements reccomend the RAVLT and Rey Auditory Verbal Learning Test* [36]
   - Visuo-spatial: as the Block Design from the Wechsler Adult Intelligence Scale [37]
   - Psychomotor speed: Consensus statements reccomend tests such the Digit Symbol Test from the Wechsler Adult Intelligence Scale [37]
   - Executive function/Verbal fluency: Consensus statements reccomend tests such the Controlled Oral Word Association Test [38]
   - Motor coordination: Consensus statements reccomend tests such the Grooved Pegboard Test*, dominant and non-dominant hand [33].
6. Neurocognitive dysfunction, as a dichotomous outcome reported by the study authors; A consensus definition is a change in a single test of >1 SD. This may be defined as change in a group mean (adjusted for baseline) or for individual patients [39]. Studies will be categorised as defining cognitive dysfunction using a Consensus versus a non-Consensus definitions.
7. Risk of receiving blood transfusion as defined by study authors.
8. Reoperation for bleeding as defined by study authors.
9. Resoiurce Use: ICU and hospital LOS as defined by study authors.
10. S100B levels as reported by study authors.

Search methods for identification of studies

For peer review only

Potentially eligible trials will be identified by searching the Cochrane Central Register of Controlled Trials, MEDLINE , and EMBASE, using a combination of subject headings and text words to identify relevant trials. The Medline search strategy below will be adapted as appropriate for other databases.

((Cardiopulmonary Bypass) OR (Cardiac Surgery) OR (Coronary Artery Bypass) OR (Extra Corporeal Circulation)OR(Perioperative Morbidity)) AND ((Near Infra-Red Spectroscopy) OR (Oximetry) OR (Brain/Metabolism)OR(Cerebral Desaturation)OR(Cerebral Perfusion)OR(Cerebral Ischemia)OR(Cerebral Oximetry)OR(Cerebral Saturation)OR(Near Infrared Oximetry)(Cognitive)).

To identify ongoing or unpublished trials we will also search the Clinicaltrials.gov using the following search terms:

Search terms: Randomized

Study Type: Interventional Studies

Conditions: Cardiac surgery OR Cardiopulmonary bypass

Interventions: Near Infra-Red Spectroscopy OR Near Infrared Oximetry OR Cerebral Desauturation

We will also examined the reference lists of eligible trials and reviews. Searches will not be restricted by language or publication status.

Data Collection and Analysis

The review will be performed in accordance with instructions given in the Cochrane Handbook for Systematic Reviews of Interventions (Higgins 2011).

Selection of Studies

Two reviewers GJM, GFS will identify trials for inclusion independently of each other. Exluded studies and the reason for exclusion will be recorded.

Data extraction (selection and coding)

Two authors will independently screen the search output to identify records of potentially eligible trials examining the outcomes, the full texts of which will be retrieved and assessed for inclusion.

A standardised form will be used to extract data from the included studies for assessment of study quality and evidence synthesis. Extracted information will include:

- Year and language of publication

For peer review only

- Country of Particiapnt recruitment
- Year of conduct of the trial
- Study setting; university teaching hospital, non universityteaching hospital
- Study population; inclusion and exlusion criteria
- Sample size
- Participant demographics
- Baseline characteristics
- Type of surgery
- Details of NIRS algorithm (Murkin, non Murkin [41])
- Deatails of Comparator; non NIRS goal directed therapy, standard care (protocolised care)
- Outcomes and times of measurement
- Information for assessment of the risk of bias.

Two review authors will extract data independently, discrepancies will be identified and resolved through discussion (with a third author where necessary). Missing data will be requested from study authors. If there is doubt as to whether trials share participants completely or partially (with common authors and centres) we will contact the study authors to ascertain whether the studt report has been duplicated.

Risk of Bias

The following bias risk domains will be assessed as Low, Uncertain, or High based on the instructions given in the in the Cochrane Handbook for Systematic Reviews of Interventions [42];

- Sequence Generation
- Allocation Concealment
- Blinding of participant, personnel
- Blinding of outcome Assessors
- Incomplete outcome data
- Selective outcome reporting
- Source of funding bias

Trials will be classified as having a low risk of bias if they are graded as being at low risk of bias in all of these domains.

Two review authors will independently assess the risk of bias in all of the studies. Discrepancies will be resolved by discussion with a third author.

Assessment of Reporting Bias

Publication bias will be assessed by the visual assessment of funnel plots and Eggers test [44].

Measures of treatment Effect

For dichotomous variables, we will calculate the risk ratio (RR) with 95% confidence interval (CI). For continuous variables, we will calculate the mean difference (MD) with 95% CI for outcomes such as hospital

For peer review only

stay, and standardised mean difference (SMD) with 95% CI for quality of life (when different scales were used).

Dealing with missing data

We will perform an intention-to-treat analysis where possible. For dichotomous data presented only as percentages we will estimate frequencies using reported sample sizes for this outcome. For continuous outcomes if the mean and the standard deviation were not available from the trial report, we will seek this information from the trial authors. If this information is still not available, we will calculate the mean and standard deviation from median (interquartile ranges) using the software available in Review Manager Version 5.

Assessment of Heterogeneity

We anticipate that major sources of clinical heterogeneity will be associated with different patient groups (adults, children, congenital versus acquired disease), the use of different goal directed NIRS algorithms, the use of co-interventions such as restrictive transfusion thresholds, and differences in the methodology used to assess neurocognitive dysfunction. We will explore heterogeneity within each meta-analysis using a Chi^2 test with significance set at a P value of 0.10, and we expressed the percentage of heterogeneity due to variation rather than to chance as I^2 [45].We defined heterogeneity as follows:

I^2 0-40%: no or mild heterogeneity

I^2 40-80%: moderate heterogeneity

I^2 > 80%: sever heterogeneity

In the presence of severe heterogeneity meta-analysis will not be performed.

Data Synthesis

Meta-analyses will be performed using the software package Review Manager version 5.2 and in accordance with the recommendations of the *Cochrane Handbook for Systematic Reviews of Intervention* [46].

For the primary analysis we will compare the results of a random-effects model versus a fixed effects model to assess the effects of small studies. For continuous outcomes, we will pool mean differences or

For peer review only

standardised mean differences by using the inverse variance method. We will use the generic inverse variance method to pool hazard ratios.

Subgroup Analyses

Subgroup analyses will be performed in trials in which the Murkin algorithm [41] was used to guide goal directed therapy versus those that did not, and by participant group; CABG versus non CABG, adults versus children, assessment of neurocognitive function that incorporates test described in a previous consensus statement, and studies that combined the NIRS algorithm with a restrictive red cell transfusion trigger. Test for sub-group differences with Review Manager will be used with a P value of <0.05 considered statistically significant.

Sensitivity Analyses

Sensitivity analysis will exclude trials with unclear or high risk of bias for random sequence generation; unclear or high risk of bias due to lack of blinding of participants, healthcare providers or outcome assessors, and unclear or high risk of bias due to incomplete outcome data, and unclear of high risk of bias for source of funder.

Summary of findings

We will presentthe main results of the review in a 'Summary of findings' table. We will include the follwing outcomes.

- Risk of mortality
- Risk of stroke, myocardial infarction, or severe acute kidney injury
- Risk of red cell transfusion
- Neurocognitive Impairment
- Resource Use; ICU and hospital length of stay.

We will use GRADEpro software to prepare the 'Summary of findings'table. We will judge the overall quality of the evidence foreach outcome as 'high,' 'moderate,' 'low' or 'very low' accordingto the GRADE (Grades of Recommendation, Assessment, Developmentand Evaluation) approach [46].We will consider the following.

- Impact of risk of bias of individual trials.
- Precision of pooled estimate.Inconsistency or heterogeneity (clinical, methodological and statistical).

For peer review only

- Indirectness of evidence.
- Impact of selective reporting and publication bias on effect estimate.

- **COMPETING INTERESTS**

The authors declare that they have no competing interests.

AUTHORS' CONTRIBUTIONS

___ had full access to all of the data in the study and take responsibility for the integrity of the data and the accuracy of the data analysis.

Study concept and design: ___.

Acquisition of data: ___.

Analysis and interpretation of data: ______.

Drafting of the manuscript: ________.

Statistical analysis: _______

Study supervision: GJM.

For peer review only

REFERENCES

1. Murphy, G J and G D Angelini. Side effects of cardiopulmonary bypass: what is the reality? J Card Surg, 2004. 19(6): p. 481-488.
2. Murphy GJ,
3. Sapire, K J, S P Gopinath, G Farhat, D R Thakar, A Gabrielli, J W Jones, C S Robertson, and B Chance. Cerebral oxygenation during warming after cardiopulmonary bypass. Crit Care Med, 1997. 25(10): p. 1655-1662.
4. Yao, F S, C C Tseng, C Y Ho, S K Levin, and P Illner. Cerebral oxygen desaturation is associated with early postoperative neuropsychological dysfunction in patients undergoing cardiac surgery. J Cardiothorac Vasc Anesth, 2004. 18(5): p. 552-558.
5. Croughwell, N, M Lyth, T J Quill, M Newman, W J Greeley, L R Smith, and J G Reves. Diabetic patients have abnormal cerebral autoregulation during cardiopulmonary bypass. Circulation, 1990. 82(5 Suppl): p. IV407-412.
6. Miyoshi, S, T Morita, Y Kadoi, and F Goto. Analysis of the factors related to a decrease in jugular venous oxygen saturation in patients with diabetes mellitus during normothermic cardiopulmonary bypass. Surg Today, 2005. 35(7): p. 530-534.
7. Monitoring during CPB
8. Haisjackl, M, G Luz, H Sparr, R Germann, N Salak, B Friesenecker, E Deusch, S Meusburger, and W Hasibeder. The effects of progressive anemia on jejunal mucosal and serosal tissue oxygenation in pigs. Anesth Analg, 1997. 84(3): p. 538-544.
9. Leung, J M, R B Weiskopf, J Feiner, H W Hopf, S Kelley, M Viele, J Lieberman, J Watson, M Noorani, D Pastor, H Yeap, R Ho, and P Toy. Electrocardiographic ST-segment changes during acute, severe isovolemic hemodilution in humans. Anesthesiology, 2000. 93(4): p. 1004-1010.
10. Tsai, A G, P Cabrales, and M Intaglietta. Microvascular perfusion upon exchange transfusion with stored red blood cells in normovolemic anemic conditions. Transfusion, 2004. 44(11): p. 1626-1634.
11. Weiskopf, R B, J H Kramer, M Viele, M Neumann, J R Feiner, J J Watson, H W Hopf, and P Toy. Acute severe isovolemic anemia impairs cognitive function and memory in humans. Anesthesiology, 2000. 92(6): p. 1646-1652.
12. Kim, M B, D S Ward, C R Cartwright, J Kolano, S Chlebowski, and L C Henson. Estimation of jugular venous O2 saturation from cerebral oximetry or arterial O2 saturation during isocapnic hypoxia. J Clin Monit Comput, 2000. 16(3): p. 191-199.
13. Pollard, V, D S Prough, A E DeMelo, D J Deyo, T Uchida, and H F Stoddart. Validation in volunteers of a near-infrared spectroscope for monitoring brain oxygenation in vivo. Anesth Analg, 1996. 82(2): p. 269-277.
14. Alexander J.C.Jr, K M A, & Dance G.R. Reduced postoperative length of stay may result from using cerebral oximetry monitoring to guide treatment. The Annals of Thoracic Surgery, 2002. 73(1): p. S373.
15. Goldman, S, F Sutter, F Ferdinand, and C Trace. Optimizing intraoperative cerebral oxygen delivery using noninvasive cerebral oximetry decreases the incidence of stroke for cardiac surgical patients. Heart Surg Forum, 2004. 7(5): p. E376-381.
16. Murkin, J M, S J Adams, R J Novick, M Quantz, D Bainbridge, I Iglesias, A Cleland, B Schaefer, B Irwin, and S Fox. Monitoring brain oxygen saturation during coronary bypass surgery: a randomized, prospective study. Anesth Analg, 2007. 104(1): p. 51-58.
17. Variability Paper
18. Vincent, J L, J F Baron, K Reinhart, L Gattinoni, L Thijs, A Webb, A Meier-Hellmann, G Nollet, D Peres-Bota, and A B C Investigators. Anemia and blood transfusion in critically ill patients. JAMA, 2002. 288(12): p. 1499-1507.
19. Walsh, T S, M Garrioch, C Maciver, R J Lee, F MacKirdy, D B McClelland, J Kinsella, C Wallis, and G Audit of Transfusion in Intensive Care in Scotland Study. Red cell requirements for intensive care units adhering to evidence-based transfusion guidelines. Transfusion, 2004. 44(10): p. 1405-1411.

For peer review only

20. Casutt, M, B Seifert, T Pasch, E R Schmid, M I Turina, and D R Spahn. Factors influencing the individual effects of blood transfusions on oxygen delivery and oxygen consumption. Crit Care Med, 1999. 27(10): p. 2194-2200.
21. Weiskopf, R B, J Feiner, H Hopf, J Lieberman, H E Finlay, C Quah, J H Kramer, A Bostrom, and P Toy. Fresh blood and aged stored blood are equally efficacious in immediately reversing anemia-induced brain oxygenation deficits in humans. Anesthesiology, 2006. 104(5): p. 911-920.
22. Utoh, J, S Moriyama, K Okamoto, R Kunitomo, M Hara, and N Kitamura. The effects of cardiopulmonary bypass on postoperative oxygen metabolism. Surg Today, 1999. 29(1): p. 28-33.
23. Shann KG, Likosky DS, Murkin JM, Baker RA, Baribeau YR, DeFoe GR, Dickinson TA, Gardner TJ, Grocott HP, O'Connor GT, Rosinski DJ, Sellke FW, Willcox TW. An evidence-based review of the practice of cardiopulmonary bypass in adults: a focus on neurologic injury, glycemic control, hemodilution, and the inflammatory response. J Thorac Cardiovasc Surg. 2006 Aug;132(2):283-90.
24. DeFoe, G R, C S Ross, E M Olmstead, S D Surgenor, M P Fillinger, R C Groom, R J Forest, J W Pieroni, C S Warren, M E Bogosian, C F Krumholz, C Clark, R A Clough, P W Weldner, S J Lahey, B J Leavitt, C A Marrin, D C Charlesworth, P Marshall, and G T O'Connor. Lowest hematocrit on bypass and adverse outcomes associated with coronary artery bypass grafting. Northern New England Cardiovascular Disease Study Group. Ann Thorac Surg, 2001. 71(3): p. 769-776.
25. Fang, W C, R E Helm, K H Krieger, T K Rosengart, W J DuBois, C Sason, M L Lesser, O W Isom, and J P Gold. Impact of minimum hematocrit during cardiopulmonary bypass on mortality in patients undergoing coronary artery surgery. Circulation, 1997. 96(9 Suppl): p. II-194-199.
26. Von Heymann, C, M Sander, A Foer, A Heinemann, B Spiess, J Braun, M Kramer, J Grosse, P Dohmen, S Dushe, J Halle, W F Konertz, K D Wernecke, and C Spies. The impact of an hematocrit of 20% during normothermic cardiopulmonary bypass for elective low risk coronary artery bypass graft surgery on oxygen delivery and clinical outcome--a randomized controlled study. Crit Care, 2006. 10(2): p. R58.
27. Murpjhy GJ Audit
28. Denault A, Deschamps A, Murkin JM. A proposed algorithm for the intraoperative use of cerebral near-infrared spectroscopy. Semin Cardiothorac Vasc Anesth. 2007 Dec;11(4):274-81
29. Newman MF, Kirchner JL, Phillips-Bute, et al. Longitudinal assessment of neurocognitive function after coronary artery bypass surgery. New Engl J Med 2001;344:395–402.
30. Selnes OA, Goldsborough MA, Borowicz LM Jr, Enger C, Quaskey SA, McKhann GM. Determinants of cognitive change after coronary artery bypass graft surgery: a multi- factorial problem. Ann Thorac Surg 1999;67:1669 –76.
31. NIRS AKI
32. Mehta, R L, J A Kellum, S V Shah, B A Molitoris, C Ronco, D G Warnock, A Levin, and N Acute Kidney Injury. Acute Kidney Injury Network: report of an initiative to improve outcomes in acute kidney injury. Crit Care, 2007. 11(2): p. R31.
33. Murkin, J M, S P Newman, D A Stump, and J A Blumenthal. Statement of consensus on assessment of neurobehavioral outcomes after cardiac surgery. Ann Thorac Surg, 1995. 59(5): p. 1289-1295.
34. Mahanna, E P, J A Blumenthal, W D White, N D Croughwell, C P Clancy, L R Smith, and M F Newman. Defining neuropsychological dysfunction after coronary artery bypass grafting. Ann Thorac Surg, 1996. 61(5): p. 1342-1347.
35. SP Newman. Analysis and interpretation of neuropsychologic tests in cardiac surgery Ann Thorac Surg, 59 (1995), pp. 1351–1355
36. Spreen O., S, E. A compendium of neuropsychological tests. 1998, New York: Oxford University Press
37. Wechsler, D. Manual of the Wechsler Test of Adult Reading (WTAR-UK). 2001, New York Psychological Corporation (Iglesias)
38. Benton, A H, R. Multilingual Aphasia Examination. 1976, Iowa City: University of Iow
39. Murkin JM, Stump DA, Blumenthal JA, McKhann G. Defining dysfunction: group means versus incidence analysis-a statement of consensus. Ann Thorac Surg. 1997 Sep;64(3):904-5.
40. Higgins JPT, Deeks JJ. Chapter 7: Selecting studies and collecting data In: Higgins JPT,Green S (editors). Cochrane Handbook for Systematic Reviews of Interventions Version 5.1.0 [updated March 2011]. The Cochrane Collaboration, 2011. www.cochrane-handbook.org.

For peer review only

41. Denault A, Deschamps A, Murkin JM. A proposed algorithm for the intraoperative use of cerebral near-infrared spectroscopy. Semin Cardiothorac Vasc Anesth. 2007 Dec;11(4):274-81
42. Higgins JPT, Altman DG, Sterne JAC. Chapter 8: Assessing risk of bias in included studies. Cochrane Handbook for Systematic Reviews of Interventions Version 5.1.0 [updated March 2011]. The Cochrane Collaboration, 2011. www.cochrane-handbook.org.
43. Sterne JAC, Egger M, Moher D. Chapter 10: Addressing reporting biases In: Higgins JPT, Green S (editors). Cochrane Handbook for Systematic Reviews of Interventions Version 5.1.0 [updated March 2011]. The Cochrane Collaboration, 2011. www.cochranehandbook.org.
44. Higgins JPT, Thompson SG. Quantifying heterogeneity in a meta-analysis. Statistics in Medicine 2002;21(11):1539–58.
45. Higgins JPT, Green S (editors). Cochrane Handbook for Systematic Reviews of Interventions Version 5.1.0 [updated March 2011]. The Cochrane Colloboration, 2011. www.cochrane-handbook.org.
46. Schünemann HJ, Oxman AD, Vist GE, Higgins JPT, Deeks JJ, Glasziou P, et al.Chapter 12: Interpreting results and drawing conclusions. Higgins JPT, Green S(editors). Cochrane Handbook for Systematic Reviewsof Interventions Version 5.1.0 [updated March 2011].The Cochrane Collaboration, 2011. www.cochrane-handbook.org.

For peer review only

PRISMA Checklist:Efficacy of Near-Infrared Spectroscopy on the outcome of patients undergoing cardiac surgery: a systematic review of randomised trials.

Authors: GF Serraino, GJ Murphy

Section/topic	#	Checklist item	Reported on page #
**TITLE**			
Title	1	Identify the report as a systematic review, meta-analysis, or both.	1
Structured summary	2	Provide a structured summary including, as applicable: background; objectives; data sources; study eligibility criteria, participants, and interventions; study appraisal and synthesis methods; results; limitations; conclusions and implications of key findings; systematic review registration number.	2
Rationale	3	Describe the rationale for the review in the context of what is already known.	4
Objectives	4	Provide an explicit statement of questions being addressed with reference to participants, interventions, comparisons, outcomes, and study design (PICOS).	4
Protocol and registration	5	Indicate if a review protocol exists, if and where it can be accessed (e.g., Web address), and, if available, provide registration information including registration number.	4
Eligibility criteria	6	Specify study characteristics (e.g., PICOS, length of follow-up) and report characteristics (e.g., years considered, language, publication status) used as criteria for eligibility, giving rationale.	5
Information sources	7	Describe all information sources (e.g., databases with dates of coverage, contact with study authors to identify additional studies) in the search and date last	4

For peer review only

Section/topic	#	Checklist item	Reported on page #
		searched.	
Search	8	Present full electronic search strategy for at least one database, including any limits used, such that it could be repeated.	4
Study selection	9	State the process for selecting studies (i.e., screening, eligibility, included in systematic review, and, if applicable, included in the meta-analysis).	5
Data collection process	10	Describe method of data extraction from reports (e.g., piloted forms, independently, in duplicate) and any processes for obtaining and confirming data from investigators.	5
Data items	11	List and define all variables for which data were sought (e.g., PICOS, funding sources) and any assumptions and simplifications made.	5
Risk of bias in individual studies	12	Describe methods used for assessing risk of bias of individual studies (including specification of whether this was done at the study or outcome level), and how this information is to be used in any data synthesis.	5
Summary measures	13	State the principal summary measures (e.g., risk ratio, difference in means).	5
Synthesis of results	14	Describe the methods of handling data and combining results of studies, if done, including measures of consistency (e.g., I^2) for each meta-analysis.	5
Risk of bias across studies	15	Specify any assessment of risk of bias that may affect the cumulative evidence (e.g., publication bias, selective reporting within studies).	5
Additional analyses	16	Describe methods of additional analyses (e.g., sensitivity or subgroup analyses, meta-regression), if done, indicating which were pre-specified.	6
Study selection	17	Give numbers of studies screened, assessed for eligibility, and included in the review, with reasons for exclusions at each stage, ideally with a flow diagram.	6

For peer review only

Section/topic	#	Checklist item	Reported on page #
Study characteristics	18	For each study, present characteristics for which data were extracted (e.g., study size, PICOS, follow-up period) and provide the citations.	6
Risk of bias within studies	19	Present data on risk of bias of each study and, if available, any outcome-level assessment (see Item 12).	8
Results of individual studies	20	For all outcomes considered (benefits or harms), present, for each study: (a) simple summary data for each intervention group and (b) effect estimates and confidence intervals, ideally with a forest plot.	9
Synthesis of results	21	Present results of each meta-analysis done, including confidence intervals and measures of consistency.	10
Risk of bias across studies	22	Present results of any assessment of risk of bias across studies (see Item 15).	11
Additional analysis	23	Give results of additional analyses, if done (e.g., sensitivity or subgroup analyses, meta-regression [see Item 16]).	11
Summary of evidence	24	Summarize the main findings including the strength of evidence for each main outcome; consider their relevance to key groups (e.g., health care providers, users, and policy makers).	12
Limitations	25	Discuss limitations at study and outcome level (e.g., risk of bias), and at review level (e.g., incomplete retrieval of identified research, reporting bias).	13
Conclusions	26	Provide a general interpretation of the results in the context of other evidence, and implications for future research.	14
Funding	27	Describe sources of funding for the systematic review and other support (e.g.,	15

For peer review only

Section/topic	#	Checklist item	Reported on page #
		supply of data); role of funders for the systematic review.	

For peer review only

BMJ Open

Effects of cerebral Near-Infrared Spectroscopy on the outcome of patients undergoing cardiac surgery: a systematic review of randomised trials.

Journal:	*BMJ Open*
Manuscript ID	bmjopen-2017-016613.R2
Article Type:	Research
Date Submitted by the Author:	22-Jun-2017
Complete List of Authors:	Serraino, Giuseppe; University of Leicester College of Medicine Biological Sciences and Psychology, Cardiovascular Sciences<br>Murphy, Gavin; University of Leicester College of Medicine Biological Sciences and Psychology, Cardiovascular Sciences
**Primary Subject Heading**:	Cardiovascular medicine
Secondary Subject Heading:	Surgery, Anaesthesia, Neurology
Keywords:	Cardiopulmonary bypass, cerebral oxygenation, near Infra-red spectroscopy

SCHOLARONE™
Manuscripts

For peer review only

Title: Effects of cerebral Near-Infrared Spectroscopy on the outcome of patients undergoing cardiac surgery: a systematic review of randomised trials.

Authors: Giuseppe F Serraino, Gavin J Murphy

Authors Affiliation: Department of Cardiovascular Sciences and NIHR Leicester Biomedical Research Unit in Cardiovascular Medicine, University of Leicester, Clinical Sciences Wing, Glenfield General Hospital, Leicester, LE3 9QP

Correspondence to: Professor Gavin Murphy, Department of Cardiovascular Sciences and NIHR Leicester Biomedical Research Unit in Cardiovascular Medicine, University of Leicester, Clinical Sciences Wing, Glenfield General Hospital, Leicester, LE3 9QP. Email: gjm19@le.ac.uk Tel: +44 116 2583021, Fax: +44 116 2583054.

Total word count: 4795

Keywords: Cardiopulmonary bypass, cerebral oxygenation, near Infra-red spectroscopy.

For peer review only

Abstract

Objectives: Goal directed optimisation of cerebral oxygenation using Near Infra-red Spectroscopy (NIRS) during cardiopulmonary bypass is widely used. We tested the hypotheses that the use of NIRS cerebral oximetry results in reductions in cerebral injury (neurocognitive function, serum biomarkers), injury to other organs including the heart and brain, transfusion rates, mortality and resource use.

Design: Systematic review and meta-analysis.

Setting: Tertiary cardiac surgery centres in North America, Europe and Asia.

Participants: A search of Cochrane Central Register of Controlled Trials, Clinical Trials.gov, MEDLINE, and EMBASE and the Cumulative Index to Nursing and Allied Health Literature (CINAHL) Plus from inception to November 2016 identified 10 randomised trials, enrolling a total of 1466 patients, all in adult cardiac surgery.

Interventions: NIRS based algorithms designed to optimise cerebral oxygenation versus standard care (non NIRS based) protocols in cardiac surgery patients during cardiopulmonary bypass.

Outcome Measures: Mortality, organ injury affecting the brain, heart and kidneys, red cell transfusion and resource use.

Results: Two of the 10 trials identified in the literature search were considered at low risk of bias. Random effects meta-analysis demonstrated similar mortality (Risk Ratio (RR) 0.76, 95% Confidence Intervals (CI) 0.30 to 1.96), major morbidity including stroke (RR 1. 08, 95% CI 0.40 to 2.91), red cell transfusion, and resource use, in NIRS treated patients and controls, with little or no heterogeneity. Grades of Recommendation, Assessment, Development and Evaluation (GRADE) assessment of the quality of the evidence was Low or Very Low for all of the outcomes assessed.

Conclusions: The results of this systematic review did not support the hypotheses that cerebral NIRS based algorithms have clinical benefits in cardiac surgery.

Registration: PROSPERO CRD42015027696

For peer review only

For peer review only - http://bmjopen.bmj.com/site/about/guidelines.xhtml

Strengths and limitations of the Study

- We performed a systematic review of randomised trials evaluating the clinical benefits of near infrared spectroscopy based cerebral oximetry monitoring during cardiopulmonary bypass.
- This is most comprehensive review of this technology to date.
- All of the existing trials had important methodological limitations, including importantly a lack of blinding of clinical personnel.
- GRADE assessment of the quality of the evidence was Low or Very Low for all of the outcomes assessed indicating a high likelihood that the findings of the review may be altered by subsequent trials.

For peer review only

For peer review only - http://bmjopen.bmj.com/site/about/guidelines.xhtml

Introduction

Brain injury is a common and severe complication of cardiac surgery with cardiopulmonary bypass (CPB), affecting up to 40% of patients, where it contributes to morbidity, mortality, and the increased use of hospital resources [1]. The pathophysiology of CPB associated brain injury is multifactorial [2] but is thought to involve regional hypoperfusion and tissue hypoxia,[3,4] often within vascular beds which are already abnormal due to advanced age or comorbidities such as diabetes.[5,6] Previous studies have suggested that cerebral oxygenation may be measured non-invasively using Near Infra-red Spectroscopy (NIRS) sensors applied to the forehead,[7] and more importantly that targeted interventions during bypass that increase NIRS measured cerebral oxygenation have clinical benefits including reductions in brain injury,[8] and reductions in injury to other organ systems as a result of improved overall perfusion.[9,10] It has also been suggested that the use of NIRS may allow the safe application of restrictive red cell transfusion thresholds where there is evidence of adequate cerebral oxygenation; a putative personalised transfusion indicator.[7,11] However, there is uncertainty as to the clinical benefits of NIRS[12] and this leads to variability in the use of these devices.[13] To address this uncertainty we performed a systematic review and meta-analysis of randomised trials that had evaluated the effects of personalised NIRS based algorithms on clinical outcomes including mortality, organ (brain, heart, kidney) injury, transfusion and resource use.

Methods

A systematic review was performed using the methods described in Cochrane Handbook for Systematic Reviews of Interventions.[14] The review protocol was registered prospectively; PROSPERO CRD42015027696.

Types of Studies

Randomized controlled trials irrespective of blinding, language, publication status, date of publication and sample size.

For peer review only - http://bmjopen.bmj.com/site/about/guidelines.xhtml

Types of Participants

Patients undergoing cardiac surgery for acquired or congenital disease or aortovascular disease with or without cardiopulmonary bypass were included. No age restriction was applied. There were no exclusion criteria.

Types of Interventions

Intervention: Goal directed NIRS algorithm during cardiac surgery with cardiopulmonary bypass.

Comparator /control: An untreated group, or alternative (non NIRS based) goal directed therapy.

Types of Outcome Measure

Primary outcomes:

Mortality: 30 day or hospital all-cause mortality.

Secondary outcomes:

1. Acute brain injury; stroke, TIA as defined by study authors.
2. Low cardiac output as defined by study authors.
3. Myocardial Infarction as defined by study authors.
4. Acute kidney Injury Stage 3 or requiring hemofiltration as defined by study authors.
5. Neurocognitive function; group means as described by neurocognitive tests. Tests recommended by a consensus statement to test all key domains of cognitive function [15] are marked with *. Studies were categorised as Yes/ No as to whether they had assessed the key domains described in the Consensus Statement. Key domains that were assessed [16] and examples of likely tests that were recorded are as follows:
    - Attention: Sustained and divided attention: Consensus statements recommend the Trail-Making Test parts A* and B*.[17,18]
    - Verbal memory: Consensus statements recommend the RAVLT and Rey Auditory Verbal Learning Test*.[18]
    - Visual-spatial: as the Block Design from the Wechsler Adult Intelligence Scale.[19]

For peer review only - http://bmjopen.bmj.com/site/about/guidelines.xhtml

- Psychomotor speed: Consensus statements recommend tests such the Digit Symbol Test from the Wechsler Adult Intelligence Scale.[19]
- Executive function/Verbal fluency: Consensus statements recommend tests such the Controlled Oral Word Association Test.[20]
- Motor coordination: Consensus statements recommend tests such the Grooved Pegboard Test*, dominant and non-dominant hand.[17]
- Frontal Lobe: Saccadic and anti-saccadic eye movements.

Assessment of important covariates (Mini Mental State, Anxiety and Depression, Intellectual Ability, and Concomitant medication) in these analyses were documented.

6. Neurocognitive dysfunction, as reported by the study authors; A consensus definition is a change in a single test of >1 SD. This was defined as either change >1 SD in a group mean (adjusted for baseline) or a change in neurocognitive scores of >1 SD for individual patients.[21] Studies were further categorised as defining cognitive dysfunction using a Consensus versus a non-Consensus definitions.
7. Risk of receiving blood transfusion as defined by study authors.
8. Reoperation for bleeding as defined by study authors.
9. Resource Use: ICU and hospital LOS as defined by study authors.
10. S100B levels, a marker of brain injury, as reported by study authors.

Search methods for identification of studies

Potentially eligible trials were identified by searching the *Cochrane Central Register of Controlled Trials*, *MEDLINE* , and *EMBASE* and the *Cumulative Index to Nursing and Allied Health Literature (CINAHL) Plus*, using a combination of subject headings and text words to identify relevant trials. The search was performed from Inception until November 2016. The Medline search strategy below was adapted as appropriate for other databases:

For peer review only - http://bmjopen.bmj.com/site/about/guidelines.xhtml

((Cardiopulmonary Bypass) OR (Cardiac Surgery) OR (Coronary Artery Bypass) OR (Extra Corporeal Circulation) OR (Perioperative Morbidity)) AND ((Near Infra-Red Spectroscopy) OR (Oximetry) OR (Brain/Metabolism) OR (Cerebral Desaturation) OR (Cerebral Perfusion) OR (Cerebral Ischemia) OR (Cerebral Oximetry) OR (Cerebral Saturation) OR (Near Infrared Oximetry) OR (Cognitive)).

To identify on going or unpublished trials we also searched the Clinicaltrials.gov using the following search terms:

Search terms: Randomized

Study Type: Interventional Studies

Conditions: ((Cardiopulmonary Bypass) OR (Cardiac Surgery) OR (Coronary Artery Bypass) OR (Extra Corporeal Circulation) OR (Perioperative Morbidity))

Interventions: ((Near Infra-Red Spectroscopy) OR (Oximetry) OR (Brain/Metabolism) OR (Cerebral Desaturation) OR (Cerebral Perfusion) OR (Cerebral Ischemia) OR (Cerebral Oximetry) OR (Cerebral Saturation) OR (Near Infrared Oximetry) OR (Cognitive))

We will also examined the reference lists of eligible trials and reviews. Searches were not restricted by language or publication status.

Data Collection and Analysis

The review was performed in accordance with instructions given in the Cochrane Handbook for Systematic Reviews of Interventions.[22]

Selection of Studies

Two reviewers GJM and GFS identified trials for inclusion independently of each other. Excluded studies and the reason for exclusion were recorded.

Data extraction (selection and coding)

Two authors independently screened the search output to identify records of potentially eligible trials examining the outcomes, the full texts of which were retrieved and assessed for inclusion.

For peer review only - http://bmjopen.bmj.com/site/about/guidelines.xhtml

A standardised form was used to extract data from the included studies for assessment of study quality and evidence synthesis. Extracted information included:

- Year and language of publication
- Country of Participant recruitment
- Year of conduct of the trial
- Study setting; university teaching hospital, non-university teaching hospital
- Study population; inclusion and exclusion criteria
- Sample size
- Participant demographics
- Baseline characteristics
- Type of surgery
- Details of NIRS algorithm (Murkin, non Murkin[23]) and co-interventions (restrictive versus non-restrictive transfusion thresholds)
- Details of Comparator; non NIRS goal directed therapy, standard care (protocolised care)
- Outcomes and times of measurement

Two review authors extracted data independently; discrepancies were identified and resolved through discussion. Missing data was requested from study authors. If there is doubt as to whether trials shared participants completely or partially (with common authors and centres) we contacted the study authors to ascertain whether the study report has been duplicated.

Risk of Bias

The following bias risk domains were assessed as Low, Uncertain, or High based on the instructions given in the in the Cochrane Handbook for Systematic Reviews of Interventions;[24]

- Sequence Generation
- Allocation Concealment
- Blinding of participant, personnel

For peer review only - http://bmjopen.bmj.com/site/about/guidelines.xhtml

- Blinding of outcome Assessors
- Incomplete outcome data
- Selective outcome reporting
- Source of funding bias

Trials were classified as having a low risk of bias if they are graded as being at low risk of bias in all of these domains. Two review authors independently assessed the risk of bias in all of the studies. Discrepancies were resolved by discussion.

Assessment of Reporting Bias

Where 10 or more studies were identified for each outcome we assessed publication bias by the visual assessment of funnel plots and Eggers test.[25]

Measures of treatment Effect

For dichotomous variables, we calculated the risk ratio (RR) with 95% confidence interval (CI). For continuous variables, we calculated the mean difference (MD) with 95% CI for outcomes such as hospital stay, and standardised mean difference (SMD) with 95% CI for quality of life (when different scales were used).

Dealing with missing data

We perform an intention-to-treat analysis where possible. For dichotomous data presented only as percentages we estimated frequencies using reported sample sizes for this outcome. For continuous outcomes if the mean and the standard deviation were not available from the trial report, we sought this information from the trial authors. If this information was still not available, we calculated the mean and standard deviation from median (interquartile ranges) using the software available in Review Manager Version 5.

Assessment of Heterogeneity

We anticipated that major sources of clinical heterogeneity would associated with different patient groups (adults, children, congenital versus acquired disease), the use of different goal directed NIRS algorithms,

For peer review only - http://bmjopen.bmj.com/site/about/guidelines.xhtml

For peer review only

the use of co-interventions such as restrictive transfusion thresholds, and differences in the methodology used to assess neurocognitive dysfunction. We explored heterogeneity within each meta-analysis using a Chi^2 test with significance set at a P value of 0.10, and expressed the percentage of heterogeneity due to variation rather than to chance as I^2.[26] We defined heterogeneity as follows:

I^2 0-40%: no or mild heterogeneity

I^2 40-80%: moderate heterogeneity

I^2 > 80%: severe heterogeneity

In the presence of severe heterogeneity meta-analysis was not performed.

Data Synthesis

Meta-analyses was performed using the software package Review Manager version 5.2 and in accordance with the recommendations of the *Cochrane Handbook for Systematic Reviews of Intervention.*[14] For the primary analysis we compared the results of a random-effects model versus a fixed effects model to assess the effects of small studies. For continuous outcomes, pooled mean differences or standardised mean differences by using the inverse variance method.

Subgroup Analyses

Subgroup analyses was performed in trials in which the Murkin algorithm was used to guide goal directed therapy versus those that did not, and by participant group; CABG versus non CABG, adults versus children, assessment of neurocognitive function that incorporated tests described in a previous consensus statement, and studies that combined the NIRS algorithm with a restrictive red cell transfusion trigger. Test for sub-group differences with Review Manager was used with a P value of <0.05 considered statistically significant.[27]

Sensitivity Analyses

Sensitivity analysis excluded trials with high risk of bias for any of the following: random sequence generation; allocation concealment, blinding of participants, healthcare providers or outcome assessors, incomplete outcome data, attrition, and other sources of bias including source of funder.

For peer review only - http://bmjopen.bmj.com/site/about/guidelines.xhtml

For peer review only

Summary of findings

We presented the main results of the review in a 'Summary of findings' table. We included the following outcomes.

- Risk of mortality
- Risk of stroke, myocardial infarction, or severe acute kidney injury
- Risk of red cell transfusion
- Neurocognitive Impairment
- Resource Use: ICU and Hospital length of stay.

We used GRADEpro software to prepare the 'Summary of findings' table. We judged the overall quality of the evidence for each outcome as 'high,' 'moderate,' 'low' or 'very low' according to the GRADE (Grades of Recommendation, Assessment, Development and Evaluation) approach.[27] We considered the following.

- Impact of risk of bias of individual trials.
- Precision of pooled estimate. Inconsistency or heterogeneity (clinical, methodological and statistical).
- Indirectness of evidence.
- Impact of selective reporting and publication bias on effect estimate.

Results

Description of Studies

Search Results: We identified a total of 17792 references through electronic searches of the Cochrane Central Register of Controlled Trials (CENTRAL) (n=1347), MEDLINE (n=9924), EMBASE (n=6159), and the Cumulative Index to Nursing and Allied Health Literature (CINAHL) Plus (n=362). From ClinicalTrials.gov, we identified 18 trials that potentially met the inclusion criteria. We have shown the flow of search results in Figure 1. We excluded 7909 duplicates, then 9820 clearly irrelevant references, by reading titles and abstracts. No additional references were identified by reference searching. Of 79 study reports retrieved in

For peer review only - http://bmjopen.bmj.com/site/about/guidelines.xhtml

full text, we excluded 69 references for the reasons listed in Figure S1 in the online only digital supplement. In total, 11 publications describing 9 randomised controlled trials fulfilled the inclusion and exclusion criteria provided quantitative data for this review [10,28-35] (Table S1 in the online only digital supplement). We also included data from a recent trial undertaken by our own unit, the results of which are in press.[36]

Description of excluded studies: Four trials that met our inclusion criteria were excluded after review of the full manuscript (Table S2 in the online only digital supplement). There were two additional reports from one trial; an interim analysis,[37] and a post hoc analysis,[38] that was also reported in full.[10] Another trial compared NIRS values in on- versus of-pump CABG patients.[39] A fourth trial by Dullenkopf and colleagues[40] was reported in the abstract as an RCT, however the randomisation of placing the NIRS sensors on either the right or left for head and there was no clinical NIRS intervention versus a control comparison.

Included studies: Ten trials that evaluated NIRS based algorithms in patients undergoing cardiac surgery were identified.[10,28-35]

Participants: Overall, 1466 participants took part in the 10 trials included in the review. The average age of participants in these trials ranged from 34.6 ± 16.3 to 71 ± 11.2 years and the proportion of females ranged from 12.5% to 66% in the trials that provided this information.[10,28-35] The proportion of post randomisation withdrawals ranged from 0% to 4.11%. After withdrawal occurred, 1452 participants were included in the quantitative meta-analyses in this systematic review. Five trials were conducted in patients undergoing CABG only,[10,28,31,32,34] and 5 trials were conducted in patients undergoing valve or CABG and valve surgery or other cardiac surgical procedure.[29,30,33,35,36] Further details of participants are listed in Table S1 in the online only digital supplement.

Intervention: Cerebral NIRS values were measured with the INVOS (Somanetics Corporation, Troy, MI) in 9 trials. In 1 trial[29] three different devices were used; the INVOS, the FORE-SIGHT (CAS Medical Systems Inc., USA), and the EQUANOX Classic 7600 (Nonin Medical Inc., USA). In 2 trials the target NIRS values were >75% of baseline.[10,31] In 4 trials the targets values were >80%.[29,30,32,34] In 1 trial the target NIRS values were

For peer review only - http://bmjopen.bmj.com/site/about/guidelines.xhtml

For peer review only

greater than an absolute NIRS value of 60% or >20% compared to the mean value during pulmonary artery catheter insertion.[35] In 2 trials the target NIRS value was a combination of >80% of baseline or an absolute measure > 50%.[28,33] In 1 trial the target regional oxygen saturation values were specified as >70% of pre-induction values or an absolute value of >50%.[36] Seven trials used a version of the Murkin algorithm to optimise NIRS values.[10,28-31,33,34,36] Two studies used non-Murkin algorithms.[32,35]

Control: All trials used standard (protocolised) care as the control group. In 7 trials NIRS values were measured in the control group, although the values were hidden from the clinical staff.[10,29-31,33,34,36] No trial considered an alternative patient specific goal directed algorithm.

Co-interventions: In 2 trials the Murkin algorithm was combined with a restrictive transfusion trigger.[35,36] In these studies a pre-specified objective was to determine whether NIRS could be utilised as part of a patient specific red cell transfusion indicator.

Risk of bias in included studies

Risk of bias in individual trials is shown in Figure 2A, and the proportions of trials with low risk, unclear risk and high risk of bias in each of the domains are shown in Figure 2B. Clinical personnel were not blinded in any trial. However, 2 trials were considered at low risk of bias in every domain.[35,36]

Sequence generation: Random sequence generation was adequate in 7 trials,[10,28,29,31,34-36] and unclear in 2 trials.[30,33] There was a high risk of bias for random sequence generation in 1 trial.[32]

Allocation: Allocation concealment was adequate in 7 trials,[10,28,29,31,34-36] and unclear in 2 trials.[30,33] There was a high risk of bias for random sequence generation in 1 trial.[32]

Blinding: Theatre staff were unblinded to group allocation in all of the studies. There was evidence of blinding of patients and clinical staff caring for patients postoperatively in 4 trials,[30,32,35,36] and unclear evidence in 6 trials.[10,28,29,31,33,34] Two trials reported the frequency of protocol compliance.[35,36] In 8 trials non-compliance was not monitored or not specified. There was evidence of blinding of outcome assessors

For peer review only

in 5 trials [10,28,32,35,36] and unclear evidence of blinding of outcome assessors in 5 trials.[29-31,33,34]

Incomplete outcome data: 6 trials reported completeness of follow-up for the primary outcome.[10,29,31,32,35,36] Of these, 5 trials reported <10% loss to follow-up, and 1 trial reported >10% loss to follow-up.[35] Four trials that failed to report completeness of follow-up were considered to be at high risk of attrition bias.[28,30,33,34]

Selective reporting: Only 1 of the 10 trials included in this review had a published trial protocol.[36] Another 5 trials had reported details of primary and secondary outcomes in trial registries.[28-30,34,35] Of these, 3 trials failed to report all the pre specified outcomes.[28,30,34] Of the remaining four trials, none were registered or had published protocols, and were considered at high risk of selective outcome reporting bias.

Source of funding bias: Sources of funding were reported in 6 trials. Of these 2 trials were funded by independent sources. In 1 trial the study was supported in part by the NIRS device manufacturer and was therefore considered at high risk of funding bias.[10] Three studies that failed to report the source of funding were considered at uncertain risk of funding bias (Table 1).

Effects of intervention

The summary effect estimates for primary and secondary outcomes are described in Table 1, Figure 2, and Figure S2 in the online only digital supplement.

Mortality: Four trials with 608 participants reported this outcome.[10,30,35,36] There was no statistically significant difference in mortality between NIRS versus controls; RR 0.76, 95% CI 0.30 to 1.96; I^2=0%; Chi^2 test for heterogeneity P value 0.92.

Stroke: Seven trials with 1138 participants reported this outcome.[10,28,29,33-36] There was no statistically significant difference in the frequency of stroke between NIRS versus controls; RR 1. 08, 95% CI 0.40 to 2.91; I2=0%; Chi2 test for heterogeneity P value 0.46.

For peer review only

Myocardial infarction: Six trials with 1038 participants reported this outcome.[10,28,29,34-36] There was no statistically significant difference in the frequency of myocardial infarction between NIRS versus controls; RR 0.90, 95% CI 0.43 to 1.89; I2=0%; Chi2 test for heterogeneity P value 0.86.

Severe Acute Kidney injury: Six trials with 1064 participants reported this outcome.[10,28,30,34-36] There was no statistically significant difference in the frequency of severe acute kidney injury between NIRS versus controls; RR 0.88, 95% CI 0.58 to 1.34; I2=0%; Chi2 test for heterogeneity P value 0.89.

Red cell transfusion: Four trials with 744 participants reported this outcome.[10,28,35,36] There was no statistically significant difference in the frequency of red cell transfusion between NIRS versus controls; RR 0.93, 95% CI 0.77 to 1.12; I2=51%; Chi2 test for heterogeneity P value 0.11.

Reoperation for Bleeding: Four trials with 744 participants reported this outcome.[10,28,35,36] There was no difference in the frequency of reoperation for bleeding between NIRS versus controls; RR 1.11, 95% CI 0.41 to 3.04; I2=0%; Chi2 test for heterogeneity P value 0.69.

Neurocognitive testing and measuring neurocognitive dysfunction: Five trials that recruited 813 patients reported this outcome.[28,32-34,36] One trial[29] reported only the incidence of delirium. Details of these trials are listed in Table 2. The Consensus Statement for the Assessment of Neurocognitive function in Cardiac Surgery[15] recommends that that the following Core tests be performed at baseline and up to 3 months post-surgery; the Rey auditory verbal learning test, the Trail-Making test Part A and Trail-Making Test Part B, and the Grooved Pegboard test to assess the neurocognitive domains Attention, Verbal Memory and Motor Coordination. The Consensus Statement defines cognitive decline as a difference for the individual of >1SD from baseline, or a difference of >1SD between group means, with adjustment for baseline for at least one test. There was significant heterogeneity for this outcome. Only 2 trials[34,36] measured cognitive function as recommended by the consensus statement. Both trials reported no difference between the groups for neurocognitive function. In 1 trial no test data was presented.[34] In the other[36] there was a significant difference between the groups for the Controlled Oral Word Association Test, which assesses

For peer review only

For peer review only - http://bmjopen.bmj.com/site/about/guidelines.xhtml

the domain Executive Function/ Verbal Fluency, however this is not a specified core domain in the consensus statement. The three remaining trials used non-consensus testing protocols and non-consensus definitions of neurocognitive decline, and only 1 tested patients at 3 months post-surgery. Because of the degree of heterogeneity we did not perform meta-analyses of these outcome.

ICU LOS: Eight trials with 1051 participants reported this outcome.[10,28-30,32,33,35,36] There were no statistically significant differences in the duration of ICU stay between NIRS versus controls with moderate heterogeneity; RR MD 0.00, 95% CI -0.44 to 0.44; I^2=73%; Chi2 test for heterogeneity P value 0.0005.

Hospital LOS: Six trials with 761 participants reported this outcome.[10,29,30,32,35,36] Hospital LOS was less in the NIRS group however this was not statistically significant; RR MD -0.45, 95% CI -0.90 to 0.01; I^2=0%; Chi2 test for heterogeneity P value 0.83.

S100B: Two trials reported this outcome in 138 patients.[31,36] One trial with 40 participants measured S100B pre- and postoperatively and reported a significant reduction in S100B in NIRS patients, MD -99.87, 95% CI -105.18 to -94.56. The time of the post-surgery sample was not reported. Another with 98 participants measured S100B preoperatively and at 4 post-surgery time points; on return to ICU, and at 6, 12-24, 24-48 and 96 hours. There was no difference between the groups; Ratio of Geometric Means 1.06, 95% CI (0.95, 1.19), p=0.29. No meta-analysis was performed due to the heterogeneity for this outcome.

Subgroup analyses

Results of these subgroup analyses for Murkin vs non- Murkin algorithms, CABG vs non CABG, and assessment of neurocognitive function using tests described in a previous consensus statement are shown in Table S3 in the online only digital supplement.

Publication Bias: A funnel plot of standard error versus risk ratio for the included outcomes showed an asymmetrical distribution that indicated publication bias. However, since there was an insufficient number of trials providing data (less than 10 studies identified for each outcome), we did not perform this analysis.

For peer review only - http://bmjopen.bmj.com/site/about/guidelines.xhtml

Sensitivity Analyses

Fixed effects models did not materially change the results of our primary analyses. We also conducted sensitivity analyses in two trials identified as being at low risk of bias.[35,36] These trials reported outcomes in 328 participants. Both trials incorporated restrictive red cell transfusion thresholds within the NIRS algorithm. They reported no difference between NIRS and control groups for mortality: RR 0.67, 95% CI 0.11 to 4.08; I2=0%; Chi2 test for heterogeneity P value 0.71; stroke: RR 3.0 95% CI -0.32to 28.54; I2=0%; Chi2 test for heterogeneity P value 1.00; myocardial infarction: RR 0.76, 95% CI 0.17 to 3.41; I2=0%; Chi2 test for heterogeneity P value 0.66; severe acute kidney injury: RR 0.83, 95% CI 0.44 to 1.54; I2=0%; Chi2 test for heterogeneity P value 0.42; reoperation for bleeding: RR 1.50, 95% CI 0.48 to 4.62; I2=0%, Chi2 test for heterogeneity P value 0.75; ICU length of stay: RR-0.03, 95% CI -0.69 to 0.62; I2=0%; Chi2 test for heterogeneity P value 0.95 and hospital length of stay: RR -0.20, 95% CI -1.29 to 0.89; I^2=0%; Chi^2 test for heterogeneity P value 0.45. Analyses of these trials suggested that the use of NIRS based algorithms resulted in reductions in red cell transfusion: RR 0.83, 95% CI 0.71 to 0.98; I2=0%; Chi2 test for heterogeneity P value 0.52, One of these trials, the PASPORT trial [36] reported the results of neurocognitive assessments and concluded that there was no difference between NIRS and control groups.

GRADE Assessment

A summary of the main findings of the review are presented in Table 3. GRADE assessments of the results were either Low or Very Low for all the outcomes, indicating a high likelihood that these conclusions may be altered by subsequent trials.

For peer review only

For peer review only - http://bmjopen.bmj.com/site/about/guidelines.xhtml

Discussion

Main Findings

A systematic review and meta-analysis of existing trials did not demonstrate clinical benefits attributable to the use of personalised NIRS based algorithms during CPB. The use of NIRS did not result in reductions in mortality, injury to the brain, heart or kidneys, or reductions in resource use. A qualitative review of studies that had evaluated the effects of NIRS on neurocognitive function did not show clear evidence of benefit. An analysis of two trials at low risk of bias where NIRS was applied along with a restrictive red cell transfusion threshold demonstrated a reduction in red cell transfusion with this approach, with no difference between NIRS treated and controls with respect to clinical outcomes or resource use. Overall the GRADE quality of the evidence was low or very low for all of the outcomes measured.

Strengths and weaknesses

This is the most comprehensive evaluation of NIRS based patient specific goal direct algorithms in cardiac surgery to date. The results supersede those of a previous quantitative review of NIRS in cardiac surgery that considered randomised (2 studies) and observational analyses (27 studies).[41] Our searches also identified a Cochrane review protocol with similar aims however the results of this review have yet to be published.[42] The current review used comprehensive search strategies in a wide range of registries and data sources, had access to the full texts of all identified trials, used contemporary risk of bias assessments (GRADE), and assessed a wide range of outcomes after cardiac surgery. The main limitation of the review is that we did not have access to all of the source data. Although additional unpublished information was also obtained from three authors and included in the meta-analysis we realise that consistent analyses of all studies can only be done when data on individual patients are combined. In addition, the review identified important limitations of existing data; all of the 10 RCTs had limitations in terms of methodological quality. The risk of procedural bias was high in these trials as there was no blinding of clinical personnel. Furthermore only two from 10 trials attempted to define the likelihood of procedural bias by describing the degree of protocol adherence. The reporting of outcomes was also heterogeneous between trials limiting

For peer review only

the number of studies that could be included in each outcome. This was most evident for the outcome cognitive function. Many trials did not report important clinical outcomes; such as death, although it is highly likely that this outcome was measured. Furthermore, in many cases although the trial report stated that important assessments had been made, particularly with respect to the testing of cognitive function, the results of these assessments were not reported. Despite these limitations the results were remarkably consistent, with low or no heterogeneity for all of the analyses, all of which suggested that there are no clinical benefits attributable to the use of NIRS based algorithms.

Clinical Importance

NIRS technology and NIRS based algorithms are used in cardiac surgery centres worldwide, although there is clear evidence of equipoise with respect to the clinical benefits this technology.[12,13] The results presented here do not support the hypotheses that the use of NIRS may reduce brain injury,[8] or by using the brain as the index organ, reducing injury to the heart or kidneys as the result of improved overall perfusion during cardiac surgery with cardiopulmonary bypass.[9] They do not provide insights into the role of NIRS in procedures that require deep hypothermic circulatory arrest. It is possible that our results are attributable to chance; our GRADE assessment of the systematic review results was very low for all the pre-specified outcomes, indicating a high likelihood that these conclusions may be altered by subsequent trials. There was almost no heterogeneity for these outcomes, however, and these findings were consistent with the results of the PASPORT trial.[36] This multicentre trial recruited a larger cohort than almost all the previous trials, was at low risk of bias, and demonstrated no benefit for NIRS based algorithms for a range of outcomes including cognitive function, and biomarkers of myocardial, renal and neurological injury.[36]

Our systematic review indicated that the combination of NIRS and a restrictive transfusion threshold resulted in a reduction in red cell transfusion, without any difference between NIRS treated patients and controls with respect to clinical outcomes or resource use. This may be interpreted as showing that NIRS may be used safely to implement restrictive transfusion thresholds. These findings must be interpreted

For peer review only - http://bmjopen.bmj.com/site/about/guidelines.xhtml

with caution however, these 2 trials enrolled only 387 patients, clinical staff were not blinded to group allocation in these trials, and endpoints such as red cell transfusion are susceptible to performance bias. A final consideration is that the review did not identify any trial in paediatric cardiac surgery, a common setting for the use of these devices, and thus has identified a knowledge gap with respect to the utility of this intervention in these patients.

Conclusions

Existing evidence suggests that the use of NIRS based patient specific algorithms that aim to optimise cerebral oximetry do not result in reductions in mortality, major morbidity or resource use in adult cardiac surgery. Assessment of the quality of the evidence indicates that there is a need for further randomised trials at low risk of bias to assess the clinical utility of NIRS in both adult and paediatric cardiac surgery. To determine the clinical utility of these devices future studies should be designed to evaluate pragmatic, clinically important outcomes such as freedom from death and disability, or major morbidity (stroke, renal failure requiring dialysis, low cardiac output).

Sources of Funding

This work was supported by the National Institute for Health Research Programme Grants for Applied Health number RP-PG-0407-10384. GFS is supported by the Leicester NIHR Cardiovascular Biomedical Research Unit. GJM is supported by British Heart Foundation Grants RG/13/6/29947 and CH/12/1/29419.

Disclosures

Both authors have completed the Unified Competing Interest form at www.icmje.org/coi_disclosure.pdf (available on request from the corresponding author). Dr Serraino declares no conflict of interest. Professor Murphy reports grants from British Heart Foundation during the conduct of the study and grants from British Heart Foundation, grants from National Institute for Health Research, grants from Zimmer Biomet, personal fees from Abbvie, and personal fees from Thrasos Inc, outside the submitted work.

For peer review only

Details of Contributors

Both of the study authors, external and internal, had full access to all of the data (including statistical reports and tables) in the study and can take responsibility for the integrity of the data and the accuracy of the data analysis. Murphy conceived and designed the study, and undertook the systematic review. Serraino wrote the protocol, undertook the systematic review, performed the analysis and drafted the report along with Murphy. Both authors reviewed the report for important intellectual content and approved the final version.

Acknowledgements

The authors gratefully acknowledge the assistance of the Mr Giovanni Mariscalco who reviewed the study protocol for important intellectual content.

Data Sharing

No additional data available.

For peer review only

For peer review only - http://bmjopen.bmj.com/site/about/guidelines.xhtml

References:

1. Selnes OA, Gottesman RF, Grega MA, Baumgartner WA, Zeger SL, McKhann GM. Cognitive and neurologic outcomes after coronary-artery bypass surgery. N Engl J Med 2012; 366: 250-7
2. Murphy GJ, Angelini GD. Side effects of cardiopulmonary bypass: what is the reality? J Card Surg 2004; 19: 481-8
3. Kim MB, Ward DS, Cartwright CR, Kolano J, Chlebowski S, Henson LC. Estimation of jugular venous O2 saturation from cerebral oximetry or arterial O2 saturation during isocapnic hypoxia. J Clin Monit Comput 2000; 16: 191-9
4. Sapire KJ, Gopinath SP, Farhat G, et al. Cerebral oxygenation during warming after cardiopulmonary bypass. Crit Care Med 1997; 25: 1655-62
5. Croughwell N, Lyth M, Quill TJ, et al. Diabetic patients have abnormal cerebral autoregulation during cardiopulmonary bypass. Circulation 1990; 82: IV407-12
6. Miyoshi S, Morita T, Kadoi Y, Goto F. Analysis of the factors related to a decrease in jugular venous oxygen saturation in patients with diabetes mellitus during normothermic cardiopulmonary bypass. Surg Today 2005; 35: 530-4
7. Vretzakis G, Georgopoulou S, Stamoulis K, et al. Cerebral oximetry in cardiac anesthesia. J Thorac Dis 2014; 6 Suppl 1: S60-9
8. Goldman S, Sutter F, Ferdinand F, Trace C. Optimizing intraoperative cerebral oxygen delivery using noninvasive cerebral oximetry decreases the incidence of stroke for cardiac surgical patients. Heart Surg Forum 2004; 7: E376-81
9. Murkin JM. Cerebral oximetry: monitoring the brain as the index organ. Anesthesiology 2011; 114: 12-3
10. Murkin JM, Adams SJ, Novick RJ, et al. Monitoring brain oxygen saturation during coronary bypass surgery: a randomized, prospective study. Anesth Analg 2007; 104: 51-8
11. Klein HG, Flegel WA, Natanson C. Red Blood Cell Transfusion: Precision vs Imprecision Medicine. JAMA 2015; 314: 1557-8
12. Kane JM, Steinhorn DM. Lack of irrefutable validation does not negate clinical utility of near-infrared spectroscopy monitoring: learning to trust new technology. J Crit Care 2009; 24: 472 e1-7
13. Zacharias DG, Lilly K, Shaw CL, et al. Survey of the clinical assessment and utility of near-infrared cerebral oximetry in cardiac surgery. J Cardiothorac Vasc Anesth 2014; 28: 308-16
14. Higgins JPT, Green, S. Cochrane Handbook for Systematic Reviews of Interventions Version 5.1.0, The Cochrane Collaboration, 2011
15. Murkin JM, Newman SP, Stump DA, Blumenthal JA. Statement of consensus on assessment of neurobehavioral outcomes after cardiac surgery. Ann Thorac Surg 1995; 59: 1289-95
16. Mahanna EP, Blumenthal JA, White WD, et al. Defining neuropsychological dysfunction after coronary artery bypass grafting. Ann Thorac Surg 1996; 61: 1342-7
17. Newman SP. Analysis and interpretation of neuropsychologic tests in cardiac surgery. Ann Thorac Surg 1995; 59: 1351-5
18. Spreen OSE. A compendium of neuropsychological tests, A compendium of neuropsychological tests. Edited by OSE Spreen. New York, Oxford University Press, 1998
19. Weschler D. Manual of the Wechsler Test of Adult Reading (WTAR-UK), Manual of the Wechsler Test of Adult Reading (WTAR-UK). Edited by D W. New York, Psychological Corporation (Iglesias), 2001
20. Benton AHR. Multilingual Aphasia Examination, Multilingual Aphasia Examination. Edited by AHR B. Iowa City, University of Iowa, 1976
21. Murkin JM, Stump DA, Blumenthal JA, McKhann G. Defining dysfunction: group means versus incidence analysis--a statement of consensus. Ann Thorac Surg 1997; 64: 904-5
22. Higgins JPT Deeks JJ. Selecting studies and collecting data Cochrane Handbook for Systematic Reviews of Interventions Version 5.1.0. Edited by Higgins JPT Green S, The Cochrane Collaboration, 2011

For peer review only - http://bmjopen.bmj.com/site/about/guidelines.xhtml

23. Denault A, Deschamps A, Murkin JM. A proposed algorithm for the intraoperative use of cerebral near-infrared spectroscopy. Semin Cardiothorac Vasc Anesth 2007; 11: 274-81
24. Higgins JPT Altman DG, Sterne JAC: Assessing risk of bias in included studies, Cochrane Handbook for Systematic Reviews of Interventions Version 5.1.0. Edited by Higgins JPT Green S, The Cochrane Collaboration, 2011
25. Sterne JAC Egger M, Moher D. Addressing reporting biases Cochrane Handbook for Systematic Reviews of Interventions Version 5.1.0. Edited by Higgins JPT Green S, The Cochrane Collaboration, 2011
26. Higgins JP, Thompson SG. Quantifying heterogeneity in a meta-analysis. Stat Med 2002; 21: 1539-58
27. Schünemann HJ Oxman AD, Vist GE, Higgins JPT, Deeks JJ, Glasziou P. Interpreting results and drawing conclusions, Cochrane Handbook for Systematic Reviewsof Interventions Version 5.1.0 Edited by Higgins JPT Green S, The Cochrane Collaboration, 2011
28. Colak Z, Borojevic M, Bogovic A, Ivancan V, Biocina B, Majeric-Kogler V. Influence of intraoperative cerebral oximetry monitoring on neurocognitive function after coronary artery bypass surgery: a randomized, prospective study. Eur J Cardiothorac Surg 2015; 47: 447-54
29. Deschamps A, Hall R, Grocott H, et al. Cerebral Oximetry Monitoring to Maintain Normal Cerebral Oxygen Saturation during High-risk Cardiac Surgery: A Randomized Controlled Feasibility Trial. Anesthesiology 2016; 124: 826-36
30. Deschamps A, Lambert J, Couture P, et al. Reversal of decreases in cerebral saturation in high-risk cardiac surgery. J Cardiothorac Vasc Anesth 2013; 27: 1260-6
31. Harilall Y, Adam JK, Biccard BM, Reddi A. The effect of optimising cerebral tissue oxygen saturation on markers of neurological injury during coronary artery bypass graft surgery. Heart Lung Circ 2014; 23: 68-74
32. Kara I, Erkin A, Sacli H, et al. The Effects of Near-Infrared Spectroscopy on the Neurocognitive Functions in the Patients Undergoing Coronary Artery Bypass Grafting with Asymptomatic Carotid Artery Disease: A Randomized Prospective Study. Ann Thorac Cardiovasc Surg 2015; 21: 544-50
33. Mohandas BS, Jagadeesh AM, Vikram SB. Impact of monitoring cerebral oxygen saturation on the outcome of patients undergoing open heart surgery. Ann Card Anaesth 2013; 16: 102-6
34. Slater JP, Guarino T, Stack J, et al. Cerebral oxygen desaturation predicts cognitive decline and longer hospital stay after cardiac surgery. Ann Thorac Surg 2009; 87: 36-44; discussion 44-5
35. Vretzakis G, Georgopoulou S, Stamoulis K, et al. Monitoring of brain oxygen saturation (INVOS) in a protocol to direct blood transfusions during cardiac surgery: a prospective randomized clinical trial. J Cardiothorac Surg 2013; 8: 145
36. Rogers CA, Stoica S, Ellis L, et al. A Randomised Trial of Near Infra-Red Spectroscopy for the Personalised Optimisation of Cerebral Tissue Oxygenation during Cardiac Surgery. British Journal of Anaesthesia 2017;DOI: 10.1093/bja/aex182
37. Murkin JM AS, Schaefer B. Monitoring cerebral oxygen saturation significantly decreases major organ morbidity in CABG patients: A randomized blinded study. Heart Surg Forum 2004; 7: 515
38. Murkin JM, Adams SJ, Pardy E, Quantz M, McKenzie FN, Guo L. Monitoring brain oxygen saturation during coronary bypass surgery improves outcomes in diabetic patients: a post hoc analysis. Heart Surg Forum 2011; 14: E1-6
39. Kok WF, van Harten AE, Koene BM, et al. A pilot study of cerebral tissue oxygenation and postoperative cognitive dysfunction among patients undergoing coronary artery bypass grafting randomised to surgery with or without cardiopulmonary bypass*. Anaesthesia 2014; 69: 613-22
40. Dullenkopf A, Baulig W, Weiss M, Schmid ER. Cerebral near-infrared spectroscopy in adult patients after cardiac surgery is not useful for monitoring absolute values but may reflect trends in venous oxygenation under clinical conditions. J Cardiothorac Vasc Anesth 2007; 21: 535-9
41. Zheng F, Sheinberg R, Yee MS, Ono M, Zheng Y, Hogue CW. Cerebral near-infrared spectroscopy monitoring and neurologic outcomes in adult cardiac surgery patients: a systematic review. Anesth Analg 2013; 116: 663-76

For peer review only

For peer review only - http://bmjopen.bmj.com/site/about/guidelines.xhtml

42. Yu Y, Zhang K, Zhang L, Zong H, Meng L, Han R. Cerebral near-infrared spectroscopy (NIRS) for perioperative monitoring of brain oxygenation in children and adults. Cochrane Database of Systematic Reviews 2014; Issue 1.

For peer review only

For peer review only - http://bmjopen.bmj.com/site/about/guidelines.xhtml

Table 1. Summary of findings for all primary and secondary outcomes

STUDY	*MORTALITY*		*MI N (%)*		*STROKE N (%)*		*AKI N (%)*		*BLEEDING N (%)*		*TRANSFUSION N (%)*		*ICU LOS (H) MEAN (SD)*		*HOSP LOS (D) MEAN (SD)*	
	NIRS	Control	NIRS	Control	NIRS	Control	NIRS	Control	NIRS	Control	NIRS	Control	NIRS	Control	NIRS	Control
**ROGERS, 2017[36]**	1 (1)	2 (1.88)	1 (1)	2 (1.88)	1 (1)	0	2 (2)	5 (4.71)	6 (6.1)	4 (3.77)	37 (37.7)	44 (45.8)	3.3 (0.81)	3.67 (0.79)	7.5 (1.15)	8 (1.17)
**DESCHAMPS, 2016 [29]**	4 (11.7)	6 (13)	0	0	1 (2.9)	2 (4.3)	12 (35.2)	17 (47.2)	-	-	-	-	2.99 (2.26)	0.39 (2.05)	11 (7.2)	9.9 (5.8)
**KARA, 2015[32]**	-	-	-	-	-	-	-	-	-	-	-	-	1.74 (0.81)	2.12 (1.05)	7.15 (1.39)	7.67 (1.14)
**COLAK, 2015[28]**	-	-	8 (8.5)	7 (7.29)	4 (4.25)	1 (1)	0	1 (1)	0	1 (1)	77 (81)	73 (76)	2.7 (6.2)	1.9 (0.9)	-	-
**DESCHAMPS, 2013[30]**	-	-	-	-	-	-	-	-	-	-	-	-	2.99 (2.26)	0.39 (2.05)	7.6 (5.4)	7.9 (3.2)
**VRETZAKIS, 2013[35]**	1 (1.33)	1 (1.33)	2 (2.66)	2 (2.66)	1 (1.33)	0	1 (1.33)	0	1 (1.33)	1 (1.33)	51 (68)	63 (84)	2.7 (3.8)	2.7 (3.6)	10.9 (3.6)	10.2 (10.7)
**MOHANDAS, 2013[33]**	-	-	-	-	0	2 (4)	-	-	-	-	-	-	1.49 (0.38)	1.7 (0.49)	-	-
**HARILALL, 2013[31]**	-	-	-	-	-	-	-	-	-	-	-	-	-			

For peer review only - http://bmjopen.bmj.com/site/about/guidelines.xhtml

**SLATER, 2008**[34]	-	-	1 (0.8)	1 (0.86)	1 (0.8)	0	1 (0.8)	1 (0.86)	-	-	-	-	-	-	-	
**MURKIN, 2007**[10]	0	1 (1%)	1 (1%)	3 (3%)	1 (1%)	4 (4%)	0	0	1 (1%)	1 (1%)	8 (8)	10 (10)	1.25 (0.84)	1.87 (2.67)	6.1 (4.4)	6.9 (5.5)
**SUMMARY EFFECT ESTIMATE RR(95% CI)**	**0.76 (0.30 to 1.96)**		**0.90 (0.43 to 1.89)**		**1.08 (0.40 to 2.91)**		**0.88 (0.52 to 1.49)**		**1.23 (0.46 to 3.32)**		**0.93 (0.77 to 1.12)**		**0.00 (-0.44 to 0.44)**		**-0.45 (-0.90 to 0.01)**	

MI, Myocardial Infarction, AKI, Acute Kidney Injury, ICU, Intensive Care Unit, Hosp, Hospital, Length of Stay, LOS.

For peer review only

For peer review only - http://bmjopen.bmj.com/site/about/guidelines.xhtml

Table 2. Assessment of Neurocognitive Dysfunction

**Domain**	**Test Protocol**	**Kara, 2015**[32]	**Colak, 2015**[28]	**Rogers, 2017**[36]	**Mohandas, 2013**[33]	**Slater, 2008**[34]
**Timing**		Baseline and and pre-discharge	Baseline and 7 days	Baseline, 7 days and three months	Baseline, 7 days and three months	Baseline,pre-discharge and three months
**Attention**	Trail- Making Test parts A and B or equivalent		X (CTT)	X		X (SCW)
**Verbal Memory**	Rey Auditory Verbal Learning Test (VLT) or Hopkins VLT			X		X
**Visuo-Spatial**	Block Design from the Wechsler Adult Intelligence Scale			X		
**Psychomotor Speed**	Digit Symbol Test from the Wechsler Adult Intelligence Scale			X		
**Executive Funtion/ Verbal Fluency**	Controlled Oral Word Association Test			X		
**Motor Cordination**	Grooved Pegboard Test		X	X		X
**Other**	Saccadic and anti-saccadic eye movements		X		X	X
	Montreal Cognitive Asessment	X				
**Measurement of Confounders**						
	MMSE		X	X	X	X
	Anxiety Depression			X		X

For peer review only

For peer review only - http://bmjopen.bmj.com/site/about/guidelines.xhtml

Domain	Test Protocol	Kara, 2015[32]	Colak, 2015[28]	Rogers, 2017[36]	Mohandas, 2013[33]	Slater, 2008[34]
	Adult Reading Test			X		
	Medication			X		
	Delirium Rating Score					X
**Presented Test Data**		X		X	X	
**Definition of Cognitive Decline**		The maximum score to get from this test is 30. >25=Normal 19–25= mild cognitive imp. <19= serious cognitive imp..	Dichotomous:decrease in an MMSE score for three or more points from baseline value AND decrease of 1 SD (standard deviation) or more in performance on CTT 1 and GP tests.	Difference in group mean (p<0.05) in >3 of 6 domains	Postoperative MMSE impairment was defined as a decrease in scores by more than 20% of the preoperative values. Postoperative ASEM impairment was defined as a decrease of scores to more than 30% of preoperative values.	Cognitive decline was defined as a decline of one standard deviation or more in performance on one or more of the neuropsychologic tests.
**Reportd Difference in Cognitive Function**		X	X		X	
**Estimate of Difference**		Mild Cognitive Impairment 7/43 NIRS, 16/36 Control, p=0.01.Severe Cognitive Impairment 0/42 NIRS, 3/36 Control, p-0.09	28% NIRS vs 52% Control, p=0.0002	Significant difference in COWAT Mean Difference 3.73 (95%CI 1.5 to 5.96), p=0.0011	ASEM at 3 months Mean 15.69 (SD 3.99) NIRS, Mean 17.68 (SD 1.79), p<0.001	58% NIRS vs 61% Control

CTT: Colour Trail Test 1, SCW: Stroop Colour and Word; GP Test: Grooved Pegboard Test; MMSE: Mini- Mental State Examinations; ASEM: Anti saccadic eye

For peer review only

movements.

For peer review only

For peer review only - http://bmjopen.bmj.com/site/about/guidelines.xhtml

Table 3. Summary of main findings of systematic review and Grade Assessment of trial results.

Near Infra Red Spectrodscopy Algorithm compared to Control (Standard Care) in Cardiac Surgery

Patient population: Adult Cardiac surgery, **Setting:** Tertiary cardiac centres

Intervention: Near Infra Red Spectroscopy Algorithms for Personalised Optimisation of Cerebral Oxygenation

Control: Standard care

Outcomes	Anticipated absolute effects* (95% CI)		Relative Effect (95% CI)	N of Participants (studies)	Quality og the evidence (GRADE)
	Risk with Control	Risk with NIRS			
Mortality	32 per 1000	**25 per 1000** (10 to 63)	**RR 0.76** (0.30 to 1.96)	608 (4 RCTs)	⊕⊕○○ Low
Red cell transfusion	504 per 1000	**469 per 1000** (388 to 564)	**RR 0.93** (0.77 to 1.12)	744 (4RCTs)	⊕⊕○○ Low
Stroke	16 per 1000	**17 per 1000** 6 to 46)	**RR 1.08** (0.40 to 2.91)	1138 (7 RCTs)	⊕○○○ Very Low
Myocardial infarction	29 per 1000	**26 per 1000** (12 to 54)	**RR 0.90** (0.43 to 1.89)	1038 (6 RCTs)	⊕○○○ Very Low

For peer review only

For peer review only - http://bmjopen.bmj.com/site/about/guidelines.xhtml

Renal failure	71 per 1000	**62 per 1000**<br>(41 to 95)	**RR 0.88**<br>(0.58 to 1.34)	1043 (6 RCTs)	⊕◯◯◯<br>Very Low
Reoperation for bleeding	19 per 1000	**21 per 1000**<br>(8 to 56)	**RR 1.11**<br>(0.41 to 3.04)	744 (4 RCTs)	⊕◯◯◯<br>Very Low
ICU length of Stay<br>(ICU LOS)		The mean ICU LOS in the intervention group was 0 (0.44 lower to 0.44 higher)			⊕◯◯◯<br>Very Low
Hospital length of stay<br>(H LOS)		The mean H LOS was 0.45 lower<br>(0.9 lower to 0.01 higher)			⊕⊕◯◯<br>Low
***The risk in the intervention group** (and its 95% confidence interval) is based on the assumed risk in the comparison group and the **relative effect** of the intervention (and its 95%CI)					
CI: Confidence interval; RR: Risk ratio; MD: Mean Difference; ICU: Intensive Care Unit; NIRS: Near Infra-red Spectroscopy					

For peer review only

For peer review only - http://bmjopen.bmj.com/site/about/guidelines.xhtml

Figure Legends

Figure 1. Risk of bias summaries for **A.** Individual studies and **B.** All studies**.**

Figure 2. Forrest plots demonstrating summary effect estimates for A. Mortality**, B.** Myocardial Infarction**, C.** Stroke**, D.** Stage 3 acute kidney injury or renal replacement therapy**.** Effect estimates derived using Random Effects meta-analysis. Heterogeneity expressed as Chi Squared and I^2.

For peer review only

For peer review only - http://bmjopen.bmj.com/site/about/guidelines.xhtml

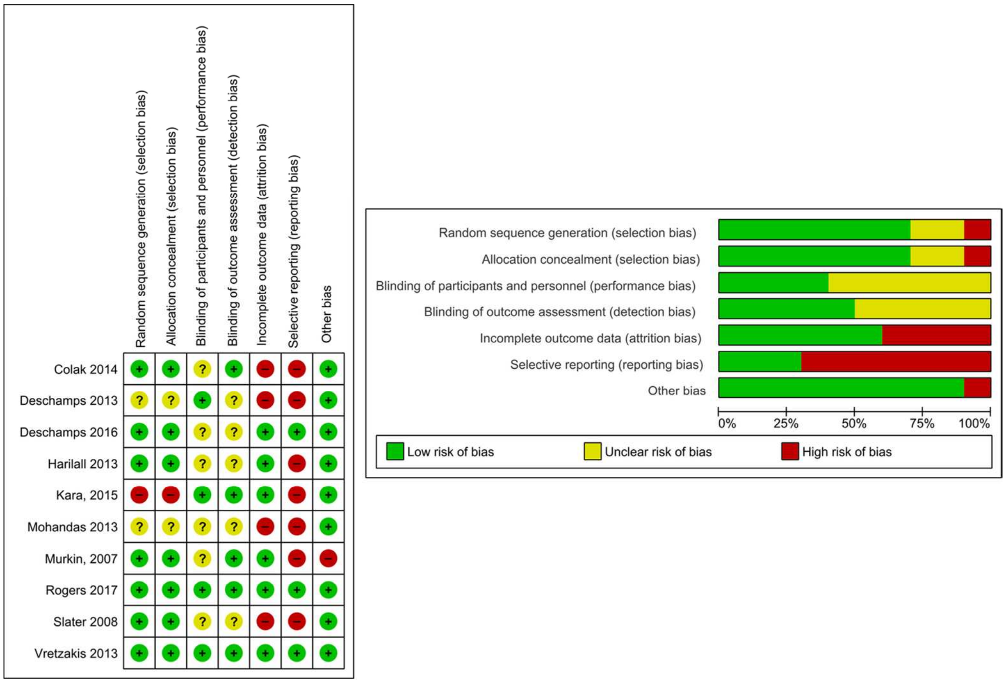

Figure 1

128x86mm (300 x 300 DPI)

view only

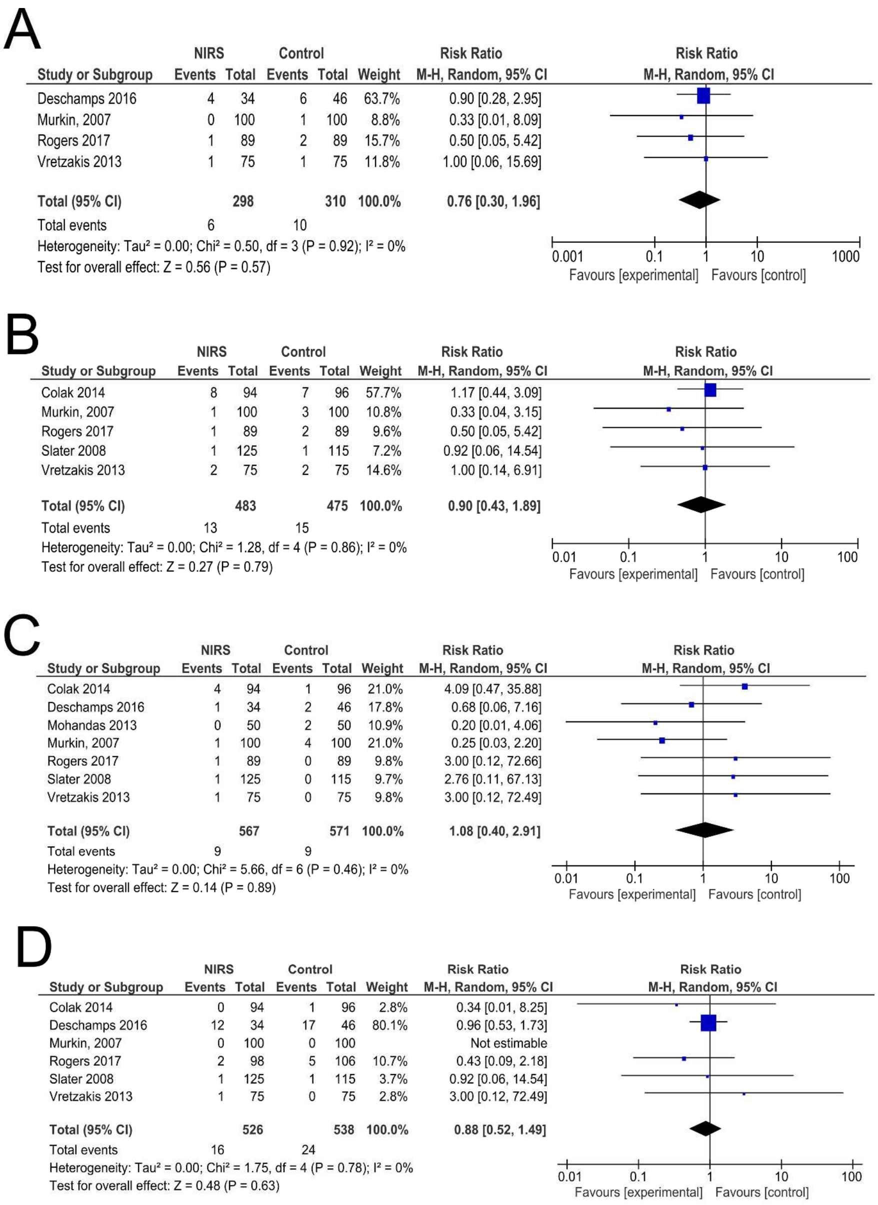

Figure 2

240x331mm (300 x 300 DPI)

Online only digital supplement: Efficacy of Near-Infrared Spectroscopy on the outcome of patients undergoing cardiac surgery: a systematic review of randomised trials.

Authors: GF Serraino, GJ Murphy

For peer review only

Table S1. Characteristics of Included Studies.

STUDY	*Location/ Funding*	*Sample size*	*Period*	*Inclusion/ Exclusion Criteria*	*Mean Age*<br>*-intervention group*<br>*-control group*<br>*Female (%)*<br>*- Intervention group*<br>*-control group*	*NIRS Device*	*Murkin Algorithm*	*Control*	*Outcomes*
Rogers, 2017[36]	3 UK centres<br>NIHR funded	204	2009-2014	Incl: CABG and non CABG.<br>Excl: Patients undergoing emergency cardiac surgery<br>Patients who are prevented from having blood and blood products according to a system of beliefs.	70±10<br>65.9±12.3<br>30%F<br>33%F	INVOS		SC	Primary: Cognitive function<br>Secondary: Units of RBC; Cerebral oxygenation; Oxygen delivery and utilisation during CPB: Quality of life; Infectious complications; Stroke; MI; ICU and H LOS; Post-operative acute kidney injury; Respiratory complications; Health and personal social services resource use and their costs. All-cause mortality within 30 days of surgery; Biochemical markers of organ injury

For peer review only

Deschamps, 2016 [29]	Multicentre<br>Regional funders<br>Canada	201	2012 – 2013	Incl: high-risk cardiac surgery<br>Excl: Off -pump coronary artery bypass surgery, emergency surgery, planned deep hypothermic circulatory arrest, acute endocarditis, presence of active delirium or encephalopathy.	69±12.6<br>72±9.4<br>27.4%F<br>28.3%F	FORE-SIGHT, EQUANOX Classic 7600, INVOS 5100C-PB		SC	Primary Outcome: success rate of reversing cerebral desaturations below 10% relative to baseline in the intervention group<br>Secondary Outcomes: cerebral desaturation load, 30-day follow-up for adverse events
Kara, 2015 [32]	Single centre,<br>Sakarya, Turkey	79	2013-2015	Incl: CABG<br>Excl: Other procedures, high degrees of aortic atherosclerosis	59.1±9.4<br>61.2±10.3<br>23.3%F<br>19.4%F	INVOS		SC	Primary: Cognitive impairement<br>(MoCa score)<br>Secondary: ICU and H LOS
Colak, 2015[28]	Single centre, Zagreb,<br>Croatia<br>Insitutional support grant	200	2009-2010	Incl: CABG<br>Excl: carotid artery stenosis, previous stroke or head injury, seizure, psychiatric illness, (NYHA III/IV) LVEF<25%, emergency off-pump CABG and severely impaired renal and liver function.	61.9±7.1<br>63.4±8.8<br>20%F<br>24%F	INVOS		SC	Primary: Cognitive impairement<br>MMSE<br>CTT1<br>GP test<br>Secondary: coma, stupor, transient ischaemic attack (TIA) or stroke, neuropsychological deficits
Deschamps, 2013[30]	Single centre, Quebec, Canada. Insitutional support grant	49	NS	Incl: CABG and non-CABG<br>Excl: Emergency surgery, first time CABG surgery, single valve surgery	70.2±9.2<br>71.1±7.9<br>15%F<br>15%F	INVOS	X	SC	Primary: rSO2<br>Secondary: ICU and H LOS

For peer review only

Vretzakis, 2013[35]	Single centre, Greece<br>Insitutional support grant	50	16-month period	Incl: CABG and non CABG<br><br>Excl: Emergency, re-do operations, combined cardiac - carotid surgery circulatory arrest. hematologic disease coagulation abnormality, advanced cirrhosis and renal dysfunction	67.3±8.5<br>65.9±9.5<br><br>12%F<br>15%F	INVOS		SC	Primary: RBC transfusion,<br>ICU and H LOS<br>Death<br><br>Secondary: Major complications
Mohandas, 2013[33]	Single centre, Bangalore, India<br>RCT	100	NS	Incl: On pump<br>Cardiac surgery<br><br>Excl: pre-existing neuropsychiatric disorders, inability to correctly perform the neurocognitive tests, and mini-mental state examination (MMSE) scores of less than 23	38.05±15.81<br>34.60±16.28<br><br>44%F<br>44%F	NONIN EQUANOX	X	SC	Primary: Cognitive decline:<br>MMSE<br>ASEM<br><br>Secondary: ICU LOS
Harilall, 2013[31]	Single centre, South Africa	40	NS	Incl: CABG<br><br>Excl: pregnancy, history of stroke or per- sistent neurological residue, history of transient ischaemic attack (TIA), stenosis of carotid artery	55.3±9.7<br><br>30%F (all)	INVOS	X	SC	Primary: rSO2<br><br>Secondary: S100B

For peer review only

Slater, 2008[34]	Single centre, New Jersey, USA	265	2004-2006	Incl: CABG<br><br>Excl: preexisting neuropsychiatric disorders, inability to correctly perform the neurocognitive tests, and mini- mental state examination score of 23 or less.	64.8±10.1<br>64.78±9.9<br><br>31%F (all)	INVOS	X	SC	Primary: Cognitive impairement<br><br>Secondary: ICU LOS
Murkin, 2007[10]	Single centre, Ontario, Canada<br><br>Grant and other support from the device manufacturer.	200	2002 – 2004	Incl: >18 yr, CABG	61.8±10.3<br>61.8±9.3<br><br>12.5%F (all)	INVOS	X	SC	Primary: 30 days Death, MMOM<br><br>Secondary: LOS

For peer review only

Table S2. Characteristics of Excluded Studies

STUDY	*Study Type/ Location*	*SAMPLE*	*PERIOD*	*INCLUS. CRIT.*	*EXCLUS. CRIT.*	*Mean Age*	*Female (%)*	*NIRS Device*	*Murkin Algorithm*	*Control*	*OUTCOMES*
Dullenkpof, 2007 [40]	Prosp, RCT, Single centreZurich, Switzerland **NO intervention**	35	NS	Elective cardiac surgery with insertion of PAC	Cerebral perfusion disturbence, CAD, intracardiac shunt	65.5±10.9	74.2	INVOS			Primary: rSO2
Kok, 2014 [39]	Single centre Groningen NH	59	2011 – 2012	CABG	Other procedures, difficulty completing cognitive tests, difficulty with dutch lang, impaired finction of the dominant arm or hand, history of head trauma, stroke or neurosurgery, severe CAD	62.8±9.4	10	Somanetics (Michigan) Casmed (Branford)		On pump vs Off Pump surgery	Primary: Cerebral desaturation Secondary: Postop cognitive disfunction; major complications; ICU and Hospital LOS

PAC pulmonary artery catheter, CABG coronary artery bypass grafts, ICU intensive care unit, LOS length of stay

For peer review only

Table S3. Sub Group Analyses

SUB GROUP	CABG	NON CABG	TEST FOR SUBGROUP DIFFERENCES	MURKIN	NON MURKIN	TEST FOR SUBGROUP DIFFERENCES	CONSENSUS NEUROCOGNITIVE ASSESSMENT	NON CONSENSUS NEUROCOGNITIVE ASSESSMENT	TEST FOR SUBGROUP DIFFERENCES	RESTRICTIVE RED CELL TRANSFUSION TRIGGER	NO RESTRICTIVE RED CELL TRANSFUSION TRIGGER	TEST FOR SUBGROUP DIFFERENCES
**MORTALITY RR (95% CI)**	0.33 (0.01 to 8.09) 1 trial, n=200, I^2 =NA	0.81 (0.30 to 2.18) 3 trials, n=408, I^2=0%	**Chi^2 = 0.50, df = 3 (P = 0.92), I^2 = 0%**	0.77 (0.26 to 2.32) 2 trials, n=289, I2 =NA	0.67 (0.11 to 3.94) 2 trials, n=328, I^2=0%	**Chi^2 = 0.50, df = 3 (P = 0.92), I^2 = 0%**	0.50 (0.05 to 5.42) 1 trial, n=178 I2=NA	0.94 (0.39 to 2.26) 3 trials, n=430 I2=4%	**Chi^2 = 0.23, df = 3 (P = 0.97), I^2 = 0%**	0.67 (0.11 to 3.94) 2 trials, n=328 I2=0%	0.77 (0.26 to 2.32) 2 trials, n= 280 I2= NA	**Chi^2 = 0.50, df = 3 (P = 0.92), I^2 = 0%**
**STROKE RR (95% CI)**	1.18 (0.38 to 3.63) 3 trials, n=630. I2 =43%	0.89 (0.26 to 3.10) 4 trials, n= 508 I2=1%	**Chi^2 = 5.66, df = 6 (P = 0.46), I^2 = 0%**	0.84 (0.33 to 2.11) 5 trials, n=810 I2=14%	3.00 (0.32 to 28.54) 3 trials, n=328 I2=0%	**Chi^2 = 5.66, df = 6 (P = 0.46), I^2 = 0%**	3.00 (0.12 to 72.66) 1 trial, n=178 I2: NA	1.00 (0.39 to 2.56) 6 trials, n=960 I2=22%	**Chi^2 = 5.66, df = 6 (P = 0.46), I^2 = 0%**	3.00 (0.32 to 28.54) 2 trials, n=328 I2=0%	0.84 (0.33 to 2.11) 5 trials, n=810 I2=14%	**Chi^2 = 5.66, df = 6 (P = 0.46), I^2 = 0%**
**MI RR (95% CI)**	0.92 (0.40 to 2.10) 3 trials, n=630, I2=0%	0.75 (0.17 to 3.30) 3 trials, n=408, I2=0%	**Chi^2 = 0.05, df = 1 (P = 0.82), I^2 = 0%**	0.92 (0.40 to 2.10) 4 trials, n=710, I2=0%	0.75 (0.17 to 3.30) 2 trials, n=328 I2=0%	**Chi^2 = 0.05, df = 1 (P = 0.82), I^2 = 0%**	0.50 (0.05 to 5.42) 1 trial, n=178 I2= NA	0.93 (0.43 to 1.99) 5 trials, n=860 I2=0%	**Chi^2 = 0.24, df = 1 (P = 0.63), I^2 = 0%**	0.75 (0.17 to 3.30) 2 trials, n=328 I2=0%	0.92 (0.40 to 2.10) 4 trials, n=710 I2=0%	**Chi^2 = 0.05, df = 1 (P = 0.82), I^2 = 0%**
**SEVERE AKI RR (95% CI)**	0.58 (0.08 to 4.41) 3 trials, n=630, I2=0%	0.88 (0.51 to 1.12) 4 trials, n=434, I2=0%	**Chi^2 = 0.15, df = 1 (P = 0.70), I^2 = 0%**	0.90 (0.51 to 0.19) 4 trials, n=710 I2=0%	0.67 (0.18 to 2.56) 2 trials, n=354 I2=12%	**Chi^2 = 0.15, df = 1 (P = 0.70), I^2 = 0%**	3.00 (0.12 to 72.49) 1 trial, n=150 I2=NA	0.96 (0.55 to 1.68) 3 trials, n=780 I2=0%	**Chi^2 = 0.48, df = 1 (P = 0.49), I^2 = 0%**	0.67 (0.18 to 2.56) 2 trials, n=354 I2= 12%	0.90 (0.51 to 1.19) 2 trials, n=630 I2=0%	**Chi^2 = 0.15, df = 1 (P = 0.70), I^2 = 0%**

**TRANSFUSION RR (95% CI)**	1.04 (0.89 to 1.23) 2 trials, n=390. I2=0%	0.85 (0.71 to 1.01) 2 trials, n=354. I2=0%	**Chi^2 = 2.81, df = 1 (P = 0.09), I^2 = 64.4%**	1.04 (0.89 to 1.23) 2 trials, n=390. I2=0%	0.85 (0.71 to 1.01) 2 trials, n=354 I2=0%	**Chi^2 = 2.81, df = 1 (P = 0.09), I^2 = 64.4%**	0.91 (0.65 to 1.28) 1 trial, n=150. I2=NA	0.94 (0.83 to 1.06) 5 trials, n=860 I2=66%	**Chi^2 = 0.04, df = 1 (P = 0.85), I^2 = 0%**	0.85 (0.71 to 1.01) 2 trials, n=354. I2= 0%	1.04 (0.89 to 1.23) 4 trials, n=710 I2=0%	**Chi^2 = 2.81, df = 1 (P = 0.09), I^2 = 64.4%**
**ICU LOS MD (95% CI)**	-0.39 (-0.71 to -0.06), 3 trials, n=469. I2=51%	-0.24 (-0.37 to -0.10), 5 trials, n=582. I2=82%	**Chi^2 = 0.70, df = 1 (P = 0.4), I^2 = 0%**	-0.19 (-0.35 to -0.03) 5 trials, n=618, I2=84%	-0.36 (-0.55 to -0.17) 3 trials, n=433, I2=0%	**Chi^2 = 1.87, df = 1 (P = 0.17), I^2 = 46.5%**	-0.37 (-0.59 to -0.15), 1 trial, n=204 I2= NA	-0.21 (-0.50 to 0.09) 6 trials, n=747 I2=81%	**Chi^2 = 0.77, df = 1 (P = 0.38), I^2 = 0%**	-0.36 (-0.57 to -0.14) 2 trials, n=354, I2=0%	-0.21 (-0.36 to -0.06), 6 trials, n=697, I2=81%	**Chi^2 = 1.18 df = 1 (P = 0.28), I^2 = 14.9%**
**HOSP LOS MD (95% CI)**	-056 (-1.08 to -0.04), 2 trials, n=279. I2=0%	-0.46 (-0.77 to -0.15), 4 trials, n=482, I2=0%	**Chi^2 = 0.1, df = 1 (P = 0.75), I^2 = 0%**	-0.43 (-1.55 to 0.69) 3 trials, n=328. I2=0%	-0.49 (-0.77 to -0.22) 3 trials, n=433 I2=0%	**Chi^2 = 0.01, df = 1 (P = 0.91), I^2 = 0%**	-0.50 (-0.82 to -0.18) 1 trial, n=204 I2= NA	-0.46 (0.05 to 0.93) 5 trials, n=557 I2=0%	**Chi^2 = 0.02, df = 1 (P = 0.89), I^2 = 0%**	-0.48 (-0.80 to -0.17), 2 trials n=354, I2=0%	-0.50 (-1.00 to -0.00) 4 trials, n=407 I2=0%	**Chi^2 = 0.00, df = 1 (P = 0.95), I^2 = 0%**

RR: Risk ratio, MD: Mean Difference, 95%CI: 95% confidence intervals, MI: Myocardial Infarction, AKI: Acute Kidney Injury, ICU: Intensive care unit, Hosp Hospital, LOS: Length of stay, CABG: coronary artery bypass grafting.

Figure. S1 PRISMA Flowchart of included studies

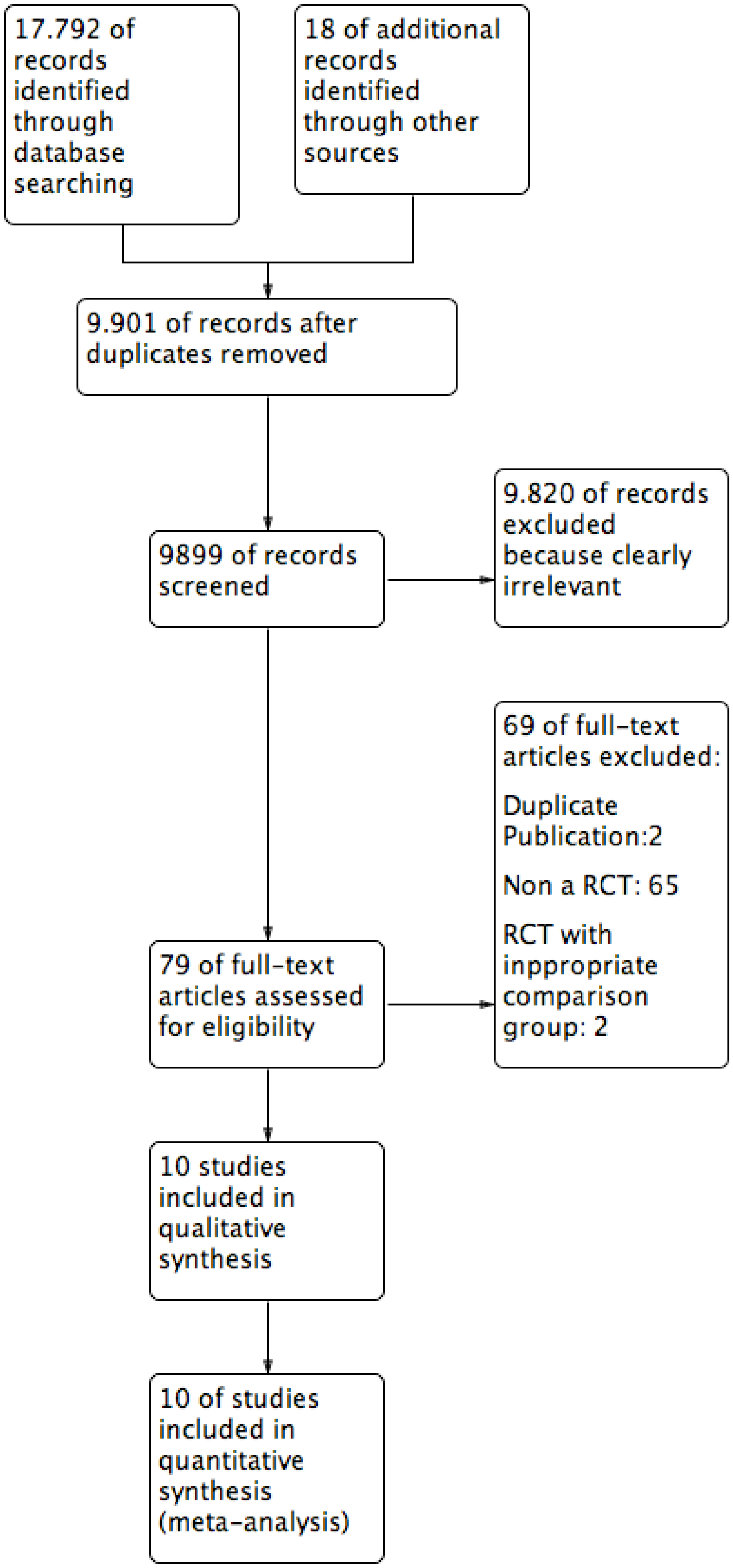

Figure S2: Effects of using NIRS on Bleeding, Transfusion and Time to discharge from ICU or Hospital.

RBC Transfusion

	NIRS		Control			Risk Ratio
Study or Subgroup	Events	Total	Events	Total	Weight	M-H, Random, 95% CI
Colak 2014	77	94	73	96	41.0%	1.08 [0.93, 1.25]
Murkin, 2007	8	100	10	100	4.0%	0.80 [0.33, 1.94]
Rogers 2017	37	98	44	106	19.3%	0.91 [0.65, 1.28]
Vretzakis 2013	51	75	63	75	35.7%	0.81 [0.67, 0.97]
**Total (95% CI)**		**367**		**377**	**100.0%**	**0.93 [0.77, 1.12]**
Total events	173		190			

Heterogeneity: Tau² = 0.02; Chi² = 6.07, df = 3 (P = 0.11); I² = 51%
Test for overall effect: Z = 0.77 (P = 0.44)

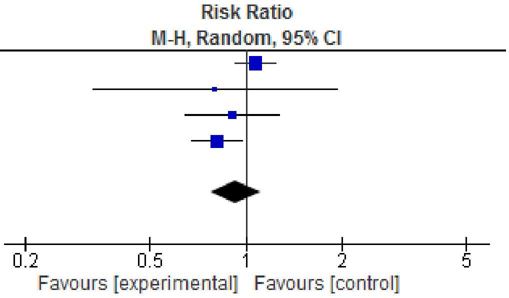

Reoperation for bleeding

	NIRS		Control			Risk Ratio
Study or Subgroup	Events	Total	Events	Total	Weight	M-H, Random, 95% CI
Colak 2014	0	94	1	96	9.7%	0.34 [0.01, 8.25]
Murkin, 2007	1	100	1	100	12.9%	1.00 [0.06, 15.77]
Rogers 2017	6	98	4	106	64.4%	1.62 [0.47, 5.58]
Vretzakis 2013	1	75	1	75	13.0%	1.00 [0.06, 15.69]
**Total (95% CI)**		**367**		**377**	**100.0%**	**1.23 [0.46, 3.32]**
Total events	8		7			

Heterogeneity: Tau² = 0.00; Chi² = 0.86, df = 3 (P = 0.83); I² = 0%
Test for overall effect: Z = 0.41 (P = 0.68)

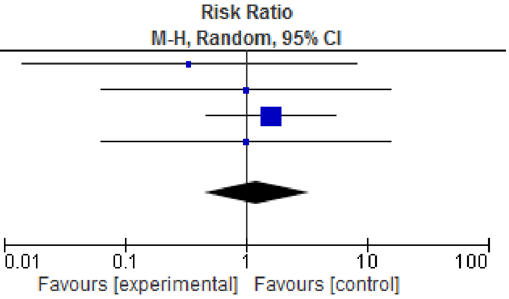

Intensive care Unit Length of Stay

	NIRS			Control				Mean Difference
Study or Subgroup	Mean	SD	Total	Mean	SD	Total	Weight	IV, Random, 95% CI
Colak 2014	2.7	6.2	94	1.9	0.9	96	5.6%	0.80 [-0.47, 2.07]
Deschamps 2013	2.99	2.26	23	0.39	2.05	25	5.9%	2.60 [1.38, 3.82]
Deschamps 2016	2.65	2.42	34	3.35	3.61	46	5.3%	-0.70 [-2.02, 0.62]
Kara, 2015	1.74	0.81	43	2.12	1.05	36	17.6%	-0.38 [-0.80, 0.04]
Mohandas 2013	1.49	0.38	50	1.7	0.49	50	22.6%	-0.21 [-0.38, -0.04]
Murkin, 2007	1.25	0.84	100	1.87	2.67	100	14.9%	-0.62 [-1.17, -0.07]
Rogers 2017	3.3	0.81	98	3.67	0.79	106	21.8%	-0.37 [-0.59, -0.15]
Vretzakis 2013	2.7	3.8	75	2.7	3.6	75	6.2%	0.00 [-1.18, 1.18]
**Total (95% CI)**			**517**			**534**	**100.0%**	**-0.13 [-0.47, 0.22]**

Heterogeneity: Tau² = 0.13; Chi² = 27.52, df = 7 (P = 0.0003); I² = 75%
Test for overall effect: Z = 0.71 (P = 0.48)

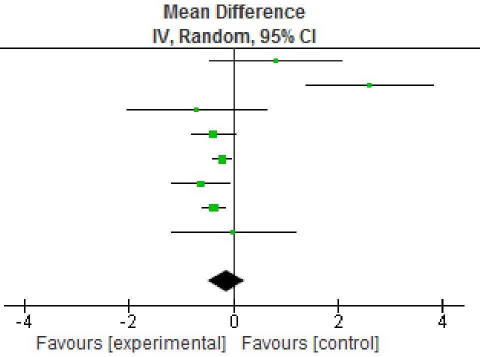

Hospital Length of Stay

	NIRS			Control				Mean Difference
Study or Subgroup	Mean	SD	Total	Mean	SD	Total	Weight	IV, Random, 95% CI
Deschamps 2013	7.6	5.4	23	7.9	3.2	25	3.2%	-0.30 [-2.84, 2.24]
Deschamps 2016	11	7.2	34	9.9	5.8	46	2.4%	1.10 [-1.84, 4.04]
Kara, 2015	7.15	1.39	43	7.67	1.14	36	66.3%	-0.52 [-1.08, 0.04]
Murkin, 2007	6.1	4.4	100	6.9	5.5	100	10.8%	-0.80 [-2.18, 0.58]
Rogers 2017	8.5	4.2	98	8.9	4.6	106	14.1%	-0.40 [-1.61, 0.81]
Vretzakis 2013	10.9	3.6	75	10.2	10.7	75	3.2%	0.70 [-1.85, 3.25]
**Total (95% CI)**			**373**			**388**	**100.0%**	**-0.45 [-0.90, 0.01]**

Heterogeneity: Tau² = 0.00; Chi² = 2.17, df = 5 (P = 0.83); I² = 0%
Test for overall effect: Z = 1.94 (P = 0.05)

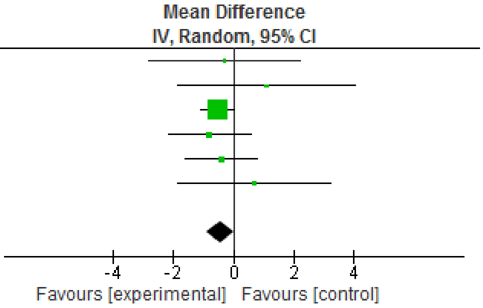

PROTOCOL INFORMATION

Protocol title: Efficacy of Near-Infrared Spectroscopy on the outcome of patients undergoing cardiac surgery: a systematic review.

For peer review only

Authors

**Prof Gavin J. Murphy**<br>British Heart Foundation<br>Professor of Cardiac Surgery<br>Department of Cardiovascular Sciences<br>University of Leicester<br>Glenfield Hospital Groby Road Leicester,<br>LE3 9QP Tel: 0116 258 3054<br>Email: gjm19@le.ac.uk	**Dr Giovanni Mariscalco**<br>Senior Lecturer<br>Department of Cardiovascular Sciences<br>University of Leicester<br>Glenfield Hospital Groby Road Leicester,<br>LE3 9QP Tel: 0116 258 3019<br>Email: gm247@leicester.ac.uk
**Dr Giuseppe Filiberto Serraino**<br>Clinical Research Fellow<br>Department of Cardiovascular Sciences University of Leicester Glenfield Hospital Groby Road Leicester,<br>LE3 9QP Email: gfs3@le.ac.uk	

Contact person: Professor Gavin J Murphy, British Heart Foundation Professor of Cardiac Surgery University of Leicester, Glenfield Hospital Groby Road Leicester, LE3 9QP. Tel: 0116 258 3054, Email: gjm19@le.ac.uk

Funding sources/sponsor

Leicester Cardiovascular Biomedical Research Unit

Dates

Anticipated or actual start date ---

Anticipated completion date ----

Type of review

Epidemiologic; Intervention

Language

English

Country

United Kingdom

Keywords

Systematic review; Cardiac Surgery; Near Infra-Red Spectroscopy; Near Infrared Oximetry; Cognitive dysfunction.

For peer review only

ABSTRACT

Background: Organ injury attributable to cardiopulmonarybypass is a major cause of morbidity, mortality and resource use in cardiac surgery patients. Regional tissue hypoxia during CPB has been implicated in perioperative organ injury. It has been suggested that goal directed patient specific optimisation of regional (brain) tissue hypoxia measured using Near Infra-Red Specroscopy (NIRS) may reduce the frequency of brain injury. Other investigators have suggested that cerebral NIRS based algorithsm may have utility for the protection of other organs, or a part of a restricitive red cell transfusion protocol.

Objectives: Our primary objective is to establish whether the use of perioperative goal directed interventions that aim to optimise cerebral Near Infrared Spectroscopy readings result in reductions in measures of cerebral injury (neurocognitive function, serum biomarkers), injury to other organs including the heart and brain, transfusion rates, mortality and resource use.

Search methods: We will conduct the search in October 2015. Potentially eligible trials will be identified by searching the Cochrane Central Register of Controlled Trials, MEDLINE , and EMBASE, using a combination of subject headings and text words to identify relevant trials. The Medline search strategy below will be adapted as appropriate for other databases.

((Cardiopulmonary Bypass) OR (Cardiac Surgery) OR (Coronary Artery Bypass) OR (Extra Corporeal Circulation)OR(Perioperative Morbidity)) AND ((Near Infra-Red Spectroscopy) OR (Oximetry) OR (Brain/Metabolism)OR(Cerebral Desaturation)OR(Cerebral Perfusion)OR(Cerebral Ischemia)OR(Cerebral Oximetry)OR(Cerebral Saturation)OR(Near Infrared Oximetry)(Cognitive)).

Selection Criteria: Two review authors will independently select references for further assessment by going through all titles and abstracts. Further selection will be based on review of full-text articles for selected references.

Data Collection and Analysis: Two review authors will independently extract study data. We will calculated the risk ratio (RR) with 95% confidence interval (CI) for binary outcomes and the mean difference (MD) or the standardised mean difference (SMD) with 95% CI for continuous outcomes. We will perform meta-analysis when possible, when I^2 is less than or equal to 80% using a fixed-effect or random-effects model, using Review Manager software. The range of point estimates for individual studies will be presented when

For peer review only

$I^2 > 80\%$. Heterogenity will be explored using subgroup analyses. Sensitivity analyses will explore the robustness of our primary analysis to exclusion of studies at high risk of bias.

Summary: We will use GRADEpro software to prepare the ’Summary of findings’table. We will judge the overall quality of the evidence foreach outcome as ‘high,’ ‘moderate,’ ‘low’ or ‘very low’ accordingto the GRADE (Grades of Recommendation, Assessment, Developmentand Evaluation) approach.

BACKGROUND

Organ injury is a common and severe complication of cardiac surgery with cardiopulmonary bypass (CPB), where it contributes to mortality, morbidity and the increased use of hospital resources [1]. In a recent trial where data on organ dysfunction was prospectively collected clinically significant kidney, lung and myocardial injury occurred in 34%, 16% an 11% of all patients and contributed to 41%, 36% and 24% of all deaths respectively [2]. The pathophysiology of CPB associated morbidity is multifactorial but is thought to involve regional hypoperfusion and tissue hypoxia [3, 4], often within vascular beds which are already abnormal due to advanced age or comorbidities such as diabetes [5, 6]. Adequate tissue oxygen delivery during CPB is achieved by optimisation of several parameters including CPB pump flows, perfusion pressure, haematocrit and the oxygen saturation of arterial blood. In contemporary clinical practice in adult cardiac surgery, the adequacy of perfusion is determined by the use of global measures of oxygen utilisation such as the mixed venous oxygen saturation (SvO2) or evidence of tissue hypoxia as implied by elevated serum lactate, a marker of cell anaerobic metabolism, or other indicators of metabolic acidosis [7]. However, in some patients, particularly those with pre-existing end organ dysfunction, global measures may not detect regional hypoxia and it has been suggested that measures of regional or tissue specific measures of oxygenation may have greater clinical utility [8-11].

The Intervention

Direct measures of regional tissue oxygen levels such as gastric tonometry, laser Doppler flowmetry of the intestinal mucosa, or cerebral venous oxygen saturation using jugular bulb catheters, can be used to measure and optimise tissue oxygenation but these modalities are invasive, which limits their use. More

For peer review only

recently, Near Infra-red Spectroscopy (NIRS) has emerged as a technique to monitor regional tissue oxygenation non-invasively and accurately with the added benefit that it can accurately measure tissue oxygenation in one of the most important end organs, the brain. NIRS sensors, when applied to the forehead, can determine the relative saturation / desaturation of blood within the cerebral arterioles and venules of the forebrain. Sas the total volume of blood is domintaed by th enemous compartment this measure is considered to accurately reflect cerebral venous oxygen saturation [12, 13] and is now approved as a non-invasive measure of regional cerebral oxygenation by the Medicines and Healthcare Products Regulatory Agency (MHRA). Early clinical studies suggested that the use of this technoliogy to optimise cerebral oxygenation during CPB have shown that optimisation of cerebral oxygenation using NIRS can be associated not only with reduced neurological morbidity but also with a reduction in renal complications and other major adverse clinical events [14-16]. However in the absence of high quality evidence there is uncertainty as to the efficacy or cost effectiveness of this device and there is wide variation in its use [ ].

Indications for red blood cell transfusion during cardiac surgery

Cerebral oximetry may also be used during CPB to develop goal-directed, patient-specific indicators of the need for red blood cell (RBC) transfusion if used as part of a wider algorithm designed to optimise cerebral oxygenation. The primary goal of RBC transfusion is to optimise tissue oxygenation. Currently, most RBC transfusions are given solely as a response to a haematocrit that has fallen below an arbitrary threshold and not as a response to incipient tissue hypoxia [18, 19]. The haematocrit is a poor indicator of regional tissue hypoxia and, therefore, is inadequate as an indicator of the need for transfusion.The haematocrit below which oxygen delivery to tissues is reduced such that anaerobic metabolism occurs is known as the critical Haematocrit (Hcrit). In healthy human adults very low haematocrits of less than 15 can cause organ hypoxia [9, 11] and transfusion in such cases is thought to be beneficial [20, 21]. The Hcrit for patients during cardiac surgery, however, is unclear. It is thought to be higher than 15, as most patients are elderly and have comorbid conditions, but it is also likely that the Hcrit varies considerably both between cardiac patients as well as for individual patients over the course of the perioperative period [22]. Hcrit is increased by conditions which impair autoregulation such as diabetes or increased age [5, 6] and during CPB, Hcrit is

also affected by multiple factors that affect the balance of oxygen supply and oxygen demand such as hypothermia, re-warming, pump flow or perfusion pressure [23]. Uncertainty about when an individual may benefit from a transfusion is reflected in the wide range in reported haematocrit transfusion thresholds used during CPB (from 17-25) [24-26] as well as the wide variation in transfusion rates in cardiac surgical patients across units in the United Kingdom (UK) (35-75%) [27]. Because the Hcrit for any particular patient at any given time is unknown, the ability to directly measure tissue oxygenation and therefore incipient tissue hypoxia may have distinct advantages over the use of generic and pre-specified haematocrit transfusion thresholds.

How the intervention might work

Existing protocols to optimise tissue oxygenation during CPB deploy manipulations of CPB (according to levels of blood markers such as lactate or pH) and the transfusion of RBC (according to the haematocrit). These methods are often treated independently of each other, and in the case of trandsfusion commonly guided by protocolised thresholds, despite the fact that both have the underlying aim of improving tissue oxygenation [7].

In contrast the patient-specific algorithm differs from the standard care in two key ways. First it is 'goal-directed', in that the algorithm is specifically targeted to maintain cerebral oxygen delivery during CPB (monitored by NIRS), a measure of regional rather than global tissue hypoxia. Second it allows the manipulation of multiple variables to optimise regional oxygenation for individual patients. This represents a form of personalised medicine. The most widely cited patient-specific algorithm developed by Murkin and colleagues [28] aims to optimise the cerebral oxygen supply / demand balance during CPB by: (a) increasing oxygen supply using hyperoxygenation, increased pump flow, perfusion pressure or hypercapnic cerebral vasodilation, (b) Increasing oxygen offloading by the use of nitrates, or (c) reducing oxygen demand by deepening anaesthesia. Cerebral oxygen saturations approaching a low threshold in the presence of anaemia (a haematocrit of between 18 and 23) and despite optimisation of other parameters suggest that the cerebral Hcrit is about to be reached and transfusion is indicated. Therefore, this algorithm is patient and time specific and goal-directed to optimise a validated objective measure of tissue oxygenation. This

For peer review only

should potentially reduce healthcare costs associated with complications attributable to tissue hypoxia during CPB or to unnecessary allogeneic RBC transfusions.

Why is it important to do this review

Numerous studies have reported the incidence of postoperative neurocognitive decline around 50% [29, 30]. Central venous oxygen desauration is common following cardiac surgery and this has been implicated in cognitive decline [3, 4]. Multiple studies have evaluated the efficacy of NIRS based algorithms that allow optimisation of cerebral saturation. Although some of these studies have been reported as positive [8] overall these results of these studies have been have been inconclusive, and no recent systematic review has attempted to summarise the available evidence. The use of this technology is variable and there is evidence of equipoise as to its effectiveness. To resolve this uncertainty we propose to undertake a systematic review and meta-analysis of the available evidence from RCTs.

OBJECTIVES

(1) To establish whether the use of perioperative goal directed interventions that aim to optimise cerebral Near Infrared Spectroscopy readings result in reductions in measures of cerebral injury (neuroscogitive function, serum biomarkers), morbidity, mortality and resource use.

(2) Does the use of perioperative goal directed interventions that aim to optimise cerebral Near Infrared Spectroscopy readings result in reductions in red cell transfusion, non red cell transfusion, or bleeding.

METHODS

Types of Studies

Randomized controlled trials irrespective of blinding, language, publication status, date of publication and sample size.

Types of Participants

Patients undergoing cardiac surgery for acquired or congenital disease, or aortovascular disease with or without cardiopulmonary bypass. No age restriction will be applied. There are no exclusion criteria.

For peer review only

Types of Interventions

Intervention: Goal directed NIRS algorithm.

Comparator /control: An untread group, or alternative (non NIRS based) goal directed therapy.

Types of Outcome Measure

Primary outcomes:

Mortality: 30 day or hospital all-cause mortality.

Secondary outcomes:

1. Acute brain injury; stroke, TIA as defined by study authors.
2. Low cardiac output as defined by study authors.
3. Myocardial Infarction as defined by study authors.
4. Acute kidney Injury Stage 3 [32] or requiring haemofiltration as defined by study authors.
5. Neurocognitive function; group means as described by neurocognitive tests. Tests recommended by a consensus statement to test all key domains of cognitive function [33] are marked with *. Studies will be categorised as Yes/ No as to whether they have assessed the key domains described in the Consensus Statement. Key domians [ ] that may be assessed are as follows
   - Attention: Sustained and divided attention: Consensus statements reccomend the Trail-Making Test parts A* and B* [35, 36].
   - Verbal memory: Consensus statements reccomend the RAVLT and Rey Auditory Verbal Learning Test* [36]
   - Visuo-spatial: as the Block Design from the Wechsler Adult Intelligence Scale [37]
   - Psychomotor speed: Consensus statements reccomend tests such the Digit Symbol Test from the Wechsler Adult Intelligence Scale [37]
   - Executive function/Verbal fluency: Consensus statements reccomend tests such the Controlled Oral Word Association Test [38]
   - Motor coordination: Consensus statements reccomend tests such the Grooved Pegboard Test*, dominant and non-dominant hand [33].
6. Neurocognitive dysfunction, as a dichotomous outcome reported by the study authors; A consensus definition is a change in a single test of >1 SD. This may be defined as change in a group mean (adjusted for baseline) or for individual patients [39]. Studies will be categorised as defining cognitive dysfunction using a Consensus versus a non-Consensus definitions.
7. Risk of receiving blood transfusion as defined by study authors.
8. Reoperation for bleeding as defined by study authors.
9. Resoiurce Use: ICU and hospital LOS as defined by study authors.
10. S100B levels as reported by study authors.

Search methods for identification of studies

For peer review only

Potentially eligible trials will be identified by searching the Cochrane Central Register of Controlled Trials, MEDLINE , and EMBASE, using a combination of subject headings and text words to identify relevant trials. The Medline search strategy below will be adapted as appropriate for other databases.

((Cardiopulmonary Bypass) OR (Cardiac Surgery) OR (Coronary Artery Bypass) OR (Extra Corporeal Circulation)OR(Perioperative Morbidity)) AND ((Near Infra-Red Spectroscopy) OR (Oximetry) OR (Brain/Metabolism)OR(Cerebral Desaturation)OR(Cerebral Perfusion)OR(Cerebral Ischemia)OR(Cerebral Oximetry)OR(Cerebral Saturation)OR(Near Infrared Oximetry)(Cognitive)).

To identify ongoing or unpublished trials we will also search the Clinicaltrials.gov using the following search terms:

Search terms: Randomized

Study Type: Interventional Studies

Conditions: Cardiac surgery OR Cardiopulmonary bypass

Interventions: Near Infra-Red Spectroscopy OR Near Infrared Oximetry OR Cerebral Desauturation

We will also examined the reference lists of eligible trials and reviews. Searches will not be restricted by language or publication status.

Data Collection and Analysis

The review will be performed in accordance with instructions given in the Cochrane Handbook for Systematic Reviews of Interventions (Higgins 2011).

Selection of Studies

Two reviewers GJM, GFS will identify trials for inclusion independently of each other. Exluded studies and the reason for exclusion will be recorded.

Data extraction (selection and coding)

Two authors will independently screen the search output to identify records of potentially eligible trials examining the outcomes, the full texts of which will be retrieved and assessed for inclusion.

A standardised form will be used to extract data from the included studies for assessment of study quality and evidence synthesis. Extracted information will include:

- Year and language of publication

For peer review only

- Country of Particiapnt recruitment
- Year of conduct of the trial
- Study setting; university teaching hospital, non universityteaching hospital
- Study population; inclusion and exlusion criteria
- Sample size
- Participant demographics
- Baseline characteristics
- Type of surgery
- Details of NIRS algorithm (Murkin, non Murkin [41])
- Deatails of Comparator; non NIRS goal directed therapy, standard care (protocolised care)
- Outcomes and times of measurement
- Information for assessment of the risk of bias.

Two review authors will extract data independently, discrepancies will be identified and resolved through discussion (with a third author where necessary). Missing data will be requested from study authors. If there is doubt as to whether trials share participiants completely or partially (with common authors and centres) we will contact the study authors to ascertain whether the studt report has been duplicated.

Risk of Bias

The following bias risk domains will be assessed as Low, Uncertain, or High based on the instructions given in the in the Cochrane Handbook for Systematic Reviews of Interventions [42];

- Sequence Generation
- Allocation Concealment
- Blinding of participant, personnel
- Blinding of outcome Assessors
- Incomplete outcome data
- Selective outcome reporting
- Source of funding bias

Trials will be classified as having a low risk of bias if they are graded as being at low risk of bias in all of these domains.

Two review authors will independently assess the risk of bias in all of the studies. Discrepancies will be resolved by discussion with a third author.

Assessment of Reporting Bias

Publication bias will be assessed by the visual assessment of funnel plots and Eggers test [44].

Measures of treatment Effect

For dichotomous variables, we will calculate the risk ratio (RR) with 95% confidence interval (CI). For continuous variables, we will calculate the mean difference (MD) with 95% CI for outcomes such as hospital

For peer review only

stay, and standardised mean difference (SMD) with 95% CI for quality of life (when different scales were used).

Dealing with missing data

We will perform an intention-to-treat analysis where possible. For dichotomous data presented only as percentages we will estimate frequencies using reported sample sizes for this outcome. For continuous outcomes if the mean and the standard deviation were not available from the trial report, we will seek this information from the trial authors. If this information is still not available, we will calculate the mean and standard deviation from median (interquartile ranges) using the software available in Review Manager Version 5.

Assessment of Heterogeneity

We anticipate that major sources of clinical heterogeneity will be associated with different patient groups (adults, children, congenital versus acquired disease), the use of different goal directed NIRS algorithms, the use of co-interventions such as restrictive transfusion thresholds, and differences in the methodology used to assess neurocognitive dysfunction. We will explore heterogeneity within each meta-analysis using a Chi^2 test with significance set at a P value of 0.10, and we expressed the percentage of heterogeneity due to variation rather than to chance as I^2 [45].We defined heterogeneity as follows:

I^2 0-40%: no or mild heterogeneity

I^2 40-80%: moderate heterogeneity

I^2 > 80%: sever heterogeneity

In the presence of severe heterogeneity meta-analysis will not be performed.

Data Synthesis

Meta-analyses will be performed using the software package Review Manager version 5.2 and in accordance with the recommendations of the *Cochrane Handbook for Systematic Reviews of Intervention* [46].

For the primary analysis we will compare the results of a random-effects model versus a fixed effects model to assess the effects of small studies. For continuous outcomes, we will pool mean differences or

For peer review only

standardised mean differences by using the inverse variance method. We will use the generic inverse variance method to pool hazard ratios.

Subgroup Analyses

Subgroup analyses will be performed in trials in which the Murkin algorithm [41] was used to guide goal directed therapy versus those that did not, and by participant group; CABG versus non CABG, adults versus children, assessment of neurocognitive function that incorporates test described in a previous consensus statement, and studies that combined the NIRS algorithm with a restrictive red cell transfusion trigger. Test for sub-group differences with Review Manager will be used with a P value of <0.05 considered statistically significant.

Sensitivity Analyses

Sensitivity analysis will exclude trials with unclear or high risk of bias for random sequence generation; unclear or high risk of bias due to lack of blinding of participants, healthcare providers or outcome assessors, and unclear or high risk of bias due to incomplete outcome data, and unclear of high risk of bias for source of funder.

Summary of findings

We will presentthe main results of the review in a 'Summary of findings' table. We will include the follwing outcomes.

- Risk of mortality
- Risk of stroke, myocardial infarction, or severe acute kidney injury
- Risk of red cell transfusion
- Neurocognitive Impairment
- Resource Use; ICU and hospital length of stay.

We will use GRADEpro software to prepare the 'Summary of findings'table. We will judge the overall quality of the evidence foreach outcome as 'high,' 'moderate,' 'low' or 'very low' accordingto the GRADE (Grades of Recommendation, Assessment, Developmentand Evaluation) approach [46].We will consider the following.

- Impact of risk of bias of individual trials.
- Precision of pooled estimate.Inconsistency or heterogeneity (clinical, methodological and statistical).

For peer review only

- Indirectness of evidence.
- Impact of selective reporting and publication bias on effect estimate.

- **COMPETING INTERESTS**

The authors declare that they have no competing interests.

AUTHORS' CONTRIBUTIONS

___ had full access to all of the data in the study and take responsibility for the integrity of the data and the accuracy of the data analysis.

Study concept and design: ___.

Acquisition of data: ___.

Analysis and interpretation of data: ______.

Drafting of the manuscript: ________.

Statistical analysis: _______

Study supervision: GJM.

For peer review only

REFERENCES

1. Murphy, G J and G D Angelini. Side effects of cardiopulmonary bypass: what is the reality? J Card Surg, 2004. 19(6): p. 481-488.
2. Murphy GJ,
3. Sapire, K J, S P Gopinath, G Farhat, D R Thakar, A Gabrielli, J W Jones, C S Robertson, and B Chance. Cerebral oxygenation during warming after cardiopulmonary bypass. Crit Care Med, 1997. 25(10): p. 1655-1662.
4. Yao, F S, C C Tseng, C Y Ho, S K Levin, and P Illner. Cerebral oxygen desaturation is associated with early postoperative neuropsychological dysfunction in patients undergoing cardiac surgery. J Cardiothorac Vasc Anesth, 2004. 18(5): p. 552-558.
5. Croughwell, N, M Lyth, T J Quill, M Newman, W J Greeley, L R Smith, and J G Reves. Diabetic patients have abnormal cerebral autoregulation during cardiopulmonary bypass. Circulation, 1990. 82(5 Suppl): p. IV407-412.
6. Miyoshi, S, T Morita, Y Kadoi, and F Goto. Analysis of the factors related to a decrease in jugular venous oxygen saturation in patients with diabetes mellitus during normothermic cardiopulmonary bypass. Surg Today, 2005. 35(7): p. 530-534.
7. Monitoring during CPB
8. Haisjackl, M, G Luz, H Sparr, R Germann, N Salak, B Friesenecker, E Deusch, S Meusburger, and W Hasibeder. The effects of progressive anemia on jejunal mucosal and serosal tissue oxygenation in pigs. Anesth Analg, 1997. 84(3): p. 538-544.
9. Leung, J M, R B Weiskopf, J Feiner, H W Hopf, S Kelley, M Viele, J Lieberman, J Watson, M Noorani, D Pastor, H Yeap, R Ho, and P Toy. Electrocardiographic ST-segment changes during acute, severe isovolemic hemodilution in humans. Anesthesiology, 2000. 93(4): p. 1004-1010.
10. Tsai, A G, P Cabrales, and M Intaglietta. Microvascular perfusion upon exchange transfusion with stored red blood cells in normovolemic anemic conditions. Transfusion, 2004. 44(11): p. 1626-1634.
11. Weiskopf, R B, J H Kramer, M Viele, M Neumann, J R Feiner, J J Watson, H W Hopf, and P Toy. Acute severe isovolemic anemia impairs cognitive function and memory in humans. Anesthesiology, 2000. 92(6): p. 1646-1652.
12. Kim, M B, D S Ward, C R Cartwright, J Kolano, S Chlebowski, and L C Henson. Estimation of jugular venous O2 saturation from cerebral oximetry or arterial O2 saturation during isocapnic hypoxia. J Clin Monit Comput, 2000. 16(3): p. 191-199.
13. Pollard, V, D S Prough, A E DeMelo, D J Deyo, T Uchida, and H F Stoddart. Validation in volunteers of a near-infrared spectroscope for monitoring brain oxygenation in vivo. Anesth Analg, 1996. 82(2): p. 269-277.
14. Alexander J.C.Jr, K M A, & Dance G.R. Reduced postoperative length of stay may result from using cerebral oximetry monitoring to guide treatment. The Annals of Thoracic Surgery, 2002. 73(1): p. S373.
15. Goldman, S, F Sutter, F Ferdinand, and C Trace. Optimizing intraoperative cerebral oxygen delivery using noninvasive cerebral oximetry decreases the incidence of stroke for cardiac surgical patients. Heart Surg Forum, 2004. 7(5): p. E376-381.
16. Murkin, J M, S J Adams, R J Novick, M Quantz, D Bainbridge, I Iglesias, A Cleland, B Schaefer, B Irwin, and S Fox. Monitoring brain oxygen saturation during coronary bypass surgery: a randomized, prospective study. Anesth Analg, 2007. 104(1): p. 51-58.
17. Variability Paper
18. Vincent, J L, J F Baron, K Reinhart, L Gattinoni, L Thijs, A Webb, A Meier-Hellmann, G Nollet, D Peres-Bota, and A B C Investigators. Anemia and blood transfusion in critically ill patients. JAMA, 2002. 288(12): p. 1499-1507.
19. Walsh, T S, M Garrioch, C Maciver, R J Lee, F MacKirdy, D B McClelland, J Kinsella, C Wallis, and G Audit of Transfusion in Intensive Care in Scotland Study. Red cell requirements for intensive care units adhering to evidence-based transfusion guidelines. Transfusion, 2004. 44(10): p. 1405-1411.

For peer review only

20. Casutt, M, B Seifert, T Pasch, E R Schmid, M I Turina, and D R Spahn. Factors influencing the individual effects of blood transfusions on oxygen delivery and oxygen consumption. Crit Care Med, 1999. 27(10): p. 2194-2200.
21. Weiskopf, R B, J Feiner, H Hopf, J Lieberman, H E Finlay, C Quah, J H Kramer, A Bostrom, and P Toy. Fresh blood and aged stored blood are equally efficacious in immediately reversing anemia-induced brain oxygenation deficits in humans. Anesthesiology, 2006. 104(5): p. 911-920.
22. Utoh, J, S Moriyama, K Okamoto, R Kunitomo, M Hara, and N Kitamura. The effects of cardiopulmonary bypass on postoperative oxygen metabolism. Surg Today, 1999. 29(1): p. 28-33.
23. Shann KG, Likosky DS, Murkin JM, Baker RA, Baribeau YR, DeFoe GR, Dickinson TA, Gardner TJ, Grocott HP, O'Connor GT, Rosinski DJ, Sellke FW, Willcox TW. An evidence-based review of the practice of cardiopulmonary bypass in adults: a focus on neurologic injury, glycemic control, hemodilution, and the inflammatory response. J Thorac Cardiovasc Surg. 2006 Aug;132(2):283-90.
24. DeFoe, G R, C S Ross, E M Olmstead, S D Surgenor, M P Fillinger, R C Groom, R J Forest, J W Pieroni, C S Warren, M E Bogosian, C F Krumholz, C Clark, R A Clough, P W Weldner, S J Lahey, B J Leavitt, C A Marrin, D C Charlesworth, P Marshall, and G T O'Connor. Lowest hematocrit on bypass and adverse outcomes associated with coronary artery bypass grafting. Northern New England Cardiovascular Disease Study Group. Ann Thorac Surg, 2001. 71(3): p. 769-776.
25. Fang, W C, R E Helm, K H Krieger, T K Rosengart, W J DuBois, C Sason, M L Lesser, O W Isom, and J P Gold. Impact of minimum hematocrit during cardiopulmonary bypass on mortality in patients undergoing coronary artery surgery. Circulation, 1997. 96(9 Suppl): p. II-194-199.
26. Von Heymann, C, M Sander, A Foer, A Heinemann, B Spiess, J Braun, M Kramer, J Grosse, P Dohmen, S Dushe, J Halle, W F Konertz, K D Wernecke, and C Spies. The impact of an hematocrit of 20% during normothermic cardiopulmonary bypass for elective low risk coronary artery bypass graft surgery on oxygen delivery and clinical outcome--a randomized controlled study. Crit Care, 2006. 10(2): p. R58.
27. Murpjhy GJ Audit
28. Denault A, Deschamps A, Murkin JM. A proposed algorithm for the intraoperative use of cerebral near-infrared spectroscopy. Semin Cardiothorac Vasc Anesth. 2007 Dec;11(4):274-81
29. Newman MF, Kirchner JL, Phillips-Bute, et al. Longitudinal assessment of neurocognitive function after coronary artery bypass surgery. New Engl J Med 2001;344:395–402.
30. Selnes OA, Goldsborough MA, Borowicz LM Jr, Enger C, Quaskey SA, McKhann GM. Determinants of cognitive change after coronary artery bypass graft surgery: a multi- factorial problem. Ann Thorac Surg 1999;67:1669 –76.
31. NIRS AKI
32. Mehta, R L, J A Kellum, S V Shah, B A Molitoris, C Ronco, D G Warnock, A Levin, and N Acute Kidney Injury. Acute Kidney Injury Network: report of an initiative to improve outcomes in acute kidney injury. Crit Care, 2007. 11(2): p. R31.
33. Murkin, J M, S P Newman, D A Stump, and J A Blumenthal. Statement of consensus on assessment of neurobehavioral outcomes after cardiac surgery. Ann Thorac Surg, 1995. 59(5): p. 1289-1295.
34. Mahanna, E P, J A Blumenthal, W D White, N D Croughwell, C P Clancy, L R Smith, and M F Newman. Defining neuropsychological dysfunction after coronary artery bypass grafting. Ann Thorac Surg, 1996. 61(5): p. 1342-1347.
35. SP Newman. Analysis and interpretation of neuropsychologic tests in cardiac surgery Ann Thorac Surg, 59 (1995), pp. 1351–1355
36. Spreen O., S, E. A compendium of neuropsychological tests. 1998, New York: Oxford University Press
37. Wechsler, D. Manual of the Wechsler Test of Adult Reading (WTAR-UK). 2001, New York Psychological Corporation (Iglesias)
38. Benton, A H, R. Multilingual Aphasia Examination. 1976, Iowa City: University of Iow
39. Murkin JM, Stump DA, Blumenthal JA, McKhann G. Defining dysfunction: group means versus incidence analysis-a statement of consensus. Ann Thorac Surg. 1997 Sep;64(3):904-5.
40. Higgins JPT, Deeks JJ. Chapter 7: Selecting studies and collecting data In: Higgins JPT,Green S (editors). Cochrane Handbook for Systematic Reviews of Interventions Version 5.1.0 [updated March 2011]. The Cochrane Collaboration, 2011. www.cochrane-handbook.org.

For peer review only

41. Denault A, Deschamps A, Murkin JM. A proposed algorithm for the intraoperative use of cerebral near-infrared spectroscopy. Semin Cardiothorac Vasc Anesth. 2007 Dec;11(4):274-81
42. Higgins JPT, Altman DG, Sterne JAC. Chapter 8: Assessing risk of bias in included studies. Cochrane Handbook for Systematic Reviews of Interventions Version 5.1.0 [updated March 2011]. The Cochrane Collaboration, 2011. www.cochrane-handbook.org.
43. Sterne JAC, Egger M, Moher D. Chapter 10: Addressing reporting biases In: Higgins JPT, Green S (editors). Cochrane Handbook for Systematic Reviews of Interventions Version 5.1.0 [updated March 2011]. The Cochrane Collaboration, 2011. www.cochranehandbook.org.
44. Higgins JPT, Thompson SG. Quantifying heterogeneity in a meta-analysis. Statistics in Medicine 2002;21(11):1539–58.
45. Higgins JPT, Green S (editors). Cochrane Handbook for Systematic Reviews of Interventions Version 5.1.0 [updated March 2011]. The Cochrane Colloboration, 2011. www.cochrane-handbook.org.
46. Schünemann HJ, Oxman AD, Vist GE, Higgins JPT, Deeks JJ, Glasziou P, et al.Chapter 12: Interpreting results and drawing conclusions. Higgins JPT, Green S(editors). Cochrane Handbook for Systematic Reviewsof Interventions Version 5.1.0 [updated March 2011].The Cochrane Collaboration, 2011. www.cochrane-handbook.org.

For peer review only

PRISMA Checklist:Efficacy of Near-Infrared Spectroscopy on the outcome of patients undergoing cardiac surgery: a systematic review of randomised trials.
Authors: GF Serraino, GJ Murphy

Section/topic	#	Checklist item	Reported on page #
**TITLE**			
Title	1	Identify the report as a systematic review, meta-analysis, or both.	1
Structured summary	2	Provide a structured summary including, as applicable: background; objectives; data sources; study eligibility criteria, participants, and interventions; study appraisal and synthesis methods; results; limitations; conclusions and implications of key findings; systematic review registration number.	2
Rationale	3	Describe the rationale for the review in the context of what is already known.	4
Objectives	4	Provide an explicit statement of questions being addressed with reference to participants, interventions, comparisons, outcomes, and study design (PICOS).	4
Protocol and registration	5	Indicate if a review protocol exists, if and where it can be accessed (e.g., Web address), and, if available, provide registration information including registration number.	4
Eligibility criteria	6	Specify study characteristics (e.g., PICOS, length of follow-up) and report characteristics (e.g., years considered, language, publication status) used as criteria for eligibility, giving rationale.	5
Information sources	7	Describe all information sources (e.g., databases with dates of coverage, contact with study authors to identify additional studies) in the search and date last	4

For peer review only - http://bmjopen.bmj.com/site/about/guidelines.xhtml

Section/topic	#	Checklist item	Reported on page #
		searched.	
Search	8	Present full electronic search strategy for at least one database, including any limits used, such that it could be repeated.	4
Study selection	9	State the process for selecting studies (i.e., screening, eligibility, included in systematic review, and, if applicable, included in the meta-analysis).	5
Data collection process	10	Describe method of data extraction from reports (e.g., piloted forms, independently, in duplicate) and any processes for obtaining and confirming data from investigators.	5
Data items	11	List and define all variables for which data were sought (e.g., PICOS, funding sources) and any assumptions and simplifications made.	5
Risk of bias in individual studies	12	Describe methods used for assessing risk of bias of individual studies (including specification of whether this was done at the study or outcome level), and how this information is to be used in any data synthesis.	5
Summary measures	13	State the principal summary measures (e.g., risk ratio, difference in means).	5
Synthesis of results	14	Describe the methods of handling data and combining results of studies, if done, including measures of consistency (e.g., I^2) for each meta-analysis.	5
Risk of bias across studies	15	Specify any assessment of risk of bias that may affect the cumulative evidence (e.g., publication bias, selective reporting within studies).	5
Additional analyses	16	Describe methods of additional analyses (e.g., sensitivity or subgroup analyses, meta-regression), if done, indicating which were pre-specified.	6
Study selection	17	Give numbers of studies screened, assessed for eligibility, and included in the review, with reasons for exclusions at each stage, ideally with a flow diagram.	6

For peer review only

Section/topic	#	Checklist item	Reported on page #
Study characteristics	18	For each study, present characteristics for which data were extracted (e.g., study size, PICOS, follow-up period) and provide the citations.	6
Risk of bias within studies	19	Present data on risk of bias of each study and, if available, any outcome-level assessment (see Item 12).	8
Results of individual studies	20	For all outcomes considered (benefits or harms), present, for each study: (a) simple summary data for each intervention group and (b) effect estimates and confidence intervals, ideally with a forest plot.	9
Synthesis of results	21	Present results of each meta-analysis done, including confidence intervals and measures of consistency.	10
Risk of bias across studies	22	Present results of any assessment of risk of bias across studies (see Item 15).	11
Additional analysis	23	Give results of additional analyses, if done (e.g., sensitivity or subgroup analyses, meta-regression [see Item 16]).	11
Summary of evidence	24	Summarize the main findings including the strength of evidence for each main outcome; consider their relevance to key groups (e.g., health care providers, users, and policy makers).	12
Limitations	25	Discuss limitations at study and outcome level (e.g., risk of bias), and at review level (e.g., incomplete retrieval of identified research, reporting bias).	13
Conclusions	26	Provide a general interpretation of the results in the context of other evidence, and implications for future research.	14
Funding	27	Describe sources of funding for the systematic review and other support (e.g.,	15

For peer review only

Section/topic	#	Checklist item	Reported on page #
		supply of data); role of funders for the systematic review.	

For peer review only
